# Supplementary material for: Neutralizing antibody correlate of protection against severe-critical COVID-19 in the ENSEMBLE single-dose Ad26.COV2.S vaccine efficacy trial
Source: Nat Commun. 2024 Nov 12;15:9785. doi: 10.1038/s41467-024-53727-y (PMC11557889; doi:10.1038/s41467-024-53727-y)
Supplement: Supplementary file 1 — Supplementary Information [file 41467_2024_53727_MOESM1_ESM.pdf]

Supplementary Material

Immune Assays Team

Affiliation	Team Members
Biomedical Advanced Research and Development Authority (BARDA), Washington, DC	Oleg Borisov, Flora Castellino, Brett Chromy, Mark Delvecchio, Ruben O. Donis, Tremel Faison, Corey Hoffman, Christopher Houchens, Tom Hu, Pennie Hylton, Lakshmi Jayashankar, Aparna Kolhekar, James Little, Karen Martins, Jeanne Novak, Azhar Ravji, Carol Sabourin, Evan Sturtevant, Kimberly Taylor, Xiaomi Tong, John Treanor, Danielle Turley, Leah Watson, Daniel Wolfe
Boston Consulting Group, Boston, MA	Gian King, Andrew Li, Najaf Shah, Smruthi Suryaprakash, Jue Xiang Wang
Division of AIDS, NIAID, NIH, Bethesda, MD	Patricia D'Souza
Division of MID (Microbiology and Infectious Diseases), NIAID, NIH, Bethesda, MD	Janie Russell
Duke University, Durham, NC	David Beaumont, Kendall Bradley, Jiayu Chen, Xiaoju Daniell, Thomas Denny, Elizabeth Domin, Amanda Eaton, Kelsey Engel, Wenhong Feng, Juanfei Gao, Hongmei Gao, Kelli Greene, Sarah Hiles, Leihua Liu, Kristy Long, Kellen Lund, Charlene McDanal, David C. Montefiori, Marcella Sarzotti-Kelsoe, Francesca Suman, Haili Tang, Jin Tong, Olivia Widman
LabCorp-Monogram Biosciences, South San Francisco, CA, USA	Christos J. Petropoulos, Terri Wrin
The Tauri Group, an LMI company - Contract Support for U.S. Department of Defense (DOD) Joint Program Executive Office for Chemical, Biological, Radiological and Nuclear Defense (JPEO-CBRND) Joint Project Manager for Chemical, Biological, Radiological, and Nuclear Medical (JPM CBRN Medical), Fort Detrick, Maryland, USA	Christopher S. Badorrek, Gregory E. Rutkowski
Vaccine Research Center, NIAID, NIH, Bethesda, MD	Obrimpong Amoa-Awua, Manjula Basappa, Robin Carroll, Britta Flach, Suprabhath Gajjala, Nazaire Jean-Baptiste, Richard A. Koup, Bob C. Lin, Adrian McDermott, Christopher Moore, Mursal Naisan, Muhammed Naqvi, Sandeep Narpala, Sarah O'Connell, Clare Whittaker, Weiwei Wu, Allen Mueller, Martin Apgar, Tommy Bruington, Joe Stashick, Leo Serebryannyy, Mike Castro, Jennifer Wang

Coronavirus Vaccine Prevention Network (CoVPN)/ENSEMBLE Team

Study Group Member	Affiliation	Location
Jerald Sadoff, M.D.	Janssen Vaccines and Prevention	Leiden, the Netherlands
Glenda Gray, M.B., B.Ch.	South African Research Council	Cape Town, South Africa
An Vandebosch, Ph.D.	Janssen Research and Development	Beerse, Belgium
Vicky Cárdenas, Ph.D.	Janssen Research and Development	Spring House, PA, USA
Georgi Shukarev, M.D.	Janssen Vaccines and Prevention	Leiden, the Netherlands
Beatriz Grinsztejn, M.D.	Evandro Chagas National Institute of Infectious Diseases–Fiocruz	Rio de Janeiro, Brazil
Paul A. Goepfert, M.D.	University of Alabama at Birmingham	Birmingham, AL, USA
Carla Truyers, Ph.D.	Janssen Research and Development	Beerse, Belgium
Hein Fennema, Ph.D.	Janssen Research and Development	Beerse, Belgium
Bart Spiessens, Ph.D.	Janssen Research and Development	Beerse, Belgium
Kim Offergeld, M.Sc.	Janssen Research and Development	Beerse, Belgium
Gert Scheper, Ph.D.	Janssen Vaccines and Prevention	Leiden, the Netherlands
Kimberly L. Taylor, Ph.D.	National Institute of Allergy and Infectious Diseases	Rockville, MD, USA
Merlin L. Robb, M.D.	Walter Reed Army Institute of Research	Silver Spring, MD, USA
John Treanor, M.D.	Biomedical Advanced Research and Development Authority	Washington, DC, USA
Dan H. Barouch, M.D.	Center for Virology and Vaccine Research, Beth Israel Deaconess Medical Center	Boston, MA, USA
Jeffrey Stoddard, M.D.	Janssen Research and Development	Raritan, NJ, USA
Martin F. Ryser, M.D.	Janssen Research and Development	Beerse, Belgium
Mary A. Marovich, M.D.	National Institute of Allergy and Infectious Diseases	Rockville, MD, USA
Kathleen M. Neuzil, M.D.	Center for Vaccine Development and Global Health, University of Maryland School of Medicine	Baltimore, MD, USA
Lawrence Corey, M.D.	Vaccine and Infectious Disease Division, Fred Hutchinson Cancer Research Center	Seattle, WA, USA
Nancy Cauwenberghs, Ph.D.	Janssen Research and Development	Beerse, Belgium
Tamzin Tanner, Ph.D.	Janssen Research and Development	Beerse, Belgium
Karin Hardt, Ph.D.	Janssen Research and Development	Beerse, Belgium

Javier Ruiz-Guiñazú, M.D.	Janssen Research and Development	Beerse, Belgium
Mathieu Le Gars, Ph.D.	Janssen Vaccines and Prevention	Leiden, the Netherlands
Hanneke Schuitemaker, Ph.D.	Janssen Vaccines and Prevention	Leiden, the Netherlands
Johan Van Hoof, M.D.	Janssen Vaccines and Prevention	Leiden, the Netherlands
Frank Struyf, M.D.	Janssen Research and Development	Beerse, Belgium
Macaya Douoguih, M.D.	Janssen Vaccines and Prevention	Leiden, the Netherlands
Richard Gorman, MD	Biomedical Advanced Research and Development Authority (BARDA)	Washington, DC, USA
Carmen A. Paez, MD, MBA	Fred Hutchinson Cancer Research Center	Seattle, WA, USA
Edith Swann, PhD	National Institute of Allergy and Infectious Diseases (NIAID)	Rockville, MD, USA
James Kublin, MD, MPH	Fred Hutchinson Cancer Research Center	Seattle, WA, USA
Simbarashe G. Takuva, MBChB, MSc	Fred Hutchinson Cancer Research Center; University of the Witwatersrand; University of Pretoria	Seattle, WA, USA; Johannesburg, South Africa; Pretoria, South Africa
Alex Greninger, MD, PhD, MS, MPhil	University of Washington; Fred Hutchinson Cancer Research Center	Seattle, WA, USA
Pavitra Roychoudhury, PhD	University of Washington; Fred Hutchinson Cancer Research Center	Seattle, WA, USA
Robert W. Coombs, MD, PhD	University of Washington	Seattle, WA, USA
Keith R. Jerome, MD, PhD	University of Washington; Fred Hutchinson Cancer Research Center	Seattle, WA, USA
Flora Castellino, MD	Biomedical Advanced Research and Development Authority (BARDA)	Washington, DC, USA
Xiaomi Tong, PhD	Biomedical Advanced Research and Development Authority (BARDA)	Washington, DC, USA
Corrina Pavetto, MS, RAC	Biomedical Advanced Research and Development Authority (BARDA)	Washington, DC, USA
Teletha Gipson, PhD, MS	Biomedical Advanced Research and Development Authority (BARDA)	Washington, DC, USA
Tina Tong, DrPH(c), MS, RAC(US)	National Institute of Allergy and Infectious Diseases (NIH/NIAID)	Rockville, MD, USA
Marina Lee, PhD	National Institute of Allergy and Infectious Diseases (NIH/NIAID)	Rockville, MD, USA
James Zhou, PhD, MS	Biomedical Advanced Research and Development Authority (BARDA)	Washington, DC, USA
Michael Fay, PhD	National Institute of Allergy and Infectious Diseases (NIH/NIAID)	Rockville, MD, USA

Kelly McQuarrie, BSN	Janssen Research & Development	Horsham, PA, USA
Chimeremma Nnadi, MD, PhD	Janssen Pharmaceuticals	Titusville, NJ, USA
Obiageli Sogbetun, MD, MPH	Janssen Infectious Disease and Vaccines	Titusville, NJ, USA
Nina Ahmad, MD	Janssen Pharmaceuticals	Titusville, NJ, USA
Ian De Proost, PhD	Johnson & Johnson	Beerse, Belgium
Cyrus Hoseyni, PhD	Janssen Research & Development	Spring House, PA, USA
Paul Coplan, ScD, MS, MBA	Johnson & Johnson Epidemiology	New Brunswick, NJ, USA
Najat Khan, PhD	Janssen Research & Development	New Brunswick, NJ, USA
Peter Ronco, BA	Janssen Research & Development	Raritan, NJ, USA
Dawn Furey, BA	Janssen Research & Development	Titusville, NJ, USA
Jodi Meck, MHA	Janssen Research & Development	Titusville, NJ, USA
Johan Vingerhoets, PhD	Janssen Pharmaceutica NV	Beerse, Belgium
Boerries Brandenburg, PhD	Janssen Vaccines & Prevention B.V.	Leiden, The Netherlands
Jerome Custers, PhD	Janssen Vaccines & Prevention B.V.	Leiden, The Netherlands
Jenny Hendriks, PhD	Janssen Vaccines & Prevention B.V.	Leiden, The Netherlands
Jarek Juraszek, PhD	Janssen Vaccines & Prevention B.V.	Leiden, The Netherlands
Anne Marit de Groot, PhD	Janssen Vaccines & Prevention B.V.	Leiden, The Netherlands
Griet Van Roey, PhD	Janssen Vaccines & Prevention B.V.	Leiden, The Netherlands
Dirk Heerwegh, PhD	Janssen Research & Development; Janssen	Beerse, Belgium
Ilse Van Dromme, PhD	Johnson & Johnson	Beerse, Belgium

CoVPN/ENSEMBLE Team, Cont'd: Participating Investigators

The following Principal Investigators participated in the ENSEMBLE study:

Name	Institute	Location
Aberg, Judith	Icahn School of Medicine at Mount Sinai	New York, NY, USA
Adams, Mark	Central Kentucky Research Associates, Inc.	Lexington, KY, USA
Adams, Michael	Synexus Clinical Research US, Inc.	Murray, UT, USA
Aguayo, Samuel	VA Medical Center	Phoenix, AZ, USA
Ahsan, Habibul	The University of Chicago Medicine	Chicago, IL, USA
Aizenberg, Diego	Centro Médico Viamonte SRL	Buenos Aires, Argentina
Alonto, Augusto	Jesse Brown VAMC Department of Surgery	Chicago, IL, USA
Alzogaray, Maria Fernanda	Instituto Médico Platense	Buenos Aires, Argentina
Anderson, Evan	Emory University School of Medicine	Atlanta, GA, USA
Andrade Pinto, Jorge	Hospital das Clínicas da Universidade Federal de Minas Gerais	Belo Horizonte, Brazil
Arns da Cunha, Clóvis	Hospital Nossa Senhora das Graças	Paraná, Brazil
Avelino Silva, Vivian	Hospital das Clínicas da Faculdade de Medicina da USP	São Paulo, Brazil
Badal-Faesen, Sharlaa	University of Witwatersrand – Helen Joseph Hospital – Themba Lethu HIV Research Centre	Johannesburg, South Africa
Baden, Lindsey	Brigham and Women's Hospital, Inc.	Boston, MA, USA
Barnabas, Shaun	Family Clinical Research Unit FAM-CRU	Western Cape, South Africa
Bedimo, Roger	AIDS Arms Incorporated Trinity Health and Wellness Center	Dallas, TX, USA
Bekker, Linda-Gail	Desmond Tutu HIV Centre, University of Cape Town	Cape Town, South Africa
Berhe, Mezgebe	North Texas Infectious Diseases Consultants	Dallas, TX, USA
Bessesen, Mary	Rocky Mountain Regional VA Medical Center	Aurora, CO, USA
Blaser, Martin J	Rutgers Robert Wood Johnson Medical School	New Brunswick, NJ, USA
Bonvehi, Pablo Eduardo	CEMIC Saavedra	Buenos Aires, Argentina
Borger, Judith	Carolina Institute for Clinical Research	Fayetteville, NC, USA

Brites Alves, Carlos Roberto	Fundação Bahiana de Infectologia	Bahia, Brazil
Brown, Sheldon	Bronx Veterans Affairs Medical Center	Bronx, NY, USA
Brumskine, William	The Aurum Institute Rustenburg Clinical Research Centre	Rustenburg, South Africa
Brune, Daniel	Optimal Research, LLC	Peoria, IL, USA
Buynak, Robert	Buynak Clinical Research	Valparaiso, IN, USA
Cabrera May, Carlos Antonio	Unidad de Atención Médica e Investigación en Salud (UNAMIS)	Yucatán, Mexico
Cadena Bonfanti, Andres Angelo	Clínica de la Costa	Barranquilla, Colombia
Cahn, Pedro	Fundación Huésped	Buenos Aires, Argentina
Carson, Jeffrey L.	Rutgers Robert Wood Johnson Medical School	New Brunswick, NJ, USA
Casapia Morales, Wilfredo Martin	Asociación Civil Selva Amazónica (ACSA)	Loreto, Peru
Cassetti, Lidia Isabel	Helios Salud S.A.	Buenos Aires, Argentina
Castex, Julie	Ochsner Medical Center	New Orleans, LA, USA
Chu, Laurence	Benchmark Research	Austin, TX, USA
Cotugno, Michael	Benchmark Research	Metairie, LA, USA
Creech, Clarence	Vanderbilt University Medical Center	Nashville, TN, USA
Crofoot, Gordon	CrofootMD Clinic and Research Center	Houston, TX, USA
Curtis, Brian	Corvallis Clinic PC	Corvallis, OR USA
da Silva Pilotto, José Henrique	Hospital Geral de Nova Igauçu	Rio de Janeiro, Brazil
Dal Ben Corradi, Mirian de Freitas	Hospital Sírio-Libanês	São Paulo, Brazil
Dal Pizzol, Felipe	Hospital São José	Santa Catarina, Brazil
Davis, Matthew	Rochester Clinical Research, Inc.	Rochester, NY, USA
De Carvalho Santana, Rodrigo	Hospital das Clínicas da Faculdade de Medicina de Ribeirão Preto daUSP	São Paulo, Brazil
de Faria Freire, Antônio Tarcísio	Santa Casa de Misericórdia de Belo Horizonte	Belo Horizonte, Brazil
DeJesus, Edwin	Orlando Immunology Center	Orlando, FL, USA
Delafontaine, Patrice	New Orleans Adolescent Trials Unit CRS	New Orleans, LA, USA
Delano Bronstein, Marcello	CPQuali Pesquisa Clínica LTDA ME	São Paulo, Brazil
Dell'Italia, Louis	University of Alabama at Birmingham	Birmingham, AL, USA

Deluca, Mercedes	Clinical Trials Division – Stamboulian Servicios de Salud	Buenos Aires, Argentina
Denham, Douglas	Clinical Trials of Texas, Inc.	San Antonio, TX, USA
Diacon, Andreas	TASK Central	Western Cape, South Africa
Dubula, Thozama	Nelson Mandela Academic Clinical Research Unit (NeMACRU)	Mthatha, South Africa
Eder, Frank	Meridian Clinical Research, LLC	Endwell, NY, USA
Edupuganti, Srilatha	The Hope Clinic at Emory University	Decatur, GA, USA
Ervin, John	The Center for Pharmaceutical Research	Kansas City, MO, USA
Esteves Coelho, Lara	FIO CRUZ – Fundação Oswaldo Cruz – Inst de Pesquisa Clínica Evandro Chagas	Rio de Janeiro, Brazil
Eudes Leal, Fabio	Universidade Municipal de São Caetano do Sul	São Paulo, Brazil
Fairlie, Lee	Shandukani Research Centre	Gauteng, South Africa
Fierro, Carlos	Johnson County Clin-Trials	Lenexa, KS, USA
Fogarty, Charles	Spartanburg Medical Research	Spartanburg, SC, USA
Fragoso, Veronica	Texas Center for Drug Development, Inc.	Houston, TX, USA
Frank, Ian	University of Pennsylvania	Philadelphia, PA, USA
Frey, Sharon	Saint Louis University	St Louis, MO, USA
Gallardo Cartagena, Jorge	Centro de Investigaciones Tecnológicas, Biomédicas y Medioambientales (CITBM)	Lima, Peru
Gamarra Ayarza, Cesar Augusto	Centro de Investigaciones Médicas	Callao, Peru
Garcia Diaz, Julia	Ochsner Medical Center	New Orleans, LA, USA
Gaur, Aditya	St Jude Children’s Research Hospital	Memphis, TN, USA
Gentile, Nina	Temple University Hospital	Philadelphia, PA, USA
Gill, Katherine	Masiphumelele Research Centre	Cape Town, South Africa
Gonzalez, Alexander	MedPlus Medicina Prepagadá S.A.	Bogotá, Colombia
Gottlieb, Robert	Baylor Scott & White Research Institute	Dallas, TX, USA
Grant, Philip	Stanford University Medical Center	Palo Alto, CA, USA
Greenberg, Richard	University of Kentucky	Lexington, KY, USA
Greiwe, Cathy	Synexus Clinical Research US, Inc.	Columbus, OH, USA

Guedes Barbosa, Luiz Sergio	Oncovida – Centro de Onco-Hematologia de Mato Grosso	Mato Grosso, Brazil
Han-Conrad, Laurie	WR-MCCR, LCC	San Diego, CA, USA
Hidalgo Vidal, Jose Alfredo	Asociación Civil Vía Libre	Lima, Peru
Higuera Cobos, Juan Diego	Fundación Oftalmológica de Santander – FOSCAL	Santander, Colombia
Hong, Matthew	Wake Research Associates	Raleigh, NJ, USA
Innes, James Craig	The Aurum Institute Klerksdorp Clinical Research Centre	Klerksdorp, South Africa
Jackson, Lisa	Kaiser Permanente Washington Health Research Institute	Seattle, WA, USA
Jackson-Booth, Peta-Gay	Optimal Research, LLC	Rockville, MD, USA
Jaller Raad, Juan Jose	Centro de Reumatología y Ortopedia	Barranquilla, Colombia
Jayaweera, Dushyantha	University of Miami – Miller School of Medicine	Miami, FL, USA
Jennings, William	Synexus Clinical Research US, Inc.	San Antonio, TX, USA
João Filho, Esaú Custódio	Hospital Federal dos Servidores do Estado	Rio de Janeiro, Brazil
Kassim, Sheetal	Desmond Tutu HIV Foundation – University of Cape Town	Cape Town, South Africa
Kennelly, Christina	Tryon Medical Partners	Charlotte, NC, USA
Khetan, Shishir	Meridian Clinical Research, LLC	Rockville, MD, USA
Kilgore, Paul E.	Henry Ford Health System	Detroit, MI, USA
Kim, Kenneth	Ark Clinical Research	Long Beach, CA, USA
Kirby, William	Synexus Clinical Research US, Inc.	Birmingham, AL, USA
Kopp, James	Synexus Clinical Research US, Inc.	Anderson, SC, USA
Kotze, Philip	Qhakaza Mbokodo Research Clinic	KwaZulu-Natal, South Africa
Kotze, Sheena	Stanza Clinical Research Centre: Mamelodi	Gauteng, South Africa
Kriesel, John	University of Utah	Salt Lake City, UT, USA
Kutner, Mark	Suncoast Research Group	Miami, FL, USA
Lacerda Nogueira, Maurício	Fundação Faculdade Regional de Medicina de São José do Rio Preto	São Paulo, Brazil
Laher, Fatima	Perinatal HIV Research Unit, Chris Hani Baragwanath Hospital	Gauteng, South Africa
Lama Valdivia, Javier Ricardo	Asociación Civil Impacta Salud y Educación – Barranco	Lima, Peru
Lazarus, Erica	Perinatal HIV Research Unit (PHRU), Kliptown	Soweto, South Africa

Lazcano Ponce, Eduardo Cesar	Instituto Nacional de Salud Pública	Morelos, Mexico
Leibman, Daniel	VA Medical Center	Columbia, SC, USA
Levin, Michael	Clinical Research Center of Nevada	Las Vegas, NV, USA
Levin, Myron	Children's Hospital Colorado	Aurora, CO, USA
Little, Susan	UCSD AntiViral Research Center (AVRC)	San Diego, CA, USA
Lombaard, Johannes	Josha Research	Bloemfontein, South Africa
Lopez Medina, Eduardo	Centro de Investigaciones Clínicas S.A.S.	Cali, Colombia
Losso, Marcelo	Hospital J.M. Ramos Mejía	Buenos Aires, Argentina
Luabeya, Angelique	SATVI, Brewelskloof Hospital	Western Cape, South Africa
Lucksinger, Gregg	Clinical Research Institute of Southern Oregon, P.C.	Medford, OR, USA
Lugogo, Njira	University of Michigan Neurosurgery A. Alfred Taubman Health CareCenter	Ann Arbor, MI, USA
Luz, Kleber Giovanni	Centro de Estudos e Pesquisas em Moléstias Infecciosas	Rio Grande do Norte, Brazil
Maboa, Rebone	Ndlovu Elandsdoorn Site	Limpopo, Dennilton, South Africa
Macareno Arroyo, Hugo Andres	Hospital Universidad del Norte	Barranquilla, Colombia
Makhaza, Disebo	CAPRISA Vulindlela Clinic	KwaZulu-Natal, South Africa
Malahleha, Mookho	Setshaba Research Centre	Soshanguve, South Africa
Malan, Daniel	PHOENIX Pharma (Pty) Ltd.	Eastern Cape, South Africa
Mamba, Musawenkosi	CRISMO Bertha Gxowa Research Centre	Gauteng, South Africa
Manning, Mary Beth	Rapid Medical Research	Cleveland, OH, USA
Martin, Judith	University of Pittsburgh	Pittsburgh, PA, USA
Mauricio da Silva, Cesar	Faculdade de Medicina Barretos – FACISB	Barretos, SP, Brazil
McGettigan, John	Quality of Life Medical & Research Center, LLC	Tucson, AZ, USA
Medrano Allende, Juan	Clínica y Maternidad Suizo Argentina	Buenos Aires, Argentina
Mena, Leandro	University of Mississippi Medical Center	Jackson, MS, USA
Messer, William	Oregon Health & Science University	Portland, OR, USA
Middleton, Randle	Optimal Research, LLC	Huntsville, AL, USA

Mills, Anthony	Anthony Mills Medical, Inc.	Los Angeles, CA, USA
Mills, Richard	PMG Research of Charleston, LLC	Mount Pleasant, SC, USA
Mngadi, Kathryn	The Aurum Institute: Tembisa – Clinic 4	Tembisa, South Africa
Moanna, Abeer	VA Medical Center – Atlanta	Decatur, GA, USA
Mofsen, Ricky	Massachusetts General Hospital	Boston, MA, USA
Moncada Vilela, Zandra	Hospital Nacional Arzobispo Loayza	Lima, Peru
Montaña, Oscar Romano	DIM Clínica Privada	Buenos Aires, Argentina
Moreno Hoyos Abril, Juan	Hospital Universitario de Nuevo León 'Dr. José Eleuterio González'	Nuevo León, Mexico
Morse, Caryn	Wake Forest Baptist Medical Center	Winston-Salem, NC, USA
Muñoz Reyes, Manuel	Hospital Dr. Hernán Henríquez Aravena	Araucanía, Chile
Murray, Linda	Synexus Clinical Research US, Inc.	Pinellas Park, FL, USA
Naicker, Nivashnee	Centre for the AIDS Programme of Research in South Africa	KwaZulu-Natal, South Africa
Naicker, Vimla	South Africa Medical Research Council	KwaZulu-Natal, South Africa
Naidoo, Logashvari	South African Medical Research Council Chatsworth Clinical ResearchSite	KwaZulu-Natal, South Africa
Nchabeleng, Maphoshane	MeCRU Clinical Research Unit	Gauteng, South Africa
Newman Lobato Souza, Tamara	Instituto de Infectologia Emilio Ribas	São Paulo, Brazil
Novak, Richard	University of Illinois at Chicago	Chicago, IL, USA
Nugent, Paul	Synexus Clinical Research US, Inc.	Cincinnati, OH, USA
O'Ryan Gallardo, Miguel Luis	Facultad de Medicina, Universidad de Chile	Santiago, Chile
Oyanguren Miranda, Martin	Hospital Nacional Edgardo Rebagliati Martins	Lima, Peru
Padala, Kalpana	Central Arkansas Veterans Healthcare System	Little Rock, AR, USA
Panettieri, Jr., Reynold A.	Rutgers Robert Wood Johnson Medical School	New Brunswick, NJ, USA
Panjwani, Sameer G.	Rush University Medical Center	Chicago, IL, USA
Patelli Juliani Souza Lima, Maria	Hospital e Maternidade Celso Pierro	São Paulo, Brazil
Pelkey, Leslie	Cherry Street Services, Inc.	Grand Rapids, MI, USA
Petrick, Friedrich	Mzansi Ethical Research Centre	Middelburg, South Africa
Pounds, Kevin	Synexus Clinical Research US, Inc.	Tucson, AZ, USA

Powell, Richard	New Horizons Clinical Research	Cincinnati, OH, USA
Pragalos, Antoinette	CTI Clinical Trial and Consulting Services	Cincinnati, OH, USA
Pratley, Richard E.	AdventHealth Orlando	Orlando, FL, USA
Presti, Rachel	Washington University School of Medicine	St Louis, MO, USA
Ramalho Madruga, José Valdez	Centro de Referência e Treinamento DST/AIDS	São Paulo, Brazil
Ramesh, Mayur	Henry Ford Health System	Detroit, MI, USA
Ramirez Sanchez, Isabel Cristina	Hospital Pablo Tobón Uribe	Antioquia, Colombia
Ramirez, Julio	University of Louisville	Louisville, KY, USA
Rankin, Bruce	Avail Clinical Research, LLC	DeLand, FL, USA
Restrepo, Jaime	Fundación Centro de Investigación Clinica (CIC)	Medellín, Colombia
Reynales Londono, Humberto	Centro de Atención e Investigación Médica S.A. – CAIMED	Bogotá, Colombia
Reynolds, Michele	Synexus Clinical Research US, Inc.	Dallas, TX, USA
Rhame, Frank	Abbott Northwestern Hospital Clinic	Minneapolis, MN, USA
Rhee, Margaret	Synexus Clinical Research US, Inc.	Akron, OH, USA
Riddle, Mark	VA Sierra Nevada Health Care System	Reno, NV, USA
Riegel Santos, Breno	Hospital Nossa Senhora da Conceição (HNSC)	Rio Grande do Sul, Brazil
Riffer, Ernie	Central Phoenix Medical Clinic	Phoenix, AZ, USA
Rizzardi, Barbara	Advanced Clinical Research	West Jordan, UT, USA
Rosso, Fernando	Fundación Valle del Lili	Valle del Cauca, Colombia
Saavedra, Carla	Bioclinica Santiago Bulnes	Santiago, Chile
Safirstein, Beth	MD Clinical	Hallandale Beach, FL, USA
Scapellato, Pablo	CEMEDIC	Buenos Aires, Argentina
Scheinberg, Phillip	Real e Benemérita Associação Portuguesa de Beneficência	São Paulo, Brazil
Schwartz, Howard	Research Centers of America, LLC	Hollywood, FL, USA
Sedillo, David J.	Rush University Medical Center	Chicago, IL, USA
Servilla, Karen	Raymond G. Murphy VA Medical Center	Albuquerque, NM, USA
Shah, Raj	Rush University Medical Center	Chicago, IL, USA

Siegel, Amy	MediSync Clinical Research	Petal, MS, USA
Silva Orellana, Rafael	Clínica del Maule	Talca, Chile
Silva, Federico	Fundación Cardiovascular de Colombia – Instituto del Corazón Floridablanca	Floridablanca, Colombia
Smith, Steven R.	AdventHealth Orlando	Orlando, FL, USA
Spooner, Elizabeth	South Africa Medical Research Council	KwaZulu-Natal, South Africa
Sprinz, Eduardo	Hospital de Clínicas de Porto Alegre	Porto Alegre, Brazil
Sriram, Peruvemba	North Florida/South Georgia Veterans Health System	Gainesville, FL, USA
Strout, Cynthia	Coastal Carolina Research Center, Inc.	Mount Pleasant, SC, USA
Swiatlo, Edwin	Southeast Louisiana Veterans Health Care System	New Orleans, LA, USA
Taiwo, Babafemi	Northwestern University	Evanston, IL, USA
Talwani, Rohit	Baltimore VA Medical Center	Baltimore, MD, USA
Tavares Russo, Luís Augusto	Instituto Brasil de Pesquisa Clínica	Rio de Janeiro, Brazil
Terront Lozano, Monica Alexandra	Solano y Terront Servicios Médicos Ltda	Bogotá, Colombia
Tharenos, Leslie	Synexus Clinical Research US, Inc.	St Louis, MO, USA
Tien, Phyllis	VA Medical Center	San Francisco, CA, USA
Tieu, Hong Van	New York Blood Center	New York, NY, USA
Toney, John	James A. Haley VA Hospital GNS	Tampa, FL, USA
Urbach, Dorothea	Synexus Helderberg Clinical Research Centre	Western Cape, South Africa
Vachris, Timothy	Optimal Research, LLC	Austin, TX, USA
Valencia, Javier	Asociación Civil Impacta Salud y Educación – San Miguel CRS	Lima, Peru
van Nieuwenhuizen, Elane	Synexus Watermeyer	Gauteng, South Africa
Vannucci Lomonte, Andrea	CEPIC – Centro Paulista de Investigação Clínica	São Paulo, Brazil
Vasconcellos, Eduardo	Instituto de Pesquisas Clínicas	Distrito Federal, Brazil
Velez, Ivan Dario	Programa de Estudio y Control de Enfermedades Tropicales	Medellín, Colombia
Ward, Amy	University of Cape Town IDM/CIDRI Research Site	Cape Town, South Africa
Wells, Cassia	Harlem Hospital Center	New York, NY, USA

White, Judith	Synexus Clinical Research US, Inc.	Orlando, FL, USA
Winkle, Peter	Anaheim Clinical Trials, LLC	Anaheim, CA, USA
Woods, Christopher	Durham VAMC	Raleigh, NC, USA
Zaidman, Cesar Javier	CIPREC	Buenos Aires, Argentina

United States Government (USG)/Coronavirus Prevention Network (CoVPN) Biostatistics Team

Affiliation	Team Members
Biomedical Advanced Research and Development Authority (BARDA), Washington, DC	Di Lu, James Zhou
Department of Biostatistics and Bioinformatics, Rollins School of Public Health, Emory University	David Benkeser, Sohail Nizam
Vaccine and Infectious Disease Division, Fred Hutchinson Cancer Center, Seattle, WA	Jessica Andriesen, Bhavesh Borate, Lindsay N. Carpp, Andrew Fiore-Gartland, Youyi Fong*, Peter B. Gilbert*, Ying Huang*, Yunda Huang*, Ollivier Hyrien, Holly E. Janes*, Michal Juraska, Yiwen Lu, April K. Randhawa, Brian D. Williamson*, Lars W.P. van der Laan, Chenchen Yu
Biostatistics Research Branch, NIAID, NIH, Bethesda, MD	Michael P. Fay, Jonathan Fintzi, Dean Follmann, Martha Nason
Clinical Monitoring Research Program Directorate, Frederick National Laboratory for Cancer Research, Frederick, MD	Eric Chu
Department of Biostatistics, University of Washington, Seattle, WA	Marco Carone, James Peng, Charlotte Talham
Department of Statistics, University of Washington, Seattle, WA	Alex Luedtke
Department of Biostatistics, Harvard T.H. Chan School of Public Health, Boston, MA	Nima S. Hejazi
Department of Population Health Sciences, NYU Grossman School of Medicine, New York, New York	Iván Díaz
Department of Biostatistics and Bioinformatics, Duke University; Global Health Institute, Duke University	Avi Kenny

*YF, PBG, YiH, and HEJ are also affiliated with the Department of Biostatistics, University of Washington, Seattle, WA. PBG and YuH are also affiliated with the Public Health Sciences Division, Fred Hutchinson Cancer Research Center, Seattle, WA. YuH is also affiliated with the Department of Global Health, University of Washington, Seattle, WA. Brian D. Williamson is also affiliated with Kaiser Permanente Washington Health Research Institute, Seattle, Washington, USA.

Supplementary Results

Latin America-specific results

Latin America: Response rates and levels
Similar to the results of the pooled analysis, in the Latin America cohort D29 antibody marker response frequencies and levels were lower in severe-critical cases than in non-cases, and to a lesser extent also lower in moderate cases than in non-cases. There was quantifiable D29 nAb-ID50 titer in 25.7% of vaccine recipient severe-critical cases and 40.7% of moderate cases, compared to 52.7% of vaccine recipient non-cases (Supplementary Fig. 4C). The geometric mean nAb-ID50 titers in vaccine recipient severe-critical cases, moderate cases, and non-cases were 3.77 IU50/ml (95% CI: 2.74, 5.20), 5.28 IU50/ml (4.60, 6.05), and 7.03 IU50/ml (5.85, 8.44), respectively, yielding a severe-critical case:non-case ratio of 0.54 (0.37, 0.78) and a moderate case:non-case ratio of 0.75 (0.60, 0.94) (Table 1). For both binding antibody markers, D29 antibody marker response rates and levels were also lower in cases than in non-cases for both endpoints, but with case:non-case ratios closer to 1 (Supplementary Fig. 4A, 4B; Table 1).

Latin America: CoR analyses (univariate)
Similar to the results of the pooled analysis, each D29 marker correlated inversely with risk in the Latin America cohort, with the D29 antibody markers potentially even stronger inverse CoRs of severe-critical COVID-19. The HR (High vs. Low nAb-ID50 tertile) of severe-critical COVID-19 was 0.12 (0.02, 0.57; FWER-adjusted overall p=0.048) (Supplementary Fig. 12D) and for moderate COVID-19 it was 0.43 (0.23, 0.82; FWER-adjusted overall p=0.133) (Supplementary Fig. 13D). Similar inverse correlations, although less strong, were also seen for both binding antibody markers and both COVID-19 endpoints (Supplementary Figs. 12D, S13D).

For the D29 quantitative markers, results were similar, with the D29 antibody markers potentially even stronger inverse CoRs of severe-critical COVID-19. The HR of severe-critical COVID-19 per 10-fold increase in nAb-ID50 was 0.20 (0.05, 0.73; FWER-adjusted p=0.048) (Supplementary Table 12), and for moderate COVID-19 it was 0.53 (0.32, 0.90; FWER-adjusted p=0.085) (Supplementary Table 12). Similar inverse correlations, although less strong, were also seen for both binding antibody markers and both COVID-19 endpoints (Supplementary Table 12).

As seen in the geographic region-pooled analysis, estimated cumulative incidence of severe-critical COVID-19 through 220 days decreased across vaccine recipient subgroups defined by having D29 antibody marker levels *at* a specific value. Cumulative incidence (95% CI) of severe-critical COVID-19 at unquantifiable nAb-ID50 titer [< 4.8975 International Units (IU)50/ml], just-quantifiable titer of 5.2 IU50/ml, and 90th percentile 28.5 IU50/ml was 1.2% (0.7%, 1.9%), 0.1% (0.01%, 1.5%), and 0.05% (0.01%, 0.4%), respectively. For moderate COVID-19, cumulative incidence at the same titer values of unquantifiable, 5.2 IU50/ml, and 28.5 IU50/ml was 8.0% (5.6%, 11.2%), 8.0% (4.4%, 11.2%), and 3.2% (1.4%, 6.8%), respectively (Supplementary Fig. 21C). A similar decrease in risk, albeit smaller in magnitude, was also seen with increasing concentration of each binding antibody marker (Supplementary Figs. 20A, 20B for severe-critical; 21A, 21B for moderate).

Vaccine recipients were also divided into subgroups defined by having D29 antibody marker levels *exceeding* a specific threshold, with application of nonparametric regression showing that the cumulative incidence of severe-critical COVID-19 (from 7 to 170 days post-D29) decreased with increasing antibody marker threshold. For vaccine recipients with any quantifiable nAb-ID50 (> 2.45 IU50/ml, with LLOQ =

4.8975 IU50/ml), risk was 0.45% (95% CI: 0.28%, 0.62%) (Supplementary Fig. 28C). This decreased to 0.34% (0.16%, 0.53%) for vaccine recipients with nAb-ID50 > 7.0 IU50/ml, with additional nAb-ID50 threshold increases yielding no further reduction of risk. No severe-critical COVID-19 endpoints were observed at nAb-ID50 titer above 185 IU50/ml. For moderate COVID-19, however, no decrease was seen, with cumulative incidence 15.8% (15.0%, 16.4%) for vaccine recipients with any quantifiable nAb-ID50 and with additional nAb-ID50 threshold increases yielding no further reduction of risk (Supplementary Fig. 29C). Similar decreases in severe-critical COVID-19 risk with increasing antibody marker threshold were seen for the two bAb markers (Supplementary Figs. 28A, 28B), especially RBD IgG (Supplementary Fig. 28B). Unlike the nAb-ID50 results, moderate COVID-19 risk decreased with bAb marker threshold (Supplementary Fig. 29A, 29B), again especially for RBD IgG (Supplementary Fig. 29B).

Latin America: CoR analyses (multivariate)

Using a Cox proportional hazards model that included baseline risk score, D29 nAb-ID50, and D29 Spike IgG, the HR of severe-critical COVID-19 per 10-fold-increase in nAb-ID50 was 0.14 (0.03, 0.60; p=0.008) compared to 1.92 (0.55, 6.75; p=0.310) per 10-fold increase in Spike IgG (Supplementary Table 16), again providing evidence for D29 nAb-ID50 as a better independent correlate of severe-critical COVID-19 than D29 Spike IgG. A similar result was seen for moderate COVID-19, where the HR was 0.56 (0.30, 1.02; p=0.059) and 0.92 (0.50, 1.70; p=0.796) per 10-fold increase in D29 nAb-ID50 and in D29 Spike IgG, respectively (Supplementary Table 16).

Latin America: CoP analyses

Vaccine efficacy (VE) against severe-critical COVID-19 increased with D29 antibody marker level. For nAb-ID50, estimated VE (95% CI) at unquantifiable nAb-ID50 titer [< 4.8975 International Units (IU)50/ml], just-quantifiable titer of 5.2 IU50/ml, and 90th percentile 28.5 IU50/ml was 58.4% (27.1%, 76.3%), 95.9% (41.1%, 99.4%), and 98.1% (55.8%, 99.4%), respectively, when using a nonparametric implementation of Gilbert et al.[1] (blue curve, Supplementary Fig. 36C). A similar increase was seen using an alternative Cox proportional hazards implementation of Gilbert et al.[1] (purple curve, Supplementary Fig. 36C). In comparison, VE estimates against moderate COVID-19 at the same titer values of unquantifiable, 5.2 IU50/ml, and 28.5 IU50/ml were 24.8% (-8.8%, 47.9%), 24.8% (-8.8%, 58.8%), and 70.1% (32.5%, 86.4%), respectively (Supplementary Fig. 37C). The severe-critical VE curves of the binding antibody markers were similar to the nAb-ID50 curve (Supplementary Fig. 37C), albeit with a smaller increase in estimated VE across the range of plotted marker values, e.g. a 22.3-fold increase in the amount of vaccine protection on the multiplicative scale for nAb-ID50 vs. a 2.4-fold increase for Spike IgG [calculated from estimated VE = 58.4% at unquantifiable nAb-ID50 titer (2.45 IU50/ml) to 98.1% at the 90th percentile (28.5 IU50/ml); for Spike IgG, estimated VE = 66.8% at negative response (5.42 BAU/ml) to 86.0% at the 90th percentile (121 BAU/ml); nonparametric curves].
Mediation analysis showed that an estimated 29.3% (1.8%, 56.8%) of VE against severe-critical COVID-19 was mediated by D29 nAb-ID50 titer (Supplementary Table 20), with a similar proportion, 23.7% (-24%, 71.4%), mediated by D29 Spike IgG concentration. In comparison, the estimated proportions of VE against moderate COVID-19 mediated by D29 nAb-ID50 titer and D29 Spike IgG concentration were 38.7% (-68.4%, 146%) and 107% (-51%, 265%), respectively (Supplementary Table 20).

Latin America: Synthesis scorecard and comparison of the D29 markers as correlates

Supplementary Table 23 presents the synthesis scorecard for the Latin America cohort for the D29 markers. In (A), Spike IgG was a better CoR and CoP against moderate COVID-19. RBD IgG was tied as a CoR against both endpoints and ranked as a better Modification CoP against severe-critical COVID-19,

but a better Mediation CoP against moderate COVID-19. nAb-ID50 ranked better as a CoR and as a Modification CoP against severe-critical COVID-19, but a better Mediation CoP against moderate COVID-19. In (B) (moderate to severe-critical), nAb-ID50 ranked as the best CoR and the best Modification CoP, while Spike IgG ranked as the best Mediation CoP. In (C) (severe-critical), nAb-ID50 ranked as the best CoR and CoP.

Latin America: Stochastic interventional vaccine efficacy (SVE)

We applied the stochastic-interventional VE (SVE) framework[2] to assess the D29 markers as CoPs against moderate to severe-critical COVID-19 in the Latin America cohort. For D29 nAb-ID50, estimated VE increased with shifts in titer: At no D29 nAb-ID50 shift, estimated SVE was 38.7% (95% CI: 34.3%, 42.8%), and with 1.6-fold, 4-fold, and 10-fold shifts, estimated SVE increased to 43.8% (37.8%, 49.2%), 54.5% (41.2%, 64.8%), and 59.4% (46.9%, 68.9%), respectively (Supplementary Fig. 50). The p-value for SVE changing with D29 nAb-ID50 was <0.001, providing further evidence in support of D29 nAb-ID50 as a CoP against moderate to severe-critical COVID-19. SVE estimates for the binding antibody markers (Spike IgG, RBD IgG) were not stable and are not shown.

Latin America: Exposure-proximal CoP analyses

Figure S54 shows the results of assessing each current marker as an exposure-proximal correlate of severe-critical COVID-19 in the Latin America cohort. Estimated exposure-proximal VE against severe-critical COVID-19 rose as current nAb-ID50 titer increased across the range of analyzed values (Supplementary Fig. 54C). Similar results were obtained for the two bAb markers (Supplementary Fig. 54A, B), except that the curves appeared less steep than the nAb-ID50 curve and had substantially wider 95% CIs at the left-end tail of each curve. Figure S55C shows that estimated VE against moderate COVID-19 also increased with current nAb-ID50 titer, with similar results for the binding antibody markers in the severe-critical COVID-19 analysis (somewhat flatter bAb vs. nAb curves, with wider 95% CIs on the bAb curves) (Supplementary Fig. S55A, B).

Supplementary Tables:

Supplementary Table 1. Numbers of baseline SARS-CoV-2 seronegative per-protocol vaccine recipients, shown by baseline sampling strata and COVID-19 case/non-case status, included in the analyses.

Case-cohort set = Baseline SARS-CoV-2 seronegative per-protocol vaccine recipients included in D29 marker correlates analysis [in the immunogenicity subcohort (IS) and/or a breakthrough COVID-19 case)]

		Baseline Sampling Strata of Baseline SARS-CoV-2 Seronegative Per-Protocol Vaccine Participants Included in Correlates Analyses																	Subtotals		
		1	2	3	4	5	6	7	8	9	10	11	12	13	14	15	16	Total	US	Lat Am	S Afr
Breakthrough COVID-19 cases (both within and outwith the IS) with D1, D29 Ab marker data	Moderate	6	5	0	1	28	9	5	7	147	59	26	26	4	8	0	1	332	61	258	13
	Severe-critical	2	0	0	0	1	1	0	2	17	6	0	8	3	2	0	0	42	6	31	5
	Moderate to severe-critical	8	5	0	1	29	10	5	9	163*	65	26	34	7	10	0	1	373	67	288	18
Non-cases in the IS with D1, D29 Ab marker data		49	51	41	55	47	54	47	52	50	48	49	50	43	40	46	52	774	396	197	181

Ab, antibody; IS, immunogenicity subcohort; Lat Am, Latin America; S Afr, South Africa; US, United States.

*Note: In stratum 9, the number of participants with a moderate to severe-critical case (163) is one less than the number of participants with a moderate case (147) plus the number of participants with a severe-critical case (17) due to one participant having two cases at different time points (first a moderate case, and later a severe-critical case).

Demographic covariate strata:

1. Minority U.S., age 18-59, comorbidities N
2. Minority U.S., age 18-59, comorbidities Y
3. Minority U.S., age ≥ 60, comorbidities N
4. Minority U.S., age ≥ 60, comorbidities Y
5. non-Minority U.S., age 18-59, comorbidities N
6. non-Minority U.S., age 18-59, comorbidities Y
7. non-Minority U.S., age ≥ 60, comorbidities N
8. non-Minority U.S., age ≥ 60, comorbidities Y
9. Latin America, age 18-59, comorbidities N
10. Latin America, age 18-59, comorbidities Y
11. Latin America, age ≥ 60, comorbidities N
12. Latin America, age ≥ 60, comorbidities Y
13. South Africa, age 18-59, comorbidities N
14. South Africa, age 18-59, comorbidities Y
15. South Africa, age ≥ 60, comorbidities N
16. South Africa, age ≥ 60, comorbidities Y

Minority is defined as the complement of being known to be White Non-Hispanic. Minority status was only reported in the U.S.
White Non-Hispanic is defined as Race=White and Ethnicity=Not Hispanic or Latino. All other Race subgroups are defined as Black, Asian, American Indian or Alaska Native, Native Hawaiian or Other Pacific Islander, Multiracial, Other, Not reported, or Unknown. (In Latin America, the American Indian or Alaska Native category was labeled as "Indigenous South American".)

Severe-critical cases are baseline SARS-CoV-2 seronegative per-protocol vaccine recipients with the severe-critical COVID-19 endpoint (severe-critical COVID-19 with onset that was both ≥ 28 days post-vaccination and ≥ 7 days post-D29) through to the data cut (July 9, 2021).
Moderate cases are baseline SARS-CoV-2 seronegative per-protocol vaccine recipients with the moderate COVID-19 endpoint (moderate COVID-19 with onset that was both ≥ 28 days post-vaccination and ≥ 7 days post-D29) through to the data cut (July 9, 2021).
Moderate to severe-critical cases are baseline SARS-CoV-2 seronegative per-protocol vaccine recipients with the moderate to severe-critical COVID-19 endpoint (moderate to severe-critical COVID-19 with onset that was both ≥ 28 days post-vaccination and ≥ 7 days post-D29) through to the data cut (July 9, 2021).
Non-cases are baseline seronegative per-protocol vaccine recipients sampled into the immunogenicity subcohort with no evidence of SARS-CoV-2 infection up to the end of the correlates study period, which is up to 220 days post D1 but no later than the data cut (July 9, 2021).

Supplementary Table 2. Demographics and clinical characteristics of baseline SARS-CoV-2 seronegative per-protocol trial participants in the immunogenicity subcohort and thus have D1, D29 antibody data. (all regions pooled)

Characteristics	Vaccine<br>(N = 839)	Placebo<br>(N = 91)	Total<br>(N = 930)
**Age**			
Age 18-59	418 (49.8%)	43 (47.3%)	461 (49.6%)
Age ≥ 60	421 (50.2%)	48 (52.7%)	469 (50.4%)
Mean (Range)	55.4 (18.0, 90.0)	57.3 (26.0, 88.0)	55.6 (18.0, 90.0)
**BMI**			
Underweight BMI < 18.5	19 (2.3%)	2 (2.2%)	21 (2.3%)
Normal 18.5 ≤ BMI < 25	235 (28.0%)	21 (23.1%)	256 (27.5%)
Overweight 25 ≤ BMI < 30	300 (35.8%)	34 (37.4%)	334 (35.9%)
Obese BMI ≥ 30	285 (34.0%)	34 (37.4%)	319 (34.3%)
**Risk for Severe COVID-19**			
At-risk	436 (52.0%)	45 (49.5%)	481 (51.7%)
Not at-risk	403 (48.0%)	46 (50.5%)	449 (48.3%)
**Age, Risk for Severe COVID-19**			
Age 18-59 At-risk	210 (25.0%)	21 (23.1%)	231 (24.8%)
Age 18-59 Not at-risk	208 (24.8%)	22 (24.2%)	230 (24.7%)
Age ≥ 60 At-risk	226 (26.9%)	24 (26.4%)	250 (26.9%)
Age ≥ 60 Not at-risk	195 (23.2%)	24 (26.4%)	219 (23.5%)
**Sex Assigned at Birth**			
Female	379 (45.2%)	37 (40.7%)	416 (44.7%)
Male	460 (54.8%)	54 (59.3%)	514 (55.3%)
**Hispanic or Latino Ethnicity**			
Hispanic or Latino	314 (37.4%)	33 (36.3%)	347 (37.3%)
Not Hispanic or Latino	498 (59.4%)	54 (59.3%)	552 (59.4%)
Not reported and unknown	27 (3.2%)	4 (4.4%)	31 (3.3%)
**Race**			
White	443 (52.8%)	49 (53.8%)	492 (52.9%)
Black or African American	246 (29.3%)	26 (28.6%)	272 (29.2%)
Asian	15 (1.8%)		15 (1.6%)
American Indian or Alaska Native	60 (7.2%)	6 (6.6%)	66 (7.1%)
Native Hawaiian or Other Pacific Islander	3 (0.4%)		3 (0.3%)
Multiracial	41 (4.9%)	7 (7.7%)	48 (5.2%)
Not reported and unknown	31 (3.7%)	3 (3.3%)	34 (3.7%)
**Underrepresented Minority Status in the U.S.***			
White Non-Hispanic	211 (25.1%)	24 (26.4%)	235 (25.3%)
Communities of Color	205 (24.4%)	24 (26.4%)	229 (24.6%)
**Country**			
United States	416 (49.6%)	48 (52.7%)	464 (49.9%)
Argentina	33 (3.9%)	2 (2.2%)	35 (3.8%)

Brazil	93 (11.1%)	12 (13.2%)	105 (11.3%)
Chile	11 (1.3%)	3 (3.3%)	14 (1.5%)
Colombia	55 (6.6%)	5 (5.5%)	60 (6.5%)
Mexico	7 (0.8%)	2 (2.2%)	9 (1.0%)
Peru	21 (2.5%)	1 (1.1%)	22 (2.4%)
South Africa	203 (24.2%)	18 (19.8%)	221 (23.8%)
**HIV Status**			
Negative	810 (96.5%)	89 (97.8%)	899 (96.7%)
Living with HIV	29 (3.5%)	2 (2.2%)	31 (3.3%)

*Data on minority status was only gathered in the U.S.

This table summarizes characteristics of per-protocol participants in the immunogenicity subcohort, which was randomly sampled from the study cohort. The sampling was stratified by strata defined by enrollment characteristics: Assigned randomization arm × Baseline SARS-CoV-2 seronegative vs. seropositive × Randomization strata. The U.S. subcohort includes 8 baseline demographic strata; the Latin America and South Africa subcohorts each include 4 baseline demographic strata.

Supplementary Table 3. Demographics and clinical characteristics of baseline SARS-CoV-2 seronegative per-protocol trial participants in the Latin America subset of the immunogenicity subcohort (strata 9-12) and thus have D1, D29 antibody data.

Characteristics	Vaccine (N = 220)	Placebo (N = 25)	Total (N = 245)
**Age**			
Age 18-59	108 (49.1%)	12 (48.0%)	120 (49.0%)
Age ≥ 60	112 (50.9%)	13 (52.0%)	125 (51.0%)
Mean (Range)	55.1 (19.0, 83.0)	56.6 (26.0, 88.0)	55.3 (19.0, 88.0)
**BMI**			
Underweight BMI < 18.5	1 (0.5%)		1 (0.4%)
Normal 18.5 ≤ BMI < 25	54 (24.5%)	6 (24.0%)	60 (24.5%)
Overweight 25 ≤ BMI < 30	95 (43.2%)	12 (48.0%)	107 (43.7%)
Obese BMI ≥ 30	70 (31.8%)	7 (28.0%)	77 (31.4%)
**Risk for Severe COVID-19**			
At-risk	112 (50.9%)	11 (44.0%)	123 (50.2%)
Not at-risk	108 (49.1%)	14 (56.0%)	122 (49.8%)
**Age, Risk for Severe COVID-19**			
Age 18-59 At-risk	54 (24.5%)	5 (20.0%)	59 (24.1%)
Age 18-59 Not at-risk	54 (24.5%)	7 (28.0%)	61 (24.9%)
Age ≥ 60 At-risk	58 (26.4%)	6 (24.0%)	64 (26.1%)
Age ≥ 60 Not at-risk	54 (24.5%)	7 (28.0%)	61 (24.9%)
**Sex Assigned at Birth**			
Female	83 (37.7%)	8 (32.0%)	91 (37.1%)
Male	137 (62.3%)	17 (68.0%)	154 (62.9%)
**Hispanic or Latino Ethnicity**			
Hispanic or Latino	209 (95.0%)	23 (92.0%)	232 (94.7%)
Not Hispanic or Latino	7 (3.2%)	2 (8.0%)	7 (2.9%)
Not reported and unknown	4 (1.8%)		6 (2.4%)
**Race**			
White	132 (60.0%)	14 (56.0%)	146 (59.6%)
Black or African American	8 (3.6%)	1 (4.0%)	9 (3.7%)
Asian	1 (0.5%)		1 (0.4%)
Multiracial	21 (9.5%)	3 (12.0%)	24 (9.8%)
Not reported and unknown	9 (4.1%)	2 (8.0%)	11 (4.5%)
**Country**			
Argentina	33 (15.0%)	2 (8.0%)	35 (14.3%)
Brazil	93 (42.3%)	12 (48.0%)	105 (42.9%)
Chile	11 (5.0%)	3 (12.0%)	14 (5.7%)
Colombia	55 (25.0%)	5 (20.0%)	60 (24.5%)
Mexico	7 (3.2%)	2 (8.0%)	9 (3.7%)
Peru	21 (9.5%)	1 (4.0%)	22 (9.0%)
**HIV Status**			
Negative	213 (96.8%)	25 (100.0%)	238 (97.1%)

Living with HIV	7 (3.2%)	7 (2.9%)

This table summarizes characteristics of per-protocol participants in the Latin America subset of the immunogenicity subcohort (strata 9-12), which was randomly sampled from the study cohort. The sampling was stratified by strata defined by enrollment characteristics: Assigned randomization arm × Baseline SARS-CoV-2 seronegative vs. seropositive × Randomization strata.

Supplementary Table 4. Demographics and clinical characteristics of baseline SARS-CoV-2 seronegative per-protocol trial participants in the South Africa subset of the immunogenicity subcohort (strata 13-16) and thus have D1, D29 antibody data.

Characteristics	Vaccine (N = 203)	Placebo (N = 18)	Total (N = 221)
**Age**			
Age 18-59	98 (48.3%)	6 (33.3%)	104 (47.1%)
Age ≥ 60	105 (51.7%)	12 (66.7%)	117 (52.9%)
Mean (Range)	55.8 (21.0, 84.0)	58.9 (33.0, 83.0)	56.1 (21.0, 84.0)
**BMI**			
Underweight BMI < 18.5	17 (8.4%)	1 (5.6%)	18 (8.1%)
Normal 18.5 ≤ BMI < 25	73 (36.0%)	3 (16.7%)	76 (34.4%)
Overweight 25 ≤ BMI < 30	49 (24.1%)	8 (44.4%)	57 (25.8%)
Obese BMI ≥ 30	64 (31.5%)	6 (33.3%)	70 (31.7%)
**Risk for Severe COVID-19**			
At-risk	104 (51.2%)	8 (44.4%)	112 (50.7%)
Not at-risk	99 (48.8%)	10 (55.6%)	109 (49.3%)
**Age, Risk for Severe COVID-19**			
Age 18-59 At-risk	49 (24.1%)	3 (16.7%)	52 (23.5%)
Age 18-59 Not at-risk	49 (24.1%)	3 (16.7%)	52 (23.5%)
Age ≥ 60 At-risk	55 (27.1%)	5 (27.8%)	60 (27.1%)
Age ≥ 60 Not at-risk	50 (24.6%)	7 (38.9%)	57 (25.8%)
**Sex Assigned at Birth**			
Female	101 (49.8%)	8 (44.4%)	109 (49.3%)
Male	102 (50.2%)	10 (55.6%)	112 (50.7%)
**Hispanic or Latino Ethnicity**			
Not Hispanic or Latino	195 (96.1%)	18 (100.0%)	213 (96.4%)
Not reported and unknown	8 (3.9%)		8 (3.6%)
**Race**			
White	40 (19.7%)	4 (22.2%)	44 (19.9%)
Black or African American	144 (70.9%)	12 (66.7%)	156 (70.6%)
Asian	1 (0.5%)		1 (0.5%)
Native Hawaiian or Other Pacific Islander	1 (0.5%)		1 (0.5%)
Multiracial	12 (5.9%)	2 (11.1%)	14 (6.3%)
Not reported and unknown	5 (2.5%)		5 (2.3%)
**Country**			
South Africa	203 (100.0%)	18 (100.0%)	221 (100.0%)
**HIV Status**			
Negative	186 (91.6%)	17 (94.4%)	203 (91.9%)
Living with HIV	17 (8.4%)	1 (5.6%)	18 (8.1%)

This table summarizes characteristics of per-protocol participants in the South Africa subset of the immunogenicity subcohort (strata 13-16), which was randomly sampled from the study cohort. The sampling was stratified by strata defined by enrollment characteristics: Assigned randomization arm × Baseline SARS-CoV-2 seronegative vs. seropositive × Randomization strata.

Supplementary Table 5. Demographics and clinical characteristics of baseline SARS-CoV-2 seronegative per-protocol trial participants in the United States subset of the immunogenicity subcohort (strata 1-8) and thus have D1, D29 antibody data.

Characteristics	Vaccine (N = 416)	Placebo (N = 48)	Total (N = 464)
**Age**			
Age 18-59	212 (51.0%)	25 (52.1%)	237 (51.1%)
Age ≥ 60	204 (49.0%)	23 (47.9%)	227 (48.9%)
Mean (Range)	55.4 (18.0, 90.0)	57.1 (26.0, 81.0)	55.6 (18.0, 90.0)
**BMI**			
Underweight BMI < 18.5	1 (0.2%)	1 (2.1%)	2 (0.4%)
Normal 18.5 ≤ BMI < 25	108 (26.0%)	12 (25.0%)	120 (25.9%)
Overweight 25 ≤ BMI < 30	156 (37.5%)	14 (29.2%)	170 (36.6%)
Obese BMI ≥ 30	151 (36.3%)	21 (43.8%)	172 (37.1%)
**Risk for Severe COVID-19**			
At-risk	220 (52.9%)	26 (54.2%)	246 (53.0%)
Not at-risk	196 (47.1%)	22 (45.8%)	218 (47.0%)
**Age, Risk for Severe COVID-19**			
Age 18-59 At-risk	107 (25.7%)	13 (27.1%)	120 (25.9%)
Age 18-59 Not at-risk	105 (25.2%)	12 (25.0%)	117 (25.2%)
Age ≥ 60 At-risk	113 (27.2%)	13 (27.1%)	126 (27.2%)
Age ≥ 60 Not at-risk	91 (21.9%)	10 (20.8%)	101 (21.8%)
**Sex Assigned at Birth**			
Female	195 (46.9%)	21 (43.8%)	216 (46.6%)
Male	221 (53.1%)	27 (56.2%)	248 (53.4%)
**Hispanic or Latino Ethnicity**			
Hispanic or Latino	105 (25.2%)	10 (20.8%)	115 (24.8%)
Not Hispanic or Latino	296 (71.2%)	36 (75.0%)	332 (71.6%)
Not reported and unknown	15 (3.6%)	2 (4.2%)	17 (3.7%)
**Race**			
White	271 (65.1%)	31 (64.6%)	302 (65.1%)
Black or African American	94 (22.6%)	13 (27.1%)	107 (23.1%)
Asian	13 (3.1%)		13 (2.8%)
American Indian or Alaska Native	11 (2.6%)	1 (2.1%)	12 (2.6%)
Native Hawaiian or Other Pacific Islander	2 (0.5%)		2 (0.4%)
Multiracial	8 (1.9%)	2 (4.2%)	10 (2.2%)
Not reported and unknown	17 (4.1%)	1 (2.1%)	18 (3.9%)
**Underrepresented Minority Status in the U.S.**			
White Non-Hispanic	211 (50.7%)	24 (50.0%)	235 (50.6%)
Communities of Color	205 (49.3%)	24 (50.0%)	229 (49.4%)
**Country**			
United States	416 (100.0%)	48 (100.0%)	464 (100.0%)
**HIV Status**			

Negative	411 (98.8%)	47 (97.9%)	458 (98.7%)
Living with HIV	5 (1.2%)	1 (2.1%)	6 (1.3%)

This table summarizes characteristics of per-protocol participants in the United States subset of the immunogenicity subcohort (strata 1-8), which was randomly sampled from the study cohort. The sampling was stratified by strata defined by enrollment characteristics: Assigned randomization arm × Baseline SARS-CoV-2 seronegative vs. seropositive × Randomization strata.

Supplementary Table 6. Symptoms of the 42 severe-critical COVID-19 cases in the vaccine group included in the analyses.

**Symptom**	**No. of Severe-Critical COVID-19 Cases (Vaccine Group)**
Shortness of breath	24
Respiratory Rate ≥ 20 breaths/minute	9
Abnormal saturation of oxygen (SpO2) but still >93%	22
Clinical or radiologic evidence of pneumonia OR COVID-19 pneumonia	5
Radiologic evidence of DVT	1
Highest temperature was ≥38.0 ºC	16
Heart rate ≥ 90 beats/minute	30
Shaking chills or rigors OR Chills or uncontrollable body shaking/shivering	19
Cough	28
Sore throat	28
Malaise	34
Headache	28
Myalgia OR Muscle aches/pains	26
Gastrointestinal symptoms OR Diarrhea, vomiting, nausea, abdominal/stomach pain	22
Anosmia (olfactory or taste disorders) OR Decreased sense of smell or Decreased sense of taste	23
Chilblains/pernio (red or bruised looking feet or toes) OR Red or bruised looking feet or toes	3
Clinical signs at rest indicative of severe systemic illness (respiratory rate ≥30 breaths/minute, heart rate ≥125 beats/minute, oxygen saturation (SpO2) ≤93% on room air at sea level, or partial pressure of oxygen/fraction of inspired oxygen (PaO2/FiO2) <300 mmHg OR One of vital signs abnormality occurred: SpO2 ≤ 93%, Heart rate ≥125 beats/minute, Respiratory Rate ≥30 breaths/minute	38
Respiratory failure (defined as needing high-flow oxygen, non-invasive ventilation, mechanical ventilation, or extracorporeal membrane oxygenation [ECMO] OR One of vital signs abnormality occurred: SpO2 ≤ 93%, Heart rate ≥125 beats/minute, Respiratory Rate ≥30 breaths/minute	1
Shock (defined as systolic blood pressure <90 mmHg, diastolic blood pressure <60 mmHg, or requiring vasopressors)	0
Significant acute renal, hepatic or neurologic dysfunction	4
Fatality	0

Supplementary Table 7. Numbers of symptoms per severe-critical COVID-19 case (vaccine group) for the 42 severe-critical cases included in the analysis. A list of the 21 symptoms is provided in Supplementary Table 6.

No. of Symptoms (of the 21 in Supplementary Table 6)	No. of Severe-Critical COVID-19 Cases (Vaccine Group)
≤ 3	8
4 to 6	3
7 to 9	5
10 to 12	21
13 to 15	5
≥ 16	0

Supplementary Table 8. D1 and D29 geometric mean titers (GMTs) and geometric mean concentrations (GMCs) for baseline SARS-CoV-2 seronegative vaccine recipients in the immunogenicity subcohort, shown separately by sex.

**Sex**	Visit	Arm	Baseline SARS-CoV-2	Marker	N	GMT/GMC (95% CI)	
Male	Day 1	Vaccine	Negative	Anti RBD IgG (BAU/ml)	461	7.35	(7.14, 7.58)
Male	Day 1	Vaccine	Negative	Anti Spike IgG (BAU/ml)	461	5.62	(5.43, 5.81)
Male	Day 1	Vaccine	Negative	nAb-ID50 (IU50/ml)	461	1.46	(1.39, 1.54)
Male	Day 29	Vaccine	Negative	Anti RBD IgG (BAU/ml)	461	30.30	(27.38, 33.53)
Male	Day 29	Vaccine	Negative	Anti Spike IgG (BAU/ml)	461	31.82	(28.38, 35.66)
Male	Day 29	Vaccine	Negative	nAb-ID50 (IU50/ml)	461	4.61	(3.98, 5.35)
Female	Day 1	Vaccine	Negative	Anti RBD IgG (BAU/ml)	383	7.38	(7.12, 7.65)
Female	Day 1	Vaccine	Negative	Anti Spike IgG (BAU/ml)	383	5.63	(5.43, 5.84)
Female	Day 1	Vaccine	Negative	nAb-ID50 (IU50/ml)	383	1.40	(1.37, 1.42)
Female	Day 29	Vaccine	Negative	Anti RBD IgG (BAU/ml)	383	34.95	(30.70, 39.78)
Female	Day 29	Vaccine	Negative	Anti Spike IgG (BAU/ml)	383	36.52	(31.76, 42.00)
Female	Day 29	Vaccine	Negative	nAb-ID50 (IU50/ml)	383	5.31	(4.45, 6.33)

BAU, binding antibody units; IU, international units; nAb-ID50, 50% inhibitory dilution neutralizing antibody titer.

Supplementary Table 9. Ratios (Males/Females) of D1 and D29 geometric mean titers (GMTs) and geometric mean concentrations (GMCs) for baseline SARS-CoV-2 seronegative vaccine recipients in the immunogenicity subcohort. Source values for the ratios are in Supplementary Table 8.

Visit	Arm	Baseline SARS-CoV-2	Marker	Ratio (Male/Female) of GMT or GMC (95% CI)
Day 1	Vaccine	Negative	Anti RBD IgG (BAU/ml)	1.00 (0.95, 1.04)
Day 1	Vaccine	Negative	Anti Spike IgG (BAU/ml)	1.00 (0.95, 1.05)
Day 1	Vaccine	Negative	nAb-ID50 (IU50/ml)	1.05 (1.00, 1.10)
Day 29	Vaccine	Negative	Anti RBD IgG (BAU/ml)	0.87 (0.73, 1.02)
Day 29	Vaccine	Negative	Anti Spike IgG (BAU/ml)	0.87 (0.73, 1.04)
Day 29	Vaccine	Negative	nAb-ID50 (IU50/ml)	0.87 (0.69, 1.10)

BAU, binding antibody units; IU, international units; nAb-ID50, 50% inhibitory dilution neutralizing antibody titer.

Supplementary Table 10. Limits of the Spike IgG binding antibody assay, the RBD binding antibody assay, and the pseudovirus neutralizing antibody assay.

MSD Binding Assay (VRC)		
Reported units	BAU/ml	
	Spike	RBD
Positivity Cutoff	10.8424	14.0858
LOD	0.3076	1.5936
LLOQ	1.8429	5.0243
ULOQ	238.1165	172.5755
All values < Positivity Cutoff were set to Positivity Cutoff/2		
All values > ULOQ were set to ULOQ (for immune correlates analyses)		

Pseudovirus neutralization ID50 titer (Monogram)	
Reported units	IU50/ml
LOD	N/A
LLOQ	4.8975
ULOQ	844.7208
All values < LLOQ were set to LLOQ/2	
All values > ULOQ were set to ULOQ (for immune correlates analyses)	

BAU = binding antibody units; IU = International Units; LLOQ, lower limit of quantitation; LOD, limit of detection; RBD, receptor binding domain; ULOQ, upper limit of quantitation.

Supplementary Table 11. Covariate-adjusted hazard ratios of severe-critical COVID-19, moderate COVID-19, or moderate to severe-critical COVID-19 per 10-fold increase or per standard deviation increase in each D29 antibody marker. Analyses were based on baseline SARS-CoV-2 seronegative per-protocol vaccine recipients and adjusted for baseline behavioral risk score and geographic region.

			**Severe-Critical COVID-19**					
D29 Marker	No. cases/No. at-risk	HR per 10-fold increase Pt. Est.	95% CI	p-value (2-sided)	FDR-adj p-value	FWER-adj p-value	HR per SD increase Pt. Est.	95% CI
Spike IgG (BAU/ml)	46/18,163	0.67	0.32, 1.39	0.285	0.619	0.567	0.83	0.59, 1.17
RBD IgG (BAU/ml)	46/18,163	0.79	0.33, 1.85	0.583	0.633	0.793	0.90	0.63, 1.30
nAb-ID50 (IU50/ml)	46/18,163	0.35	0.13, 0.90	0.030	0.106	0.098	0.59	0.36, 0.95
			**Moderate COVID-19**					
D29 Marker	No. cases/No. at-risk*	HR per 10-fold increase Pt. Est.	95% CI	p-value (2-sided)	FDR-adj p-value**	FWER-adj p-value**	HR per SD increase Pt. Est.	95% CI
Spike IgG (BAU/ml)	375/18,163	0.67	0.45, 1.01	0.057	0.114	0.146	0.83	0.69, 1.01
RBD IgG (BAU/ml)	375/18,163	0.59	0.37, 0.93	0.025	0.073	0.076	0.80	0.65, 0.97
nAb-ID50 (IU50/ml)	375/18,163	0.53	0.34, 0.82	0.005	0.052	0.031	0.73	0.58, 0.91
			**Moderate to Severe-Critical COVID-19**					
D29 Marker	No. cases/No. at-risk	HR per 10-fold increase Pt. Est.	95% CI	p-value (2-sided)	FDR-adj p-value	FWER-adj p-value	HR per SD increase Pt. Est.	95% CI
Spike IgG (BAU/ml)	420/18,163	0.67	0.45, 1.00	0.048	0.103	0.128	0.83	0.69, 1.00
RBD IgG (BAU/ml)	420/18,163	0.60	0.38, 0.95	0.028	0.082	0.084	0.80	0.66, 0.98
nAb-ID50 (IU50/ml)	420/18,163	0.50	0.32, 0.78	0.002	0.026	0.016	0.71	0.57, 0.88

*No. at-risk = estimated number in the population for analysis, i.e. baseline negative per-protocol vaccine recipients not experiencing the designated COVID-19 endpoint or infected through 6 days post Day 29 visit; no. cases = number of this cohort with an observed designated COVID-19 endpoint (calculated via inverse probability of sampling Day 29 marker weighting).
**q-value and FWER (family-wide error rate) are computed over the set of p-values both for quantitative markers and categorical markers using the Westfall and Young permutation method (10000 replicates).
p-values were obtained using a two-sided Wald test.
Cases were counted starting 7 days post Day 29.

BAU, antibody binding units; CI, confidence interval; FDR, false discovery rate; FWER, family-wise error rate; HR, hazard ratio; IU, international units; nAb-ID50, 50% inhibitory dilution neutralizing antibody; Pt. Est., point estimate; RBD, receptor binding domain; SD, standard deviation.

Supplementary Table 12. Covariate-adjusted hazard ratios of severe-critical COVID-19, moderate COVID-19, or moderate to severe-critical COVID-19 per 10-fold increase or per standard deviation increase in each D29 antibody marker in participants in Latin America. Analyses were based on baseline SARS-CoV-2 seronegative per-protocol vaccine recipients in Latin America and adjusted for baseline behavioral risk score.

				**Severe-Critical COVID-19**				
D29 Marker	No. cases/No. at-risk	HR per 10-fold increase Pt. Est.	95% CI	p-value (2-sided)	FDR-adj p-value	FWER-adj p-value	HR per SD increase Pt. Est.	95% CI
Spike IgG (BAU/ml)	34/7,694	0.70	0.29, 1.66	0.415	0.774	0.753	0.84	0.56, 1.27
RBD IgG (BAU/ml)	34/7,694	0.75	0.26, 2.11	0.580	0.783	0.875	0.88	0.57, 1.37
nAb-ID50 (IU50/ml)	34/7,694	0.20	0.05, 0.73	0.015	0.047	0.048	0.45	0.24, 0.86
				**Moderate COVID-19**				
D29 Marker	No. cases/No. at-risk*	HR per 10-fold increase Pt. Est.	95% CI	p-value (2-sided)	FDR-adj p-value**	FWER-adj p-value**	HR per SD increase Pt. Est.	95% CI
Spike IgG (BAU/ml)	290/7,694	0.67	0.40, 1.10	0.114	0.197	0.262	0.83	0.65, 1.05
RBD IgG (BAU/ml)	290/7,694	0.55	0.31, 0.99	0.047	0.138	0.135	0.78	0.61, 1.00
nAb-ID50 (IU50/ml)	290/7,694	0.53	0.32, 0.90	0.018	0.138	0.085	0.74	0.57, 0.95
				**Moderate to Severe-Critical COVID-19**				
D29 Marker	No. cases/No. at-risk	HR per 10-fold increase Pt. Est.	95% CI	p-value (2-sided)	FDR-adj p-value	FWER-adj p-value	HR per SD increase Pt. Est.	95% CI
Spike IgG (BAU/ml)	323/7,694	0.66	0.40, 1.09	0.106	0.207	0.254	0.83	0.65, 1.04
RBD IgG (BAU/ml)	323/7,694	0.56	0.32, 1.00	0.052	0.151	0.143	0.79	0.62, 1.00
nAb-ID50 (IU50/ml)	323/7,694	0.49	0.29, 0.82	0.007	0.062	0.040	0.70	0.54, 0.91

*No. at-risk = estimated number in the population for analysis, i.e. baseline negative per-protocol vaccine recipients in Latin America not experiencing the designated COVID-19 endpoint or infected through 6 days post Day 29 visit; no. cases = number of this cohort with an observed designated COVID-19 endpoint (calculated via inverse probability of sampling Day 29 marker weighting).
**q-value and FWER (family-wide error rate) are computed over the set of p-values both for quantitative markers and categorical markers using the Westfall and Young permutation method (10000 replicates).
p-values were obtained using a two-sided Wald test.
Cases were counted starting 7 days post Day 29.

BAU, antibody binding units; CI, confidence interval; FDR, false discovery rate; FWER, family-wise error rate; HR, hazard ratio; IU, international units; nAb-ID50, 50% inhibitory dilution neutralizing antibody; Pt. Est., point estimate; RBD, receptor binding domain; SD, standard deviation.

Supplementary Table 13. Covariate-adjusted hazard ratios of moderate to severe-critical COVID-19 per 10-fold increase or per standard deviation increase in each D29 antibody marker in participants in South Africa. Analyses were based on baseline SARS-CoV-2 seronegative per-protocol vaccine recipients in South Africa and adjusted for baseline behavioral risk score. Hazard ratios of severe-critical COVID-19 could not be assessed due to too few severe-critical cases in South Africa.

		**Moderate to Severe-Critical COVID-19**							
D29 Marker	No. cases/No. at-risk	HR per 10-fold increase Pt. Est.	95% CI	p-value (2-sided)	FDR-adj p-value	FWER-adj p-value	HR per SD increase Pt. Est.	95% CI
Spike IgG (BAU/ml)	22/2,214	0.74	0.26, 2.17	0.590	0.905	0.952	0.87	0.53, 1.43
RBD IgG (BAU/ml)	22/2,214	0.87	0.20, 3.83	0.858	0.931	0.998	0.94	0.50, 1.77
nAb-ID50 (IU50/ml)	22/2,214	0.78	0.30, 2.00	0.608	0.905	0.952	0.88	0.53, 1.45

*No. at-risk = estimated number in the population for analysis, i.e. baseline negative per-protocol vaccine recipients in South Africa not experiencing the designated COVID-19 endpoint or infected through 6 days post Day 29 visit; no. cases = number of this cohort with an observed designated COVID-19 endpoint (calculated via inverse probability of sampling Day 29 marker weighting).
**q-value and FWER (family-wide error rate) are computed over the set of p-values both for quantitative markers and categorical markers using the Westfall and Young permutation method (10000 replicates).
p-values were obtained using a two-sided Wald test.
Cases were counted starting 7 days post Day 29.

BAU, antibody binding units; CI, confidence interval; FDR, false discovery rate; FWER, family-wise error rate; HR, hazard ratio; IU, international units; nAb-ID50, 50% inhibitory dilution neutralizing antibody; Pt. Est., point estimate; RBD, receptor binding domain; SD, standard deviation.

Supplementary Table 14. Covariate-adjusted hazard ratios of moderate to severe-critical COVID-19 per 10-fold increase or per standard deviation (SD) increase in each D29 antibody marker in participants in the United States. Analyses were based on baseline SARS-CoV-2 seronegative per-protocol vaccine recipients in the United States and adjusted for baseline behavioral risk score. Hazard ratios of severe-critical COVID-19 could not be assessed due to too few severe-critical cases in the United States.

		**Moderate to Severe-Critical COVID-19**						
D29 Marker	No. cases/No. at-risk	HR per 10-fold increase Pt. Est.	95% CI	p-value (2-sided)	FDR-adj p-value	FWER-adj p-value	HR per SD increase Pt. Est.	95% CI
Spike IgG (BAU/ml)	75/8,255	0.63	0.37, 1.10	0.103	0.205	0.253	0.81	0.62, 1.04
RBD IgG (BAU/ml)	75/8,255	0.67	0.37, 1.22	0.188	0.297	0.396	0.84	0.65, 1.09
nAb-ID50 (IU50/ml)	75/8,255	0.51	0.26, 0.98	0.045	0.169	0.150	0.71	0.51, 0.99

*No. at-risk = estimated number in the population for analysis, i.e. baseline negative per-protocol vaccine recipients in the United States not experiencing the designated COVID-19 endpoint or infected through 6 days post Day 29 visit; no. cases = number of this cohort with an observed designated COVID-19 endpoint (calculated via inverse probability of sampling Day 29 marker weighting).
**q-value and FWER (family-wide error rate) are computed over the set of p-values both for quantitative markers and categorical markers using the Westfall and Young permutation method (10000 replicates).
p-values were obtained using a two-sided Wald test.
Cases were counted starting 7 days post Day 29.

BAU, antibody binding units; CI, confidence interval; FDR, false discovery rate; FWER, family-wise error rate; HR, hazard ratio; IU, international units; nAb-ID50, 50% inhibitory dilution neutralizing antibody; Pt. Est., point estimate; RBD, receptor binding domain; SD, standard deviation.

Supplementary Table 15. Covariate-adjusted hazard ratios, assessed using multivariable models, of moderate COVID-19, severe-critical COVID-19, or moderate to severe-critical COVID-19 per 10-fold increase in each D29 antibody marker. Analyses were based on baseline SARS-CoV-2 seronegative per-protocol vaccine recipients and adjusted for baseline behavioral risk score and geographic region.

	**Severe-Critical COVID-19**		**Moderate COVID-19**		**Moderate to Severe-Critical COVID-19**	
	Hazard Ratio (95% CI)	P-value	Hazard Ratio (95% CI)	P-value	Hazard Ratio (95% CI)	P-value
Risk score	2.27 (1.28, 4.04)	0.005	1.59 (1.16, 2.19)	0.004	1.66 (1.21, 1.27)	0.002
Region: Latin America*	1.56 (0.50, 4.82)	0.441	1.82 (1.19, 2.79)	0.006	1.79 (1.18, 2.71)	0.006
Region: South Africa*	2.57 (0.77, 8.55)	0.124	0.65 (0.36, 1.16)	0.145	0.81 (0.49, 1.34)	0.412
Spike IgG (BAU/ml)	1.22 (0.49, 3.02)	0.674	0.92 (0.57, 1.50)	0.741	0.94 (0.58, 1.52)	0.807
nAb-ID50 (IU50/ml)	0.31 (0.11, 0.89)	0.029	0.55 (0.33, 0.92)	0.023	0.52 (0.31, 0.86)	0.011

*Reference region = United States.
Maximum failure event time 181 days (moderate COVID-19), 170 days (severe-critical COVID-19), or 181 days (moderate to severe-critical COVID-19) post D29. Cases were counted starting 7 days post D29.
P-values are unadjusted and were obtained using a two-sided Wald test.
BAU, antibody binding units; CI, confidence interval; IU, international units; nAb-ID50, 50% inhibitory dilution neutralizing antibody.

Supplementary Table 16. Covariate-adjusted hazard ratios, assessed using multivariable models, of moderate COVID-19, severe-critical COVID-19, or moderate to severe-critical COVID-19 per 10-fold increase in each D29 antibody marker in participants in Latin America. Analyses were based on baseline SARS-CoV-2 seronegative per-protocol vaccine recipients in Latin America and adjusted for baseline behavioral risk score.

	**Severe-Critical COVID-19**		**Moderate COVID-19**		**Moderate to Severe-Critical COVID-19**	
	Hazard Ratio (95% CI)	P-value	Hazard Ratio (95% CI)	P-value	Hazard Ratio (95% CI)	P-value
Risk score	2.36 (1.16, 4.83)	0.018	1.41 (1.01, 1.98)	0.046	1.49 (1.06, 2.10)	0.023
Spike IgG (BAU/ml)	1.92 (0.55, 6.75)	0.310	0.92 (0.50, 1.70)	0.796	0.98 (0.53, 1.81)	0.950
nAb-ID50 (IU50/ml)	0.14 (0.03, 0.60)	0.008	0.56 (0.30, 1.02)	0.059	0.49 (0.27, 0.91)	0.024

Maximum failure event time 181 days (moderate COVID-19), 170 days (severe-critical COVID-19), or 181 days (moderate to severe-critical COVID-19) post D29. Cases were counted starting 7 days post D29.
P-values are unadjusted and were obtained using a two-sided Wald test.
BAU, antibody binding units; CI, confidence interval; IU, international units; nAb-ID50, 50% inhibitory dilution neutralizing antibody.

Supplementary Table 17. Covariate-adjusted hazard ratios, assessed using multivariable models, of moderate to severe-critical COVID-19 per 10-fold increase in each D29 antibody marker in participants in South Africa. Analyses were based on baseline SARS-CoV-2 seronegative per-protocol vaccine recipients in South Africa and adjusted for baseline behavioral risk score. Hazard ratios of severe-critical COVID-19 could not be assessed due to too few severe-critical cases in South Africa.

	**Moderate to Severe-Critical COVID-19**	
	Hazard Ratio (95% CI)	P-value
Risk score	4.32 (1.40, 13.34)	0.011
Spike IgG (BAU/ml)	0.81 (0.24, 2.80)	0.744
nAb-ID50 (IU50/ml)	0.86 (0.30, 2.47)	0.777

Maximum failure event time 101 days (same for moderate COVID-19 and for moderate to severe-critical COVID-19) post D29. Cases were counted starting 7 days post D29.
P-values are unadjusted and were obtained using a two-sided Wald test.
BAU, antibody binding units; CI, confidence interval; IU, international units; nAb-ID50, 50% inhibitory dilution neutralizing antibody.

Supplementary Table 18. Covariate-adjusted hazard ratios, assessed using multivariable models, of moderate to severe-critical COVID-19 per 10-fold increase in each D29 antibody marker in participants in the United States. Analyses were based on baseline SARS-CoV-2 seronegative per-protocol vaccine recipients in the United States and adjusted for baseline behavioral risk score. Hazard ratios of severe-critical COVID-19 could not be assessed due to too few severe-critical cases in the United States.

	**Moderate to Severe-Critical COVID-19**	
	Hazard Ratio (95% CI)	P-value
Risk score	3.49 (1.55, 7.87)	0.003
Spike IgG (BAU/ml)	0.79 (0.42, 1.48)	0.466
nAb-ID50 (IU50/ml)	0.58 (0.29, 1.17)	0.127

Maximum failure event time 109 days (same for moderate COVID-19 and for moderate to severe-critical COVID-19) post D29. Cases were counted starting 7 days post D29.
P-values are unadjusted and were obtained using a two-sided Wald test.
BAU, antibody binding units; CI, confidence interval; IU, international units; nAb-ID50, 50% inhibitory dilution neutralizing antibody.

Supplementary Table 19. Mediation effect estimates for D29 quantitative markers with 95% confidence intervals.

Non-marker mediated VE = VE comparing vaccine vs. placebo with antibody marker set to value if assigned placebo.

Marker-mediated VE = VE in vaccinated comparing observed antibody marker vs. hypothetical marker had the participant received placebo.

Prop. Mediated = fraction of total risk reduction from vaccine attributed to the antibody marker.

**VE Against Severe-Critical COVID-19**			
	Non-Marker Mediated VE (95% CI)	Marker Mediated VE (95% CI)	Prop. Mediated (95% CI)
Spike IgG (BAU/ml)	0.667 (0.191, 0.863)	0.297 (-0.349, 0.634)	0.243 (-0.214, 0.700)
RBD IgG (BAU/ml)	0.696 (0.257, 0.876)	0.228 (-0.497, 0.602)	0.179 (-0.282, 0.639)
nAb-ID50 (IU50/ml)	0.645 (0.311, 0.817)	0.340 (0.166, 0.477)	0.286 (0.085, 0.487)
**VE Against Moderate COVID-19**			
	Non-Marker Mediated VE (95% CI)	Marker Mediated VE (95% CI)	Prop. Mediated (95% CI)
Spike IgG (BAU/ml)	-0.013 (-0.605, 0.361)	0.365 (0.031, 0.583)	1.03 (-0.022, 2.08)
RBD IgG (BAU/ml)	0.063 (-0.410, 0.377)	0.313 (0.002, 0.527)	0.852 (-0.051, 1.76)
nAb-ID50 (IU50/ml)	0.196 (-0.048, 0.383)	0.199 (0.017, 0.348)	0.505 (0.008, 1.00)
**VE Against Moderate to Severe-Critical COVID-19**			
	Non-Marker Mediated VE (95% CI)	Marker Mediated VE (95% CI)	Prop. Mediated (95% CI)
Spike IgG (BAU/ml)	0.160 (-0.311, 0.461)	0.364 (0.057, 0.572)	0.723 (0.051, 1.39)
RBD IgG (BAU/ml)	0.220 (-0.164, 0.478)	0.315 (0.025, 0.519)	0.603 (0.015, 1.19)
nAb-ID50 (IU50/ml)	0.307 (0.090, 0.472)	0.229 (0.065, 0.365)	0.415 (0.088, 0.742)

BAU, antibody binding units; IU, international units; nAb-ID50, 50% inhibitory dilution neutralizing antibody.
Overall VE (95% CI) against the severe-critical, moderate, and moderate to severe-critical endpoints starting 7 days post-D29 through 220 days post-vaccination was 73.1% (58.7%, 84.1%), 41.3% (28.6%, 51.3%), and 48.6% (38.6%, 57.0%), respectively. Proportion mediated is not a true proportion in that it can take values outside of [0, 1]. Proportion mediated = 1 is equivalent to Non-marker-mediated VE = 0% and Proportion mediated = 0 is equivalent to Non-marker-mediated VE = Overall VE.

Supplementary Table 20. Mediation effect estimates for participants in Latin America for D29 quantitative markers with 95% confidence intervals.

Non-marker mediated VE = VE comparing vaccine vs. placebo with antibody marker set to value if assigned placebo.

Marker-mediated VE = VE in vaccinated comparing observed antibody marker vs. hypothetical marker had the participant received placebo.

Prop. mediated = fraction of total risk reduction from vaccine attributed to the antibody marker.

**VE Against Severe-Critical COVID-19**			
	Non-Marker Mediated VE (95% CI)	Marker Mediated VE (95% CI)	Prop. Mediated (95% CI)
Spike IgG (BAU/ml)	0.693 (0.107, 0.895)	0.308 (-0.426, 0.664)	0.237 (-0.240, 0.714)
RBD IgG (BAU/ml)	0.710 (0.124, 0.904)	0.269 (-0.574, 0.661)	0.202 (-0.300, 0.705)
nAb-ID50 (IU50/ml)	0.666 (0.174, 0.865)	0.365 (0.106, 0.549)	0.293 (0.018, 0.568)
**VE Against Moderate COVID-19**			
	Non-Marker Mediated VE (95% CI)	Marker Mediated VE (95% CI)	Prop. Mediated (95% CI)
Spike IgG (BAU/ml)	-0.022 (-0.654, 0.368)	0.282 (-0.123, 0.541)	1.07 (-0.505, 2.65)
RBD IgG (BAU/ml)	0.091 (-0.513, 0.454)	0.193 (-0.315, 0.504)	0.691 (-0.894, 2.28)
nAb-ID50 (IU50/ml)	0.173 (-0.220, 0.440)	0.113 (-0.229, 0.360)	0.387 (-0.684, 1.46)
**VE Against Moderate to Severe-Critical COVID-19**			
	Non-Marker Mediated VE (95% CI)	Marker Mediated VE (95% CI)	Prop. Mediated (95% CI)
Spike IgG (BAU/ml)	0.178 (-0.371, 0.507)	0.280 (-0.130, 0.541)	0.625 (-0.271, 1.52)
RBD IgG (BAU/ml)	0.263 (-0.274, 0.573)	0.197 (-0.321, 0.512)	0.418 (-0.534, 1.37)
nAb-ID50 (IU50/ml)	0.339 (-0.051, 0.585)	0.104 (-0.307, 0.386)	0.209 (-0.516, 0.935)

BAU, antibody binding units; IU, international units; nAb-ID50, 50% inhibitory dilution neutralizing antibody In Latin America, overall VE (95% CI) against the severe-critical, moderate, and moderate to severe-critical endpoints starting 7 days post-D29 through 220 days post-vaccination was 72.3% (95% CI 55.9 to 83.6%), 28.4% (95% CI 13.9 to 41.1%), and 38.5% (95% CI 26.7 to 49.0%), respectively. Proportion mediated is not a true proportion in that it can take values outside of [0, 1]. Proportion mediated = 1 is equivalent to Non-marker-mediated VE = 0% and Proportion mediated = 0 is equivalent to Non-marker-mediated VE = Overall VE.

Supplementary Table 21. Mediation effect estimates for participants in South Africa for D29 quantitative markers with 95% confidence intervals.

Non-marker mediated VE = VE comparing vaccine vs. placebo with antibody marker set to value if assigned placebo.
Marker-mediated VE = VE in vaccinated comparing observed antibody marker vs. hypothetical marker had the participant received placebo.
Prop. mediated = fraction of total risk reduction from vaccine attributed to the antibody marker.

**VE Against Moderate to Severe-Critical COVID-19**			
	Non-Marker Mediated VE (95% CI)	Marker Mediated VE (95% CI)	Prop. Mediated (95% CI)
Spike IgG (BAU/ml)	0.454 (-0.666, 0.821)	0.289 (-0.959, 0.742)	0.361 (-0.722, 1.44)
RBD IgG (BAU/ml)	0.276 (-1.01, 0.739)	0.464 (-0.311, 0.781)	0.659 (-0.345, 1.66)
nAb-ID50 (IU50/ml)	0.564 (0.158, 0.775)	0.109 (-0.379, 0.425)	0.122 (-0.344, 0.589)

BAU, antibody binding units; IU, international units; nAb-ID50, 50% inhibitory dilution neutralizing antibody.
In South Africa, overall VE (95% CI) against the s moderate to severe-critical endpoint starting 7 days post-D29 through 220 days post-vaccination was 61.2% (95% CI 37.3 to 77.0%). Proportion mediated is not a true proportion in that it can take values outside of [0, 1]. Proportion mediated = 1 is equivalent to Non-marker-mediated VE = 0% and Proportion mediated = 0 is equivalent to Non-marker-mediated VE = Overall VE.

Supplementary Table 22. Mediation effect estimates for participants in the United States for D29 quantitative markers with 95% confidence intervals.

Non-marker mediated VE = VE comparing vaccine vs. placebo with antibody marker set to value if assigned placebo.
Marker-mediated VE = VE in vaccinated comparing observed antibody marker vs. hypothetical marker had the participant received placebo.
Prop. mediated = fraction of total risk reduction from vaccine attributed to the antibody marker.

**VE Against Moderate to Severe-Critical COVID-19**			
	Non-Marker Mediated VE (95% CI)	Marker Mediated VE (95% CI)	Prop. Mediated (95% CI)
Spike IgG (BAU/ml)	0.256 (-1.14, 0.742)	0.524 (-0.117, 0.797)	0.715 (-0.240, 1.67)
RBD IgG (BAU/ml)	-0.075 (-3.40, 0.737)	0.671 (-0.341, 0.919)	1.07 (-0.290, 2.43)
nAb-ID50 (IU50/ml)	0.559 (0.267, 0.735)	0.196 (-0.072, 0.397)	0.210 (-0.096, 0.517)

BAU, antibody binding units; IU, international units; nAb-ID50, 50% inhibitory dilution neutralizing antibody.
In the United States, overall VE (95% CI) against the s moderate to severe-critical endpoint starting 7 days post-D29 through 220 days post-vaccination was 65.8% (95% CI 55.2 to 75.0%). Proportion mediated is not a true proportion in that it can take values outside of [0, 1]. Proportion mediated = 1 is equivalent to Non-marker-mediated VE = 0% and Proportion mediated = 0 is equivalent to Non-marker-mediated VE = Overall VE.

Supplementary Table 23. Scorecard for ranking D29 antibody marker performance in the Latin America cohort in each of two categories of immune correlate-quality criteria.

			**Category 1: Correlate of Risk (CoR)**					**Category 2: Correlate of Protection (CoP): VE Modification**							**Category 3: CoP: VE Modification**				
			HR per SD (Cox, quant.)		HR High vs. Low tertile (Cox)			Fold-increase in CVE[1] (Cox)		Fold-increase in CVE[1] (NP)		E-val. Marg. RR Pt. Est			Proportion mediated				
Comparison	D29 Marker	COVID-19 endpoint	Pt. Est. (95% CI)	Rank	Pt. Est. (95% CI)	Rank	**Cat. 1 Mean rank**	Fold-increase	Rank	Fold-increase	Rank	E-val.	Rank	**Cat. 2 Mean rank**	Pt. Est. (95% CI)	Rank	95% LCL	Rank	**Cat. 3 Mean rank**
	Spike IgG (BAU/ml)	Sev-crit	0.84 (0.56, 1.27)	2	0.66 (0.24, 1.82)	2	**2**	1.6	2	2.4	1	2.4	2	**1.67**	0.237 (-0.240, 0.714)	2	-0.240	NA	**2**
A	Spike IgG (BAU/ml)	Mod	0.83 (0.65, 1.05)	1	0.62 (0.35, 1.10)	1	**1***	1.7	1	2.0	2	2.5	1	**1.33***	1.07 (-0.505, 2.65)	1	-0.505	NA	**1***
	RBD IgG (BAU/ml)	Sev-crit	0.88 (0.57, 1.37)	2	0.56 (0.19, 1.67)	1	**1.5***	1.4	2	4.1	1	3.0	1	**1.33***	0.202 (-0.300, 0.705)	2	-0.300	NA	**2**
A	RBD IgG (BAU/ml)	Mod	0.78 (0.61, 1.00)	1	0.59 (0.33, 1.04)	2	**1.5***	2.0	1	1.7	2	2.7	2	**1.67**	0.691 (-0.894, 2.28)	1	-0.894	NA	**1***
	nAb-ID50 (IU50/ml)	Sev-crit	0.45 (0.24, 0.86)	1	0.12 (0.02, 0.57)	1	**1***	5.6	1	22.3	1	16.2	1	**1***	0.293 (0.018, 0.568)	2	0.018	NA	**2**
A	nAb-ID50 (IU50/ml)	Mod	0.74 (0.57, 0.95)	2	0.43 (0.23, 0.82)	2	**2**	1.9	2	2.5	2	3.9	2	**2**	0.387 (-0.684, 1.46)	1	-0.684	NA	**1***
	Spike IgG (BAU/ml)	Mod to sev-crit	0.83 (0.65, 1.04)	3	0.62 (0.35, 1.08)	3	**3**	1.7	3	2.0	2	2.5	3	**2.67**	0.625 (-0.271, 1.52)	1	-0.271	1	**1***
B	RBD IgG (BAU/ml)	Mod to sev-crit	0.79 (0.62, 1.00)	2	0.58 (0.33, 1.02)	2	**2**	1.9	2	1.8	3	2.8	2	**2.33**	0.418 (-0.534, 1.37)	2	-0.534	3	**2.5**
	nAb-ID50 (IU50/ml)	Mod to sev-crit	0.70 (0.54, 0.91)	1	0.39 (0.20, 0.74)	1	**1***	2.1	1	3.8	1	4.4	1	**1***	0.209 (-0.516, 0.935)	3	-0.516	2	**2.5**
	Spike IgG (BAU/ml)	Sev-crit	0.84 (0.56, 1.27)	2	0.66 (0.24, 1.82)	3	**2.5**	1.6	2	2.4	3	2.4	3	**2.67**	0.237 (-0.240, 0.714)	2	-0.240	2	**2**
C	RBD IgG (BAU/ml)	Sev-crit	0.88 (0.57, 1.37)	3	0.56 (0.19, 1.67)	2	**2.5**	1.4	3	4.1	2	3.0	2	**2.33**	0.202 (-0.300, 0.705)	3	-0.300	3	**3**
	nAb-ID50 (IU50/ml)	Sev-crit	0.45 (0.24, 0.86)	1	0.12 (0.02, 0.57)	1	**1***	5.6	1	22.3	1	16.2	1	**1***	0.293 (0.018, 0.568)	1	0.018	1	**1***

[1]Fold-increase calculated on the [1-VE(unquantifiable or negative)]/[1-VE(90th percentile)] scale

*Antibody marker(s) with the best performance, within each comparison, within each category.

Bold font indicates the three categories of criteria and the mean D29 antibody marker rank within each category.

Baseline covariates adjusted for: Baseline risk score. Maximum failure event time 181 days (moderate COVID-19), 170 days (severe-critical COVID-19), or 181 days (moderate to severe-critical COVID-19) post D29. Cases were counted starting 7 days post D29. All serological assay readouts were expressed in WHO International Standard units (see Methods). The Proportion mediated is not a true proportion in that it can take values outside of the interval [0, 1]. Proportion mediated = 1 is equivalent to Non-marker-mediated VE = 0% and Proportion mediated = 0 is equivalent to Non-marker-mediated VE = Overall VE. BAU, binding antibody units; Cat., category; CI, confidence interval; Comp., comparison; E-val., E-value; HR, hazard ratio; IU, international units; LCL, lower confidence limit; Marg., marginalized; mod., moderate; nAb-ID50, 50% inhibitory dilution neutralizing antibody titer; NP, nonparametric; Pt. Est., point estimate; RR, risk ratio; SD, standard deviation; sev-crit, severe-critical.

Supplementary Fig. 1. (A) Relationship between moderate, severe-critical, and moderate to severe-critical COVID-19 cases with the immunogenicity subcohort. **(B)** Phases of the ENSEMBLE trial, timing of Ad26.COV2.S dose and serum sampling, and time period for diagnosis of the moderate, severe-critical, and moderate to severe-critical endpoints, for all regions pooled and for each region separately.

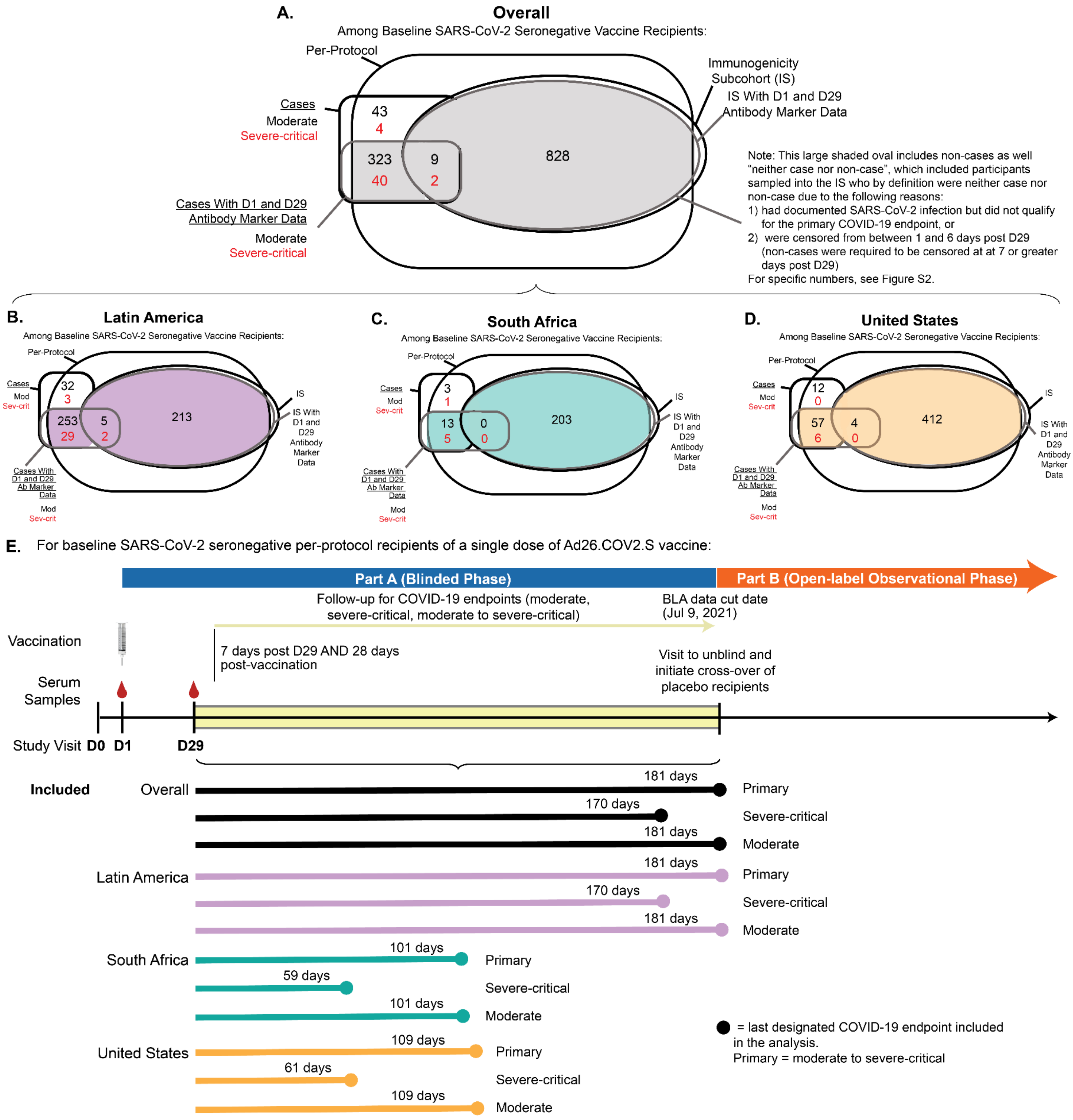

Supplementary Fig. 2. Schematic diagram showing participant flow from enrollment to the case-cohort set of baseline SARS-CoV-2 seronegative per-protocol participants.

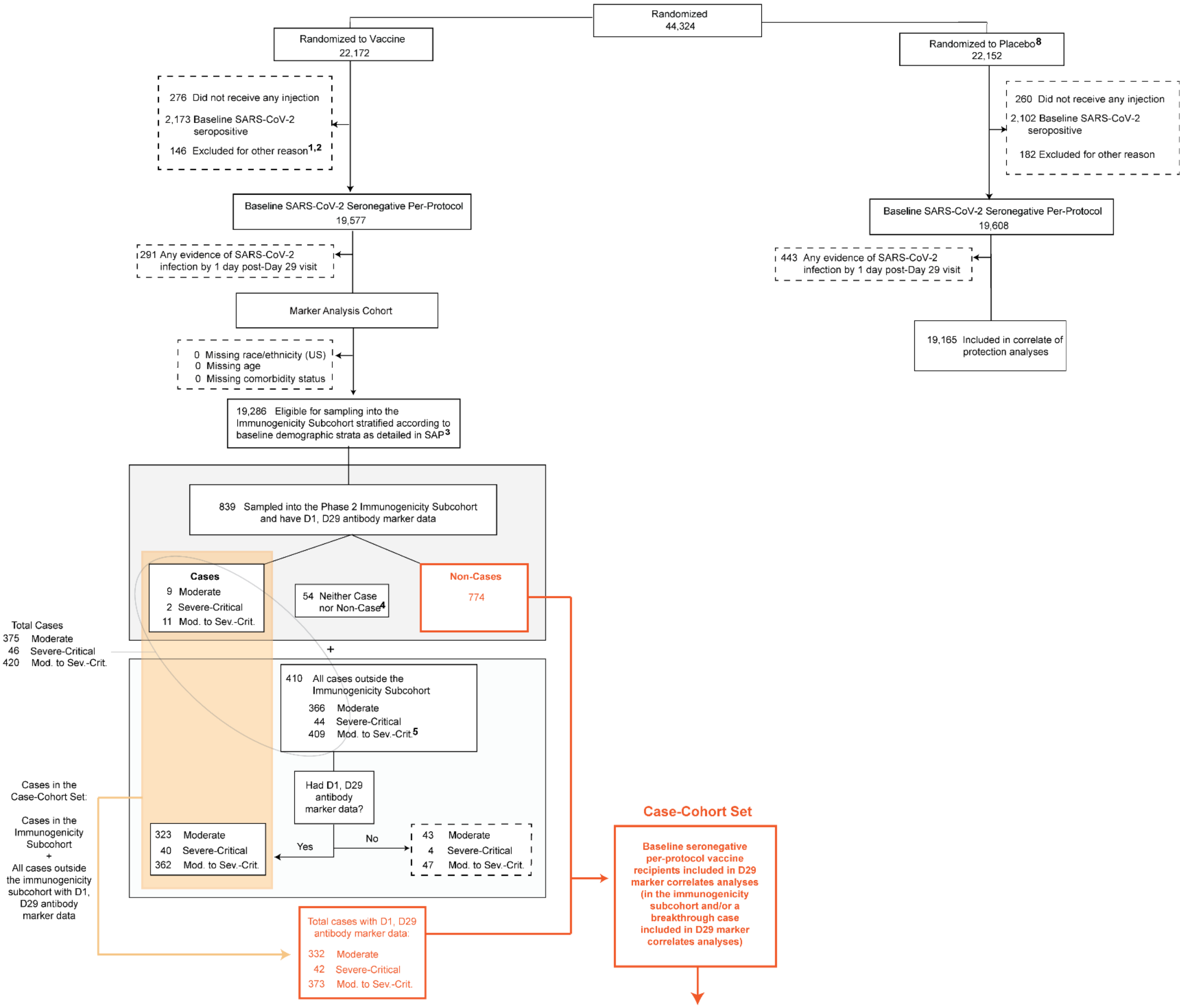

Baseline demographic covariate strata

		1	2	3	4	5	6	7	8	9	10	11	12	13	14	15	16	Total
Cases	Moderate	6	5	0	1	28	9	5	7	147	59	26	26	4	8	0	1	332
	Severe-Critical	2	0	0	0	1	1	0	2	17	6	0	8	3	2	0	0	42
	Mod. to Sev.-Crit.	8	5	0	1	29	10	5	9	163	65	26	34	7	10	0	1	373
	Non-Cases	49	51	41	55	47	54	47	52	50	48	49	50	43	40	46	52	774
		Minority US[6]				Non-Minority US				Latin America				South Africa				
	Age	18-59	18-59	≥60	≥60	18-59	18-59	≥60	≥60	18-59	18-59	≥60	≥60	18-59	18-59	≥60	≥60	
	Co-morbidities[7]	N	Y	N	Y	N	Y	N	Y	N	Y	N	Y	N	Y	N	Y	

1 Participants could have been excluded for more than one reason.
2 Other reasons for exclusion included RT-PCR positive at baseline, violated inclusion or exclusion criteria, received wrong vaccine or incorrect dose, received disallowed concomitant medication, or other.
3 See the SAP for details.
4 Participants who by definition were neither Case nor Non-Case for the correlates analysis, including participants who had a documented SARS-CoV-2 infection but did not qualify for the primary COVID-19 endpoint, as well as participants who were censored from between 1 and 6 days post D29 (non-cases were required to be censored at at 7 or greater days post D29).
5 The number of moderate to severe-critical cases (163) is one less than the number of moderate cases (147) plus the number of severe-critical cases (17) due to one participant having two cases at different time points (first a moderate case, and later a severe-critical case; participant in stratum 9).
6 Minority is defined as the complement of being known to be White Non-Hispanic. White Non-Hispanic is defined as Race=White and Ethnicity=Not Hispanic or Latino. All other Race subgroups are defined as Black, Asian, American Indian or Alaska Native, Native Hawaiian or Other Pacific Islander, Multiracial, Other, Not reported, or Unknown. (In Latin America, the American Indian or Alaska Native category was labeled as "Indigenous South American".) Minority status was only reported in the U.S.
7 Co-morbidities are listed in Table S4 of Sadoff et al. 2021, NEJM and consisted of conditions that have been associated with increased risk of severe COVID-19.
8 Placebo recipient ptids are used in the correlate of protection analyses. However, antibody data from the placebo arm are not used in correlates analyses, given no variability in values; they were only used to verify low false positive rates of the immunoassays.

Supplementary Fig. 3. SARS-CoV-2 variants causing the severe-critical COVID-19 endpoints, shown by days post-D29 visit of severe-critical COVID-19 occurrence, broken out by geographic region and treatment assignment. Endpoint counts do not require having D1 and D29 antibody marker data (see flowchart Figure S2). As in Sadoff et al.,[3] "Reference" refers to the index strain (GenBank accession number: MN908947.3) harboring the D614G point mutation.

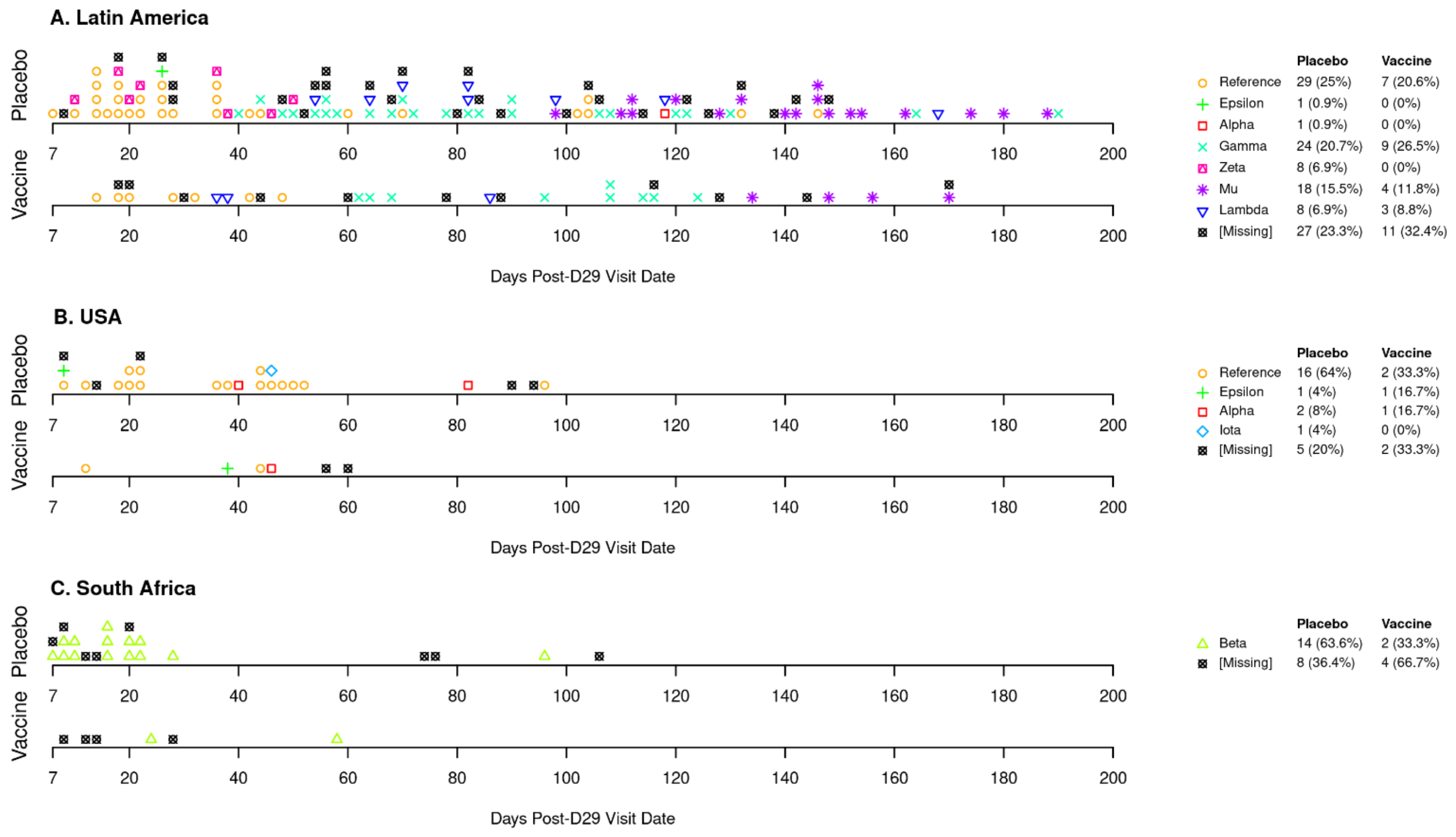

Supplementary Fig. 4. D29 antibody marker level in participants in Latin America by COVID-19 outcome status (moderate COVID-19 case, severe-critical COVID-19 case, or non-case). (A) Anti-Spike IgG concentration, **(B)** anti-receptor binding domain (RBD) IgG concentration, and **(C)** 50% inhibitory dilution neutralizing antibody (nAb-ID50) titer. Data points are from baseline SARS-CoV-2 seronegative per-protocol vaccine recipients in Latin America. The violin plots contain interior box plots with upper and lower horizontal edges the 25th and 75th percentiles of antibody level and middle line the 50th percentile, and vertical bars the distance from the 25th (or 75th) percentile of antibody level and the minimum (or maximum) antibody level within the 25th (or 75th) percentile of antibody level minus (or plus) 1.5 times the interquartile range. Each side shows a rotated probability density (estimated by a kernel density estimator with a default Gaussian kernel) of the data. Positive response frequencies (Freq) were computed with inverse probability of sampling weighting. Positive response for Spike IgG was defined by IgG > 10.8424 BAU/ml and for RBD IgG was defined by IgG > 14.0858 BAU/ml. ULoQ = 238.1165 BAU/ml for Spike IgG and 172.5755 BAU/ml for RBD IgG. Positive response for nAb-ID50 was defined by a D1 nAb-ID50 titer < LLOQ (LLOQ = 4.8975 IU50/ml) with detectable D29 nAb-ID50 (≥ LLOQ), or by D1 nAb-ID50 > LLOQ with at least a 4-fold increase in D29 nAb-ID50. ULoQ = 844.7208 IU50/ml for ID50. Moderate COVID-19 cases are baseline SARS-CoV-2 seronegative per-protocol vaccine recipients with the moderate COVID-19 endpoint (moderate COVID-19 with onset both ≥ 7 days post D29 and ≥ 28 days post-vaccination) up to 181 days post D29 but no later than the data cut (July 9, 2021). Severe-critical COVID-19 cases are baseline SARS-CoV-2 seronegative per-protocol vaccine recipients with the severe-critical COVID-19 endpoint (severe-critical COVID-19 with onset both ≥ 7 days post D29 and ≥ 28 days post-vaccination) up to 170 days post D29 but no later than the data cut (July 9, 2021). Non-cases are baseline seronegative per-protocol vaccine recipients sampled into the immunogenicity subcohort with no evidence of SARS-CoV-2 infection up to the end of the correlates study period, which is up to 181 days post D29 but no later than the data cut (July 9, 2021). BAU, binding antibody units; IU, international units; LLoQ, lower limit of quantitation; (designated as LoQ on panel C); Pos.Cut, positivity cut-off; ULoQ, upper limit of quantitation.

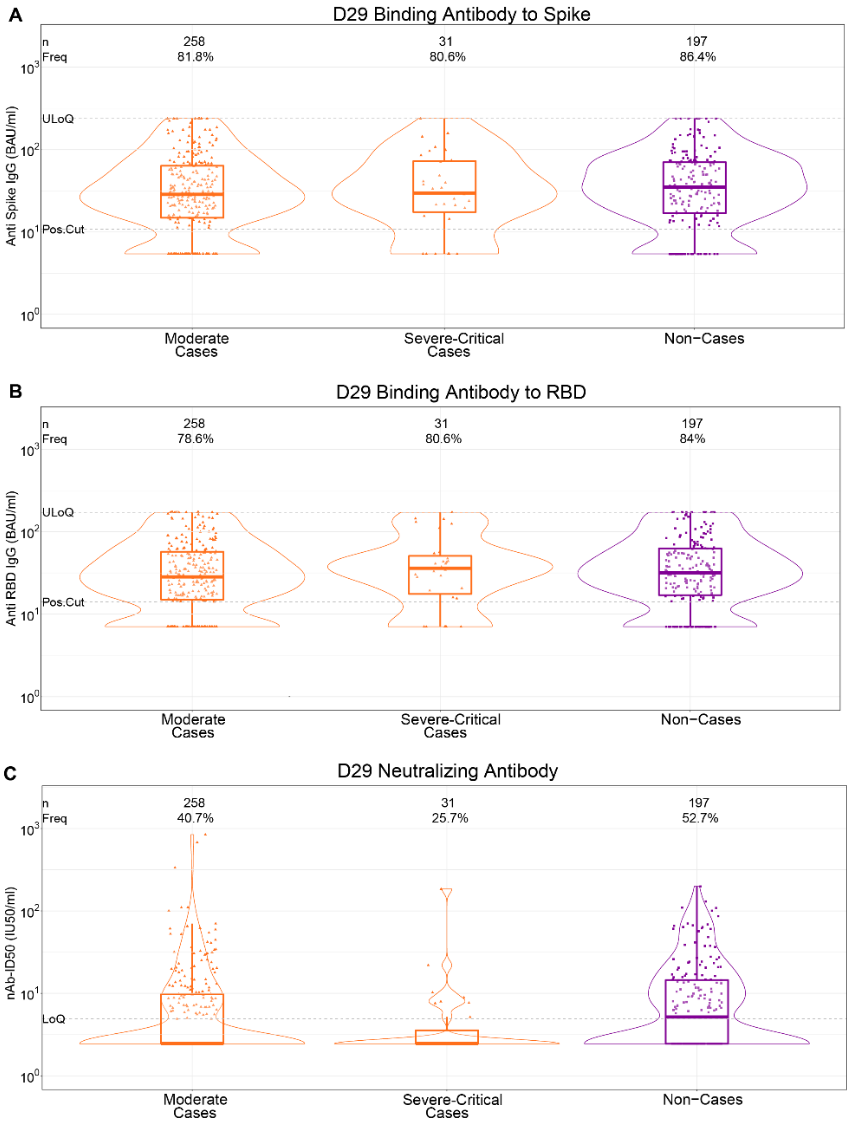

Supplementary Fig. 5. D29 antibody marker level in participants in South Africa by COVID-19 outcome status (moderate COVID-19 case, severe-critical COVID-19 case, or non-case). (A) Anti-Spike IgG concentration, **(B)** anti-receptor binding domain (RBD) IgG concentration, and **(C)** 50% inhibitory dilution neutralizing antibody (nAb-ID50) titer. Data points are from baseline SARS-CoV-2 seronegative per-protocol vaccine recipients in South Africa. The violin plots contain interior box plots with upper and lower horizontal edges the 25th and 75th percentiles of antibody level and middle line the 50th percentile, and vertical bars the distance from the 25th (or 75th) percentile of antibody level and the minimum (or maximum) antibody level within the 25th (or 75th) percentile of antibody level minus (or plus) 1.5 times the interquartile range. Each side shows a rotated probability density (estimated by a kernel density estimator with a default Gaussian kernel) of the data. Positive response frequencies (Freq) were computed with inverse probability of sampling weighting. Positive response for Spike IgG was defined by IgG > 10.8424 BAU/ml and for RBD IgG was defined by IgG > 14.0858 BAU/ml. ULoQ = 238.1165 BAU/ml for Spike IgG and 172.5755 BAU/ml for RBD IgG. Positive response for nAb-ID50 was defined by a D1 nAb-ID50 titer < LLOQ (LLOQ = 4.8975 IU50/ml) with detectable D29 nAb-ID50 (≥ LLOQ), or by D1 nAb-ID50 > LLOQ with at least a 4-fold increase in D29 nAb-ID50. ULoQ = 844.7208 IU50/ml for ID50. Moderate COVID-19 cases are baseline SARS-CoV-2 seronegative per-protocol vaccine recipients with the moderate COVID-19 endpoint (moderate COVID-19 with onset both ≥ 7 days post D29 and ≥ 28 days post-vaccination) up to 101 days post D29 but no later than the data cut (July 9, 2021). Severe-critical COVID-19 cases are baseline SARS-CoV-2 seronegative per-protocol vaccine recipients with the severe-critical COVID-19 endpoint (severe-critical COVID-19 with onset both ≥ 7 days post D29 and ≥ 28 days post-vaccination) up to 59 days post D29 but no later than the data cut (July 9, 2021). Non-cases are baseline seronegative per-protocol vaccine recipients sampled into the immunogenicity subcohort with no evidence of SARS-CoV-2 infection up to the end of the correlates study period, which is up to 101 days post D29 but no later than the data cut (July 9, 2021). BAU, binding antibody units; IU, international units; LLoQ, lower limit of quantitation (designated as LoQ on panel C); Pos.Cut, positivity cut-off; ULoQ, upper limit of quantitation.

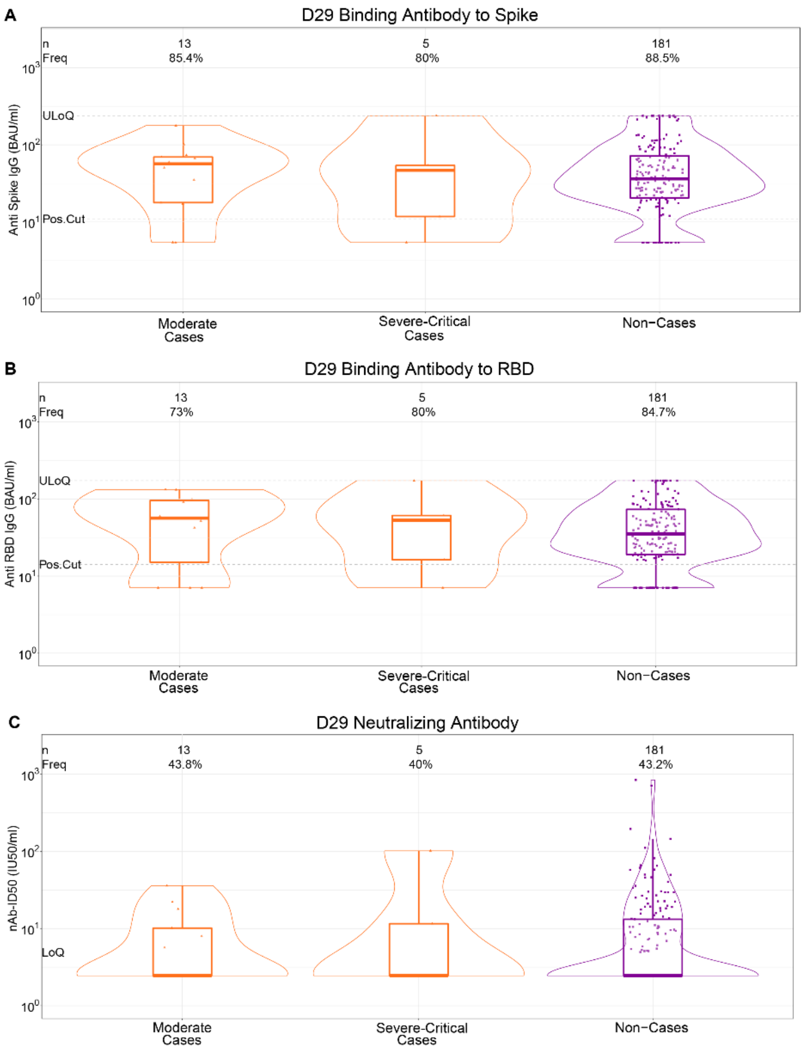

Supplementary Fig. 6. D29 antibody marker level in participants in the United States by COVID-19 outcome status (moderate COVID-19 case, severe-critical COVID-19 case, or non-case). **(A)** Anti-Spike IgG concentration, **(B)** anti-receptor binding domain (RBD) IgG concentration, and **(C)** 50% inhibitory dilution neutralizing antibody (nAb-ID50) titer. Data points are from baseline SARS-CoV-2 seronegative per-protocol vaccine recipients in the United States. The violin plots contain interior box plots with upper and lower horizontal edges the 25th and 75th percentiles of antibody level and middle line the 50th percentile, and vertical bars the distance from the 25th (or 75th) percentile of antibody level and the minimum (or maximum) antibody level within the 25th (or 75th) percentile of antibody level minus (or plus) 1.5 times the interquartile range. Each side shows a rotated probability density (estimated by a kernel density estimator with a default Gaussian kernel) of the data. Positive response frequencies (Freq) were computed with inverse probability of sampling weighting. Positive response for Spike IgG was defined by IgG > 10.8424 BAU/ml and for RBD IgG was defined by IgG > 14.0858 BAU/ml. ULoQ = 238.1165 BAU/ml for Spike IgG and 172.5755 BAU/ml for RBD IgG. Positive response for nAb-ID50 was defined by a D1 nAb-ID50 titer < LLOQ (LLOQ = 4.8975 IU50/ml) with detectable D29 nAb-ID50 (≥ LLOQ), or by D1 nAb-ID50 > LLOQ with at least a 4-fold increase in D29 nAb-ID50. ULoQ = 844.7208 IU50/ml for ID50. Moderate COVID-19 cases are baseline SARS-CoV-2 seronegative per-protocol vaccine recipients with the moderate COVID-19 endpoint (moderate COVID-19 with onset both ≥ 7 days post D29 and ≥ 28 days post-vaccination) up to 109 days post D29 but no later than the data cut (July 9, 2021). Severe-critical COVID-19 cases are baseline SARS-CoV-2 seronegative per-protocol vaccine recipients with the severe-critical COVID-19 endpoint (severe-critical COVID-19 with onset both ≥ 7 days post D29 and ≥ 28 days post-vaccination) up to 61 days post D29 but no later than the data cut (July 9, 2021). Non-cases are baseline seronegative per-protocol vaccine recipients sampled into the immunogenicity subcohort with no evidence of SARS-CoV-2 infection up to the end of the correlates study period, which is up to 109 days post D29 but no later than the data cut (July 9, 2021). BAU, binding antibody units; IU, international units; LLoQ, lower limit of quantitation (designated as LoQ on panel C); Pos.Cut, positivity cut-off; ULoQ, upper limit of quantitation.

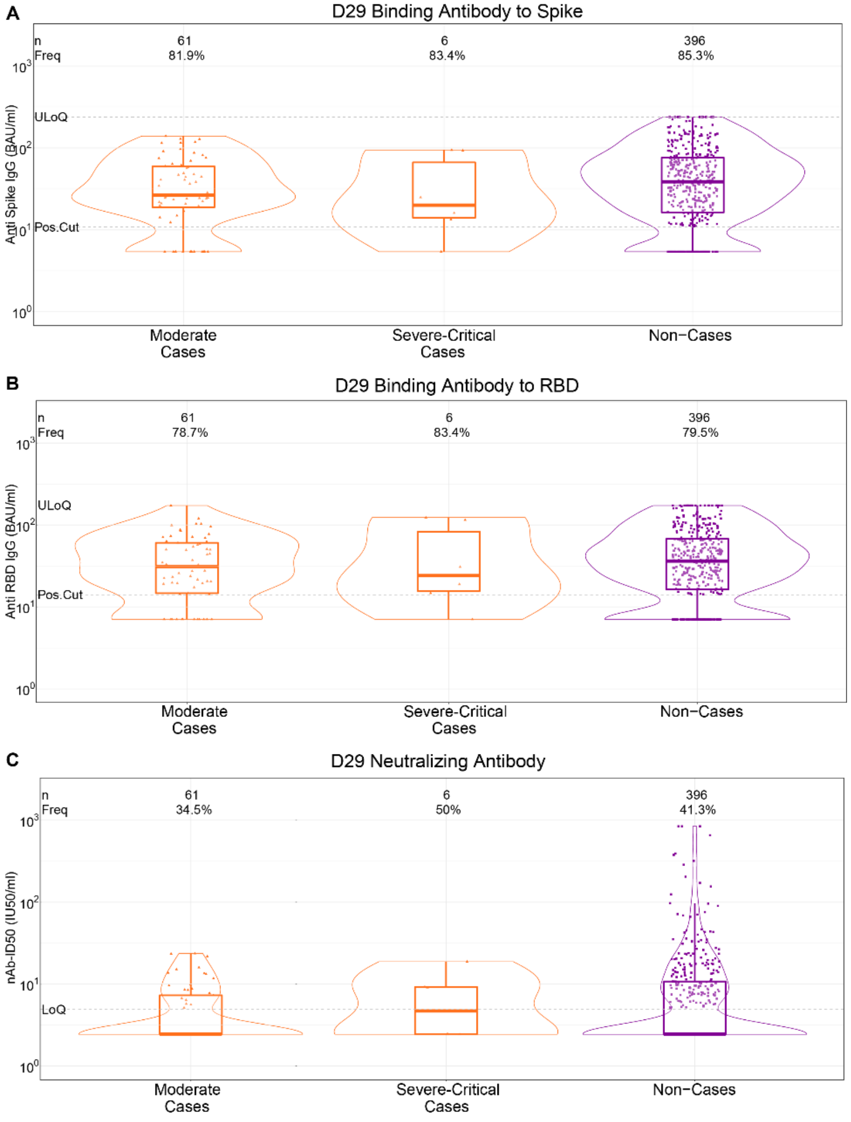

Supplementary Fig. 7. D29 antibody marker level by COVID-19 outcome status (moderate COVID-19 case, severe-critical COVID-19 case, or non-case) in placebo recipients. (A) Anti-Spike IgG concentration, **(B)** anti-receptor binding domain (RBD) IgG concentration, and **(C)** 50% inhibitory dilution neutralizing antibody (nAb-ID50) titer. Data points are from baseline SARS-CoV-2 seronegative per-protocol placebo recipients. The violin plots contain interior box plots with upper and lower horizontal edges the 25th and 75th percentiles of antibody level and middle line the 50th percentile, and vertical bars the distance from the 25th (or 75th) percentile of antibody level and the minimum (or maximum) antibody level within the 25th (or 75th) percentile of antibody level minus (or plus) 1.5 times the interquartile range. Each side shows a rotated probability density (estimated by a kernel density estimator with a default Gaussian kernel) of the data. Positive response frequencies (Freq) were computed with inverse probability of sampling weighting. Positive response for Spike IgG was defined by IgG > 10.8424 BAU/ml and for RBD IgG was defined by IgG > 14.0858 BAU/ml. ULoQ = 238.1165 BAU/ml for Spike IgG and 172.5755 BAU/ml for RBD IgG. Positive response for nAb-ID50 was defined by a D1 nAb-ID50 titer < LLOQ (LLOQ = 4.8975 IU50/ml) with detectable D29 nAb-ID50 (≥ LLOQ), or by D1 nAb-ID50 > LLOQ with at least a 4-fold increase in D29 nAb-ID50. ULoQ = 844.7208 IU50/ml for ID50. Moderate COVID-19 cases are baseline SARS-CoV-2 seronegative per-protocol vaccine recipients with the moderate COVID-19 endpoint (moderate COVID-19 with onset both ≥ 7 days post D29 and ≥ 28 days post-vaccination) up to 181 days post D29 but no later than the data cut (July 9, 2021). Severe-critical COVID-19 cases are baseline SARS-CoV-2 seronegative per-protocol vaccine recipients with the severe-critical COVID-19 endpoint (severe-critical COVID-19 with onset both ≥ 7 days post D29 and ≥ 28 days post-vaccination) up to 170 days post D29 but no later than the data cut (July 9, 2021). Non-cases are baseline seronegative per-protocol vaccine recipients sampled into the immunogenicity subcohort with no evidence of SARS-CoV-2 infection up to the end of the correlates study period, which is up to 181 days post D29 but no later than the data cut (July 9, 2021). BAU, binding antibody units; IU, international units; LLoQ, lower limit of quantitation (designated as LoQ on panel C); Pos.Cut, positivity cut-off; ULoQ, upper limit of quantitation.

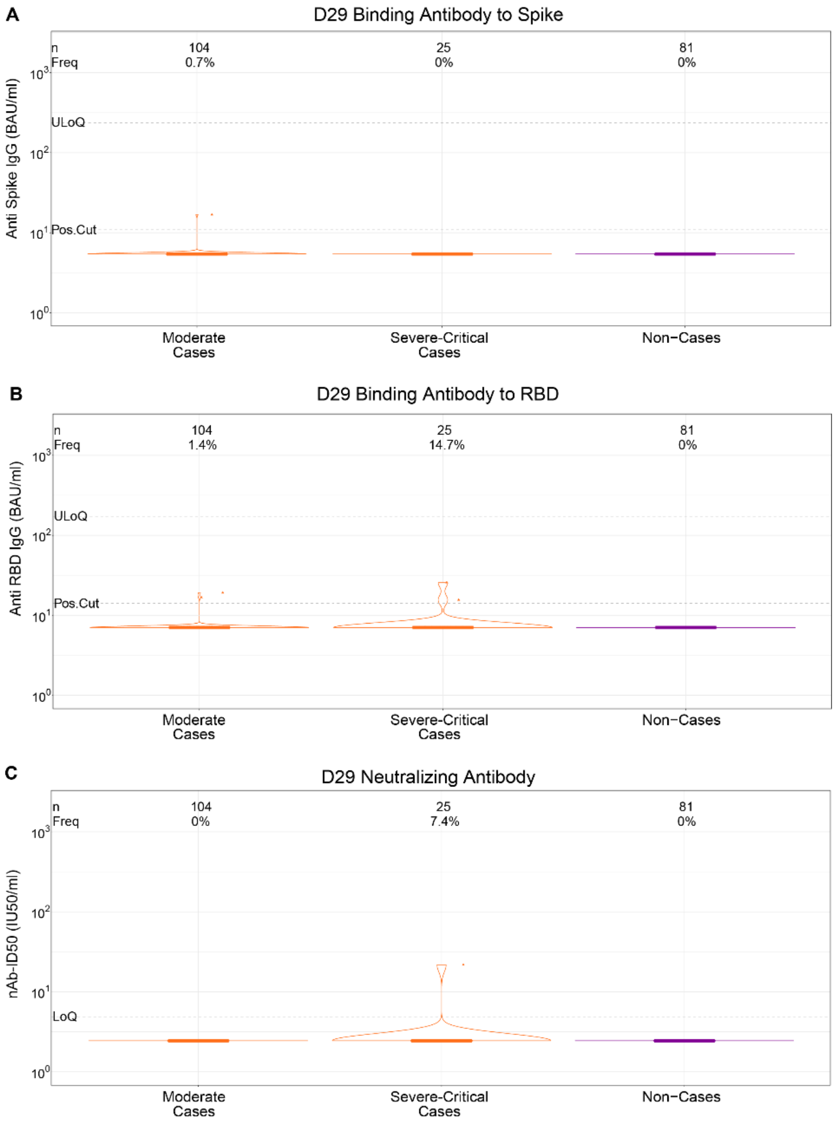

Supplementary Fig. 8. D29 (A) Anti-Spike IgG concentration, (B) anti-RBD IgG concentration, and (C) 50% inhibitory dilution neutralizing antibody titer (nAb-ID50), shown separately by sex (male, female), in baseline SARS-CoV-2 seronegative vaccine recipients in the immunogenicity subcohort. In (A) and (B), the three dotted lines from top to bottom are: upper limit of quantitation (238.1165 BAU/ml for Spike IgG and 172.5755 BAU/ml for RBD IgG), positivity cut-off (10.8424 BAU/ml for Spike IgG and 14.0858 BAU/ml for RBD IgG), and lower limit of quantitation (1.8429 BAU/ml for Spike IgG and 5.0243 for RBD IgG). BAU, binding antibody units; IU, international units. In (C), the two dotted lines from top to bottom are: upper limit of quantitation (844.7208 IU50/ml), lower limit of quantitation (4.8975 IU50/ml).

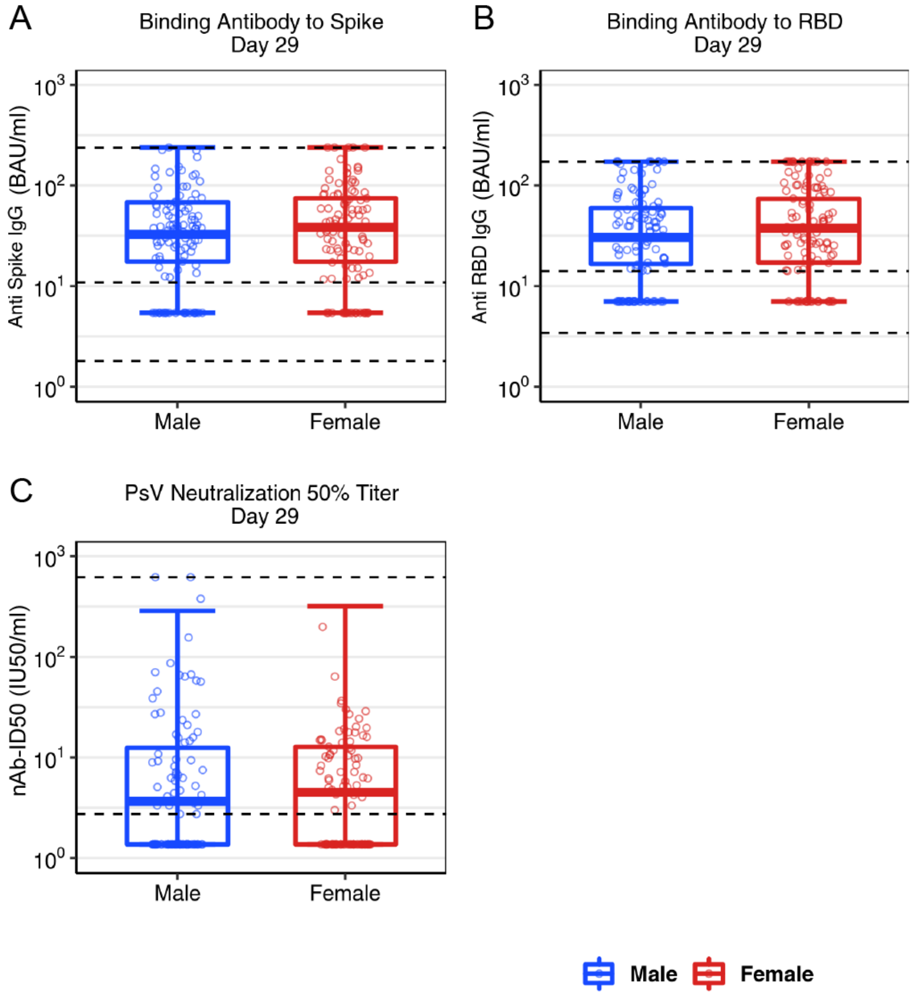

Supplementary Fig. 9. Severe-critical COVID-19 risk by D29 antibody marker tertile. (A-C) Covariate-adjusted cumulative incidence of severe-critical COVID-19 by Low, Medium, and High tertiles of D29 **(A)** anti-Spike IgG concentration, **(B)** anti-RBD IgG concentration, or **(C)** neutralizing antibody titer (nAb-ID50) in baseline SARS-CoV-2–negative per-protocol vaccine recipients. **(D)** Covariate-adjusted hazard ratio of severe-critical COVID-19 across D29 antibody marker tertiles. Endpoint counts for (**A-C**) calculated by inverse probability of sampling D29 marker weighting. The overall p value is from a generalized Wald test of whether the hazard rate of severe-critical COVID-19 differed across the Low, Medium, and High subgroups. Analyses adjusted for baseline behavioral risk score and geographic region. BAU, binding antibody units; CI, confidence interval; FDR, false discovery rate; FWER, family-wise error rate; IU, international units; nAb-ID50, 50% inhibitory dilution neutralizing antibody; Pt. Est., point estimate.

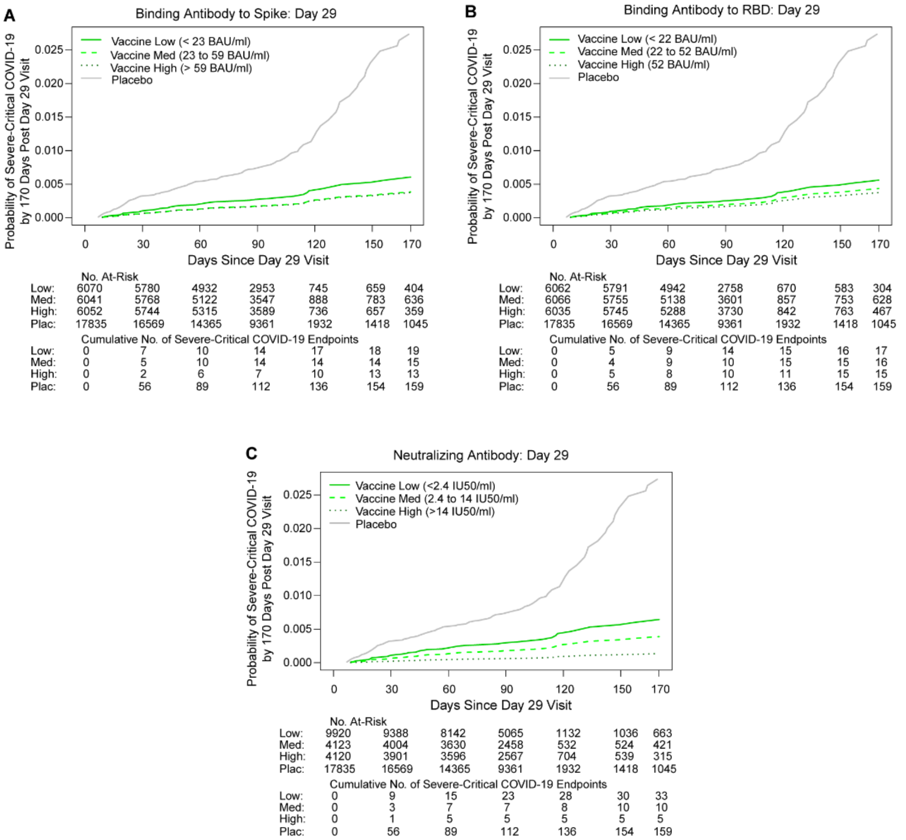

*No. At-Risk = estimated number in the population for analysis: baseline seronegative per-protocol vaccine recipients not experiencing the severe-critical COVID-19 endpoint or with any evidence of infection through D29.
**Cumulative No. of Severe-Critical COVID-19 Endpoints = estimated cumulative number of this cohort with a severe-critical COVID-19 endpoint.

D

D29 Immunologic Marker	Tertile	No. cases / No. at-risk§	Attack rate	Haz. Ratio Pt. Est.	Haz. Ratio 95% CI	P-value (2-sided)	Overall P-value	FDR-adjusted P-value†	FWER-adjusted P-value†
Anti Spike IgG (BAU/ml)	Low	19/6,070	0.0031	1	N/A	N/A	0.411	0.633	0.723
	Medium	15/6,041	0.0025	0.63	(0.28, 1.43)	0.271			
	High	13/6,052	0.0021	0.62	(0.27, 1.42)	0.254			
Anti RBD IgG (BAU/ml)	Low	17/6,062	0.0028	1	N/A	N/A	0.632	0.633	0.793
	Medium	16/6,066	0.0026	0.78	(0.35, 1.73)	0.534			
	High	15/6,035	0.0025	0.67	(0.28, 1.57)	0.352			
nAb-ID50 (IU50/ml)	Low	33/9,920	0.0033	1	N/A	N/A	0.025	0.106	0.087
	Medium	10/4,123	0.0024	0.60	(0.26, 1.42)	0.247			
	High	5/4,120	0.0012	0.21	(0.07, 0.67)	0.008			
Placebo		163/17,835	0.0091						

Baseline covariates adjusted for: baseline risk score, geographic region. RBD, receptor binding domain; ID50, 50% inhibitory dilution titer.
§ No. at-risk = estimated number in the population for analysis, i.e. baseline SARS-CoV-2 seronegative per-protocol vaccine recipients not experiencing the severe-critical COVID-19 endpoint or with evidence of SARS-CoV-2 infection through D29; no. cases = numbers of this cohort with an observed severe-critical COVID-19 endpoint (with onset ≥ 7 days post D29 and ≥ 28 days post vaccination).
† q-value and FWER (family-wise error rate) are computed over the set of p-values both for quantitative markers and categorical markers using the Westfall and Young permutation method (10000 replicates).

Supplementary Fig. 10. Moderate COVID-19 risk by D29 antibody marker tertile. (A-C) Covariate-adjusted cumulative incidence of moderate COVID-19 by Low, Medium, and High tertiles of D29 **(A)** anti-Spike IgG concentration, **(B)** anti-RBD IgG concentration, or **(C)** neutralizing antibody titer (nAb-ID50) in baseline SARS-CoV-2–negative per-protocol vaccine recipients. **(D)** Covariate-adjusted hazard ratio of moderate COVID-19 across D29 antibody marker tertiles. Endpoint counts for (**A-C**) calculated by inverse probability of sampling D29 marker weighting. The overall p value is from a generalized Wald test of whether the hazard rate of moderate COVID-19 differed across the Low, Medium, and High subgroups. Analyses adjusted for baseline behavioral risk score and geographic region. BAU, binding antibody units; CI, confidence interval; FDR, false discovery rate; FWER, family-wise error rate; IU, international units; nAb-ID50, 50% inhibitory dilution neutralizing antibody; Pt. Est., point estimate.

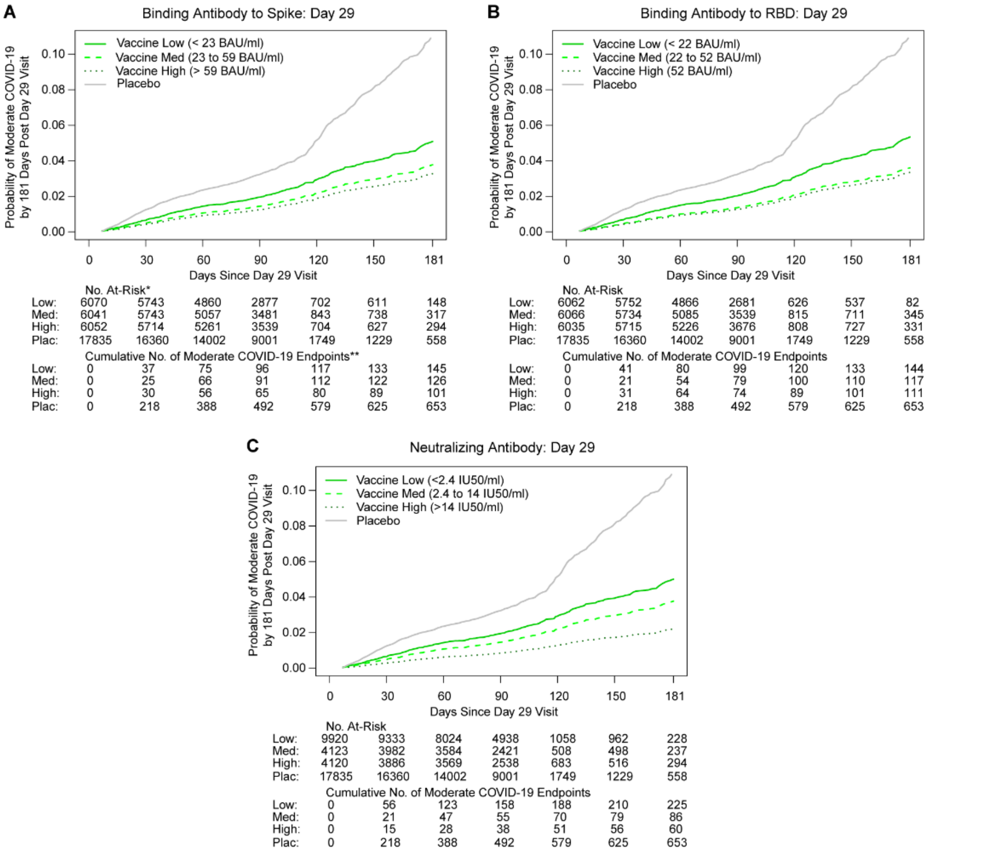

*No. At-Risk = estimated number in the population for analysis: baseline seronegative per-protocol vaccine recipients not experiencing the moderate COVID-19 endpoint or with any evidence of infection through D29.
**Cumulative No. of Moderate COVID-19 Endpoints = estimated cumulative number of this cohort with a moderate COVID-19 endpoint.

D

D29 Immunologic Marker	Tertile	No. cases / No. at-risk§	Attack rate	Haz. Ratio Pt. Est.	Haz. Ratio 95% CI	P-value (2-sided)	Overall P-value	FDR-adjusted P-value†	FWER-adjusted P-value†
Anti Spike IgG (BAU/ml)	Low	145/6,070	0.0239	1	N/A	N/A	0.142	0.169	0.220
	Medium	127/6,041	0.0210	0.73	(0.46, 1.16)	0.188			
	High	102/6,052	0.0169	0.64	(0.40, 1.01)	0.057			
Anti RBD IgG (BAU/ml)	Low	144/6,062	0.0238	1	N/A	N/A	0.085	0.122	0.173
	Medium	118/6,066	0.0195	0.67	(0.42, 1.05)	0.079			
	High	112/6,035	0.0186	0.62	(0.39, 0.99)	0.047			
nAb-ID50 (IU50/ml)	Low	225/9,920	0.0227	1	N/A	N/A	0.012	0.054	0.052
	Medium	88/4,123	0.0213	0.75	(0.46, 1.21)	0.231			
	High	61/4,120	0.0148	0.43	(0.25, 0.75)	0.003			
Placebo		657/17,835	0.0368						

Baseline covariates adjusted for: baseline risk score, geographic region. RBD, receptor binding domain; ID50, 50% inhibitory dilution titer.
§ No. at-risk = estimated number in the population for analysis, i.e. baseline SARS-CoV-2 seronegative per-protocol vaccine recipients not experiencing the moderate COVID-19 endpoint or with evidence of SARS-CoV-2 infection through D29; no. cases = numbers of this cohort with an observed moderate COVID-19 endpoint (with onset ≥ 7 days post D29 and ≥ 28 days post vaccination).
† q-value and FWER (family-wise error rate) are computed over the set of p-values both for quantitative markers and categorical markers using the Westfall and Young permutation method (10000 replicates).

Supplementary Fig. 11. Moderate to severe-critical COVID-19 risk by D29 antibody marker tertile. **(A-C)** Covariate-adjusted cumulative incidence of moderate to severe-critical COVID-19 by Low, Medium, and High tertiles of D29 **(A)** anti-Spike IgG concentration, **(B)** anti-RBD IgG concentration, or **(C)** neutralizing antibody titer (nAb-ID50) in baseline SARS-CoV-2–seronegative per-protocol vaccine recipients. **(D)** Covariate-adjusted hazard ratio of moderate to severe-critical COVID-19 across D29 antibody marker tertiles. Endpoint counts for (**A-C**) calculated by inverse probability of sampling D29 marker weighting. The overall p value is from a generalized Wald test of whether the hazard rate of moderate to severe-critical COVID-19 differed across the Low, Medium, and High subgroups. Analyses adjusted for baseline behavioral risk score and geographic region. BAU, binding antibody units; CI, confidence interval; FDR, false discovery rate; FWER, family-wise error rate; IU, international units; nAb-ID50, 50% inhibitory dilution neutralizing antibody; Pt. Est., point estimate.

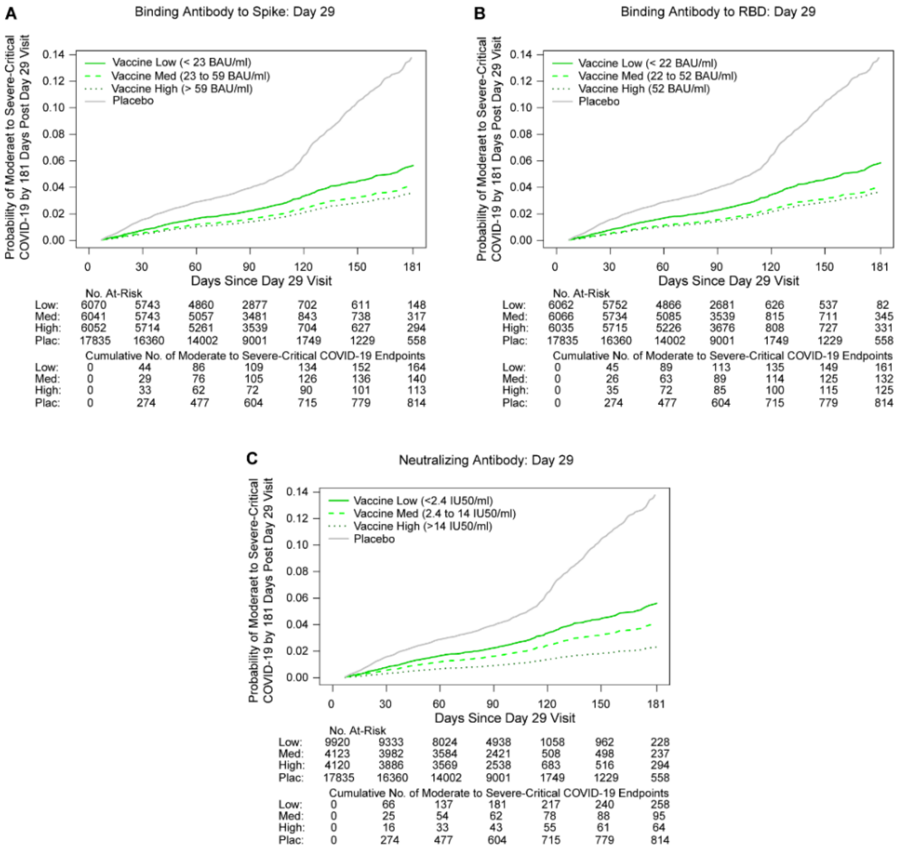

*No. At-Risk = estimated number in the population for analysis: baseline seronegative per-protocol vaccine recipients not experiencing the moderate to severe-critical COVID-19 endpoint or with any evidence of infection through D29.
**Cumulative No. of Moderate to Severe-Critical COVID-19 Endpoints = estimated cumulative number of this cohort with a moderate to severe-critical COVID-19 endpoint.

D

D29 Immunologic Marker	Tertile	No. cases / No. at-risk§	Attack rate	Haz. Ratio Pt. Est.	Haz. Ratio 95% CI	P-value (2-sided)	Overall P-value	FDR-adjusted P-value†	FWER-adjusted P-value†
Anti Spike IgG (BAU/ml)	Low	164/6,070	0.0270	1	N/A	N/A	0.112	0.156	0.204
	Medium	142/6,041	0.0235	0.72	(0.46, 1.13)	0.156			
	High	115/6,052	0.0190	0.63	(0.40, 0.99)	0.045			
Anti RBD IgG (BAU/ml)	Low	161/6,062	0.0266	1	N/A	N/A	0.081	0.138	0.185
	Medium	134/6,066	0.0221	0.68	(0.44, 1.05)	0.085			
	High	126/6,035	0.0209	0.62	(0.39, 0.98)	0.042			
nAb-ID50 (IU50/ml)	Low	258/9,920	0.0260	1	N/A	N/A	0.005	0.026	0.026
	Medium	97/4,123	0.0235	0.72	(0.45, 1.15)	0.172			
	High	65/4,120	0.0158	0.40	(0.23, 0.69)	0.001			
Placebo		820/17,835	0.0460						

Baseline covariates adjusted for: baseline risk score, geographic region. RBD, receptor binding domain; ID50, 50% inhibitory dilution titer.
§ No. at-risk = estimated number in the population for analysis, i.e. baseline SARS-CoV-2 seronegative per-protocol vaccine recipients not experiencing the moderate to severe-critical COVID-19 endpoint or with evidence of SARS-CoV-2 infection through D29; no. cases = numbers of this cohort with an observed moderate to severe-critical COVID-19 endpoint (with onset ≥ 7 days post D29 and ≥ 28 days post vaccination).
† q-value and FWER (family-wise error rate) are computed over the set of p-values both for quantitative markers and categorical markers using the Westfall and Young permutation method (10000 replicates).

Supplementary Fig. 12. Severe-critical COVID-19 risk by D29 antibody marker tertile in participants in Latin America. (A-C) Covariate-adjusted cumulative incidence of severe-critical COVID-19 by Low, Medium, and High tertiles of D29 **(A)** anti-Spike IgG concentration, **(B)** anti-RBD IgG concentration, or **(C)** neutralizing antibody titer (nAb-ID50) in baseline SARS-CoV-2–seronegative per-protocol vaccine recipients in Latin America. **(D)** Covariate-adjusted hazard ratio of severe-critical COVID-19 across D29 antibody marker tertiles. Endpoint counts for (**A-C**) calculated by inverse probability of sampling D29 marker weighting. The overall p value is from a generalized Wald test of whether the hazard rate of severe-critical COVID-19 differed across the Low, Medium, and High subgroups. Analyses adjusted for baseline behavioral risk score. BAU, binding antibody units; CI, confidence interval; FDR, false discovery rate; FWER, family-wise error rate; IU, international units; nAb-ID50, 50% inhibitory dilution neutralizing antibody; Pt. Est., point estimate.

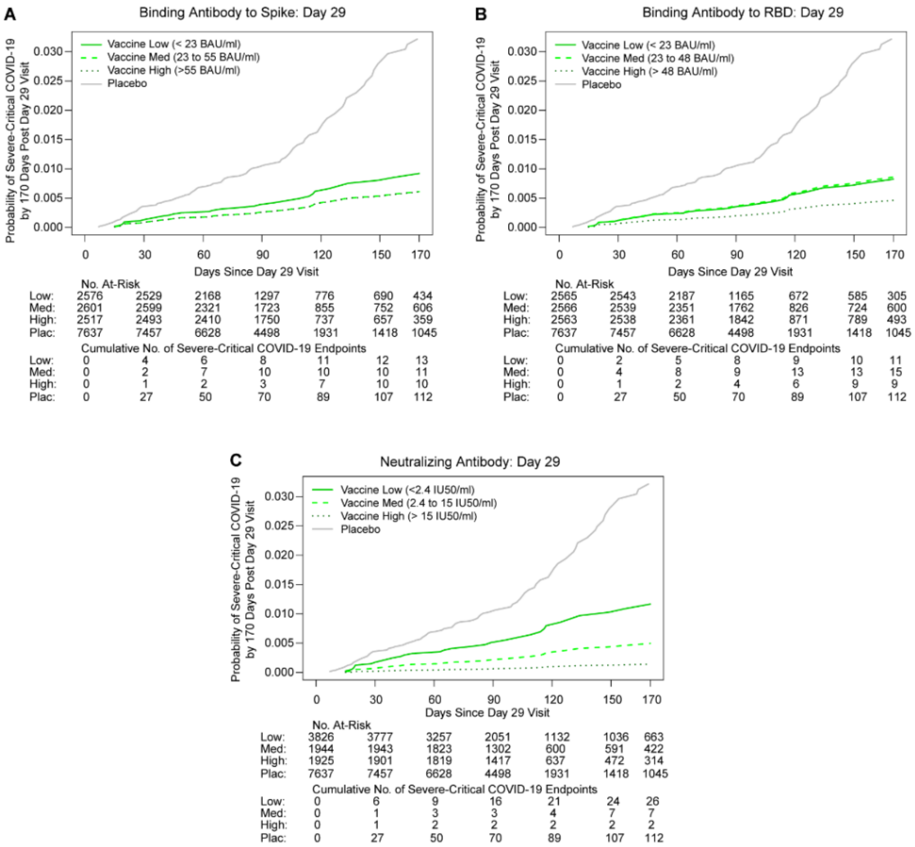

*No. At-Risk = estimated number in the population for analysis: baseline seronegative per-protocol vaccine recipients in Latin America not experiencing the severe-critical COVID-19 endpoint or with any evidence of infection through D29.
**Cumulative No. of Moderate to Severe-Critical COVID-19 Endpoints = estimated cumulative number of this cohort with a severe-critical COVID-19 endpoint.

D

D29 Immunologic Marker	Tertile	No. cases / No. at-risk§	Attack rate	Haz. Ratio Pt. Est.	95% CI	P-value (2-sided)	Overall P-value	FDR-adjusted P-value†	FWER-adjusted P-value†
Anti Spike IgG (BAU/ml)	Low	13/2,576	0.0050	1	N/A	N/A	0.629	0.783	0.874
	Medium	11/2,601	0.0042	0.66	(0.25, 1.77)	0.412			
	High	10/2,517	0.0040	0.66	(0.24, 1.82)	0.420			
Anti RBD IgG (BAU/ml)	Low	11/2,565	0.0043	1	N/A	N/A	0.474	0.774	0.806
	Medium	15/2,566	0.0058	1.04	(0.40, 2.72)	0.930			
	High	9/2,563	0.0035	0.56	(0.19, 1.67)	0.298			
nAb-ID50 (IU50/ml)	Low	26/3,826	0.0068	1	N/A	N/A	0.014	0.047	0.048
	Medium	7/1,944	0.0036	0.42	(0.15, 1.20)	0.104			
	High	2/1,925	0.0010	0.12	(0.02, 0.57)	0.008			
Placebo		116/7,637	0.0152						

Baseline covariates adjusted for: baseline risk score. RBD, receptor binding domain; ID50, 50% inhibitory dilution titer.
§ No. at-risk = estimated number in the population for analysis, i.e. baseline SARS-CoV-2 seronegative per-protocol vaccine recipients in Latin America not experiencing the severe-critical COVID-19 endpoint or with evidence of SARS-CoV-2 infection through D29; no. cases = numbers of this cohort with an observed severe-critical COVID-19 endpoint (with onset $\geq$ 7 days post D29 and $\geq$ 28 days post vaccination).
† q-value and FWER (family-wise error rate) are computed over the set of p-values both for quantitative markers and categorical markers using the Westfall and Young permutation method (10000 replicates).

Supplementary Fig. 13. Moderate COVID-19 risk by D29 antibody marker tertile in participants in Latin America. (A-C) Covariate-adjusted cumulative incidence of moderate COVID-19 by Low, Medium, and High tertiles of D29 **(A)** anti-Spike IgG concentration, **(B)** anti-RBD IgG concentration, or **(C)** neutralizing antibody titer (nAb-ID50) in baseline SARS-CoV-2–seronegative per-protocol vaccine recipients in Latin America. **(D)** Covariate-adjusted hazard ratio of moderate COVID-19 across D29 antibody marker tertiles. Endpoint counts for (**A-C**) calculated by inverse probability of sampling D29 marker weighting. The overall p value is from a generalized Wald test of whether the hazard rate of moderate COVID-19 differed across the Low, Medium, and High subgroups. Analyses adjusted for baseline behavioral risk score. BAU, binding antibody units; CI, confidence interval; FDR, false discovery rate; FWER, family-wise error rate; IU, international units; nAb-ID50, 50% inhibitory dilution neutralizing antibody; Pt. Est., point estimate.

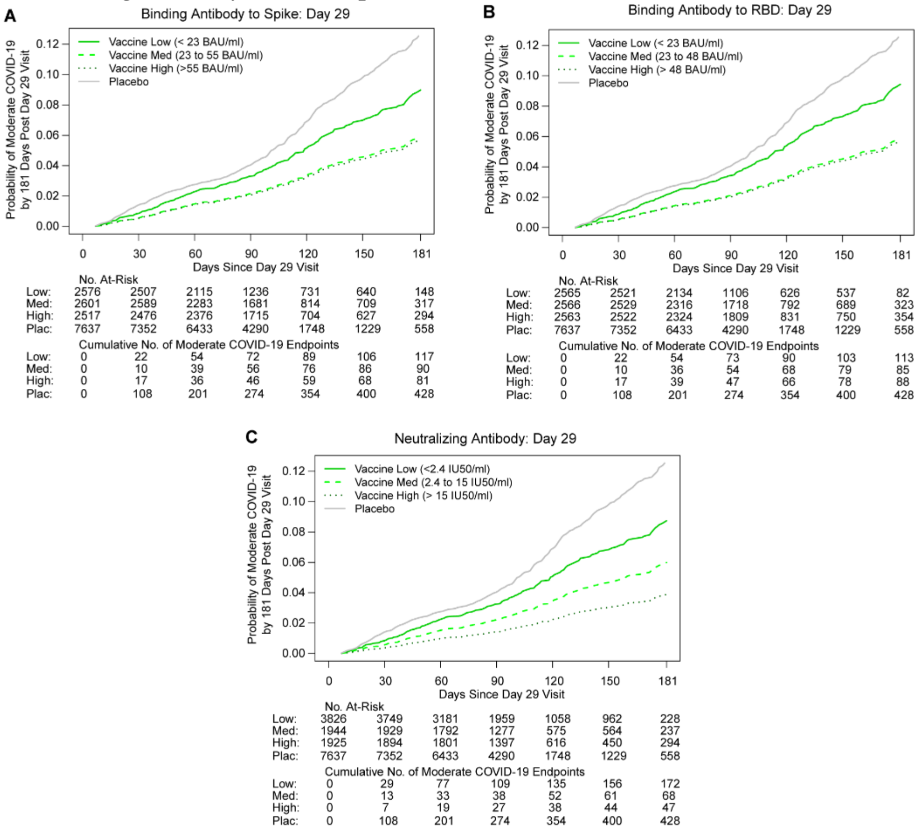

*No. At-Risk = estimated number in the population for analysis: baseline seronegative per-protocol vaccine recipients in Latin America not experiencing the moderate COVID-19 endpoint or with any evidence of infection through D29.
**Cumulative No. of Moderate COVID-19 Endpoints = estimated cumulative number of this cohort with a moderate COVID-19 endpoint.

D

D29 Immunologic Marker	Tertile	No. cases / No. at-risk§	Attack rate	Haz. Ratio Pt. Est.	95% CI	P-value (2-sided)	Overall P-value	FDR-adjusted P-value†	FWER-adjusted P-value†
Anti Spike IgG (BAU/ml)	Low	117/2,576	0.0454	1	N/A	N/A	0.176	0.248	0.315
	Medium	91/2,601	0.0350	0.65	(0.37, 1.13)	0.126			
	High	82/2,517	0.0326	0.62	(0.35, 1.10)	0.102			
Anti RBD IgG (BAU/ml)	Low	113/2,565	0.0441	1	N/A	N/A	0.111	0.197	0.263
	Medium	86/2,566	0.0335	0.61	(0.35, 1.06)	0.080			
	High	90/2,563	0.0351	0.59	(0.33, 1.04)	0.069			
nAb-ID50 (IU50/ml)	Low	172/3,826	0.0450	1	N/A	N/A	0.034	0.138	0.133
	Medium	69/1,944	0.0355	0.67	(0.38, 1.20)	0.178			
	High	48/1,925	0.0249	0.43	(0.23, 0.82)	0.011			
Placebo		432/7,637	0.0566						

Baseline covariates adjusted for: baseline risk score. RBD, receptor binding domain; ID50, 50% inhibitory dilution titer.
§ No. at-risk = estimated number in the population for analysis, i.e. baseline SARS-CoV-2 seronegative per-protocol vaccine recipients in Latin America not experiencing the moderate COVID-19 endpoint or with evidence of SARS-CoV-2 infection through D29; no. cases = numbers of this cohort with an observed moderate COVID-19 endpoint (with onset ≥ 7 days post D29 and ≥ 28 days post vaccination).
† q-value and FWER (family-wise error rate) are computed over the set of p-values both for quantitative markers and categorical markers using the Westfall and Young permutation method (10000 replicates).

Supplementary Fig. 14. Moderate to severe-critical COVID-19 risk by D29 antibody marker tertile in participants in Latin America. (A-C) Covariate-adjusted cumulative incidence of moderate to severe-critical COVID-19 by Low, Medium, and High tertiles of D29 **(A)** anti-Spike IgG concentration, **(B)** anti-RBD IgG concentration, or **(C)** neutralizing antibody titer (nAb-ID50) in baseline SARS-CoV-2–seronegative per-protocol vaccine recipients in Latin America. **(D)** Covariate-adjusted hazard ratio of moderate to severe-critical COVID-19 across D29 antibody marker tertiles. Endpoint counts for (**A-C**) calculated by inverse probability of sampling D29 marker weighting. The overall p value is from a generalized Wald test of whether the hazard rate of moderate to severe-critical COVID-19 differed across the Low, Medium, and High subgroups. Analyses adjusted for baseline behavioral risk score. BAU, binding antibody units; CI, confidence interval; FDR, false discovery rate; FWER, family-wise error rate; IU, international units; nAb-ID50, 50% inhibitory dilution neutralizing antibody; Pt. Est., point estimate.

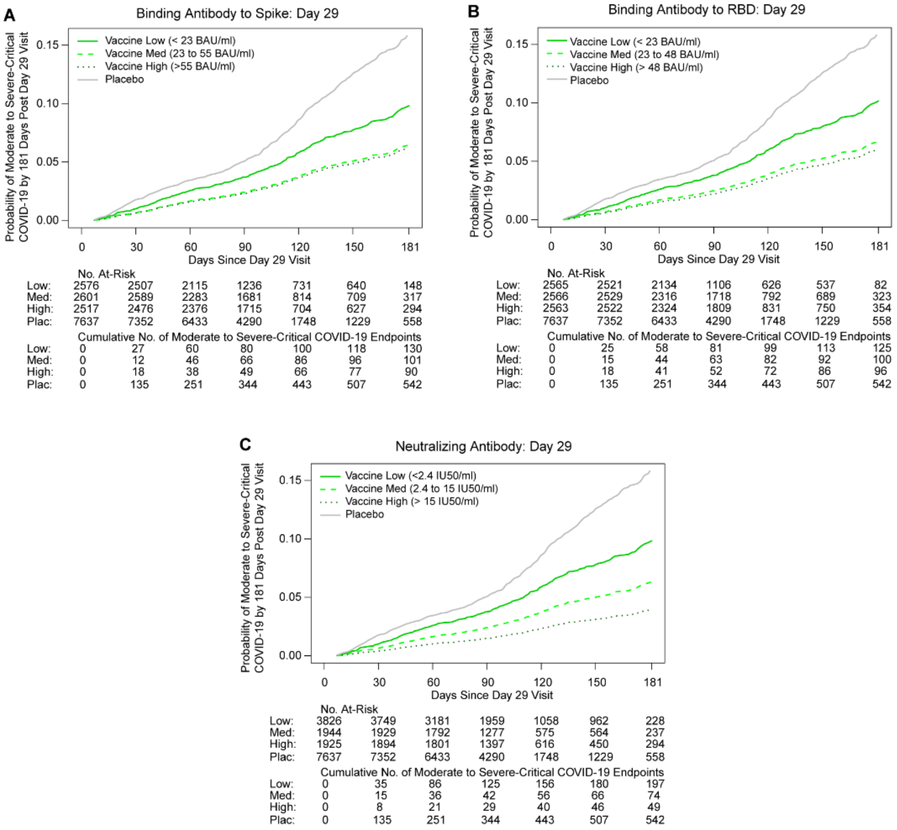

*No. At-Risk = estimated number in the population for analysis: baseline seronegative per-protocol vaccine recipients in Latin America not experiencing the moderate to severe-critical COVID-19 endpoint or with any evidence of infection through D29.
**Cumulative No. of Moderate to Severe-Critical COVID-19 Endpoints = estimated cumulative number of this cohort with a moderate to severe-critical COVID-19 endpoint.

D

D29 Immunologic Marker	Tertile	No. cases / No. at-risk§	Attack rate	Haz. Ratio Pt. Est.	Haz. Ratio 95% CI	P-value (2-sided)	Overall P-value	FDR-adjusted P-value†	FWER-adjusted P-value†
Anti Spike IgG (BAU/ml)		130/2,576	0.0505	1	N/A	N/A	0.165	0.234	0.294
		102/2,601	0.0392	0.65	(0.37, 1.13)	0.124			
		91/2,517	0.0362	0.62	(0.35, 1.08)	0.093			
Anti RBD IgG (BAU/ml)		125/2,565	0.0487	1	N/A	N/A	0.122	0.207	0.270
		101/2,566	0.0394	0.65	(0.37, 1.12)	0.119			
		97/2,563	0.0378	0.58	(0.33, 1.02)	0.058			
nAb-ID50 (IU50/ml)		197/3,826	0.0515	1	N/A	N/A	0.013	0.062	0.062
		75/1,944	0.0386	0.63	(0.36, 1.12)	0.113			
		50/1,925	0.0260	0.39	(0.20, 0.74)	0.004			
Placebo		548/7,637	0.0718						

Baseline covariates adjusted for: baseline risk score. RBD, receptor binding domain; ID50, 50% inhibitory dilution titer.
§ No. at-risk = estimated number in the population for analysis, i.e. baseline SARS-CoV-2 seronegative per-protocol vaccine recipients in Latin America not experiencing the moderate to severe-critical COVID-19 endpoint or with evidence of SARS-CoV-2 infection through D29; no. cases = numbers of this cohort with an observed moderate to severe-critical COVID-19 endpoint (with onset ≥ 7 days post D29 and ≥ 28 days post vaccination).
† q-value and FWER (family-wise error rate) are computed over the set of p-values both for quantitative markers and categorical markers using the Westfall and Young permutation method (10000 replicates).

Supplementary Fig. 15. Moderate to severe-critical COVID-19 risk by D29 antibody marker tertile in participants in South Africa. (A-C) Covariate-adjusted cumulative incidence of moderate to severe-critical COVID-19 by Low, Medium, and High tertiles of D29 **(A)** anti-Spike IgG concentration, **(B)** anti-RBD IgG concentration, or **(C)** neutralizing antibody titer (nAb-ID50) in baseline SARS-CoV-2–seronegative per-protocol vaccine recipients in South Africa. **(D)** Covariate-adjusted hazard ratio of moderate to severe-critical COVID-19 across D29 antibody marker tertiles. Endpoint counts for (**A-C**) calculated by inverse probability of sampling D29 marker weighting. The overall p value is from a generalized Wald test of whether the hazard rate of moderate to severe-critical COVID-19 differed across the Low, Medium, and High subgroups. Analyses adjusted for baseline behavioral risk score. BAU, binding antibody units; CI, confidence interval; FDR, false discovery rate; FWER, family-wise error rate; IU, international units; nAb-ID50, 50% inhibitory dilution neutralizing antibody; Pt. Est., point estimate.

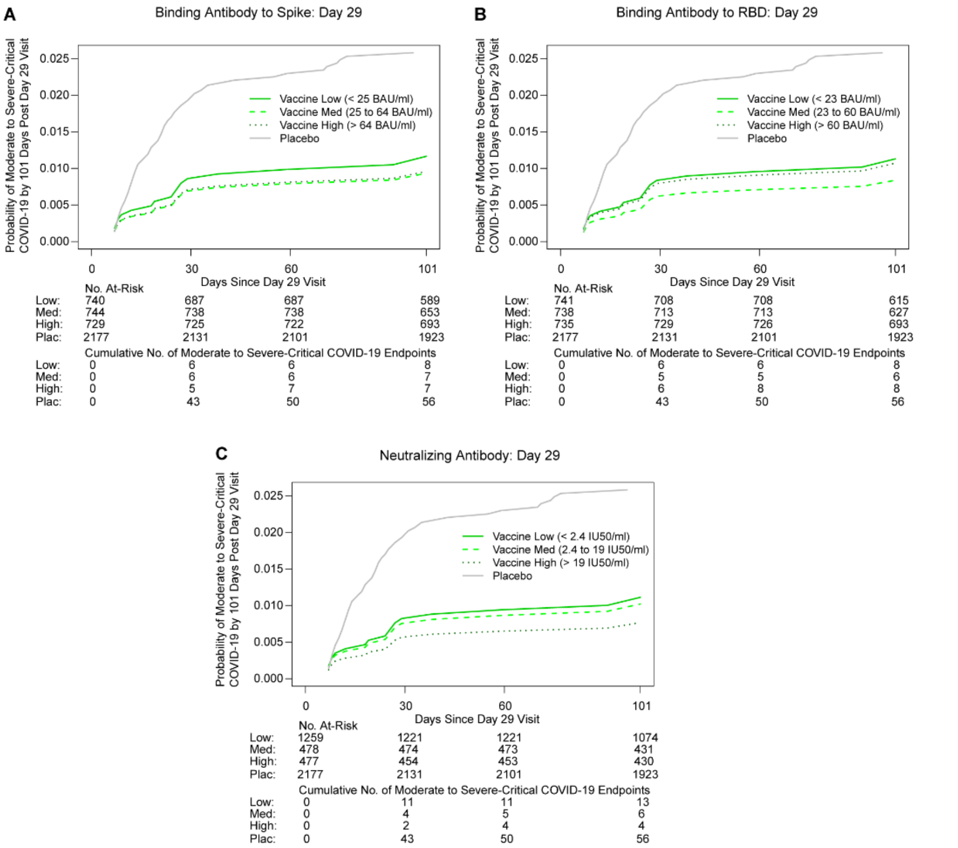

*No. At-Risk = estimated number in the population for analysis: baseline seronegative per-protocol vaccine recipients in South Africa not experiencing the moderate to severe-critical COVID-19 endpoint or with any evidence of infection through D29.
**Cumulative No. of Moderate to Severe-Critical COVID-19 Endpoints = estimated cumulative number of this cohort with a moderate to severe-critical COVID-19 endpoint.

D

D29 Immunologic Marker	Tertile	No. cases / No. at-risk§	Attack rate	Haz. Ratio Pt. Est.	Haz. Ratio 95% CI	P-value (2-sided)	Overall P-value	FDR-adjusted P-value†	FWER-adjusted P-value†
Anti Spike IgG (BAU/ml)	Low	6/ 740	0.0081	1	N/A	N/A	0.909	0.969	0.997
	Medium	5/ 744	0.0067	0.74	(0.17,3.27)	0.695			
	High	6/ 729	0.0082	0.96	(0.23,4.09)	0.957			
Anti RBD IgG (BAU/ml)	Low	6/ 741	0.0081	1	N/A	N/A	0.974	0.976	0.997
	Medium	5/ 738	0.0068	0.84	(0.19,3.80)	0.820			
	High	6/ 735	0.0082	0.93	(0.22,3.88)	0.918			
nAb-ID50 (IU50/ml)	Low	9/1,259	0.0071	1	N/A	N/A	0.854	0.969	0.997
	Medium	5/ 478	0.0105	0.96	(0.24,3.81)	0.955			
	High	2/ 477	0.0042	0.62	(0.11,3.41)	0.586			
Placebo		36/2,177	0.0165						

Baseline covariates adjusted for: baseline risk score. RBD, receptor binding domain; ID50, 50% inhibitory dilution titer.

§ No. at-risk = estimated number in the population for analysis, i.e. baseline SARS-CoV-2 seronegative per-protocol vaccine recipients in South Africa not experiencing the moderate to severe-critical COVID-19 endpoint or with evidence of SARS-CoV-2 infection through D29; no. cases = numbers of this cohort with an observed moderate to severe-critical COVID-19 endpoint (with onset ≥ 7 days post D29 and ≥ 28 days post vaccination).

† q-value and FWER (family-wise error rate) are computed over the set of p-values both for quantitative markers and categorical markers using the Westfall and Young permutation method (10000 replicates).

Supplementary Fig. 16. Moderate to severe-critical COVID-19 risk by D29 antibody marker tertile in participants in the United States. (A-C) Covariate-adjusted cumulative incidence of moderate to severe-critical COVID-19 by Low, Medium, and High tertiles of D29 **(A)** anti-Spike IgG concentration, **(B)** anti-RBD IgG concentration, or **(C)** neutralizing antibody titer (nAb-ID50) in baseline SARS-CoV-2–seronegative per-protocol vaccine recipients in Latin America. **(D)** Covariate-adjusted hazard ratio of moderate to severe-critical COVID-19 across D29 antibody marker tertiles. Endpoint counts for (**A-C**) calculated by inverse probability of sampling D29 marker weighting. The overall p value is from a generalized Wald test of whether the hazard rate of moderate to severe-critical COVID-19 differed across the Low, Medium, and High subgroups. Analyses adjusted for baseline behavioral risk score. BAU, binding antibody units; CI, confidence interval; FDR, false discovery rate; FWER, family-wise error rate; IU, international units; nAb-ID50, 50% inhibitory dilution neutralizing antibody; Pt. Est., point estimate.

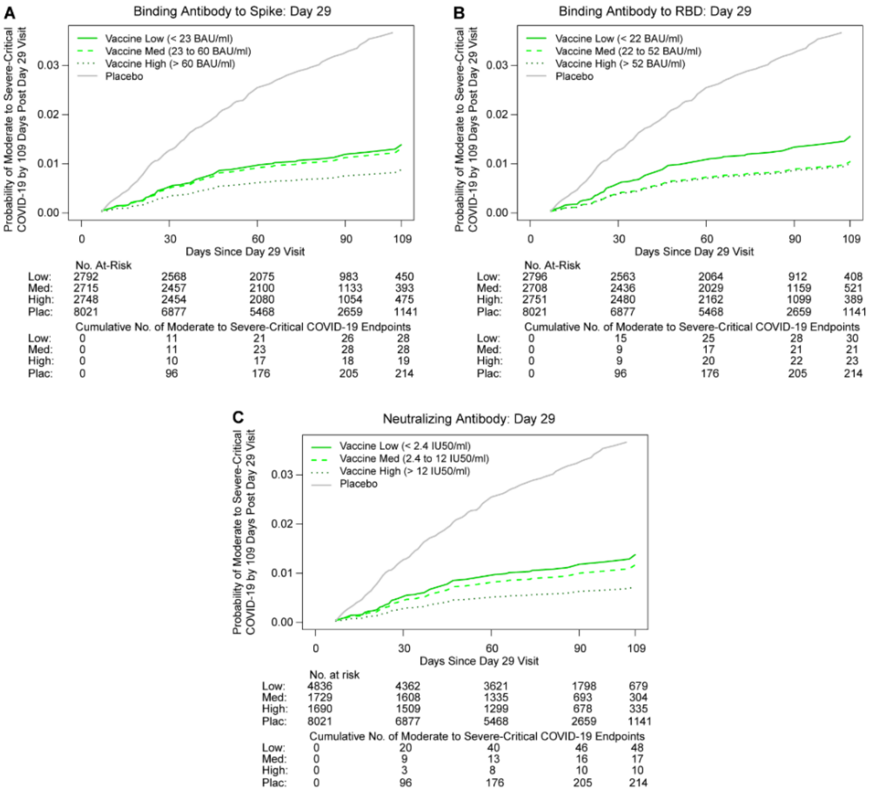

*No. At-Risk = estimated number in the population for analysis: baseline seronegative per-protocol vaccine recipients in the United States not experiencing the moderate to severe-critical COVID-19 endpoint or with any evidence of infection through D29.
**Cumulative No. of Moderate to Severe-Critical COVID-19 Endpoints = estimated cumulative number of this cohort with a moderate to severe-critical COVID-19 endpoint.

D

D29 Immunologic Marker	Tertile	No. cases / No. at-risk§	Attack rate	Haz. Ratio Pt. Est.	Haz. Ratio 95% CI	P-value (2-sided)	Overall P-value	FDR-adjusted P-value†	FWER-adjusted P-value†
Anti Spike IgG (BAU/ml)	Low	28/2,792	0.0100	1	N/A	N/A	0.388	0.391	0.550
	Medium	28/2,715	0.0103	0.94	(0.49, 1.82)	0.862			
	High	19/2,748	0.0069	0.63	(0.31, 1.27)	0.199			
Anti RBD IgG (BAU/ml)	Low	30/2,796	0.0107	1	N/A	N/A	0.366	0.391	0.550
	Medium	21/2,708	0.0078	0.67	(0.34, 1.32)	0.246			
	High	23/2,751	0.0084	0.65	(0.33, 1.28)	0.210			
nAb-ID50 (IU50/ml)	Low	48/4,836	0.0099	1	N/A	N/A	0.305	0.391	0.550
	Medium	17/1,729	0.0098	0.85	(0.42, 1.71)	0.642			
	High	10/1,690	0.0059	0.53	(0.24, 1.19)	0.124			
Placebo		214/8,021	0.0267						

Baseline covariates adjusted for: baseline risk score. RBD, receptor binding domain; ID50, 50% inhibitory dilution titer.
§ No. at-risk = estimated number in the population for analysis, i.e. baseline SARS-CoV-2 seronegative per-protocol vaccine recipients in the United States not experiencing the moderate to severe-critical COVID-19 endpoint or with evidence of SARS-CoV-2 infection through D29; no. cases = numbers of this cohort with an observed moderate to severe-critical COVID-19 endpoint (with onset ≥ 7 days post D29 and ≥ 28 days post vaccination).
† q-value and FWER (family-wise error rate) are computed over the set of p-values both for quantitative markers and categorical markers using the Westfall and Young permutation method (10000 replicates).

Supplementary Fig. 17. Severe-critical COVID-19 risk by D29 antibody marker level. Covariate-adjusted cumulative incidence of severe-critical COVID-19 by D29 **(A)** anti-Spike IgG concentration, **(B)** anti-RBD IgG concentration, or **(C)** neutralizing antibody titer (nAb-ID50) in baseline SARS-CoV-2–seronegative per-protocol vaccine recipients. Incidence is shown through 170 days post D29 and was estimated using a Cox model (purple) or a nonparametric method (blue). Dotted lines and shading indicate bootstrap pointwise 95% CIs. The upper and lower horizontal gray lines are the overall cumulative incidence of severe-critical COVID-19 from 1 to 170 days post D29 in placebo and vaccine recipients, respectively. The green histograms are estimates of the frequency distribution of each D29 antibody marker in baseline SARS-CoV-2 seronegative per-protocol vaccine recipients. Analyses adjusted for baseline behavioral risk score and geographic region. Nonparametric curves are plotted over the Spike IgG concentration range from negative response to the 90th percentile (125 BAU/ml), RBD IgG concentration range from negative response to the 90th percentile (118 BAU/ml), and nAb-ID50 titer range from unquantifiable to the 90th percentile (30.2 IU50/ml). Cox curves are plotted over the Spike IgG concentration range from negative response to the 97.5th percentile (238 BAU/ml), RBD IgG concentration range from negative response to the 97.5th percentile (173 BAU/ml), and nAb-ID50 titer range from unquantifiable to the 97.5th percentile (123 IU50/ml). Positivity cutoffs: 10.8424 BAU/ml for Spike and 14.0858 BAU/ml for RBD. nAb-ID50 LLOQ=4.8975 IU50/ml. BAU, binding antibody units; CI, confidence interval; IU, international units; LLOQ, lower limit of quantitation; nAb-ID50, 50% inhibitory dilution neutralizing antibody.

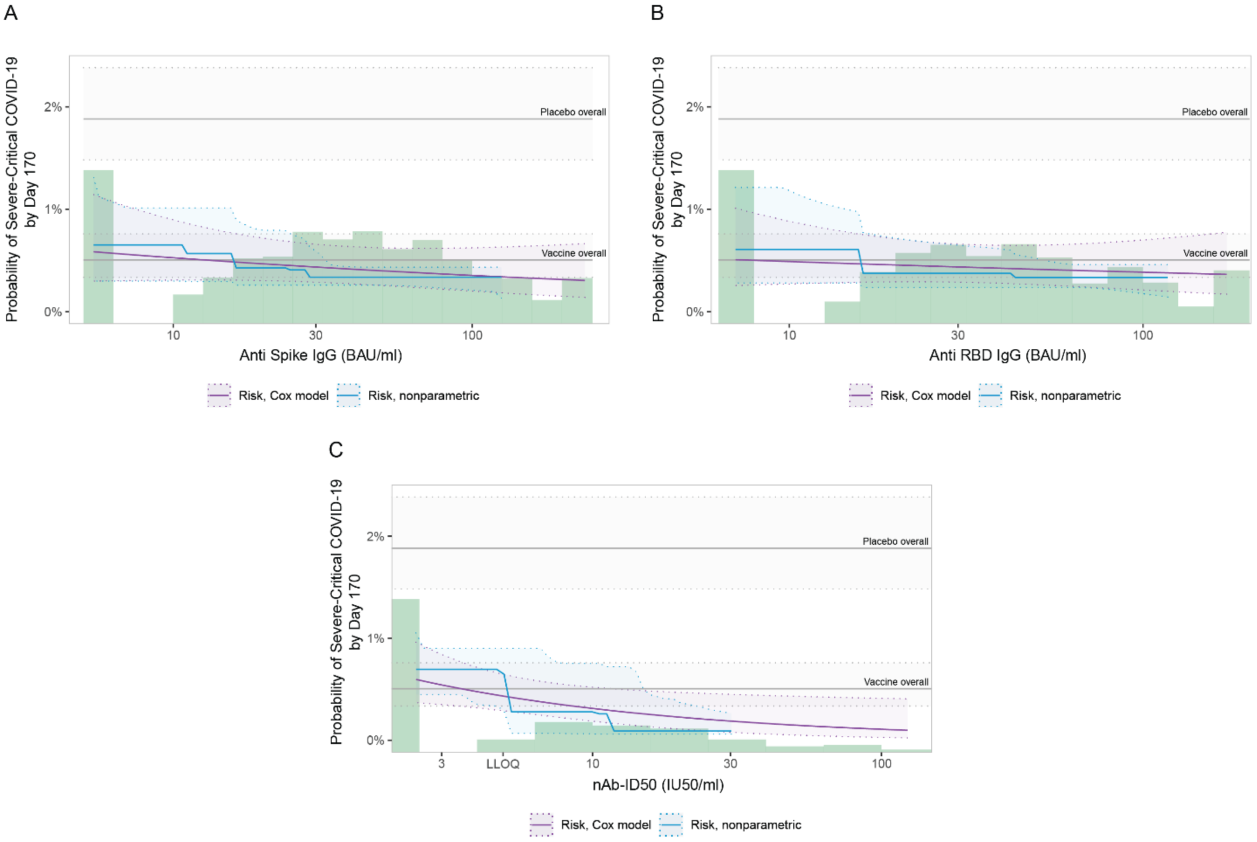

Supplementary Fig. 18. Moderate COVID-19 risk by D29 antibody marker level. Covariate-adjusted cumulative incidence of moderate COVID-19 by D29 **(A)** anti-Spike IgG concentration, **(B)** anti-RBD IgG concentration, or **(C)** neutralizing antibody titer (nAb-ID50) in baseline SARS-CoV-2–seronegative per-protocol vaccine recipients. Incidence is shown through 181 days post D29 and was estimated using a Cox model (purple) or a nonparametric method (blue). Dotted lines and shading indicate bootstrap pointwise 95% CIs. The upper and lower horizontal gray lines are the overall cumulative incidence of moderate COVID-19 from 1 to 181 days post D29 in placebo and vaccine recipients, respectively. The green histograms are estimates of the frequency distribution of each D29 antibody marker in baseline SARS-CoV-2 seronegative per-protocol vaccine recipients. Analyses adjusted for baseline behavioral risk score and geographic region. Nonparametric curves are plotted over the Spike IgG concentration range from negative response to the 90th percentile (125 BAU/ml), RBD IgG concentration range from negative response to the 90th percentile (118 BAU/ml), and nAb-ID50 titer range from unquantifiable to the 90th percentile (30.2 IU50/ml). Cox curves are plotted over the Spike IgG concentration range from negative response to the 97.5th percentile (238 BAU/ml), RBD IgG concentration range from negative response to the 97.5th percentile (173 BAU/ml), and nAb-ID50 titer range from unquantifiable to the 97.5th percentile (123 IU50/ml). Positivity cutoffs: 10.8424 BAU/ml for Spike and 14.0858 BAU/ml for RBD. nAb-ID50 LLOQ=4.8975 IU50/ml. BAU, binding antibody units; CI, confidence interval; IU, international units; LLOQ, lower limit of quantitation; nAb-ID50, 50% inhibitory dilution neutralizing antibody.

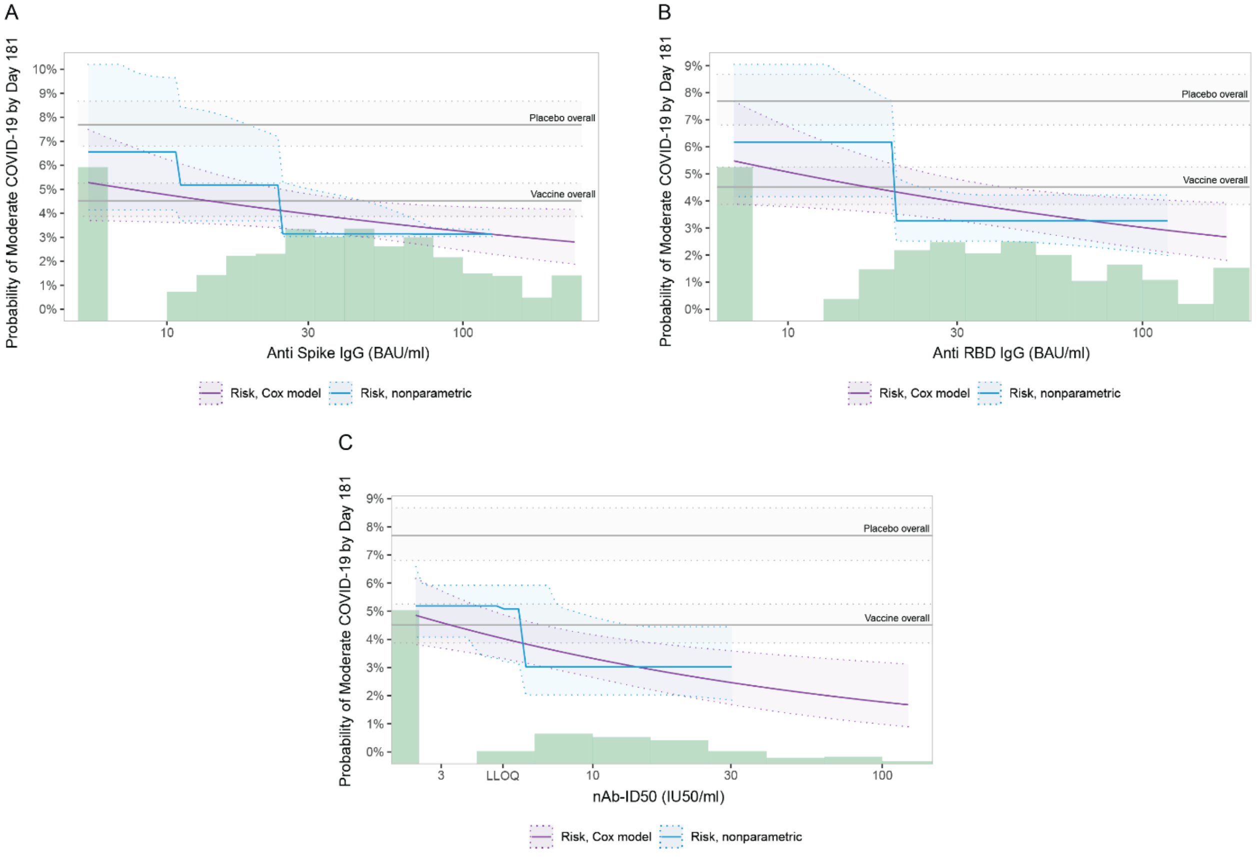

Supplementary Fig. 19. Moderate to severe-critical COVID-19 risk by D29 antibody marker level. Covariate-adjusted cumulative incidence of moderate to severe-critical COVID-19 by D29 **(A)** anti-Spike IgG concentration, **(B)** anti-RBD IgG concentration, or **(C)** neutralizing antibody titer (nAb-ID50) in baseline SARS-CoV-2–seronegative per-protocol vaccine recipients. Incidence is shown through 181 days post D29 and was estimated using a Cox model (purple) or a nonparametric method (blue). Dotted lines and shading indicate bootstrap pointwise 95% CIs. The upper and lower horizontal gray lines are the overall cumulative incidence of moderate to severe-critical COVID-19 from 1 to 181 days post D29 in placebo and vaccine recipients, respectively. The green histograms are estimates of the frequency distribution of each D29 antibody marker in baseline SARS-CoV-2 seronegative per-protocol vaccine recipients. Analyses adjusted for baseline behavioral risk score and geographic region. Nonparametric curves are plotted over the Spike IgG concentration range from negative response to the 90th percentile (125 BAU/ml), RBD IgG concentration range from negative response to the 90th percentile (118 BAU/ml), and nAb-ID50 titer range from unquantifiable to the 90th percentile (30.2 IU50/ml). Cox curves are plotted over the Spike IgG concentration range from negative response to the 97.5th percentile (238 BAU/ml), RBD IgG concentration range from negative response to the 97.5th percentile (173 BAU/ml), and nAb-ID50 titer range from unquantifiable to the 97.5th percentile (123 IU50/ml). Positivity cutoffs: 10.8424 BAU/ml for Spike and 14.0858 BAU/ml for RBD. nAb-ID50 LLOQ=4.8975 IU50/ml. BAU, binding antibody units; CI, confidence interval; IU, international units; LLOQ, lower limit of quantitation; nAb-ID50, 50% inhibitory dilution neutralizing antibody.

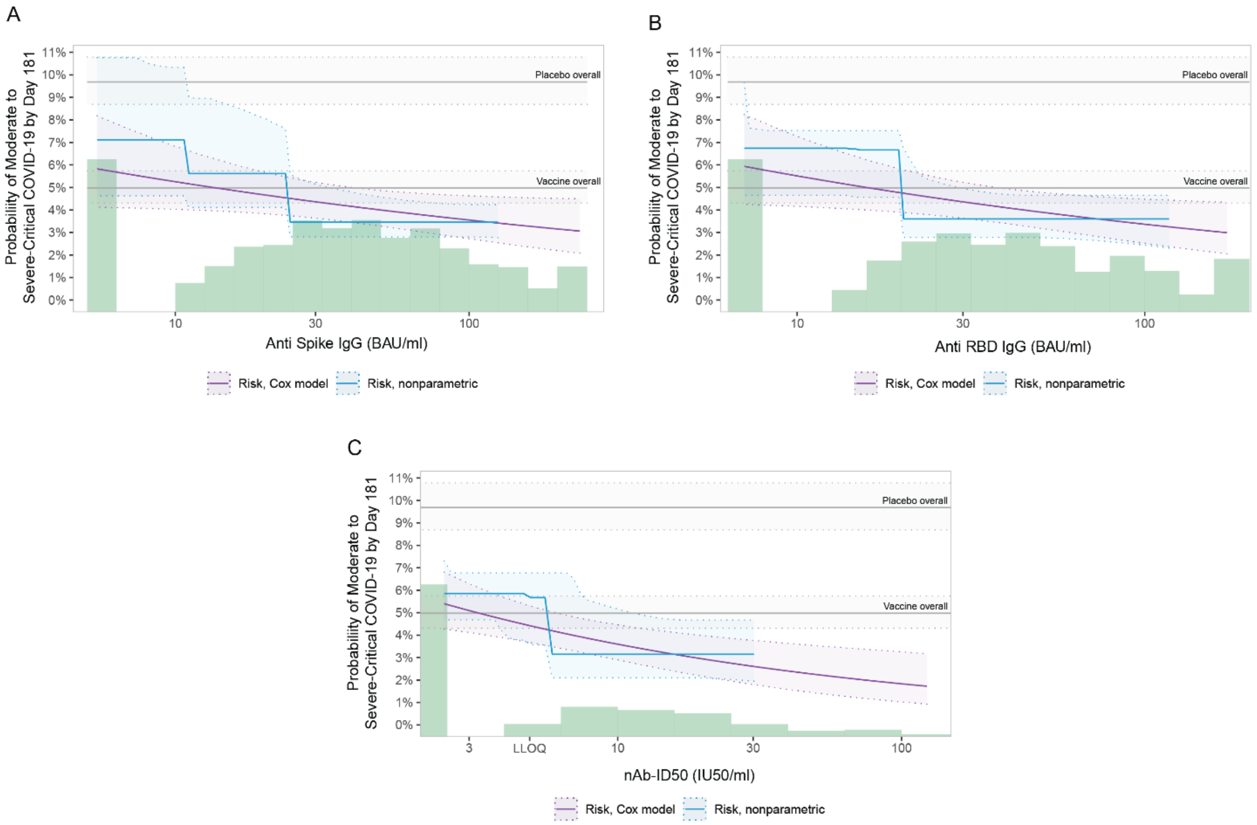

Supplementary Fig. 20. Severe-critical COVID-19 risk by D29 antibody marker level in participants in Latin America. Covariate-adjusted cumulative incidence of severe-critical COVID-19 by D29 **(A)** anti-Spike IgG concentration, **(B)** anti-RBD IgG concentration, or **(C)** neutralizing antibody titer (nAb-ID50) in baseline SARS-CoV-2–seronegative per-protocol vaccine recipients in Latin America. Incidence is shown through 170 days post D29 and was estimated using a Cox model (purple) or a nonparametric method (blue). Dotted lines and shading indicate bootstrap pointwise 95% CIs. The upper and lower horizontal gray lines are the overall cumulative incidence of severe-critical COVID-19 from 1 to 170 days post D29 in placebo and vaccine recipients, respectively, in Latin America. The green histograms are estimates of the frequency distribution of each D29 antibody marker in baseline SARS-CoV-2 seronegative per-protocol vaccine recipients in Latin America. Analyses adjusted for baseline behavioral risk score. Nonparametric curves are plotted over the Spike IgG concentration range from negative response to the 90th percentile (121 BAU/ml), RBD IgG concentration range from negative response to the 90th percentile (114 BAU/ml), and nAb-ID50 titer range from unquantifiable to the 90th percentile (28.5 IU50/ml). Cox curves are plotted over the Spike IgG concentration range from negative response to the 97.5th percentile (238 BAU/ml), RBD IgG concentration range from negative response to the 97.5th percentile (173 BAU/ml), and nAb-ID50 titer range from unquantifiable to the 97.5th percentile (97.2 IU50/ml). Positivity cutoffs: 10.8424 BAU/ml for Spike and 14.0858 BAU/ml for RBD. nAb-ID50 LLOQ=4.8975 IU50/ml. BAU, binding antibody units; CI, confidence interval; IU, international units; LLOQ, lower limit of quantitation; nAb-ID50, 50% inhibitory dilution neutralizing antibody.

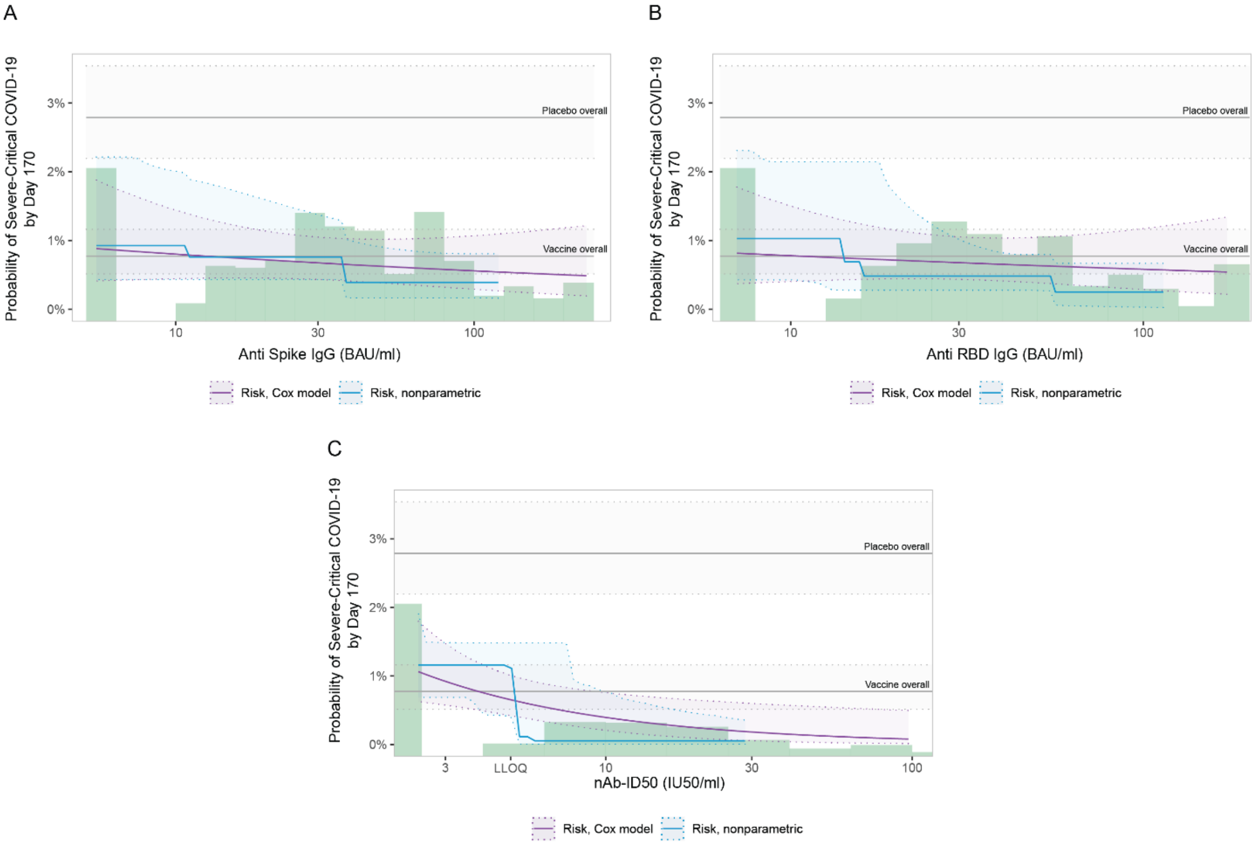

Supplementary Fig. 21. Moderate COVID-19 risk by D29 antibody marker level in participants in Latin America. Covariate-adjusted cumulative incidence of moderate COVID-19 by D29 **(A)** anti-Spike IgG concentration, **(B)** anti-RBD IgG concentration, or **(C)** neutralizing antibody titer (nAb-ID50) in baseline SARS-CoV-2–seronegative per-protocol vaccine recipients in Latin America. Incidence is shown through 181 days post D29 and was estimated using a Cox model (purple) or a nonparametric method (blue). Dotted lines and shading indicate bootstrap pointwise 95% CIs. The upper and lower horizontal gray lines are the overall cumulative incidence of moderate COVID-19 from 1 to 181 days post D29 in placebo and vaccine recipients, respectively, in Latin America. The green histograms are estimates of the frequency distribution of each D29 antibody marker in baseline SARS-CoV-2 seronegative per-protocol vaccine recipients in Latin America. Analyses adjusted for baseline behavioral risk score. Nonparametric curves are plotted over the Spike IgG concentration range from negative response to the 90th percentile (121 BAU/ml), RBD IgG concentration range from negative response to the 90th percentile (114 BAU/ml), and nAb-ID50 titer range from unquantifiable to the 90th percentile (28.5 IU50/ml). Cox curves are plotted over the Spike IgG concentration range from negative response to the 97.5th percentile (238 BAU/ml), RBD IgG concentration range from negative response to the 97.5th percentile (173 BAU/ml), and nAb-ID50 titer range from unquantifiable to the 97.5th percentile (97.2 IU50/ml). Positivity cutoffs: 10.8424 BAU/ml for Spike and 14.0858 BAU/ml for RBD. nAb-ID50 LLOQ=4.8975 IU50/ml. BAU, binding antibody units; CI, confidence interval; IU, international units; LLOQ, lower limit of quantitation; nAb-ID50, 50% inhibitory dilution neutralizing antibody.

A

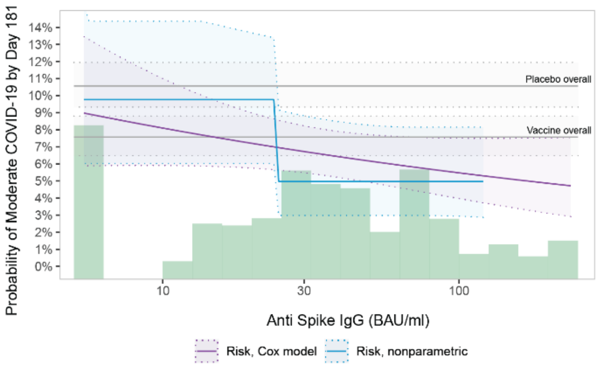

B

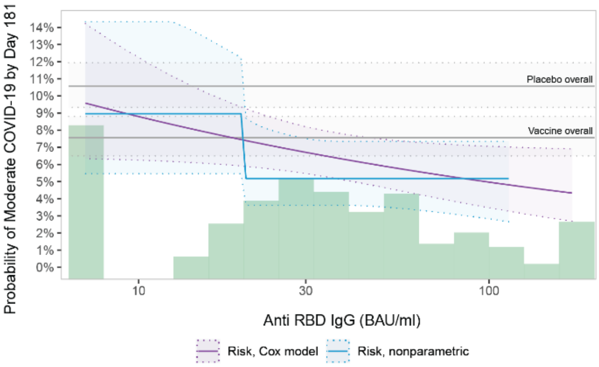

C

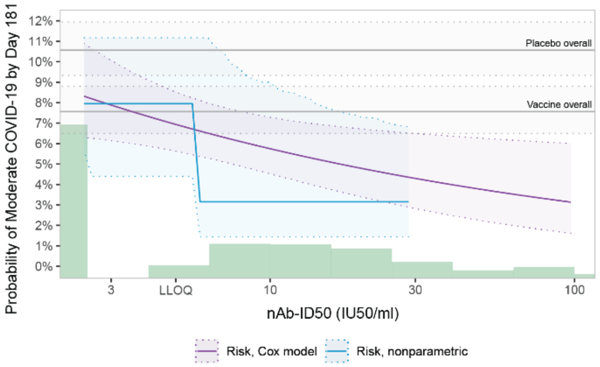

Supplementary Fig. 22. Moderate to severe-critical COVID-19 risk by D29 antibody marker level in participants in Latin America. Covariate-adjusted cumulative incidence of moderate to severe-critical COVID-19 by D29 **(A)** anti-Spike IgG concentration, **(B)** anti-RBD IgG concentration, or **(C)** neutralizing antibody titer (nAb-ID50) in baseline SARS-CoV-2–seronegative per-protocol vaccine recipients in Latin America. Incidence is shown through 181 days post D29 and was estimated using a Cox model (purple) or a nonparametric method (blue). Dotted lines and shading indicate bootstrap pointwise 95% CIs. The upper and lower horizontal gray lines are the overall cumulative incidence of moderate to severe-critical COVID-19 from 1 to 181 days post D29 in placebo and vaccine recipients, respectively, in Latin America. The green histograms are estimates of the frequency distribution of each D29 antibody marker in baseline SARS-CoV-2 seronegative per-protocol vaccine recipients in Latin America. Analyses adjusted for baseline behavioral risk score. Nonparametric curves are plotted over the Spike IgG concentration range from negative response to the 90th percentile (121 BAU/ml), RBD IgG concentration range from negative response to the 90th percentile (114 BAU/ml), and nAb-ID50 titer range from unquantifiable to the 90th percentile (28.5 IU50/ml). Cox curves are plotted over the Spike IgG concentration range from negative response to the 97.5th percentile (238 BAU/ml), RBD IgG concentration range from negative response to the 97.5th percentile (173 BAU/ml), and nAb-ID50 titer range from unquantifiable to the 97.5th percentile (97.2 IU50/ml). Positivity cutoffs: 10.8424 BAU/ml for Spike and 14.0858 BAU/ml for RBD. nAb-ID50 LLOQ=4.8975 IU50/ml. BAU, binding antibody units; CI, confidence interval; IU, international units; LLOQ, lower limit of quantitation; nAb-ID50, 50% inhibitory dilution neutralizing antibody.

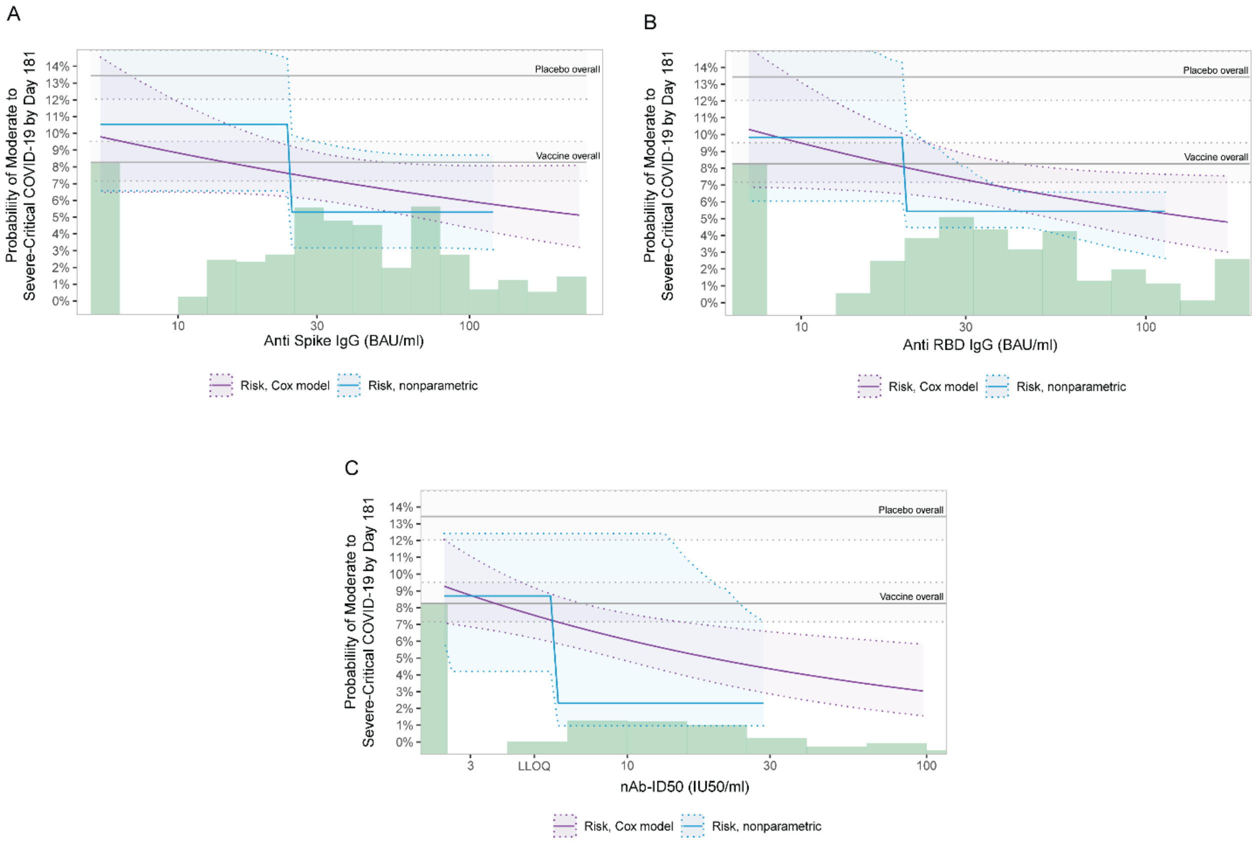

Supplementary Fig. 23. Moderate to severe-critical COVID-19 risk by D29 antibody marker level in participants in South Africa. Covariate-adjusted cumulative incidence of moderate to severe-critical COVID-19 by D29 **(A)** anti-Spike IgG concentration, **(B)** anti-RBD IgG concentration, or **(C)** neutralizing antibody titer (nAb-ID50) in baseline SARS-CoV-2–seronegative per-protocol vaccine recipients in South Africa. Incidence is shown through 101 days post D29 and was estimated using a Cox model (purple) or a nonparametric method (blue). Dotted lines and shading indicate bootstrap pointwise 95% CIs. The upper and lower horizontal gray lines are the overall cumulative incidence of moderate to severe-critical COVID-19 from 1 to 101 days post D29 in placebo and vaccine recipients, respectively, in South Africa. The green histograms are estimates of the frequency distribution of each D29 antibody marker in baseline SARS-CoV-2 seronegative per-protocol vaccine recipients in South Africa. Analyses adjusted for baseline behavioral risk score. Nonparametric curves are plotted over the Spike IgG concentration range from negative response to the 90th percentile (125 BAU/ml), RBD IgG concentration range from negative response to the 90th percentile (125 BAU/ml), and nAb-ID50 titer range from unquantifiable to the 90th percentile (30.2 IU50/ml). Cox curves are plotted over the Spike IgG concentration range from negative response to the 97.5th percentile (238 BAU/ml), RBD IgG concentration range from negative response to the 97.5th percentile (173 BAU/ml), and nAb-ID50 titer range from unquantifiable to the 97.5th percentile (91.7 IU50/ml). Positivity cutoffs: 10.8424 BAU/ml for Spike and 14.0858 BAU/ml for RBD. nAb-ID50 LLOQ=4.8975 IU50/ml. BAU, binding antibody units; CI, confidence interval; IU, international units; LLOQ, lower limit of quantitation; nAb-ID50, 50% inhibitory dilution neutralizing antibody.

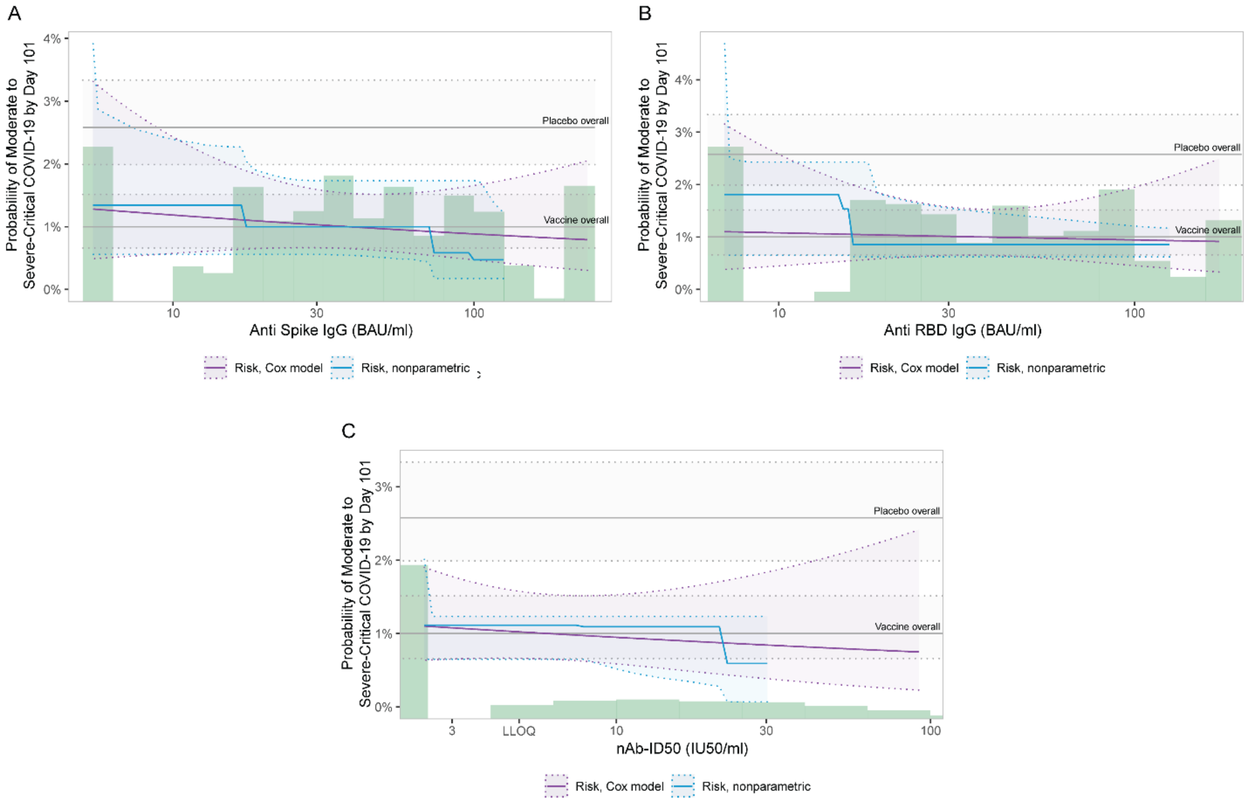

Supplementary Fig. 24. Moderate to severe-critical COVID-19 risk by D29 antibody marker level in participants in the United States. Covariate-adjusted cumulative incidence of moderate to severe-critical COVID-19 by D29 **(A)** anti-Spike IgG concentration, **(B)** anti-RBD IgG concentration, or **(C)** neutralizing antibody titer (nAb-ID50) in baseline SARS-CoV-2–seronegative per-protocol vaccine recipients in the United States. Incidence is shown through 109 days post D29 and was estimated using a Cox model (purple) or a nonparametric method (blue). Dotted lines and shading indicate bootstrap pointwise 95% CIs. The upper and lower horizontal gray lines are the overall cumulative incidence of moderate to severe-critical COVID-19 from 1 to 109 days post D29 in placebo and vaccine recipients, respectively, in the United States. The green histograms are estimates of the frequency distribution of each D29 antibody marker in baseline SARS-CoV-2 seronegative per-protocol vaccine recipients in the United States. Analyses adjusted for baseline behavioral risk score. Nonparametric curves are plotted over the Spike IgG concentration range from negative response to the 90th percentile (130 BAU/ml), RBD IgG concentration range from negative response to the 90th percentile (118 BAU/ml), and nAb-ID50 titer range from unquantifiable to the 90th percentile (30.2 IU50/ml). Cox curves are plotted over the Spike IgG concentration range from negative response to the 97.5th percentile (213 BAU/ml), RBD IgG concentration range from negative response to the 97.5th percentile (173 BAU/ml), and nAb-ID50 titer range from unquantifiable to the 97.5th percentile (164 IU50/ml). Positivity cutoffs: 10.8424 BAU/ml for Spike and 14.0858 BAU/ml for RBD. nAb-ID50 LLOQ=4.8975 IU50/ml. BAU, binding antibody units; CI, confidence interval; IU, international units; LLOQ, lower limit of quantitation; nAb-ID50, 50% inhibitory dilution neutralizing antibody.

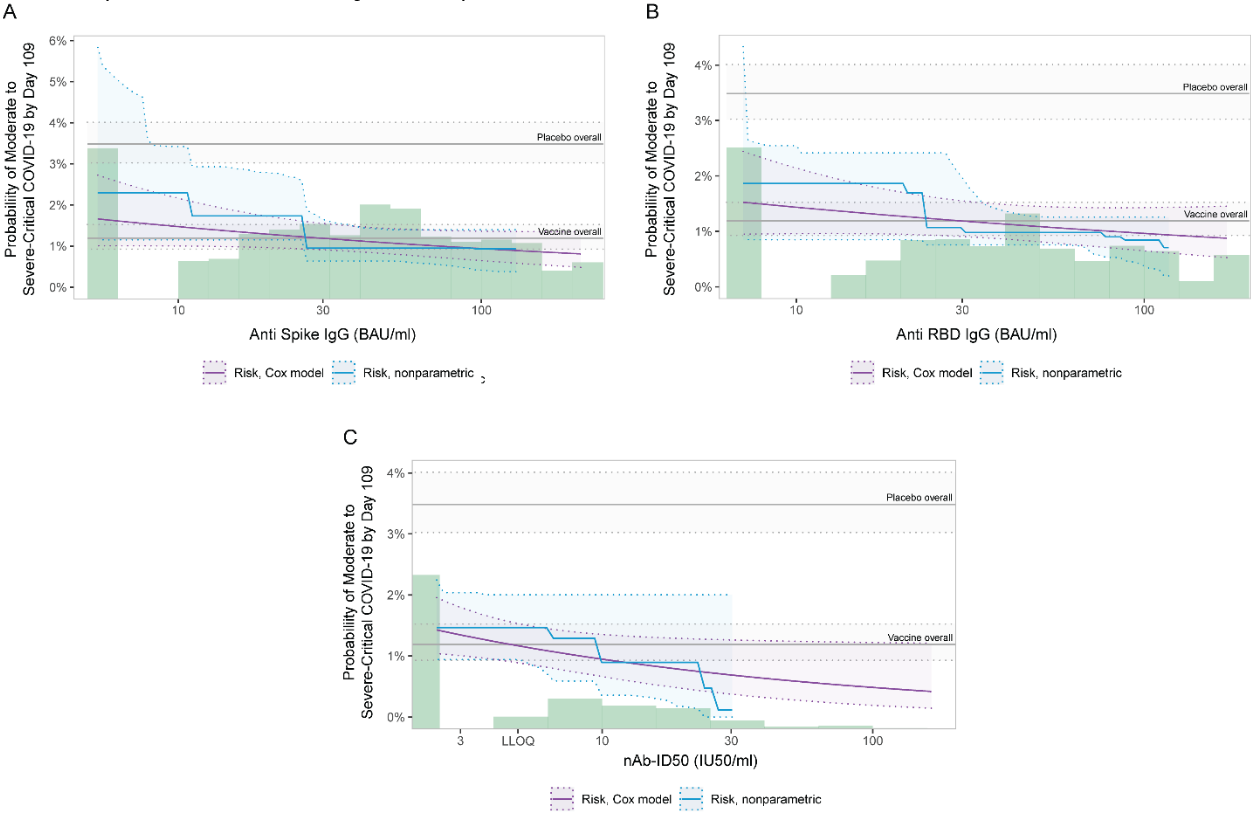

Supplementary Fig. 25. Severe-critical COVID-19 risk by D29 antibody marker level *above* a threshold. Covariate-adjusted cumulative incidence of severe-critical COVID-19 by D29 **(A)** anti-Spike IgG concentration, **(B)** anti-RBD IgG concentration, or **(C)** neutralizing antibody titer (nAb-ID50) *above* a threshold in baseline SARS-CoV-2–seronegative per-protocol vaccine recipients. Incidence is shown through 170 days post D29. Blue dots are point estimates at each severe-critical COVID-19 endpoint linearly interpolated as shown by solid black lines; the grey shaded area indicates pointwise 95% CIs. The estimates and CIs were adjusted using the assumption that the true threshold-response is non-increasing. The upper boundary of the green shaded area is the estimate of the reverse cumulative distribution function (CDF) of D29 antibody marker level. The vertical red dashed line is the D29 antibody marker threshold above which no severe-critical COVID-19 endpoints occurred. Analyses adjusted for baseline behavioral risk score and geographic region. POS, positivity cutoff: 10.8424 BAU/ml for Spike and 14.0858 BAU/ml for RBD. LLOQ, lower limit of quantitation = 4.8975 IU50/ml for nAb-ID50.

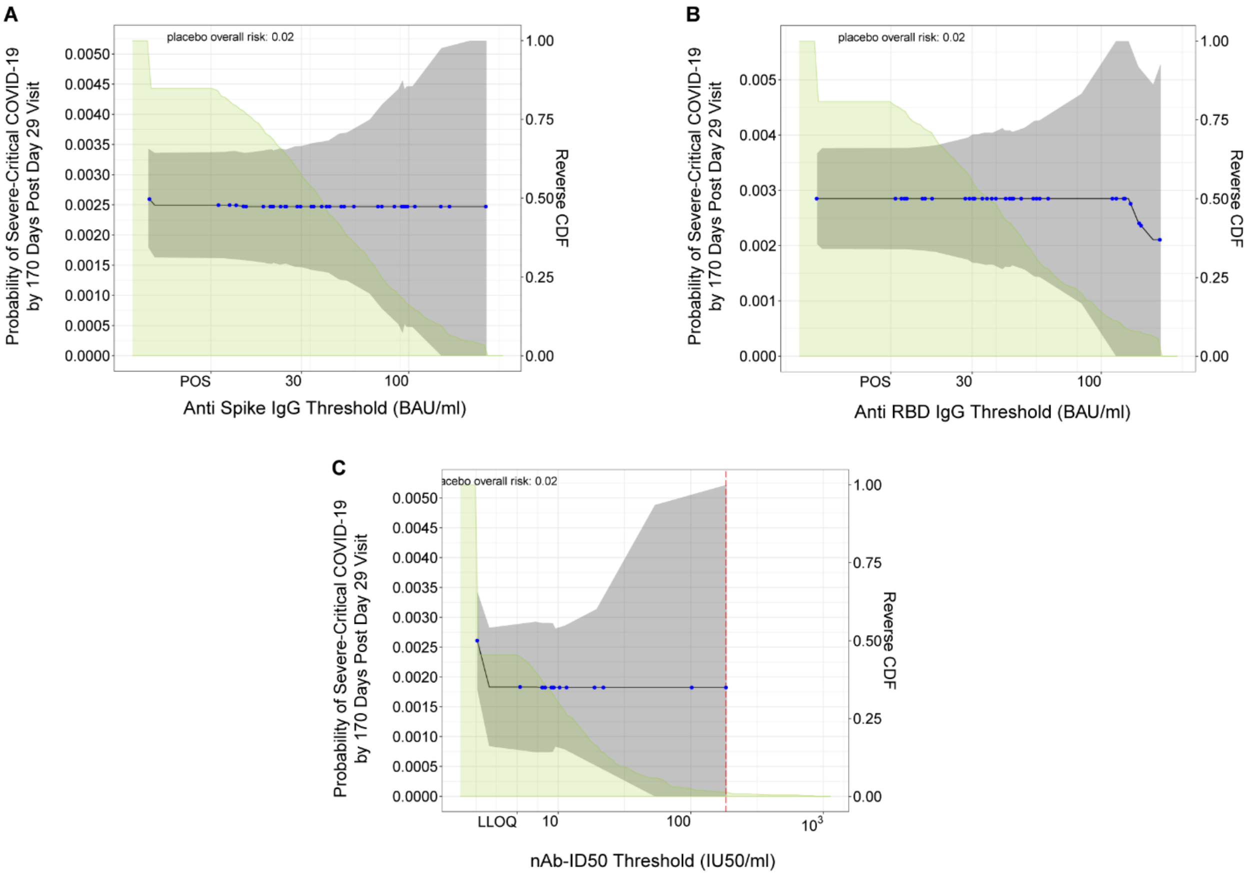

Supplementary Fig. 26. Moderate COVID-19 risk by D29 antibody marker level *above* a threshold. Covariate-adjusted cumulative incidence of moderate COVID-19 by D29 **(A)** anti-Spike IgG concentration, **(B)** anti-RBD IgG concentration, or **(C)** neutralizing antibody titer (nAb-ID50) *above* a threshold in baseline SARS-CoV-2–seronegative per-protocol vaccine recipients. Incidence is shown through 181 days post D29. Blue dots are point estimates at each moderate COVID-19 endpoint linearly interpolated as shown by solid black lines; the grey shaded area indicates pointwise 95% CIs. The estimates and CIs were adjusted using the assumption that the true threshold-response is non-increasing. The upper boundary of the green shaded area is the estimate of the reverse cumulative distribution function (CDF) of D29 antibody marker level. The vertical red dashed line is the D29 antibody marker threshold above which no moderate COVID-19 endpoints occurred. Analyses adjusted for baseline behavioral risk score and geographic region. POS, positivity cutoff: 10.8424 BAU/ml for Spike and 14.0858 BAU/ml for RBD. LLOQ, lower limit of quantitation = 4.8975 IU50/ml for nAb-ID50.

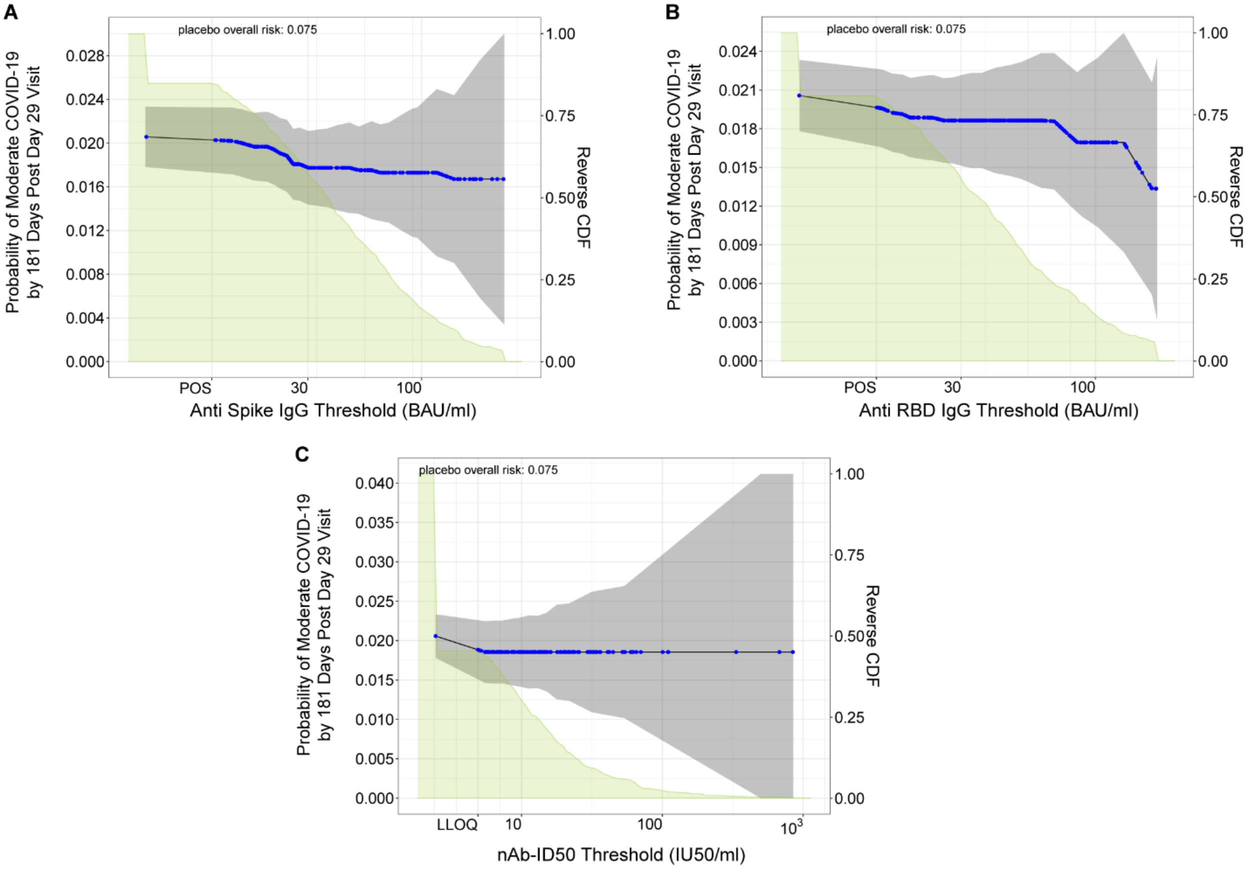

Supplementary Fig. 27. Moderate to severe-critical COVID-19 risk by D29 antibody marker level *above* a threshold. Covariate-adjusted cumulative incidence of moderate to severe-critical COVID-19 by D29 **(A)** anti-Spike IgG concentration, **(B)** anti-RBD IgG concentration, or **(C)** neutralizing antibody titer (nAb-ID50) *above* a threshold in baseline SARS-CoV-2–seronegative per-protocol vaccine recipients. Incidence is shown through 181 days post D29. Blue dots are point estimates at each moderate to severe-critical COVID-19 endpoint linearly interpolated as shown by solid black lines; the grey shaded area indicates pointwise 95% CIs. The estimates and CIs were adjusted using the assumption that the true threshold-response is non-increasing. The upper boundary of the green shaded area is the estimate of the reverse cumulative distribution function (CDF) of D29 antibody marker level. The vertical red dashed line is the D29 antibody marker threshold above which no moderate to severe-critical COVID-19 endpoints occurred. Analyses adjusted for baseline behavioral risk score and geographic region. POS, positivity cutoff: 10.8424 BAU/ml for Spike and 14.0858 BAU/ml for RBD. LLOQ, lower limit of quantitation = 4.8975 IU50/ml for nAb-ID50.

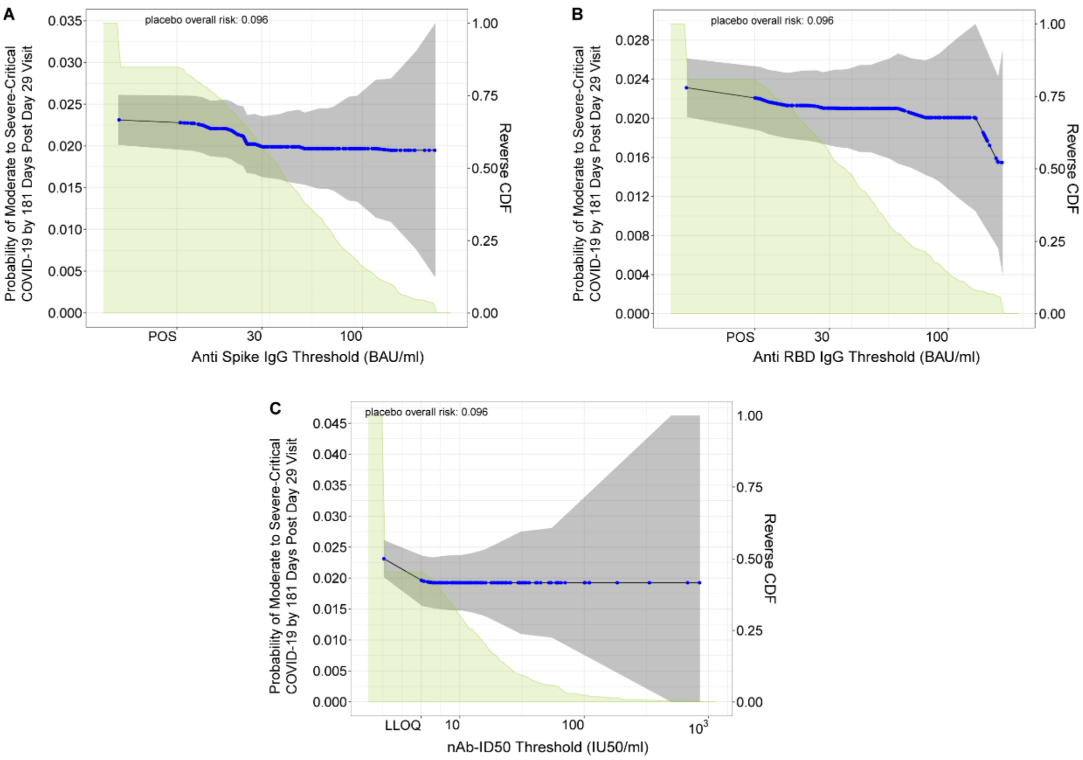

Supplementary Fig. 28. Severe-critical COVID-19 risk by D29 antibody marker level *above* a threshold in participants in Latin America. Covariate-adjusted cumulative incidence of severe-critical COVID-19 by D29 **(A)** anti-Spike IgG concentration, **(B)** anti-RBD IgG concentration, or **(C)** neutralizing antibody titer (nAb-ID50) *above* a threshold in baseline SARS-CoV-2–seronegative per-protocol vaccine recipients in Latin America. Incidence is shown through 170 days post D29. Blue dots are point estimates at each severe-critical COVID-19 endpoint linearly interpolated as shown by solid black lines; the grey shaded area indicates pointwise 95% CIs. The estimates and CIs were adjusted using the assumption that the true threshold-response is non-increasing. The upper boundary of the green shaded area is the estimate of the reverse cumulative distribution function (CDF) of D29 antibody marker level. The vertical red dashed line is the D29 antibody marker threshold above which no severe-critical COVID-19 endpoints occurred. Analyses adjusted for baseline behavioral risk score. POS, positivity cutoff: 10.8424 BAU/ml for Spike and 14.0858 BAU/ml for RBD. LLOQ, lower limit of quantitation = 4.8975 IU50/ml for nAb-ID50.

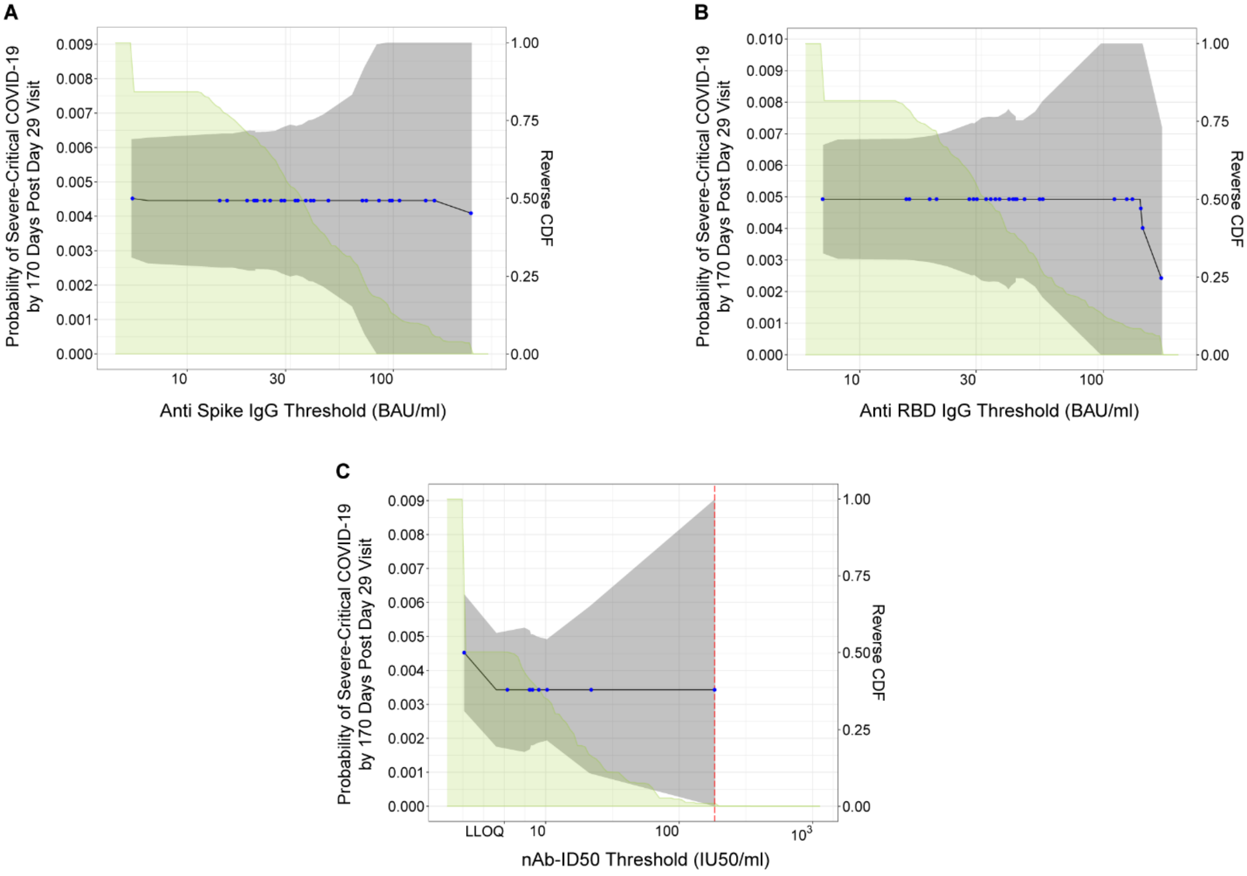

Supplementary Fig. 29. Moderate COVID-19 risk by D29 antibody marker level *above* a threshold in participants in Latin America. Covariate-adjusted cumulative incidence of moderate COVID-19 by D29 **(A)** anti-Spike IgG concentration, **(B)** anti-RBD IgG concentration, or **(C)** neutralizing antibody titer (nAb-ID50) *above* a threshold in baseline SARS-CoV-2–seronegative per-protocol vaccine recipients in Latin America. Incidence is shown through 181 days post D29. Blue dots are point estimates at each moderate COVID-19 endpoint linearly interpolated as shown by solid black lines; the grey shaded area indicates pointwise 95% CIs. The estimates and CIs were adjusted using the assumption that the true threshold-response is non-increasing. The upper boundary of the green shaded area is the estimate of the reverse cumulative distribution function (CDF) of D29 antibody marker level. The vertical red dashed line is the D29 antibody marker threshold above which no moderate COVID-19 endpoints occurred. Analyses adjusted for baseline behavioral risk score. POS, positivity cutoff: 10.8424 BAU/ml for Spike and 14.0858 BAU/ml for RBD. LLOQ, lower limit of quantitation = 4.8975 IU50/ml for nAb-ID50.

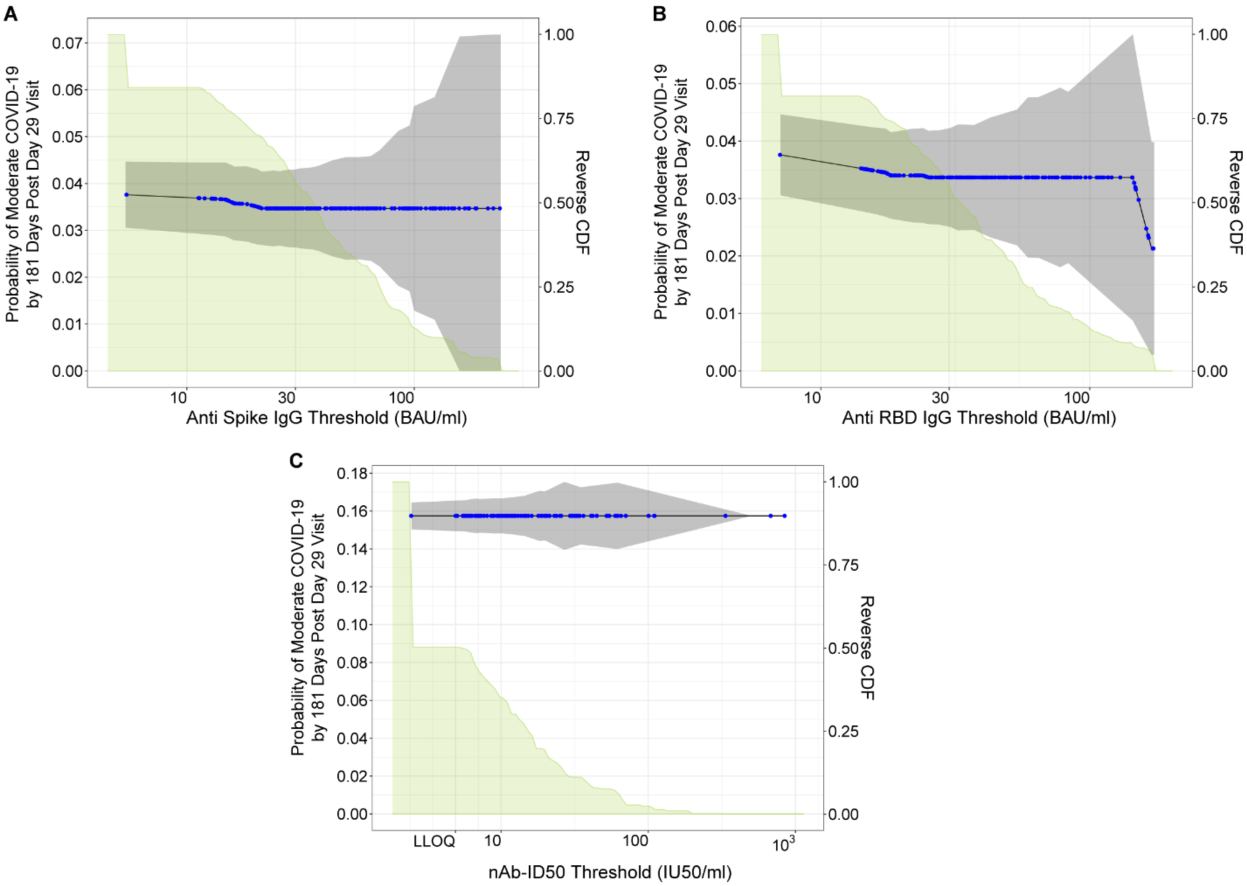

Supplementary Fig. 30. Moderate to severe-critical COVID-19 risk by D29 antibody marker level *above* a threshold in participants in Latin America. Covariate-adjusted cumulative incidence of moderate to severe-critical COVID-19 by D29 **(A)** anti-Spike IgG concentration, **(B)** anti-RBD IgG concentration, or **(C)** neutralizing antibody titer (nAb-ID50) *above* a threshold in baseline SARS-CoV-2–seronegative per-protocol vaccine recipients in Latin America. Incidence is shown through 181 days post D29. Blue dots are point estimates at each moderate to severe-critical COVID-19 endpoint linearly interpolated as shown by solid black lines; the grey shaded area indicates pointwise 95% CIs. The estimates and CIs were adjusted using the assumption that the true threshold-response is non-increasing. The upper boundary of the green shaded area is the estimate of the reverse cumulative distribution function (CDF) of D29 antibody marker level. The vertical red dashed line is the D29 antibody marker threshold above which no moderate to severe-critical COVID-19 endpoints occurred. Analyses adjusted for baseline behavioral risk score. POS, positivity cutoff: 10.8424 BAU/ml for Spike and 14.0858 BAU/ml for RBD. LLOQ, lower limit of quantitation = 4.8975 IU50/ml for nAb-ID50.

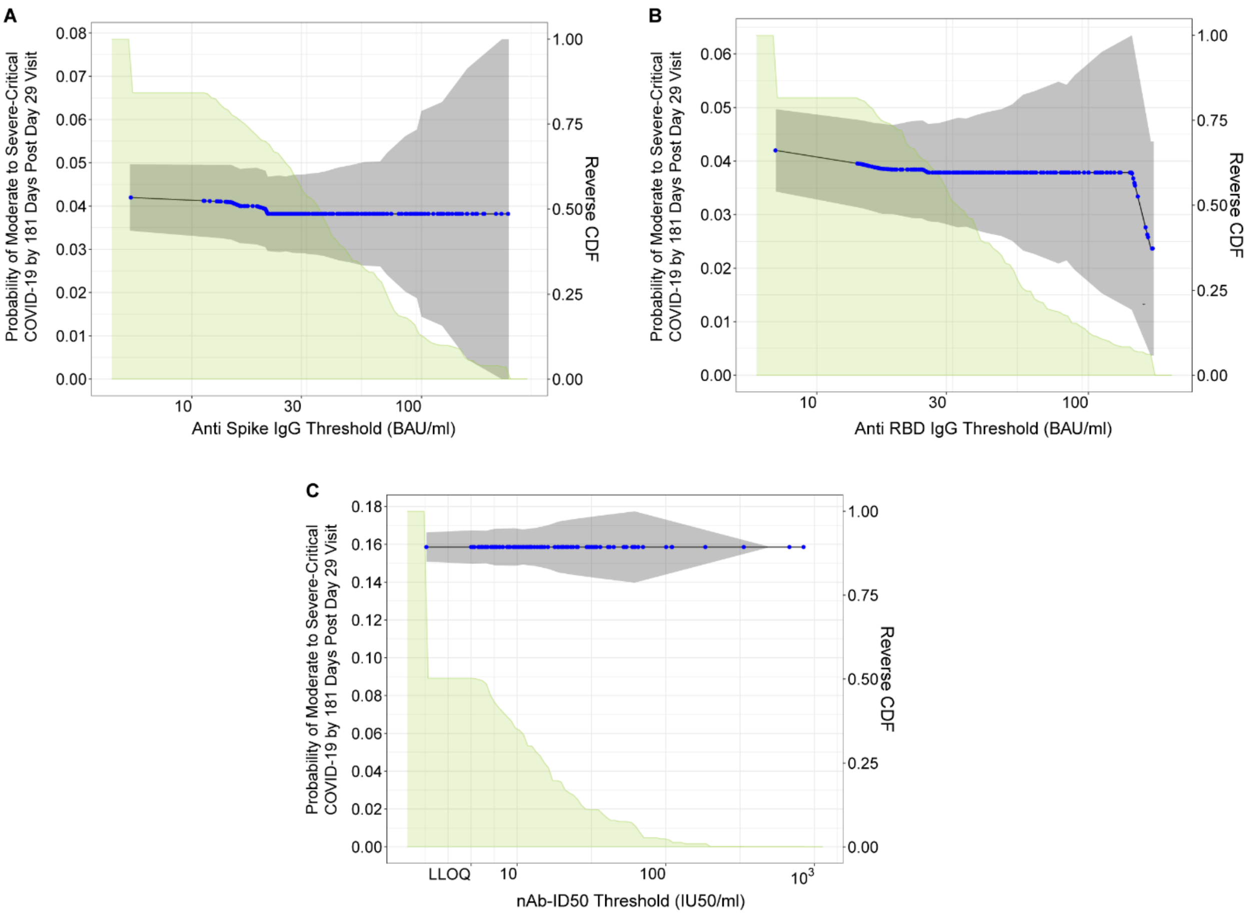

Supplementary Fig. 31. Moderate to severe-critical COVID-19 risk by D29 antibody marker level *above* a threshold in participants in South Africa. Covariate-adjusted cumulative incidence of moderate to severe-critical COVID-19 by D29 **(A)** anti-Spike IgG concentration, **(B)** anti-RBD IgG concentration, or **(C)** neutralizing antibody titer (nAb-ID50) *above* a threshold in baseline SARS-CoV-2–seronegative per-protocol vaccine recipients in South Africa. Incidence is shown through 101 days post D29. Blue dots are point estimates at each moderate to severe-critical COVID-19 endpoint linearly interpolated as shown by solid black lines; the grey shaded area indicates pointwise 95% CIs. The estimates and CIs were adjusted using the assumption that the true threshold-response is non-increasing. The upper boundary of the green shaded area is the estimate of the reverse cumulative distribution function (CDF) of D29 antibody marker level. The vertical red dashed line is the D29 antibody marker threshold above which no moderate to severe-critical COVID-19 endpoints occurred. Analyses adjusted for baseline behavioral risk score. POS, positivity cutoff: 10.8424 BAU/ml for Spike and 14.0858 BAU/ml for RBD. LLOQ, lower limit of quantitation = 4.8975 IU50/ml for nAb-ID50.

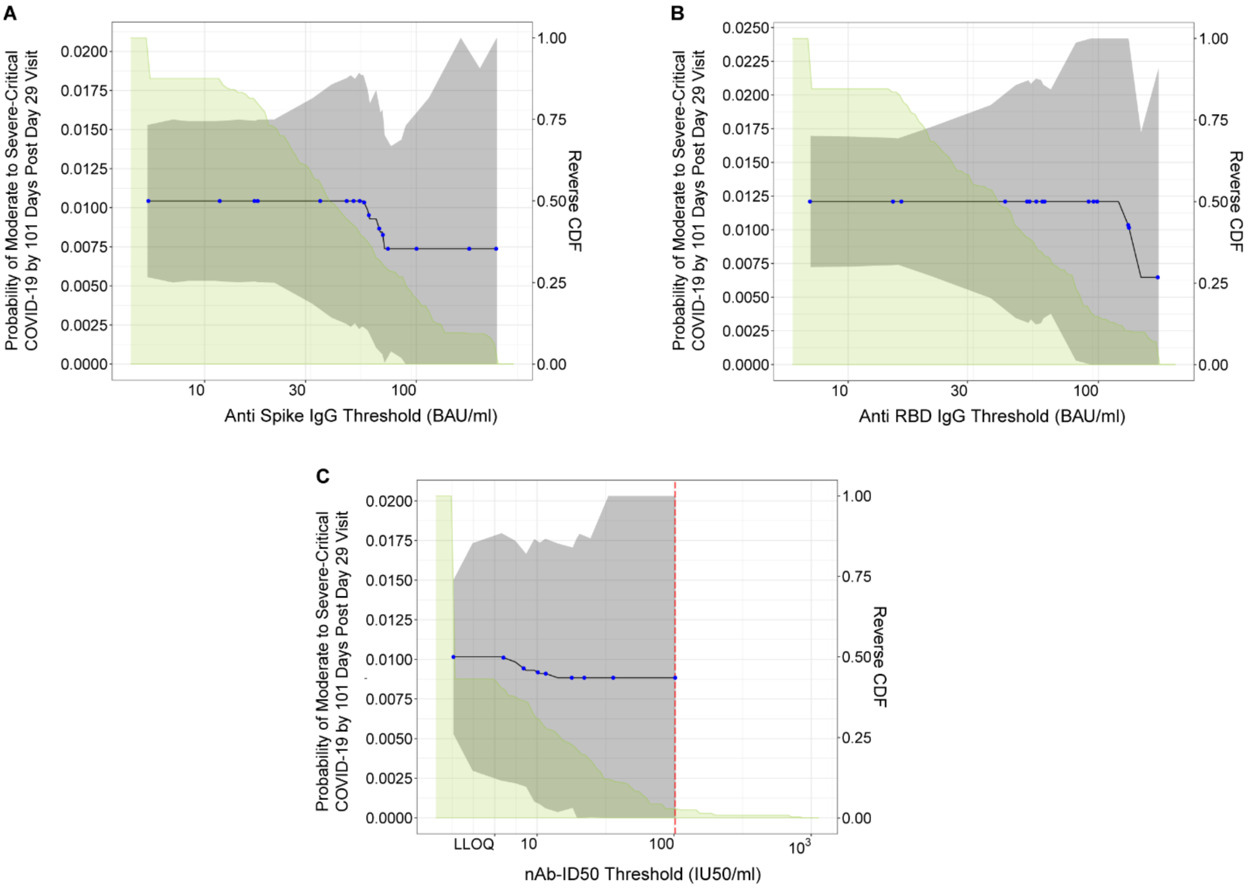

Supplementary Fig. 32. Moderate to severe-critical COVID-19 risk by D29 antibody marker level *above* a threshold in participants in the United States. Covariate-adjusted cumulative incidence of moderate to severe-critical COVID-19 by D29 **(A)** anti-Spike IgG concentration, **(B)** anti-RBD IgG concentration, or **(C)** neutralizing antibody titer (nAb-ID50) *above* a threshold in baseline SARS-CoV-2–seronegative per-protocol vaccine recipients in the United States. Incidence is shown through 109 days post D29. Blue dots are point estimates at each moderate to severe-critical COVID-19 endpoint linearly interpolated as shown by solid black lines; the grey shaded area indicates pointwise 95% CIs. The estimates and CIs were adjusted using the assumption that the true threshold-response is non-increasing. The upper boundary of the green shaded area is the estimate of the reverse cumulative distribution function (CDF) of D29 antibody marker level. The vertical red dashed line is the D29 antibody marker threshold above which no moderate to severe-critical COVID-19 endpoints occurred. Analyses adjusted for baseline behavioral risk score. POS, positivity cutoff: 10.8424 BAU/ml for Spike and 14.0858 BAU/ml for RBD. LLOQ, lower limit of quantitation = 4.8975 IU50/ml for nAb-ID50.

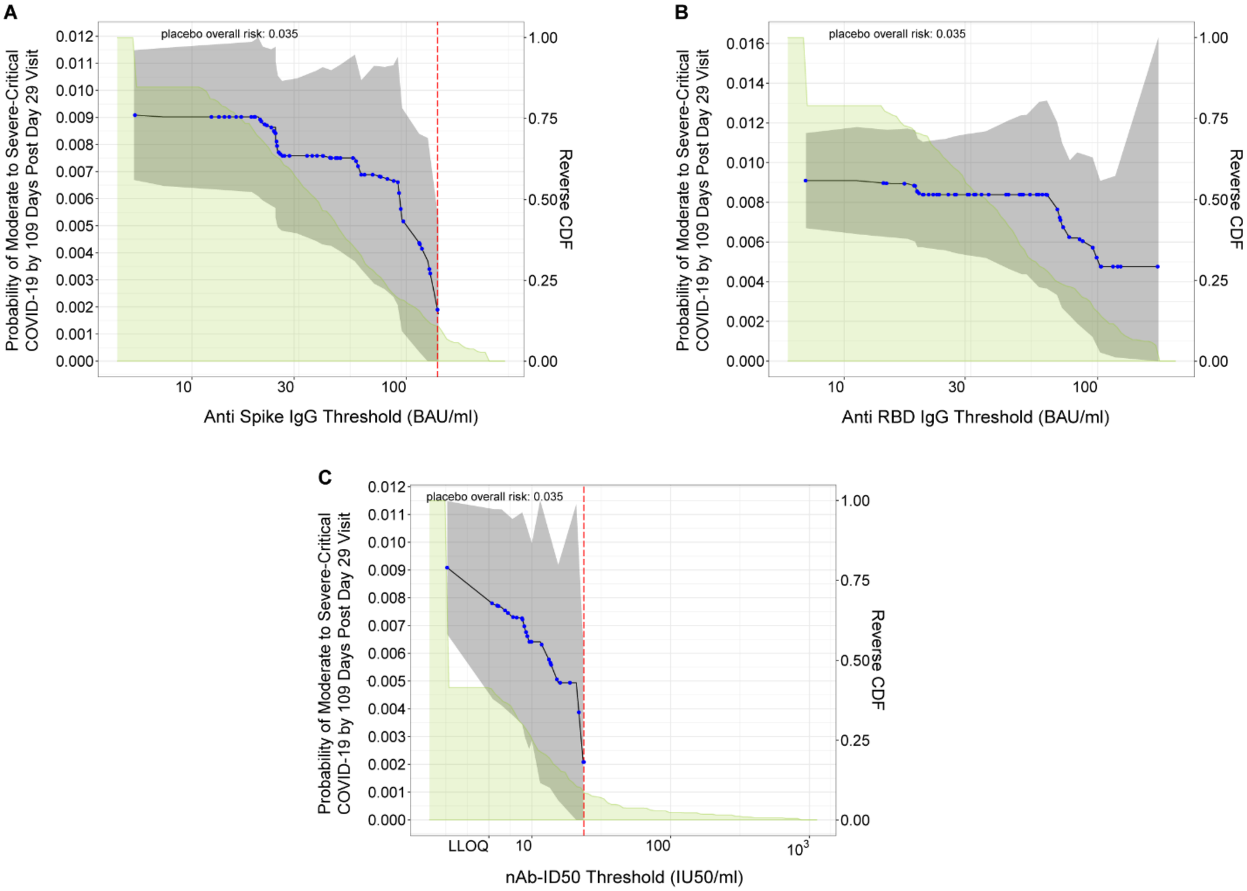

Supplementary Fig. 33. Vaccine efficacy against severe-critical COVID-19 by D29 antibody marker level. Analyses were performed in baseline SARS-CoV-2 seronegative per-protocol vaccine recipients. Estimates were obtained using (solid purple line) a Cox proportional hazards implementation of Gilbert et al.[1] or (solid blue line) a nonparametric implementation of Gilbert et al.[1] Each point on the curve represents the vaccine efficacy at the given D29 antibody marker level: **(A)** anti-Spike IgG concentration, **(B)** anti-RBD IgG concentration, **(C)** neutralizing antibody titer (nAb-ID50). The dotted lines indicate bootstrap pointwise 95% CIs. The green histograms are estimates of the frequency distribution of each D29 antibody marker, with the maroon dots representing the marker levels of the individual cases. Analyses adjusted for baseline behavioral risk score and geographic region. Nonparametric curves are plotted over the Spike IgG concentration range from negative response to the 90th percentile (125 BAU/ml), RBD IgG concentration range from negative response to the 90th percentile (118 BAU/ml), and nAb-ID50 titer range from unquantifiable to the 90th percentile (30.2 IU50/ml). Cox curves are plotted over the Spike IgG concentration range from negative response to the 97.5th percentile (238 BAU/ml), RBD IgG concentration range from negative response to the 97.5th percentile (173 BAU/ml), and nAb-ID50 titer range from unquantifiable to the 97.5th percentile (123 IU50/ml). Positivity cutoffs: 10.8424 BAU/ml for Spike and 14.0858 BAU/ml for RBD. nAb-ID50 LLOQ=4.8975 IU50/ml. BAU, binding antibody units; CI, confidence interval; IU, international units; LLOQ, lower limit of quantitation; nAb-ID50, 50% inhibitory dilution neutralizing antibody.

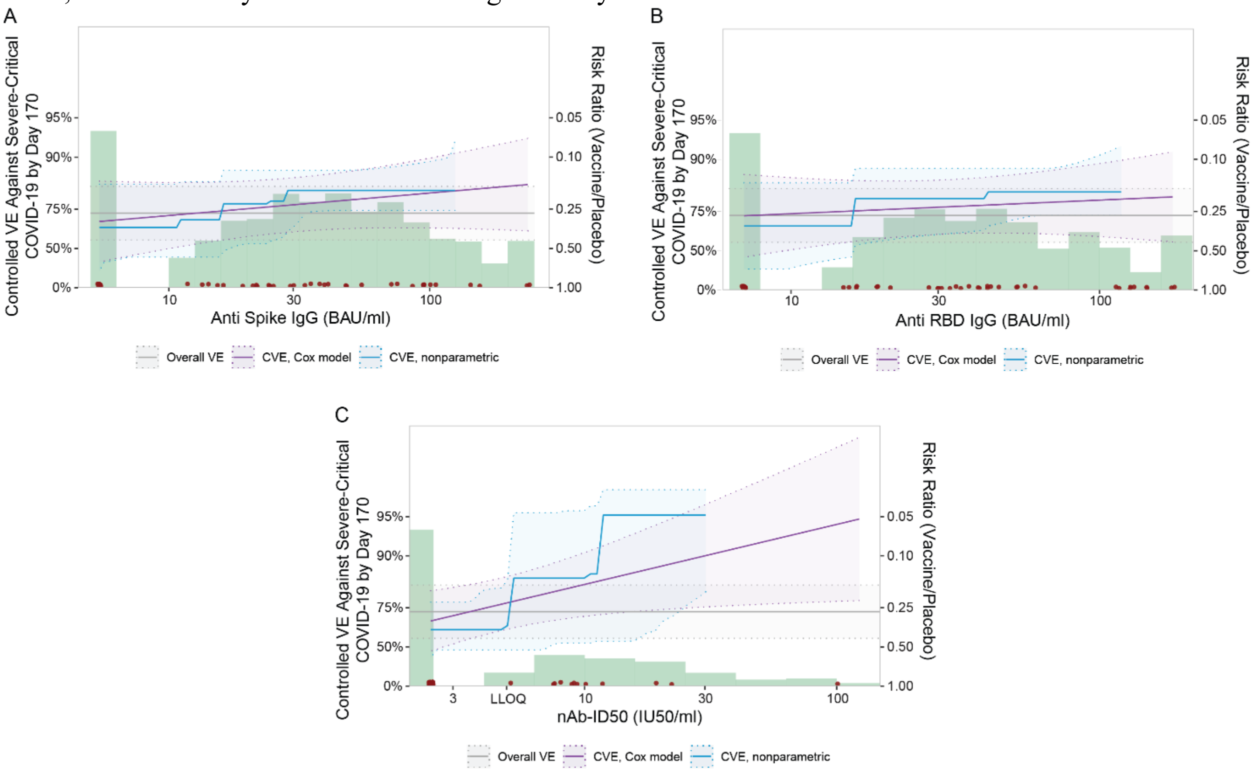

Supplementary Fig. 34. Vaccine efficacy against moderate COVID-19 by D29 antibody marker level. Analyses were performed in baseline SARS-CoV-2 seronegative per-protocol vaccine recipients. Estimates were obtained using (solid purple line) a Cox proportional hazards implementation of Gilbert et al.[1] or (solid blue line) a nonparametric implementation of Gilbert et al.[1] Each point on the curve represents the vaccine efficacy at the given D29 antibody marker level: **(A)** anti-Spike IgG concentration, **(B)** anti-RBD IgG concentration, **(C)** neutralizing antibody titer (nAb-ID50). The dotted lines indicate bootstrap pointwise 95% CIs. The green histograms are estimates of the frequency distribution of each D29 antibody marker, with the maroon dots representing the marker levels of the individual cases. Analyses adjusted for baseline behavioral risk score and geographic region. Nonparametric curves are plotted over the Spike IgG concentration range from negative response to the 90th percentile (125 BAU/ml), RBD IgG concentration range from negative response to the 90th percentile (118 BAU/ml), and nAb-ID50 titer range from unquantifiable to the 90th percentile (30.2 IU50/ml). Cox curves are plotted over the Spike IgG concentration range from negative response to the 97.5th percentile (238 BAU/ml), RBD IgG concentration range from negative response to the 97.5th percentile (173 BAU/ml), and nAb-ID50 titer range from unquantifiable to the 97.5th percentile (123 IU50/ml). Positivity cutoffs: 10.8424 BAU/ml for Spike and 14.0858 BAU/ml for RBD. nAb-ID50 LLOQ=4.8975 IU50/ml. BAU, binding antibody units; CI, confidence interval; IU, international units; LLOQ, lower limit of quantitation; nAb-ID50, 50% inhibitory dilution neutralizing antibody.

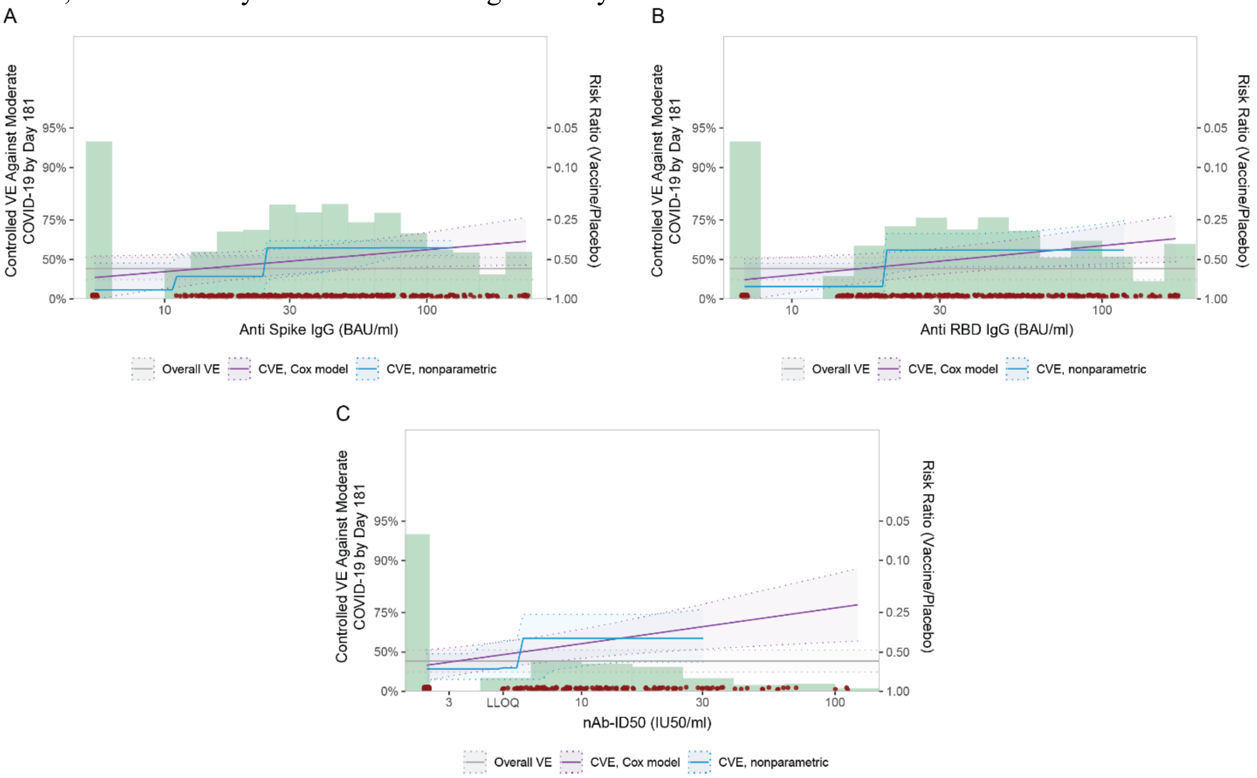

Supplementary Fig. 35. Vaccine efficacy against moderate to severe-critical COVID-19 by D29 antibody marker level. Analyses were performed in baseline SARS-CoV-2 seronegative per-protocol vaccine recipients. Estimates were obtained using (solid purple line) a Cox proportional hazards implementation of Gilbert et al.[1] or (solid blue line) a nonparametric implementation of Gilbert et al.[1] Each point on the curve represents the vaccine efficacy at the given D29 antibody marker level: **(A)** anti-Spike IgG concentration, **(B)** anti-RBD IgG concentration, **(C)** neutralizing antibody titer (nAb-ID50). The dotted lines indicate bootstrap pointwise 95% CIs. The green histograms are estimates of the frequency distribution of each D29 antibody marker, with the maroon dots representing the marker levels of the individual cases. Analyses adjusted for baseline behavioral risk score and geographic region. Nonparametric curves are plotted over the Spike IgG concentration range from negative response to the 90th percentile (125 BAU/ml), RBD IgG concentration range from negative response to the 90th percentile (118 BAU/ml), and nAb-ID50 titer range from unquantifiable to the 90th percentile (30.2 IU50/ml). Cox curves are plotted over the Spike IgG concentration range from negative response to the 97.5th percentile (238 BAU/ml), RBD IgG concentration range from negative response to the 97.5th percentile (173 BAU/ml), and nAb-ID50 titer range from unquantifiable to the 97.5th percentile (123 IU50/ml). Positivity cutoffs: 10.8424 BAU/ml for Spike and 14.0858 BAU/ml for RBD. nAb-ID50 LLOQ=4.8975 IU50/ml. BAU, binding antibody units; CI, confidence interval; IU, international units; LLOQ, lower limit of quantitation; nAb-ID50, 50% inhibitory dilution neutralizing antibody.

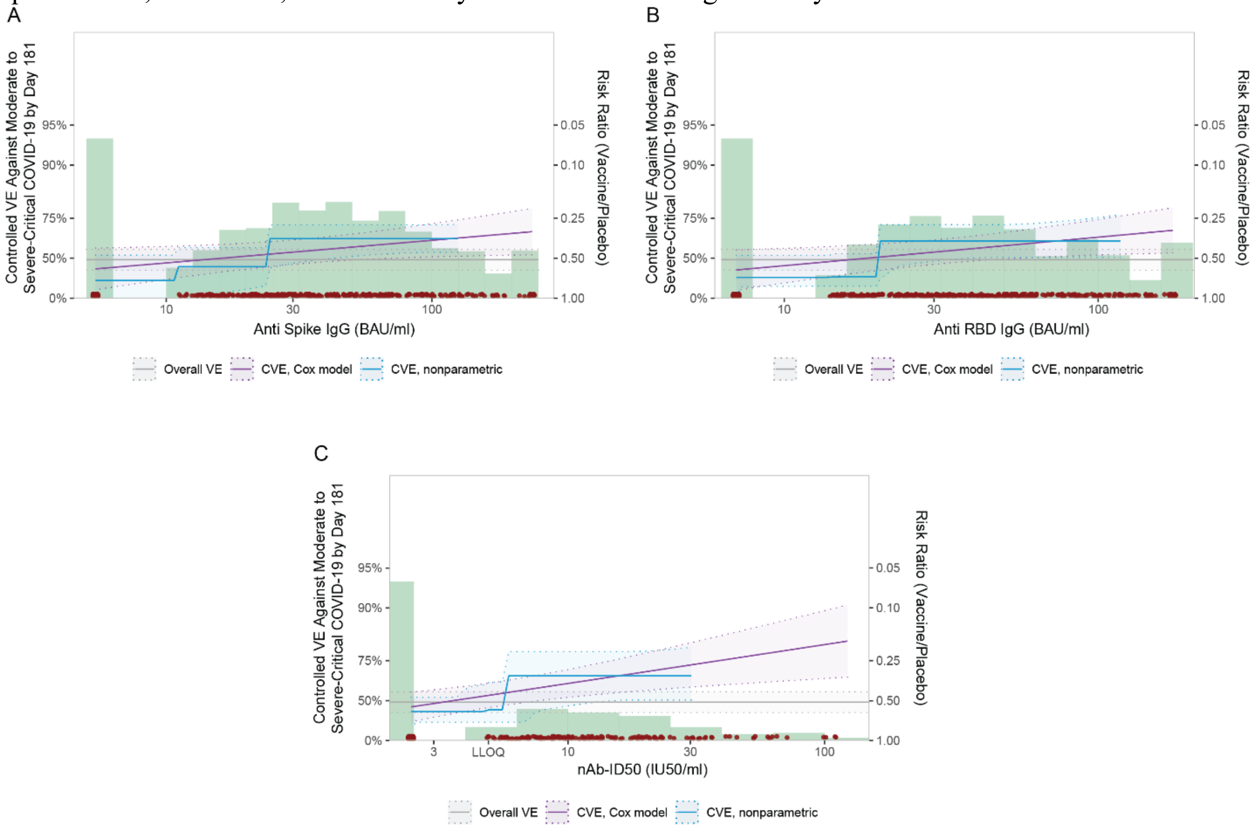

Supplementary Fig. 36. Vaccine efficacy against severe-critical COVID-19 by D29 antibody marker level in participants in Latin America. Analyses were performed in baseline SARS-CoV-2 seronegative per-protocol vaccine recipients in Latin America. Estimates were obtained using (solid purple line) a Cox proportional hazards implementation of Gilbert et al.[1] or (solid blue line) a nonparametric implementation of Gilbert et al.[1] Each point on the curve represents the vaccine efficacy at the given D29 antibody marker level: **(A)** anti-Spike IgG concentration, **(B)** anti-RBD IgG concentration, **(C)** neutralizing antibody titer (nAb-ID50). The dotted lines indicate bootstrap pointwise 95% CIs. The green histograms are estimates of the frequency distribution of each D29 antibody marker, with the maroon dots representing the marker levels of the individual cases. Analyses adjusted for baseline behavioral risk score. Nonparametric curves are plotted over the Spike IgG concentration range from negative response to the 90th percentile (121 BAU/ml), RBD IgG concentration range from negative response to the 90th percentile (114 BAU/ml), and nAb-ID50 titer range from unquantifiable to the 90th percentile (28.5 IU50/ml). Cox curves are plotted over the Spike IgG concentration range from negative response to the 97.5th percentile (238 BAU/ml), RBD IgG concentration range from negative response to the 97.5th percentile (173 BAU/ml), and nAb-ID50 titer range from unquantifiable to the 97.5th percentile (97.2 IU50/ml). Positivity cutoffs: 10.8424 BAU/ml for Spike and 14.0858 BAU/ml for RBD. nAb-ID50 LLOQ=4.8975 IU50/ml. BAU, binding antibody units; CI, confidence interval; IU, international units; LLOQ, lower limit of quantitation; nAb-ID50, 50% inhibitory dilution neutralizing antibody.

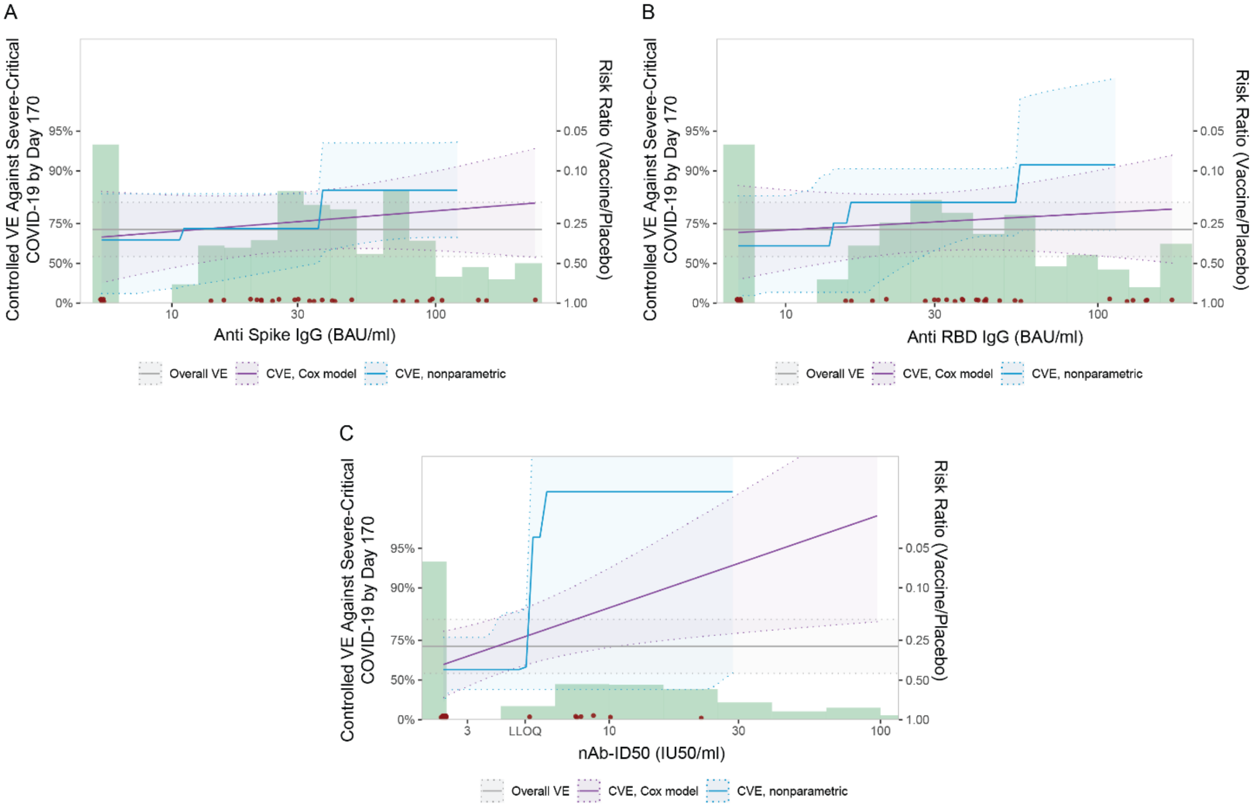

Supplementary Fig. 37. Vaccine efficacy against moderate COVID-19 by D29 antibody marker level in participants in Latin America. Analyses were performed in baseline SARS-CoV-2 seronegative per-protocol vaccine recipients in Latin America. Estimates were obtained using (solid purple line) a Cox proportional hazards implementation of Gilbert et al.[1] or (solid blue line) a nonparametric implementation of Gilbert et al.[1] Each point on the curve represents the vaccine efficacy at the given D29 antibody marker level: **(A)** anti-Spike IgG concentration, **(B)** anti-RBD IgG concentration, **(C)** neutralizing antibody titer (nAb-ID50). The dotted lines indicate bootstrap pointwise 95% CIs. The green histograms are estimates of the frequency distribution of each D29 antibody marker, with the maroon dots representing the marker levels of the individual cases. Analyses adjusted for baseline behavioral risk score. Nonparametric curves are plotted over the Spike IgG concentration range from negative response to the 90th percentile (121 BAU/ml), RBD IgG concentration range from negative response to the 90th percentile (114 BAU/ml), and nAb-ID50 titer range from unquantifiable to the 90th percentile (28.5 IU50/ml). Cox curves are plotted over the Spike IgG concentration range from negative response to the 97.5th percentile (238 BAU/ml), RBD IgG concentration range from negative response to the 97.5th percentile (173 BAU/ml), and nAb-ID50 titer range from unquantifiable to the 97.5th percentile (97.2 IU50/ml). Positivity cutoffs: 10.8424 BAU/ml for Spike and 14.0858 BAU/ml for RBD. nAb-ID50 LLOQ=4.8975 IU50/ml. BAU, binding antibody units; CI, confidence interval; IU, international units; LLOQ, lower limit of quantitation; nAb-ID50, 50% inhibitory dilution neutralizing antibody.

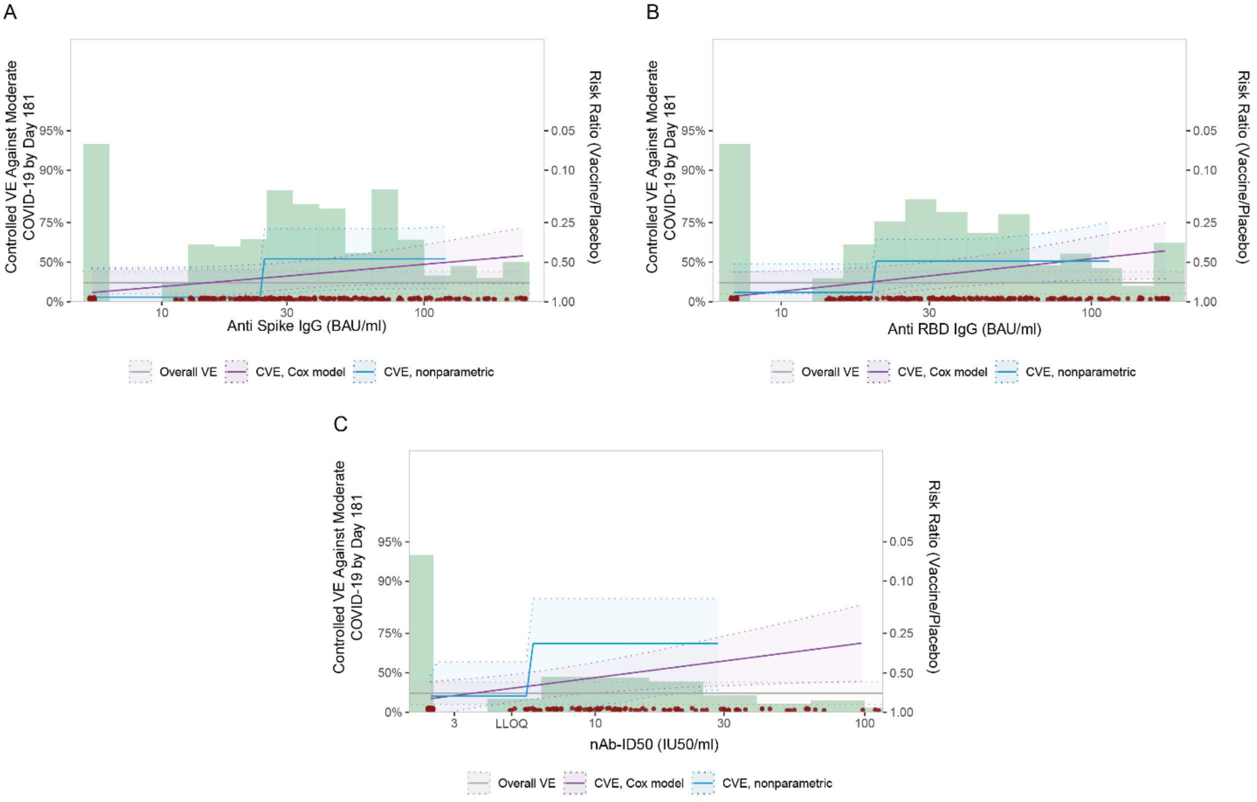

Supplementary Fig. 38. Vaccine efficacy against moderate to severe-critical COVID-19 by D29 antibody marker level in participants in Latin America. Analyses were performed in baseline SARS-CoV-2 seronegative per-protocol vaccine recipients in Latin America. Estimates were obtained using (solid purple line) a Cox proportional hazards implementation of Gilbert et al.[1] or (solid blue line) a nonparametric implementation of Gilbert et al.[1] Each point on the curve represents the vaccine efficacy at the given D29 antibody marker level: **(A)** anti-Spike IgG concentration, **(B)** anti-RBD IgG concentration, **(C)** neutralizing antibody titer (nAb-ID50). The dotted lines indicate bootstrap pointwise 95% CIs. The green histograms are estimates of the frequency distribution of each D29 antibody marker, with the maroon dots representing the marker levels of the individual cases. Analyses adjusted for baseline behavioral risk score. Nonparametric curves are plotted over the Spike IgG concentration range from negative response to the 90th percentile (121 BAU/ml), RBD IgG concentration range from negative response to the 90th percentile (114 BAU/ml), and nAb-ID50 titer range from unquantifiable to the 90th percentile (28.5 IU50/ml). Cox curves are plotted over the Spike IgG concentration range from negative response to the 97.5th percentile (238 BAU/ml), RBD IgG concentration range from negative response to the 97.5th percentile (173 BAU/ml), and nAb-ID50 titer range from unquantifiable to the 97.5th percentile (97.2 IU50/ml). Positivity cutoffs: 10.8424 BAU/ml for Spike and 14.0858 BAU/ml for RBD. nAb-ID50 LLOQ=4.8975 IU50/ml. BAU, binding antibody units; CI, confidence interval; IU, international units; LLOQ, lower limit of quantitation; nAb-ID50, 50% inhibitory dilution neutralizing antibody.

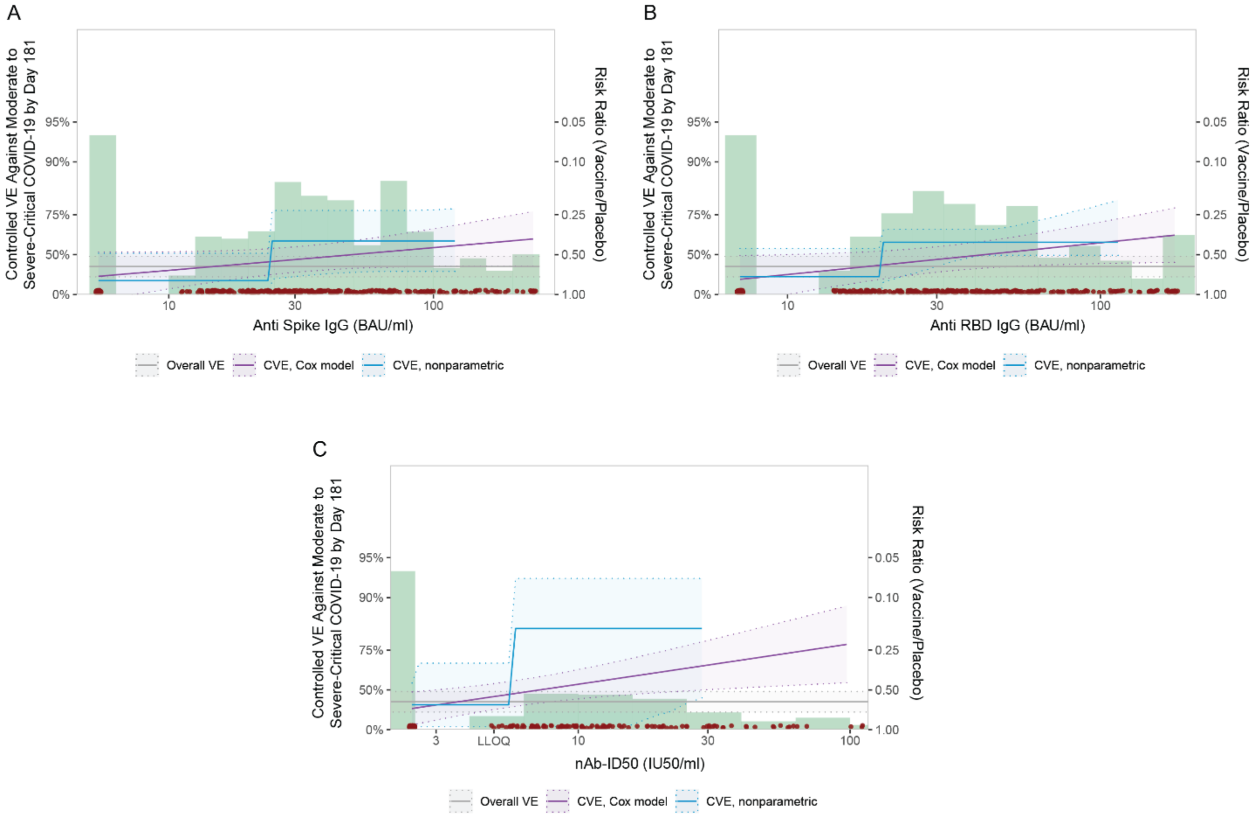

Supplementary Fig. 39. Vaccine efficacy against moderate to severe-critical COVID-19 by D29 antibody marker level in participants in South Africa. Analyses were performed in baseline SARS-CoV-2 seronegative per-protocol vaccine recipients in South Africa. Estimates were obtained using (solid purple line) a Cox proportional hazards implementation of Gilbert et al.[1] or (solid blue line) a nonparametric implementation of Gilbert et al.[1] Each point on the curve represents the vaccine efficacy at the given D29 antibody marker level: **(A)** anti-Spike IgG concentration, **(B)** anti-RBD IgG concentration, **(C)** neutralizing antibody titer (nAb-ID50). The dotted lines indicate bosotstrap pointwise 95% CIs. The green histograms are estimates of the frequency distribution of each D29 antibody marker, with the maroon dots representing the marker levels of the individual cases. Analyses adjusted for baseline behavioral risk score. Nonparametric curves are plotted over the Spike IgG concentration range from negative response to the 90th percentile (125 BAU/ml), RBD IgG concentration range from negative response to the 90th percentile (125 BAU/ml), and nAb-ID50 titer range from unquantifiable to the 90th percentile (30.2 IU50/ml). Cox curves are plotted over the Spike IgG concentration range from negative response to the 97.5th percentile (238 BAU/ml), RBD IgG concentration range from negative response to the 97.5th percentile (173 BAU/ml), and nAb-ID50 titer range from unquantifiable to the 97.5th percentile (91.7 IU50/ml). Positivity cutoffs: 10.8424 BAU/ml for Spike and 14.0858 BAU/ml for RBD. nAb-ID50 LLOQ=4.8975 IU50/ml. BAU, binding antibody units; CI, confidence interval; IU, international units; LLOQ, lower limit of quantitation; nAb-ID50, 50% inhibitory dilution neutralizing antibody.

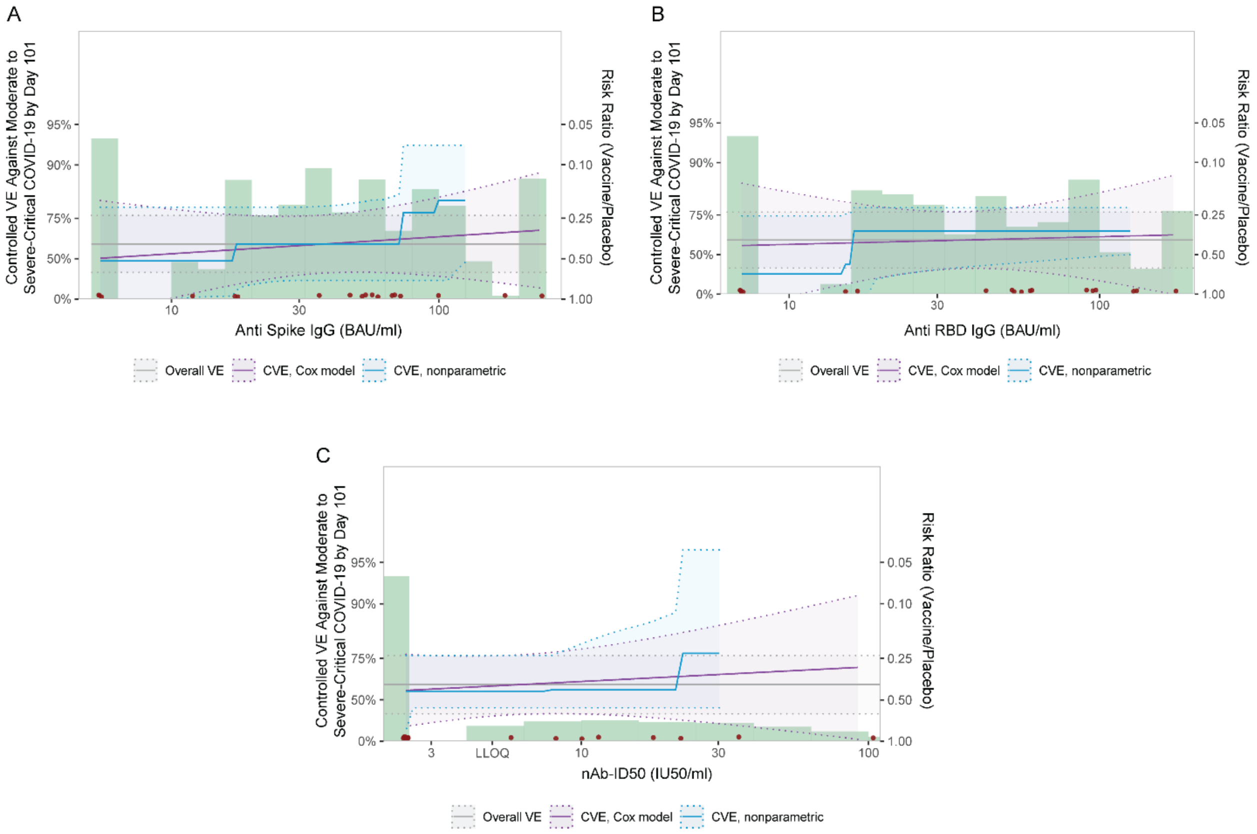

Supplementary Fig. 40. Vaccine efficacy against moderate to severe-critical COVID-19 by D29 antibody marker level in participants in the United States. Analyses were performed in baseline SARS-CoV-2 seronegative per-protocol vaccine recipients in the United States. Estimates were obtained using (solid purple line) a Cox proportional hazards implementation of Gilbert et al.[1] or (solid blue line) a nonparametric implementation of Gilbert et al.[1] Each point on the curve represents the vaccine efficacy at the given D29 antibody marker level: **(A)** anti-Spike IgG concentration, **(B)** anti-RBD IgG concentration, **(C)** neutralizing antibody titer (nAb-ID50). The dotted lines indicate bootstrap pointwise 95% CIs. The green histograms are estimates of the frequency distribution of each D29 antibody marker, with the maroon dots representing the marker levels of the individual cases. Analyses adjusted for baseline behavioral risk score. Nonparametric curves are plotted over the following ranges: Spike IgG, negative response to the 90% quantile (131 BAU/ml); RBD IgG, negative response to the 90% quantile (117 BAU/ml); nAb-ID50, unquantifiable titer to the 90% quantile (28.9 IU50/ml). Cox model curves are plotted over the following ranges: Spike IgG, negative response to the 97.5% quantile (209 BAU/ml); RBD IgG, negative response to the 97.5% quantile (201 BAU/ml); nAb-ID50, unquantifiable titer to the 97.5% quantile (157 IU50/ml). Positivity cutoffs: 10.8424 BAU/ml for Spike and 14.0858 BAU/ml for RBD. nAb-ID50 LLOQ=4.8975 IU50/ml. BAU, binding antibody units; CI, confidence interval; CVE, controlled vaccine efficacy; IU, international units; nAb-ID50, 50% inhibitory dilution neutralizing antibody.

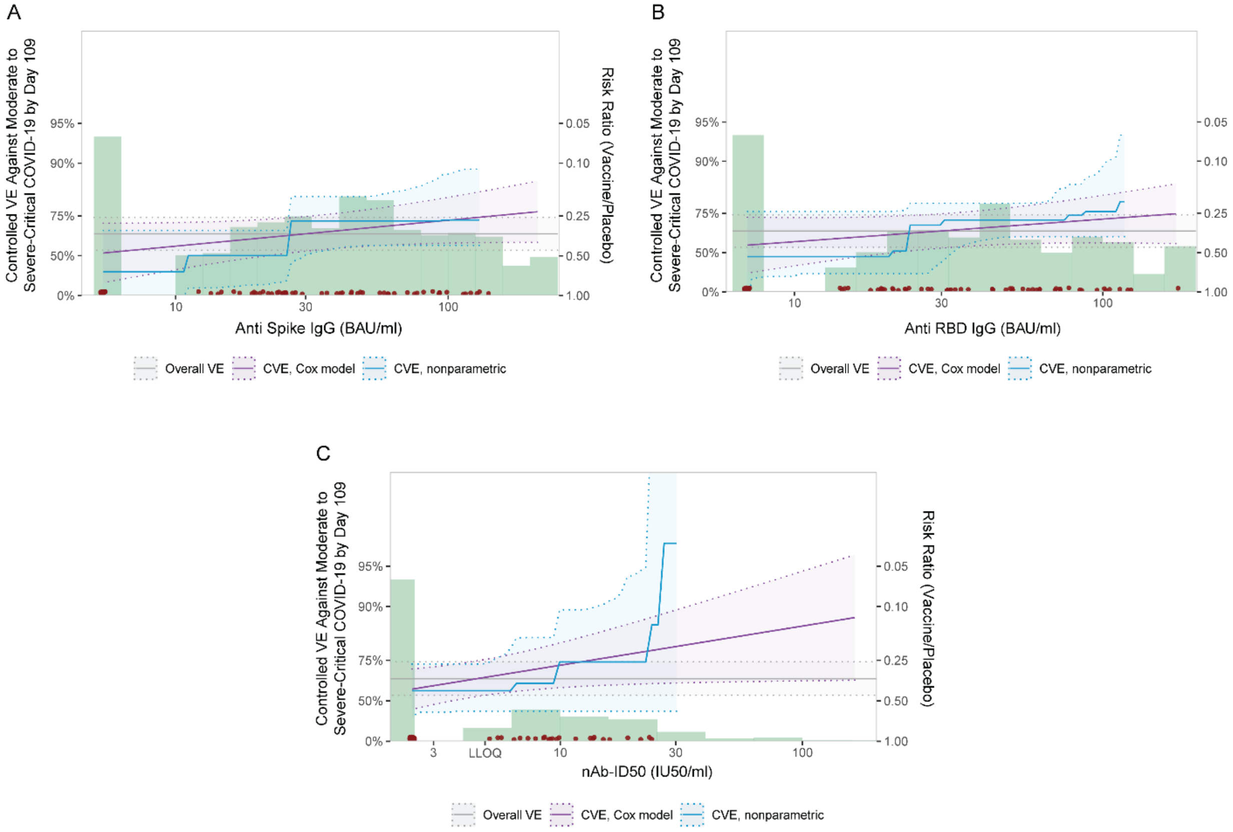

Supplementary Fig. 41. Vaccine efficacy with sensitivity analysis against severe-critical COVID-19 by D29 antibody marker level. Analyses were performed in baseline SARS-CoV-2 seronegative per-protocol vaccine recipients. Estimates were obtained using the method of Gilbert et al.[1] Each point on the curve represents the vaccine efficacy at the given D29 antibody marker level: **(A)** anti-Spike IgG concentration, **(B)** anti-RBD IgG concentration, **(C)** neutralizing antibody titer (nAb-ID50). The pink solid line is point estimates assuming no unmeasured confounding; the dashed lines are bootstrap pointwise 95% CIs. The red solid line is point estimates assuming unmeasured confounding in a sensitivity analysis (dashed lines are bootstrap pointwise 95% CIs); see the Statistical Analysis Plan for details. The upper boundary of the green shaded area is the estimate of the reverse cumulative distribution function of the marker in baseline SARS-CoV-2 negative per-protocol vaccine recipients. Analyses adjusted for baseline behavioral risk score and geographic region. Curves are plotted over the range from the 2.5th percentile to the 97.5th percentile of each marker: Spike IgG, negative response to 238 BAU/ml; RBD IgG, negative response to173 BAU/ml; nAb-ID50, unquantifiable to 130 IU50/ml. BAU, binding antibody units; CI, confidence interval; IU, international units; nAb-ID50, 50% inhibitory dilution neutralizing antibody. OS, positivity cutoff: 10.8424 BAU/ml for Spike and 14.0858 BAU/ml for RBD. LLOQ, lower limit of quantitation = 4.8975 IU50/ml for nAb-ID50.

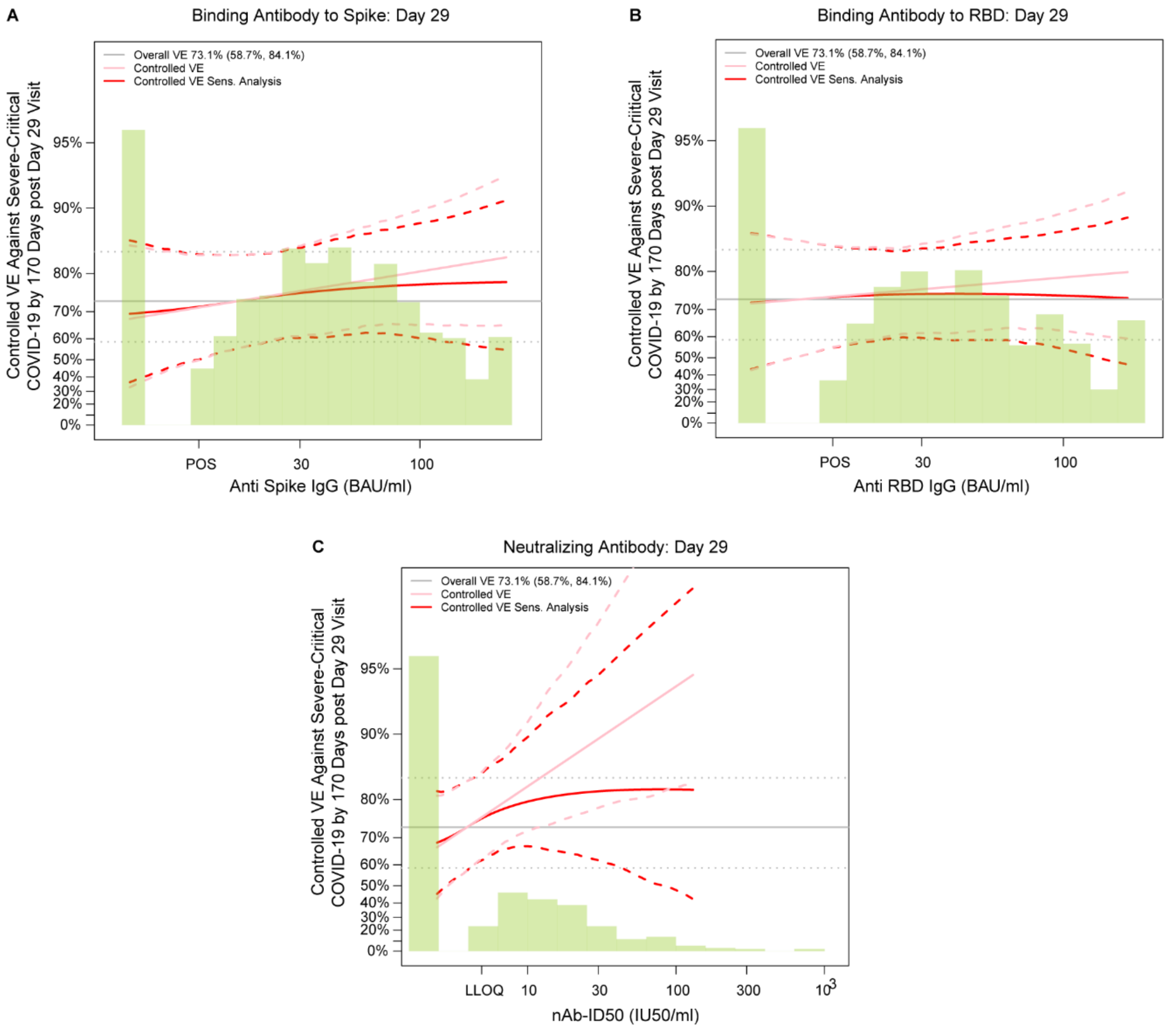

Supplementary Fig. 42. Vaccine efficacy with sensitivity analysis against moderate COVID-19 by D29 antibody marker level. Analyses were performed in baseline SARS-CoV-2 seronegative per-protocol vaccine recipients. Estimates were obtained using the method of Gilbert et al.[1] Each point on the curve represents the vaccine efficacy at the given D29 antibody marker level: **(A)** anti-Spike IgG concentration, **(B)** anti-RBD IgG concentration, **(C)** neutralizing antibody titer (nAb-ID50). The pink solid line is point estimates assuming no unmeasured confounding; the dashed lines are bootstrap pointwise 95% CIs. The red solid line is point estimates assuming unmeasured confounding in a sensitivity analysis (dashed lines are bootstrap pointwise 95% CIs); see the Statistical Analysis Plan for details. The upper boundary of the green shaded area is the estimate of the reverse cumulative distribution function of the marker in baseline SARS-CoV-2 negative per-protocol vaccine recipients. Analyses adjusted for baseline behavioral risk score and geographic region. Curves are plotted over the range from the 2.5th percentile to the 97.5th percentile of each marker: Spike IgG, negative response to 238 BAU/ml; RBD IgG, negative response to173 BAU/ml; nAb-ID50, unquantifiable to 130 IU50/ml. BAU, binding antibody units; CI, confidence interval; IU, international units; nAb-ID50, 50% inhibitory dilution neutralizing antibody. POS, positivity cutoff: 10.8424 BAU/ml for Spike and 14.0858 BAU/ml for RBD. LLOQ, lower limit of quantitation = 4.8975 IU50/ml for nAb-ID50.

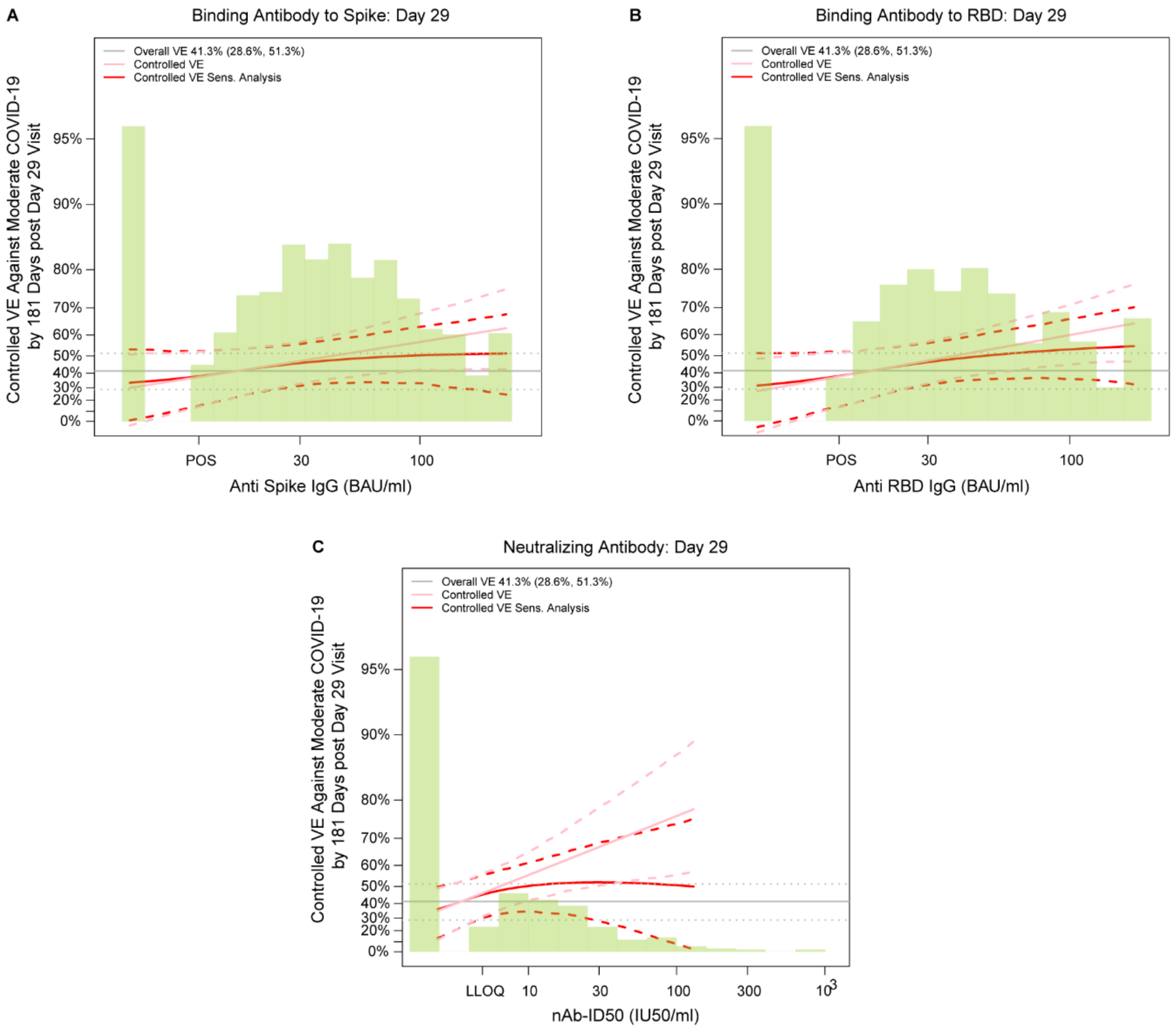

Supplementary Fig. 43. Vaccine efficacy with sensitivity analysis against moderate to severe-critical COVID-19 by D29 antibody marker level. Analyses were performed in baseline SARS-CoV-2 seronegative per-protocol vaccine recipients. Estimates were obtained using the method of Gilbert et al.[1] Each point on the curve represents the vaccine efficacy at the given D29 antibody marker level: **(A)** anti-Spike IgG concentration, **(B)** anti-RBD IgG concentration, **(C)** neutralizing antibody titer (nAb-ID50). The pink solid line is point estimates assuming no unmeasured confounding; the dashed lines are bootstrap pointwise 95% CIs. The red solid line is point estimates assuming unmeasured confounding in a sensitivity analysis (dashed lines are bootstrap pointwise 95% CIs); see the Statistical Analysis Plan for details. The upper boundary of the green shaded area is the estimate of the reverse cumulative distribution function of the marker in baseline SARS-CoV-2 negative per-protocol vaccine recipients. Analyses adjusted for baseline behavioral risk score and geographic region. Curves are plotted over the range from the 2.5th percentile to the 97.5th percentile of each marker: Spike IgG, negative response to 238 BAU/ml; RBD IgG, negative response to 173 BAU/ml; nAb-ID50, unquantifiable to 130 IU50/ml. BAU, binding antibody units; CI, confidence interval; IU, international units; nAb-ID50, 50% inhibitory dilution neutralizing antibody. POS, positivity cutoff: 10.8424 BAU/ml for Spike and 14.0858 BAU/ml for RBD. LLOQ, lower limit of quantitation = 4.8975 IU50/ml for nAb-ID50.

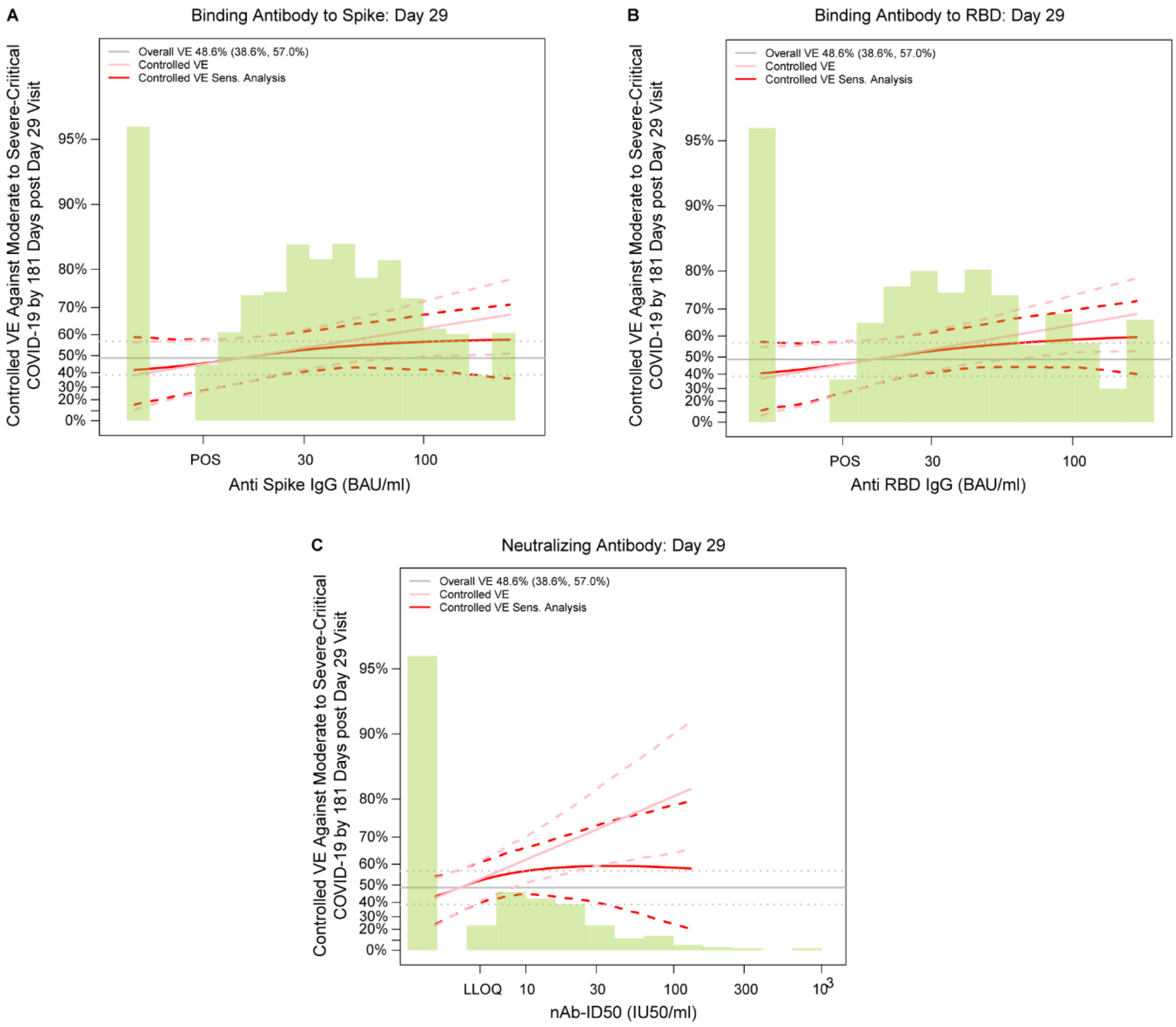

Supplementary Fig. 44. Vaccine efficacy with sensitivity analysis against severe-critical COVID-19 by D29 antibody marker level in participants in Latin America. Analyses were performed in baseline SARS-CoV-2 seronegative per-protocol vaccine recipients in Latin America. Estimates were obtained using the method of Gilbert et al.[1] Each point on the curve represents the vaccine efficacy at the given D29 antibody marker level: **(A)** anti-Spike IgG concentration, **(B)** anti-RBD IgG concentration, **(C)** neutralizing antibody titer (nAb-ID50). The pink solid line is point estimates assuming no unmeasured confounding; the dashed lines are bootstrap pointwise 95% CIs. The red solid line is point estimates assuming unmeasured confounding in a sensitivity analysis (dashed lines are bootstrap pointwise 95% CIs); see the Statistical Analysis Plan for details. The upper boundary of the green shaded area is the estimate of the reverse cumulative distribution function of the marker in baseline SARS-CoV-2 negative per-protocol vaccine recipients in Latin America. Analyses adjusted for baseline behavioral risk score. Curves are plotted over the range from the 2.5th percentile to the 97.5th percentile of each marker: Spike IgG, negative response to 238 BAU/ml; RBD IgG, negative response to 173 BAU/ml; nAb-ID50, unquantifiable to 109 IU50/ml. BAU, binding antibody units; CI, confidence interval; IU, international units; nAb-ID50, 50% inhibitory dilution neutralizing antibody. Positivity cutoffs: 10.8424 BAU/ml for Spike and 14.0858 BAU/ml for RBD. LLOQ, lower limit of quantitation = 4.8975 IU50/ml for nAb-ID50.

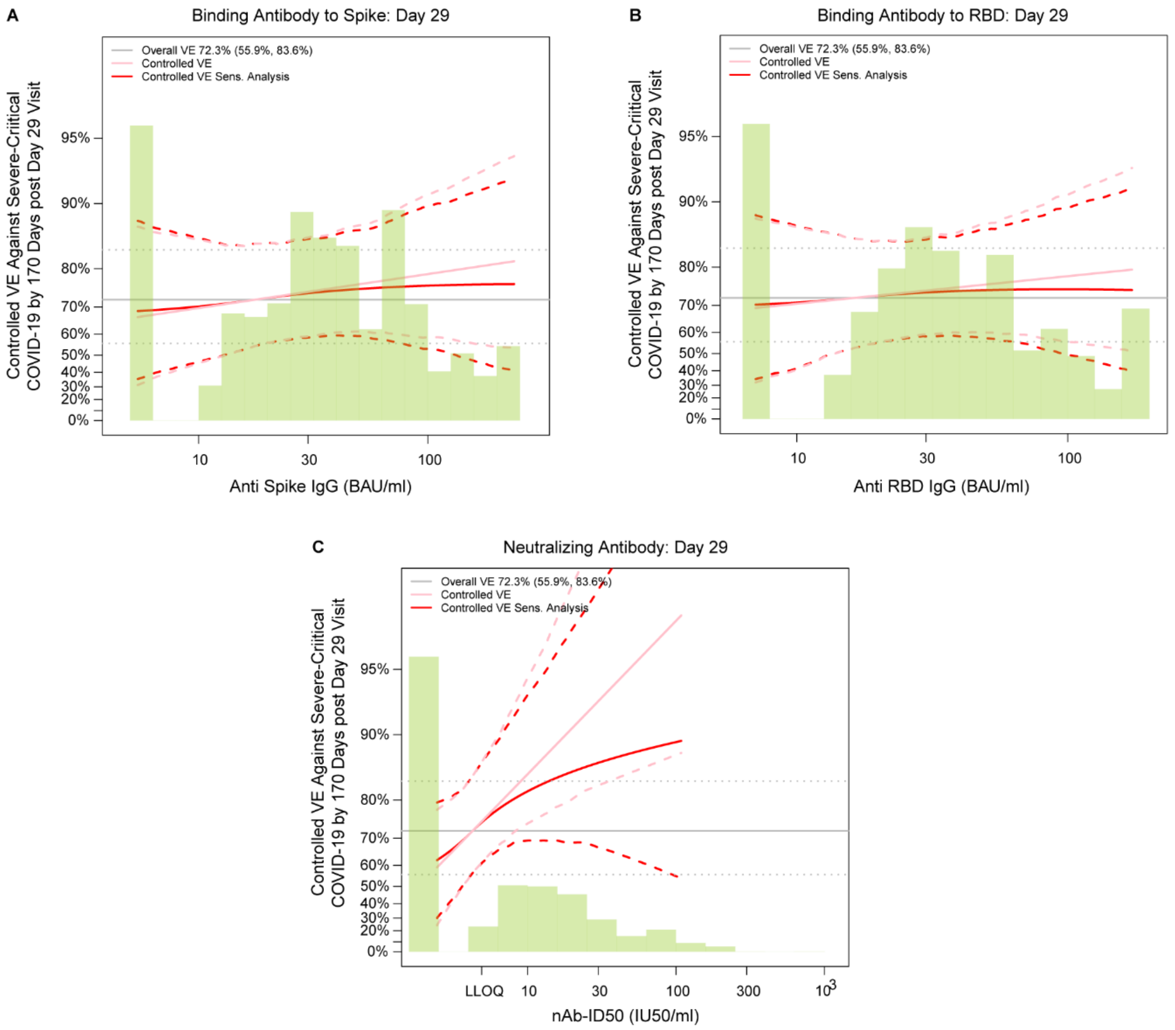

Supplementary Fig. 45. Vaccine efficacy with sensitivity analysis against moderate COVID-19 by D29 antibody marker level in participants in Latin America. Analyses were performed in baseline SARS-CoV-2 seronegative per-protocol vaccine recipients in Latin America. Estimates were obtained using the method of Gilbert et al.[1] Each point on the curve represents the vaccine efficacy at the given D29 antibody marker level: **(A)** anti-Spike IgG concentration, **(B)** anti-RBD IgG concentration, **(C)** neutralizing antibody titer (nAb-ID50). The pink solid line is point estimates assuming no unmeasured confounding; the dashed lines are bootstrap pointwise 95% CIs. The red solid line is point estimates assuming unmeasured confounding in a sensitivity analysis (dashed lines are bootstrap pointwise 95% CIs); see the Statistical Analysis Plan for details. The upper boundary of the green shaded area is the estimate of the reverse cumulative distribution function of the marker in baseline SARS-CoV-2 negative per-protocol vaccine recipients in Latin America. Analyses adjusted for baseline behavioral risk score. Curves are plotted over the range from the 2.5th percentile to the 97.5th percentile of each marker: Spike IgG, negative response to 238 BAU/ml; RBD IgG, negative response to 173 BAU/ml; nAb-ID50, unquantifiable to 109 IU50/ml. BAU, binding antibody units; CI, confidence interval; IU, international units; nAb-ID50, 50% inhibitory dilution neutralizing antibody. Positivity cutoffs: 10.8424 BAU/ml for Spike and 14.0858 BAU/ml for RBD. LLOQ, lower limit of quantitation = 4.8975 IU50/ml for nAb-ID50.

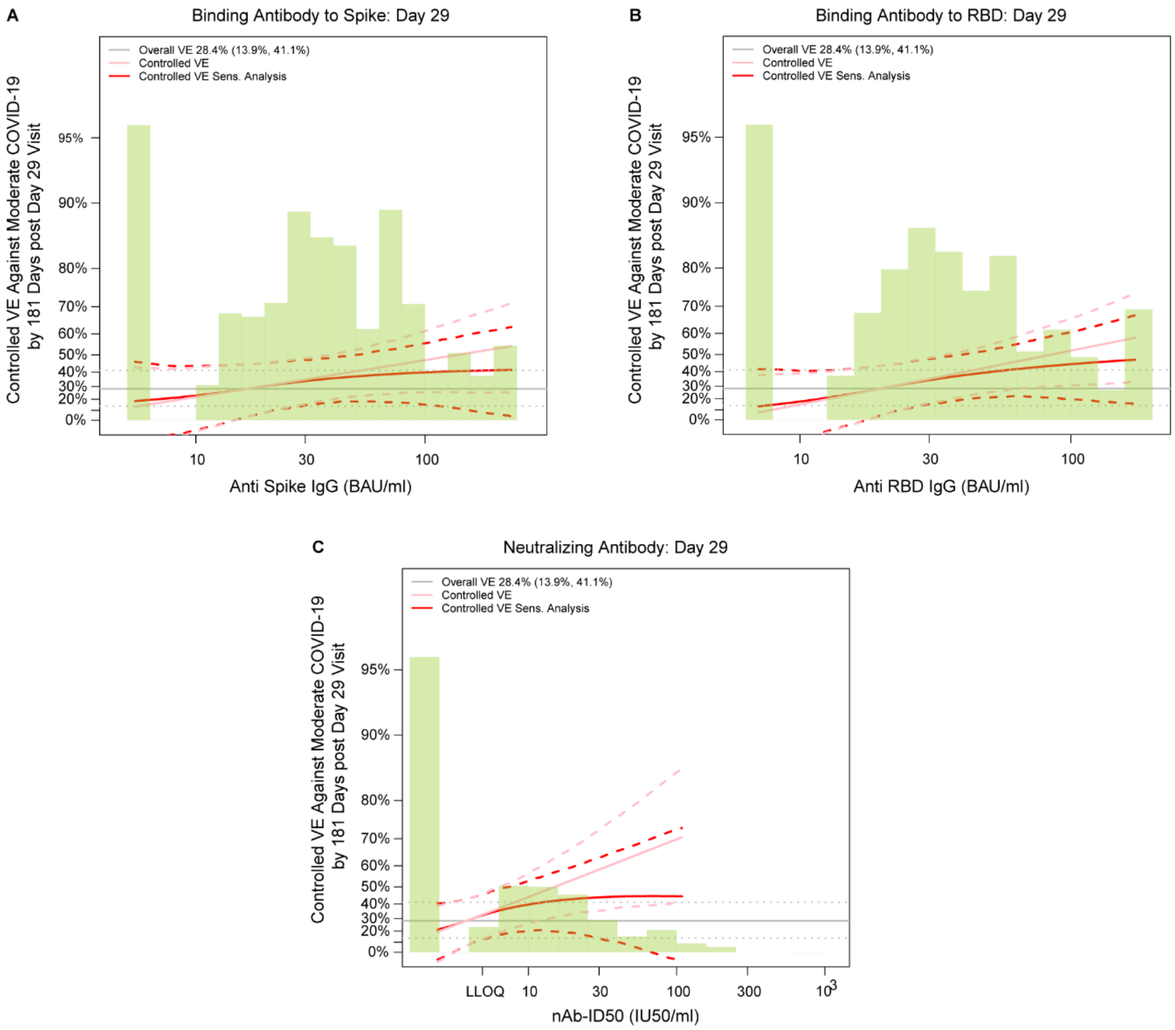

Supplementary Fig. 46. Vaccine efficacy with sensitivity analysis against moderate to severe-critical COVID-19 by D29 antibody marker level in participants in Latin America. Analyses were performed in baseline SARS-CoV-2 seronegative per-protocol vaccine recipients in Latin America. Estimates were obtained using the method of Gilbert et al.[1] Each point on the curve represents the vaccine efficacy at the given D29 antibody marker level: **(A)** anti-Spike IgG concentration, **(B)** anti-RBD IgG concentration, **(C)** neutralizing antibody titer (nAb-ID50). The pink solid line is point estimates assuming no unmeasured confounding; the dashed lines are bootstrap pointwise 95% CIs. The red solid line is point estimates assuming unmeasured confounding in a sensitivity analysis (dashed lines are bootstrap pointwise 95% CIs); see the Statistical Analysis Plan for details. The upper boundary of the green shaded area is the estimate of the reverse cumulative distribution function of the marker in baseline SARS-CoV-2 negative per-protocol vaccine recipients in Latin America. Analyses adjusted for baseline behavioral risk score. Curves are plotted over the range from the 2.5th percentile to the 97.5th percentile of each marker: Spike IgG, negative response to 238 BAU/ml; RBD IgG, negative response to 173 BAU/ml; nAb-ID50, unquantifiable to 109 IU50/ml. BAU, binding antibody units; CI, confidence interval; IU, international units; nAb-ID50, 50% inhibitory dilution neutralizing antibody. Positivity cutoffs: 10.8424 BAU/ml for Spike and 14.0858 BAU/ml for RBD. LLOQ, lower limit of quantitation = 4.8975 IU50/ml for nAb-ID50.

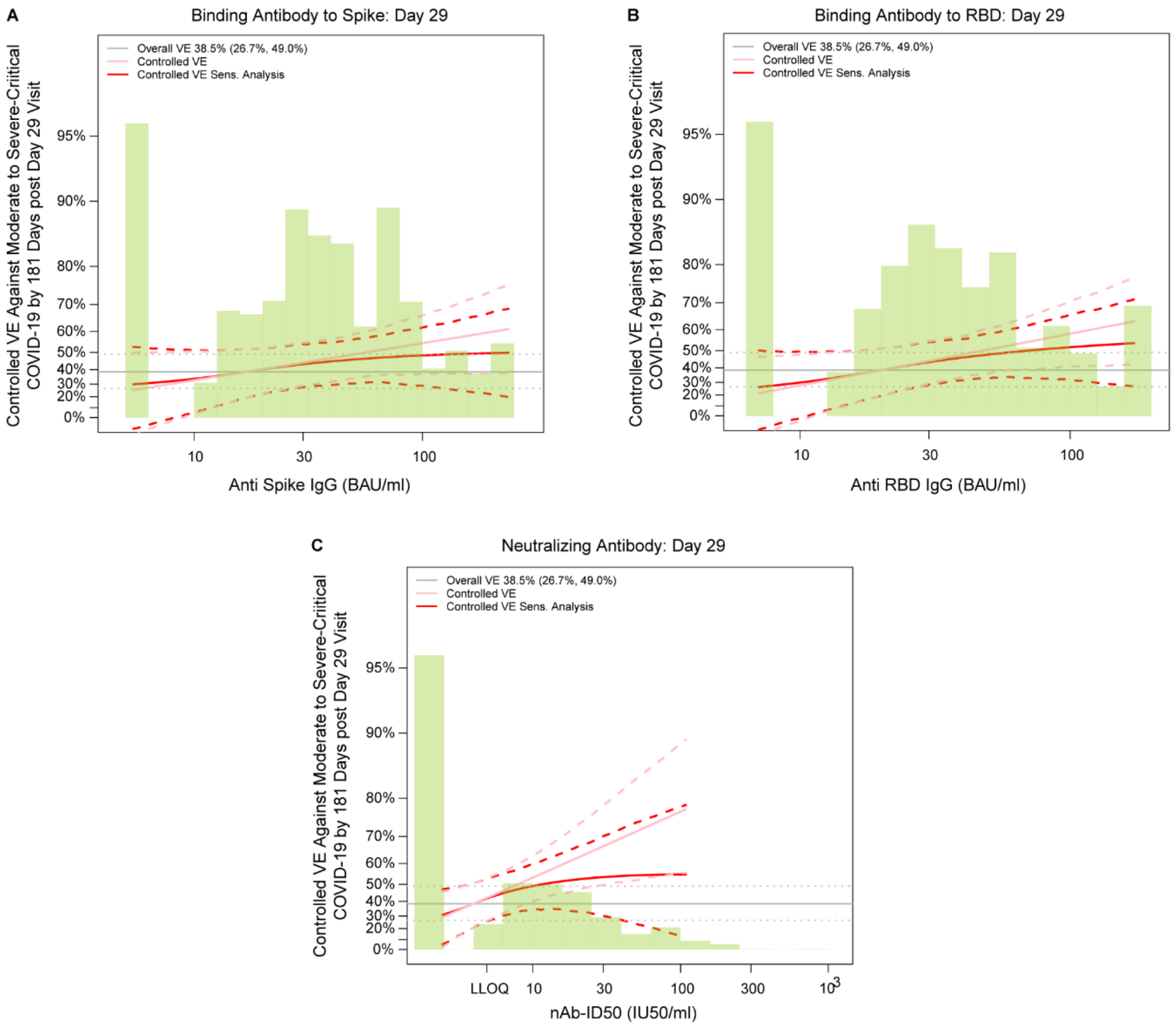

Supplementary Fig. 47. Vaccine efficacy with sensitivity analysis against moderate to severe-critical COVID-19 by D29 antibody marker level in participants in South Africa. Analyses were performed in baseline SARS-CoV-2 seronegative per-protocol vaccine recipients in South Africa. Estimates were obtained using the method of Gilbert et al.[1] Each point on the curve represents the vaccine efficacy at the given D29 antibody marker level: **(A)** anti-Spike IgG concentration, **(B)** anti-RBD IgG concentration, **(C)** neutralizing antibody titer (nAb-ID50). The pink solid line is point estimates assuming no unmeasured confounding; the dashed lines are bootstrap pointwise 95% CIs. The red solid line is point estimates assuming unmeasured confounding in a sensitivity analysis (dashed lines are bootstrap pointwise 95% CIs); see the Statistical Analysis Plan for details. The upper boundary of the green shaded area is the estimate of the reverse cumulative distribution function of the marker in baseline SARS-CoV-2 negative per-protocol vaccine recipients in South Africa. Analyses adjusted for baseline behavioral risk score. Curves are plotted over the range from the 2.5th percentile to the 97.5th percentile of each marker: Spike IgG, negative response to 238 BAU/ml; RBD IgG, negative response to 173 BAU/ml; nAb-ID50, unquantifiable to 82.9 IU50/ml. BAU, binding antibody units; CI, confidence interval; IU, international units; nAb-ID50, 50% inhibitory dilution neutralizing antibody. Positivity cutoffs: 10.8424 BAU/ml for Spike and 14.0858 BAU/ml for RBD. LLOQ, lower limit of quantitation = 4.8975 IU50/ml for nAb-ID50.

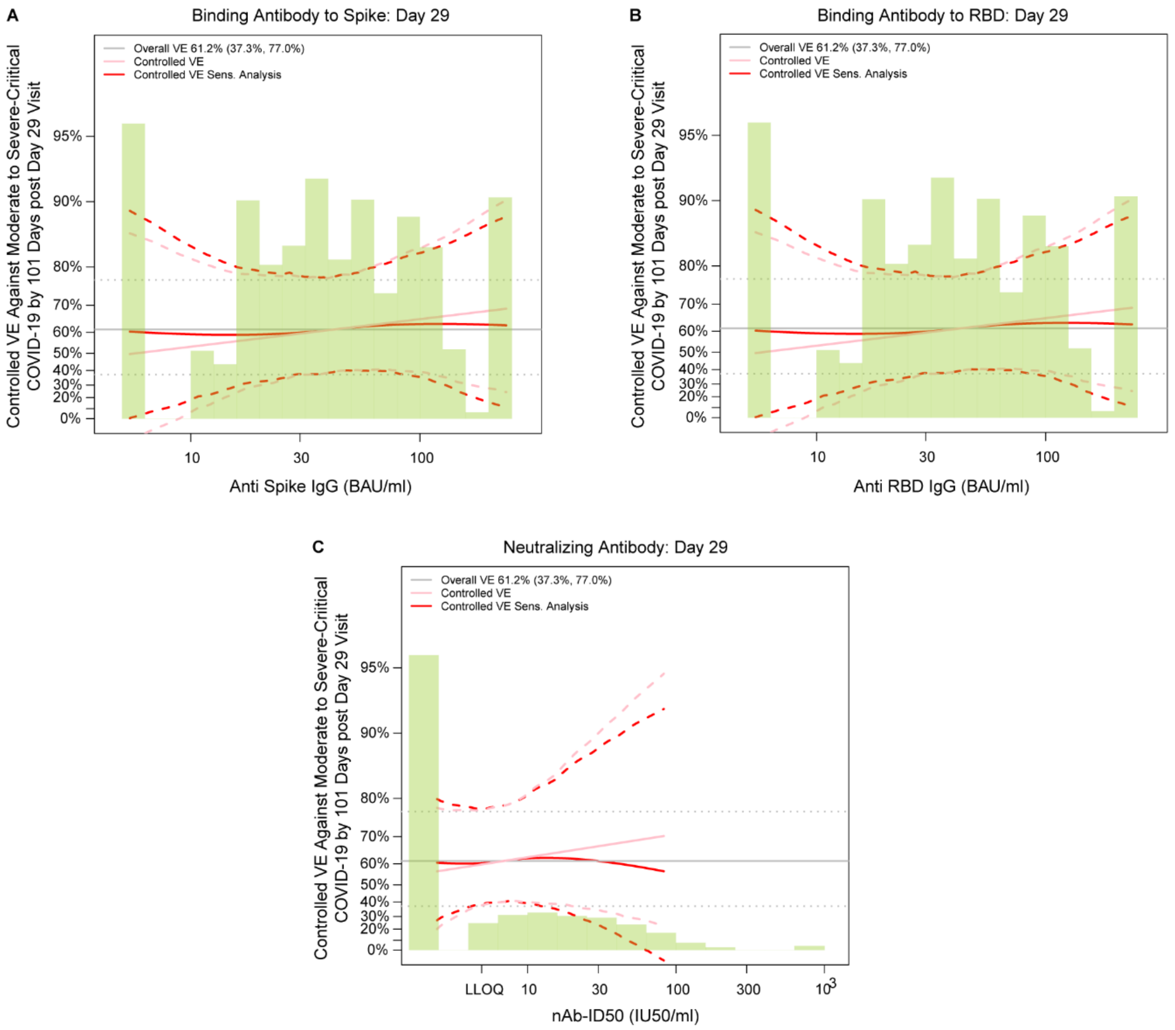

Supplementary Fig. 48. Vaccine efficacy with sensitivity analysis against moderate to severe-critical COVID-19 by D29 antibody marker level in participants in the United States. Analyses were performed in baseline SARS-CoV-2 seronegative per-protocol vaccine recipients in the United States. Estimates were obtained using the method of Gilbert et al.[1] Each point on the curve represents the vaccine efficacy at the given D29 antibody marker level: **(A)** anti-Spike IgG concentration, **(B)** anti-RBD IgG concentration, **(C)** neutralizing antibody titer (nAb-ID50). The pink solid line is point estimates assuming no unmeasured confounding; the dashed lines are bootstrap pointwise 95% CIs. The red solid line is point estimates assuming unmeasured confounding in a sensitivity analysis (dashed lines are bootstrap pointwise 95% CIs); see the Statistical Analysis Plan for details. The upper boundary of the green shaded area is the estimate of the reverse cumulative distribution function of the marker in baseline SARS-CoV-2 negative per-protocol vaccine recipients in the United States. Analyses adjusted for baseline behavioral risk score. Curves are plotted over the range from the 2.5th percentile to the 97.5th percentile of each marker: Spike IgG, negative response to 238 BAU/ml; RBD IgG, negative response to 173 BAU/ml; nAb-ID50, unquantifiable to 151 IU50/ml. BAU, binding antibody units; CI, confidence interval; IU, international units; nAb-ID50, 50% inhibitory dilution neutralizing antibody. Positivity cutoffs: 10.8424 BAU/ml for Spike and 14.0858 BAU/ml for RBD. LLOQ, lower limit of quantitation = 4.8975 IU50/ml for nAb-ID50.

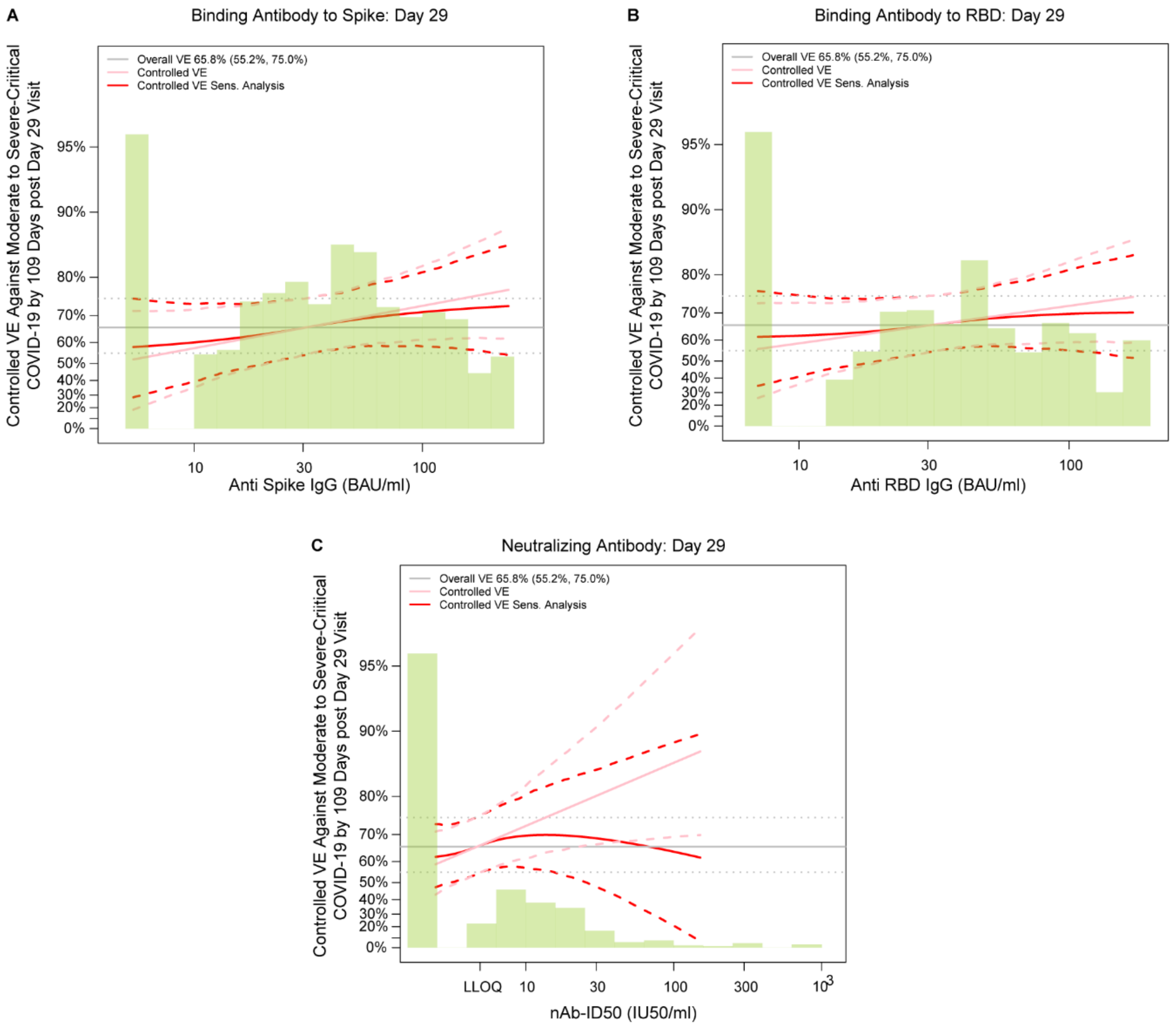

Supplementary Fig. 49. Stochastic interventional vaccine efficacy (SVE) against moderate to severe-critical COVID-19 under hypothetical shifts in D29 antibody level, for all geographic regions pooled. Analyses were performed in baseline SARS-CoV-2 seronegative per-protocol vaccine recipients. The y-axis plots estimated SVE, with 95% confidence intervals, for a vaccine that elicits hypothetical D29 geometric mean **(A)** anti-Spike IgG concentration, **(B)** anti-RBD IgG concentration, or **(C)** nAb-ID50 titer as indicated on the x-axis. SVE estimates were obtained using the method described in Hejazi et al.[2] The vertical red line corresponds to the geometric mean concentration or titer in the ENSEMBLE study population (baseline negative per-protocol vaccine recipients in the immunogenicity subcohort) and the horizontal red line corresponds to the estimated VE against moderate to severe-critical COVID-19 in ENSEMBLE (by 181 days post D29) at a shift of 0, i.e., the observed marker level. BAU, binding antibody units; IU, international units; LLOQ, lower limit of quantitation; nAb-ID50, 50% inhibitory dilution neutralizing antibody; Pos.Cutoff, positivity cutoff.

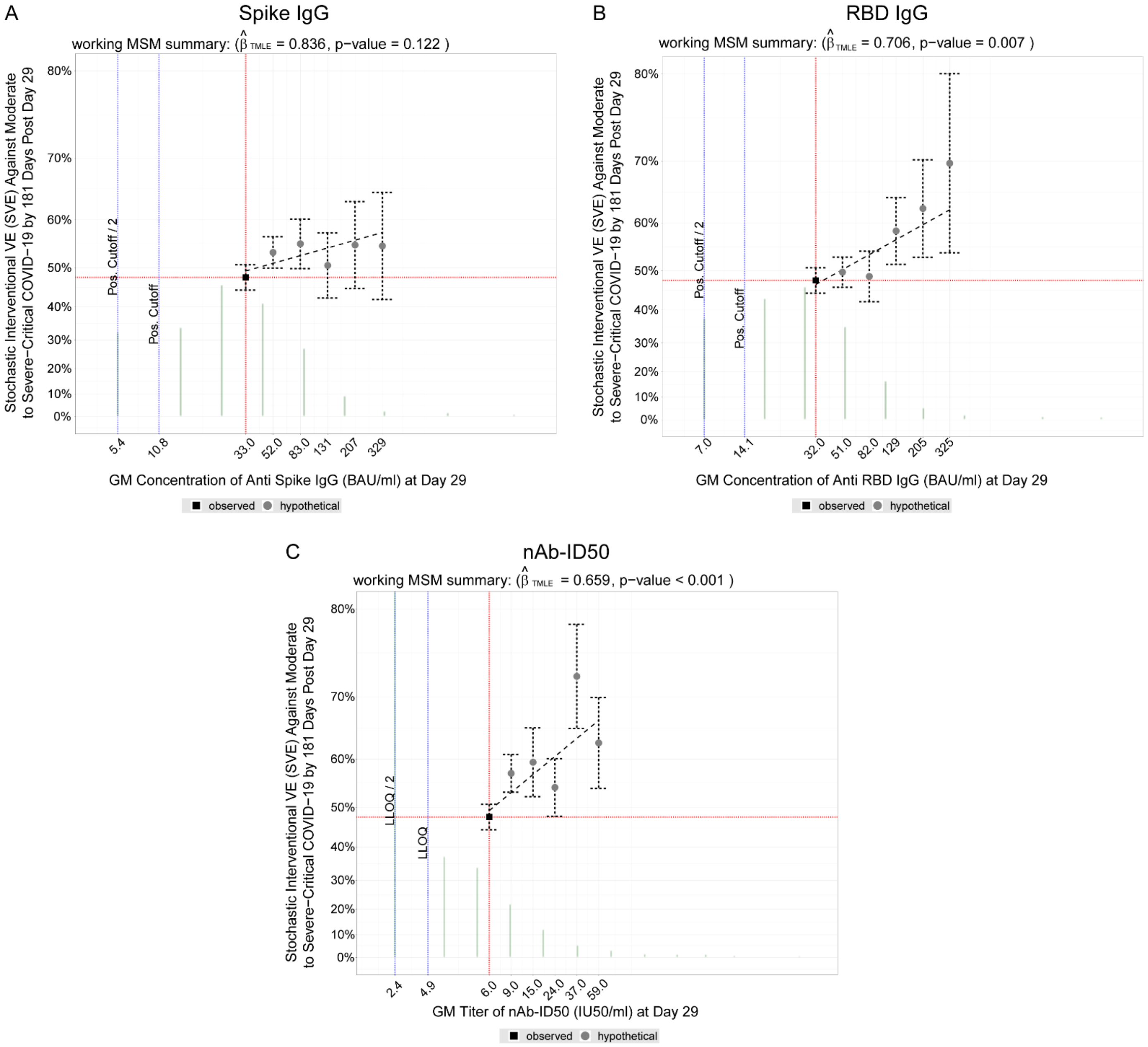

Supplementary Fig. 50. Stochastic interventional vaccine efficacy (SVE) against moderate to severe-critical COVID-19 under hypothetical shifts in D29 nAb-ID50 titer, for participants in Latin America. Analyses were performed in baseline SARS-CoV-2 seronegative per-protocol vaccine recipients. The y-axis plots estimated SVE, with 95% confidence intervals, for a vaccine that elicits hypothetical D29 geometric mean nAb-ID50 titer as indicated on the x-axis. SVE estimates were obtained using the method described in Hejazi et al.[2] The vertical red line corresponds to the geometric mean concentration or titer in the ENSEMBLE study population (baseline negative per-protocol vaccine recipients in the immunogenicity subcohort) and the horizontal red line corresponds to the estimated VE against moderate to severe-critical COVID-19 in ENSEMBLE (by 181 days post D29) at a shift of 0, i.e., the observed marker level. IU, international units; LLOQ, lower limit of quantitation; nAb-ID50, 50% inhibitory dilution neutralizing antibody.

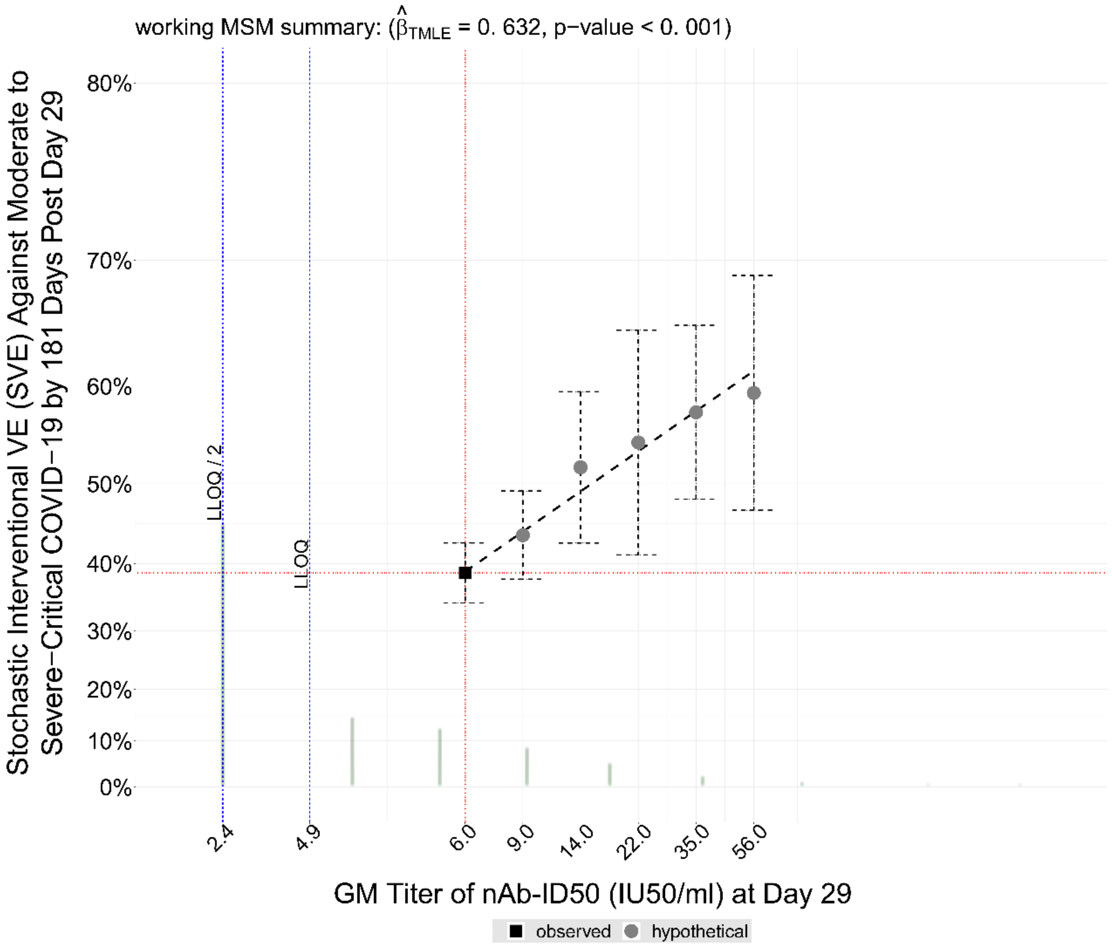

Supplementary Fig. 51. Exposure-proximal vaccine efficacy against severe-critical COVID-19 by current antibody marker level. Analyses were performed in baseline SARS-CoV-2 seronegative per-protocol vaccine recipients. Exposure-proximal vaccine efficacy estimates (through 170 days post-D29) were obtained using the method of Huang and Follmann,[4] with "current" referring to the true underlying antibody marker level not subject to technical measurement error, in a hypothetical scenario in which the value was available from serum samples collected every day over the follow-up period (see Methods). Each point on the curve represents the vaccine efficacy at the given current antibody marker level: **(A)** anti-Spike IgG concentration, **(B)** anti-RBD IgG concentration, **(C)** neutralizing antibody titer (nAb-ID50). The dashed lines are bootstrap pointwise 95% CIs. Analyses adjusted for baseline behavioral risk score and geographic region. Curves are plotted over the range from negative binding antibody response (or unquantifiable neutralizing antibody titer) to the 97.5th percentile of each current antibody marker level: Spike IgG negative response to 448 BAU/ml; RBD IgG, negative response to 579 BAU/ml; nAb-ID50, unquantifiable to 73.4 IU50/ml. Positivity cutoffs: 10.8424 BAU/ml for Spike and 14.0858 BAU/ml for RBD; nAb-ID50 LLOQ=4.8975 IU50/ml. BAU, binding antibody units; CI, confidence interval; IU, international units; LLOQ, lower limit of quantitation; nAb-ID50, 50% inhibitory dilution neutralizing antibody.

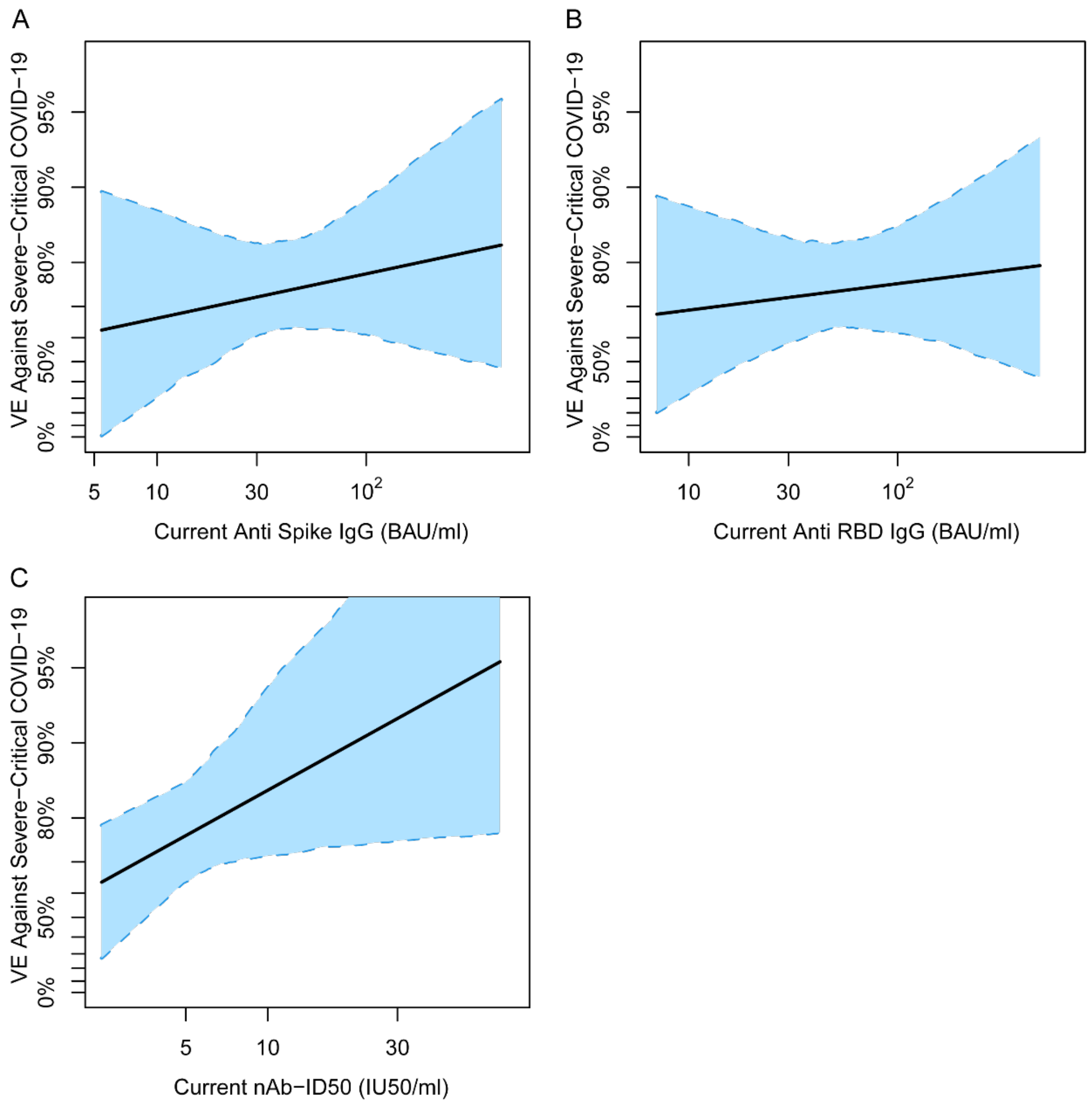

Supplementary Fig. 52. Exposure-proximal vaccine efficacy against moderate COVID-19 by current antibody marker level. Analyses were performed in baseline SARS-CoV-2 seronegative per-protocol vaccine recipients. Exposure-proximal vaccine efficacy estimates (through 181 days post-D29) were obtained using the method of Huang and Follmann,[4] with "current" referring to the true underlying antibody marker level not subject to technical measurement error, in a hypothetical scenario in which the value was available from serum samples collected every day over the follow-up period (see Methods). Each point on the curve represents the vaccine efficacy at the given current antibody marker level: **(A)** anti-Spike IgG concentration, **(B)** anti-RBD IgG concentration, **(C)** neutralizing antibody titer (nAb-ID50). The dashed lines are bootstrap pointwise 95% CIs. Analyses adjusted for baseline behavioral risk score and geographic region. Curves are plotted over the range from negative binding antibody response (or unquantifiable neutralizing antibody titer) to the 97.5th percentile of each current antibody marker level: Spike IgG, negative response to 352 BAU/ml; RBD IgG, negative response to 486 BAU/ml; nAb-ID50, unquantifiable to 43.4 IU50/ml. Positivity cutoffs: 10.8424 BAU/ml for Spike and 14.0858 BAU/ml for RBD; nAb-ID50 LLOQ=4.8975 IU50/ml. BAU, binding antibody units; CI, confidence interval; IU, international units; LLOQ, lower limit of quantitation; nAb-ID50, 50% inhibitory dilution neutralizing antibody.

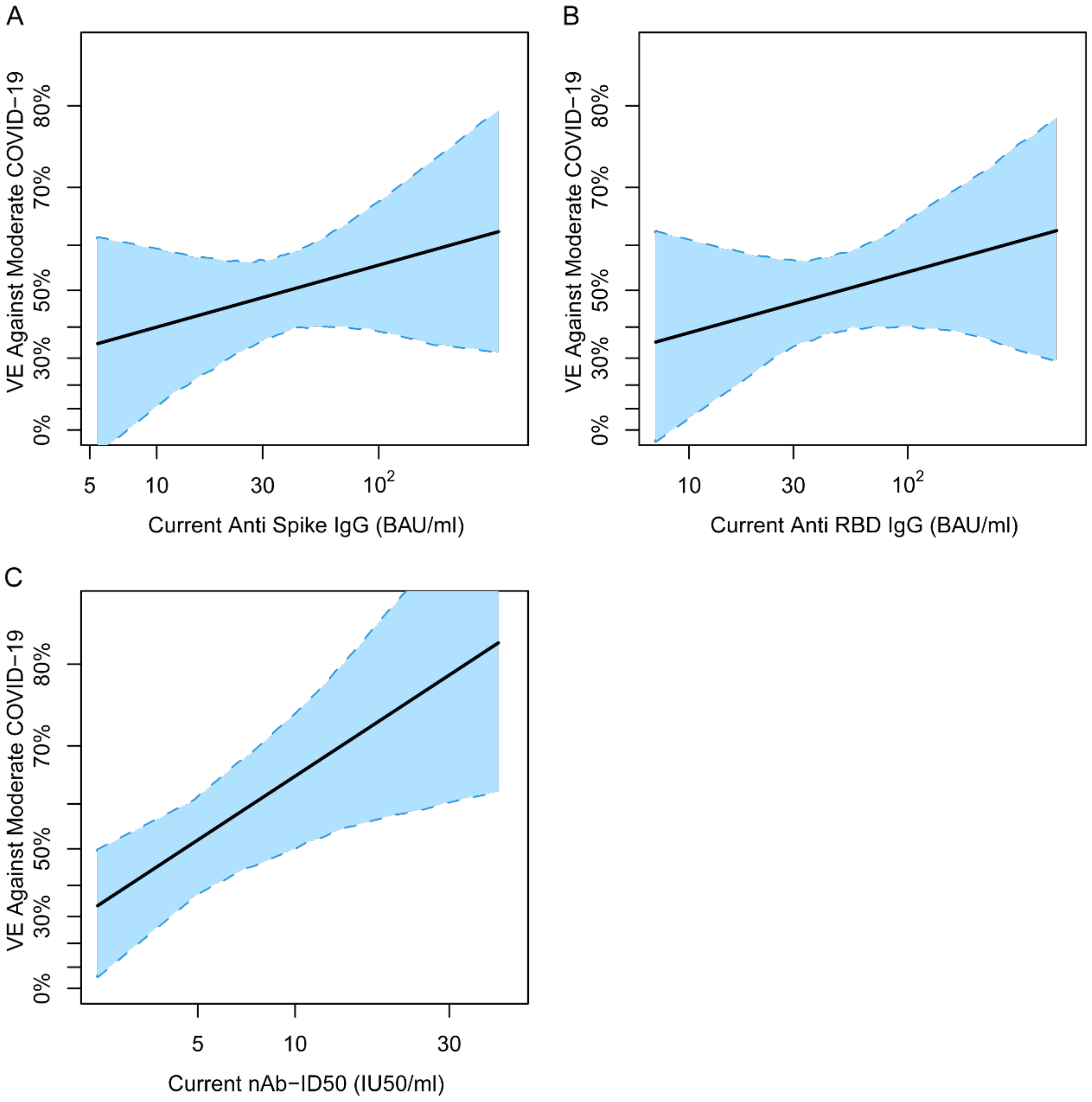

Supplementary Fig. 53. Exposure-proximal vaccine efficacy against moderate to severe-critical COVID-19 by current antibody marker level. Analyses were performed in baseline SARS-CoV-2 seronegative per-protocol vaccine recipients. Exposure-proximal vaccine efficacy estimates (through 181 days post-D29) were obtained using the method of Huang and Follmann,[4] with "current" referring to the true underlying antibody marker level not subject to technical measurement error, in a hypothetical scenario in which the value was available from serum samples collected every day over the follow-up period (see Methods). Each point on the curve represents the vaccine efficacy at the given current antibody marker level: **(A)** anti-Spike IgG concentration, **(B)** anti-RBD IgG concentration, **(C)** neutralizing antibody titer (nAb-ID50). The dashed lines are bootstrap pointwise 95% CIs. Analyses adjusted for baseline behavioral risk score and geographic region. Curves are plotted over the range from negative binding antibody response (or unquantifiable neutralizing antibody titer) to the 97.5th percentile of each current antibody marker level: Spike IgG, negative response to 364 BAU/ml; RBD IgG, negative response to 534 BAU/ml; nAb-ID50, unquantifiable to 44.8 IU50/ml. Positivity cutoffs: 10.8424 BAU/ml for Spike and 14.0858 BAU/ml for RBD; nAb-ID50 LLOQ=4.8975 IU50/ml. BAU, binding antibody units; CI, confidence interval; IU, international units; LLOQ, lower limit of quantitation; nAb-ID50, 50% inhibitory dilution neutralizing antibody.

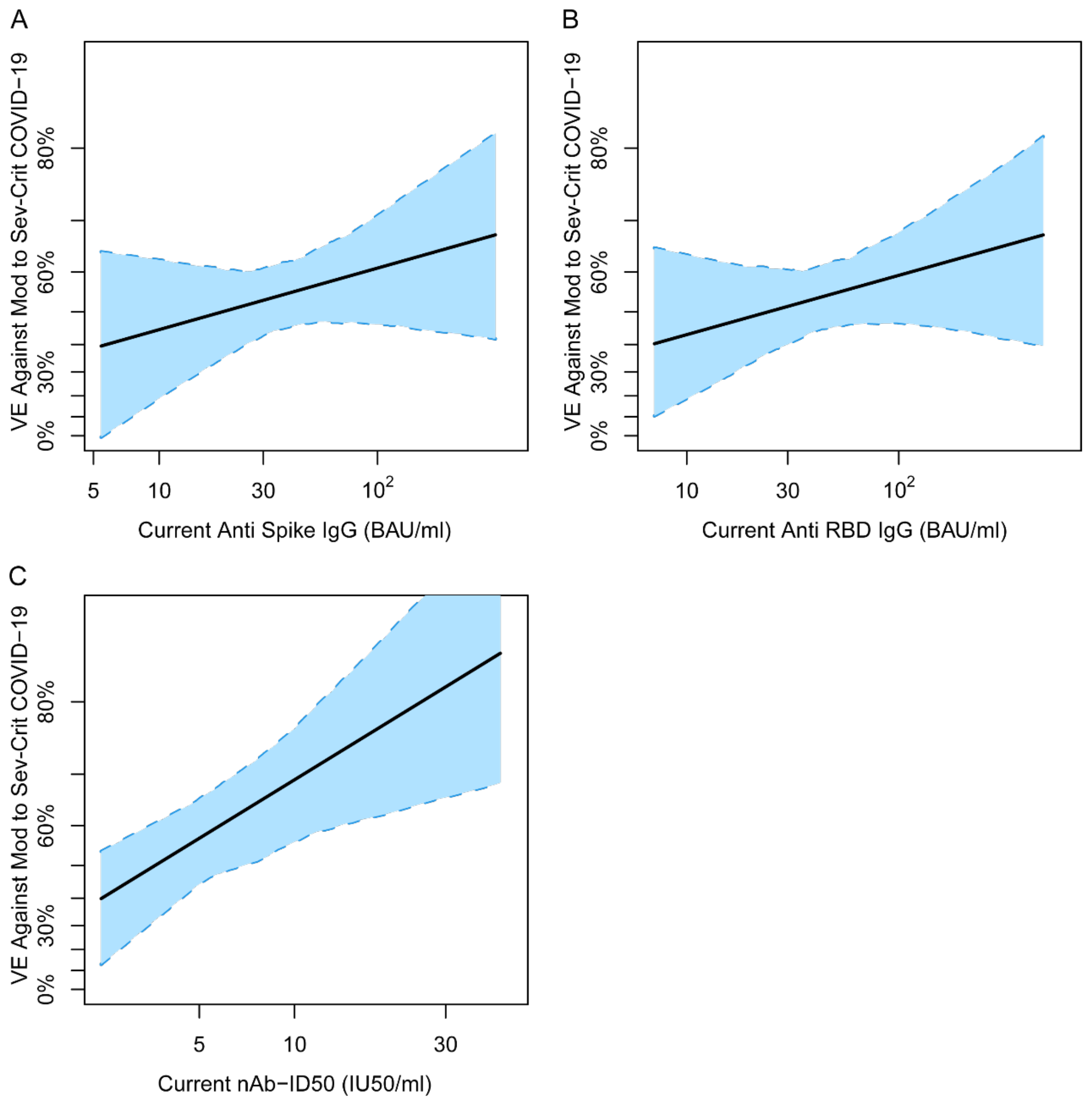

Supplementary Fig. 54. Exposure-proximal vaccine efficacy against severe-critical COVID-19 by current antibody marker level, for the Latin America cohort. Analyses were performed in baseline SARS-CoV-2 seronegative per-protocol vaccine recipients. Exposure-proximal vaccine efficacy estimates (through 170 days post-D29) were obtained using the method of Huang and Follmann,[4] with "current" referring to the true underlying antibody marker level not subject to technical measurement error, in a hypothetical scenario in which the value was available from serum samples collected every day over the follow-up period (see Methods). Each point on the curve represents the vaccine efficacy at the given current antibody marker level: **(A)** anti-Spike IgG concentration, **(B)** anti-RBD IgG concentration, **(C)** neutralizing antibody titer (nAb-ID50). The dashed lines are bootstrap pointwise 95% CIs. Analyses adjusted for baseline behavioral risk score. Curves are plotted over the range from negative binding antibody response (or unquantifiable neutralizing antibody titer) to the 97.5th percentile of each current antibody marker level: Spike IgG, negative response to 410 BAU/ml; RBD IgG, negative response to 611 BAU/ml; nAb-ID50, unquantifiable to 32.0 IU50/ml. Positivity cutoffs: 10.8424 BAU/ml for Spike and 14.0858 BAU/ml for RBD; nAb-ID50 LLOQ=4.8975 IU50/ml. BAU, binding antibody units; CI, confidence interval; IU, international units; LLOQ, lower limit of quantitation; nAb-ID50, 50% inhibitory dilution neutralizing antibody. United States- and South Africa-specific plots are not shown due to the low numbers of vaccine endpoints (6 and 5, respectively), which resulted in substantially wide 95% CIs.

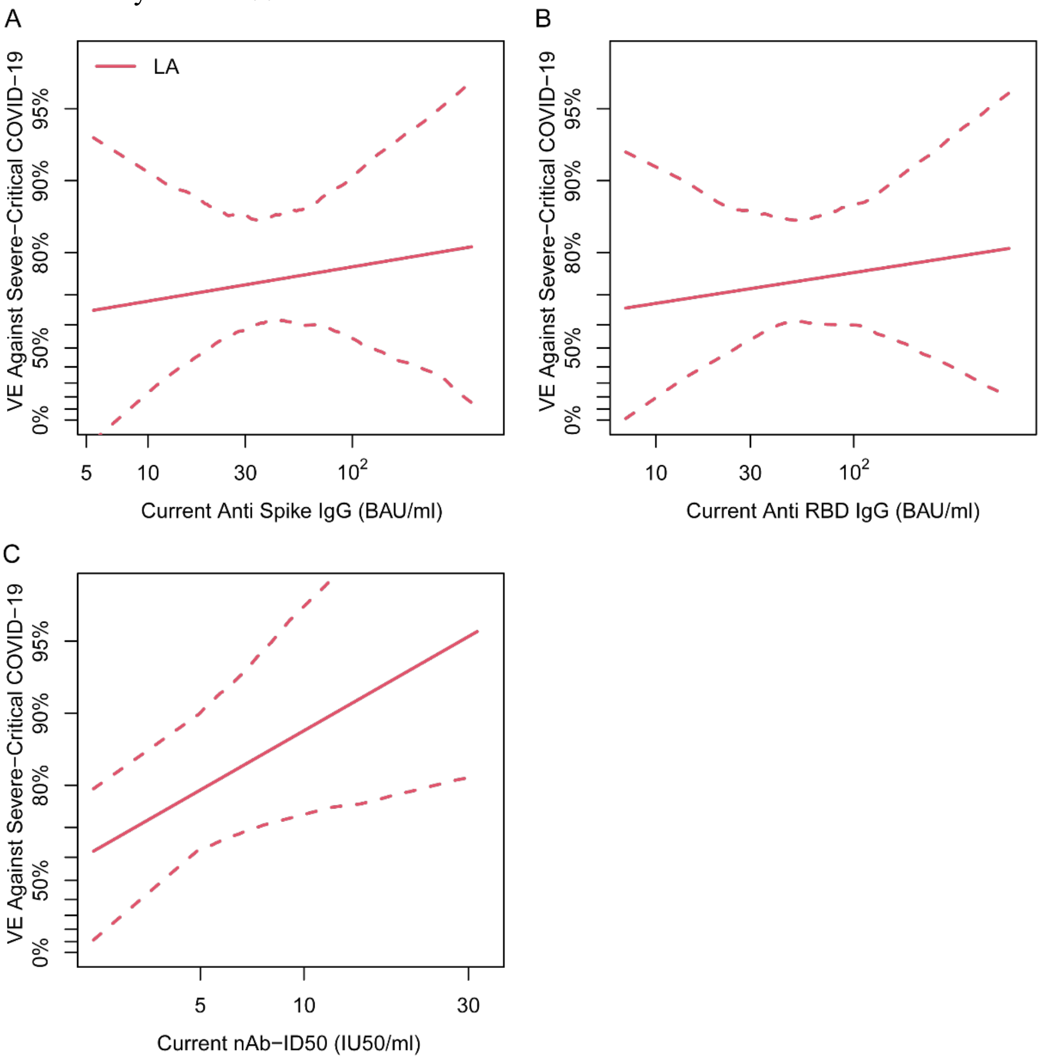

Supplementary Fig. 55. Exposure-proximal vaccine efficacy against moderate COVID-19 by current antibody marker level, for the Latin America cohort. Analyses were performed in baseline SARS-CoV-2 seronegative per-protocol vaccine recipients. Exposure-proximal vaccine efficacy estimates (through 181 days post-D29) were obtained using the method of Huang and Follmann,[4] with "current" referring to the true underlying antibody marker level not subject to technical measurement error, in a hypothetical scenario in which the value was available from serum samples collected every day over the follow-up period (see Methods). Each point on the curve represents the vaccine efficacy at the given current antibody marker level: **(A)** anti-Spike IgG concentration, **(B)** anti-RBD IgG concentration, **(C)** neutralizing antibody titer (nAb-ID50). The dashed lines are bootstrap pointwise 95% CIs. Analyses adjusted for baseline behavioral risk score. Curves are plotted over the range from negative binding antibody response (or unquantifiable neutralizing antibody titer) to the 97.5th percentile of each current antibody marker level: Spike IgG, negative response to 397 BAU/ml; RBD IgG, negative response to 567 BAU/ml; nAb-ID50, unquantifiable to 46.1 IU50/ml. Positivity cutoffs: 10.8424 BAU/ml for Spike and 14.0858 BAU/ml for RBD; nAb-ID50 LLOQ=4.8975 IU50/ml. BAU, binding antibody units; CI, confidence interval; IU, international units; LLOQ, lower limit of quantitation; nAb-ID50, 50% inhibitory dilution neutralizing antibody.

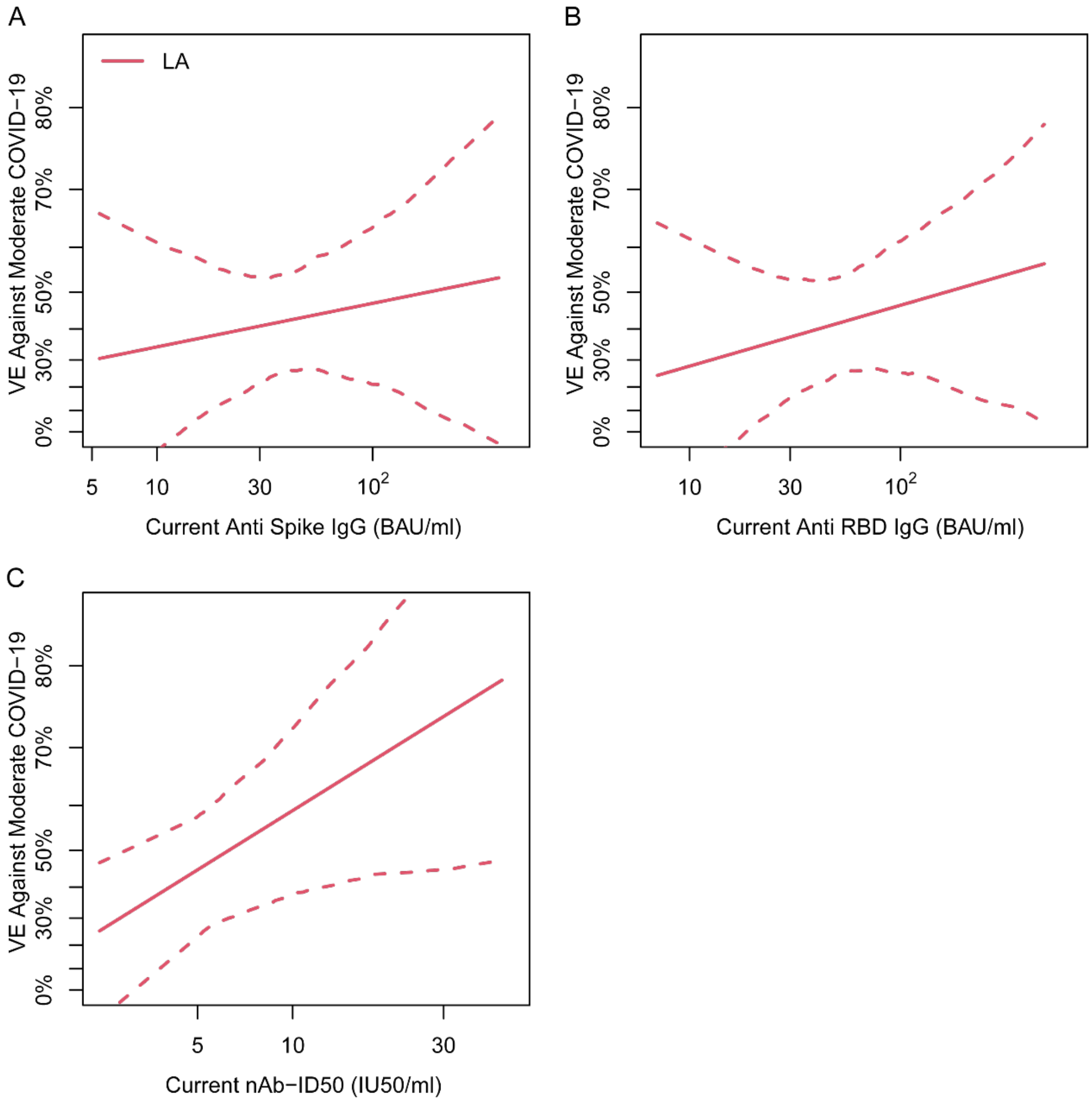

Supplementary Fig. 56. Exposure-proximal vaccine efficacy against moderate to severe-critical COVID-19 by current antibody marker level, shown separately by geographic region. Analyses were performed in baseline SARS-CoV-2 seronegative per-protocol vaccine recipients. Exposure-proximal vaccine efficacy estimates (through 181, 101, or 109 days post-D29 for Latin America, South America, United States, respectively) were obtained using the method of Huang and Follmann,[4] with "current" referring to the true underlying antibody marker level not subject to technical measurement error, in a hypothetical scenario in which the value was available from serum samples collected every day over the follow-up period (see Methods). Each point on the curve represents the vaccine efficacy at the given current antibody marker level: **(A)** anti-Spike IgG concentration, **(B)** anti-RBD IgG concentration, **(C)** neutralizing antibody titer (nAb-ID50). The dashed lines are bootstrap pointwise 95% CIs. Analyses adjusted for baseline behavioral risk score. Curves are plotted over the range from negative binding antibody response (or unquantifiable neutralizing antibody titer) to the 97.5th percentile of each current antibody marker level: Spike IgG, negative response to 408 (Latin America), 328 (South Africa) or 158 (United States) BAU/ml; RBD IgG, negative response to 573 (Latin America), 353 (South Africa) or 187 (United States) BAU/ml; nAb-ID50, unquantifiable to 46.5 (Latin America), 53.1 (South Africa) or 16.7 (United States) IU50/ml. Positivity cutoffs: 10.8424 BAU/ml for Spike and 14.0858 BAU/ml for RBD; nAb-ID50 LLOQ=4.8975 IU50/ml. BAU, binding antibody units; CI, confidence interval; IU, international units; LLOQ, lower limit of quantitation; nAb-ID50, 50% inhibitory dilution neutralizing antibody.

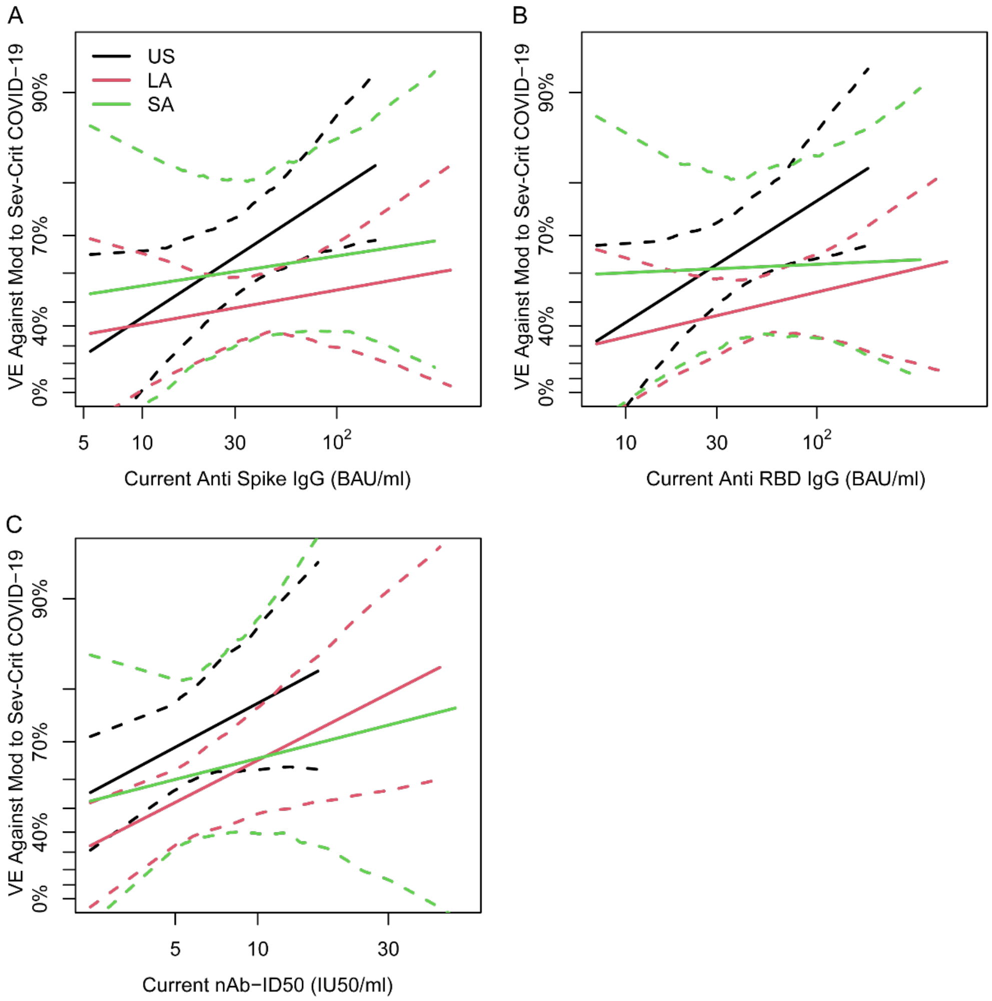

Supplementary Fig. 57. For a random sample of 25 baseline SARS-CoV-2 seronegative vaccine recipients, D29 and D71 A) anti-Spike IgG concentration (BAU/ml), B) anti-RBD IgG concentration (BAU/ml), and C) 50% inhibitory dilution neutralizing antibody titer (nAb-ID50) measurements are shown. The 25 participants were sampled from baseline SARS-CoV-2 seronegative vaccine recipients for whom D29, D71 binding antibody data (both Spike and RBD) and nAb-ID50 data were available. These plots are shown as examples of D29 to D71 antibody trajectories; D29 and D71 antibody data were used in the exposure-proximal correlates analysis as described in Methods. Each line connects the D29, D71 measurements from a single participant. BAU, binding antibody units; IU, international units; LLOQ, lower limit of quantification; Pos.Cut, positivity cutoff; ULOQ, upper limit of quantitation.

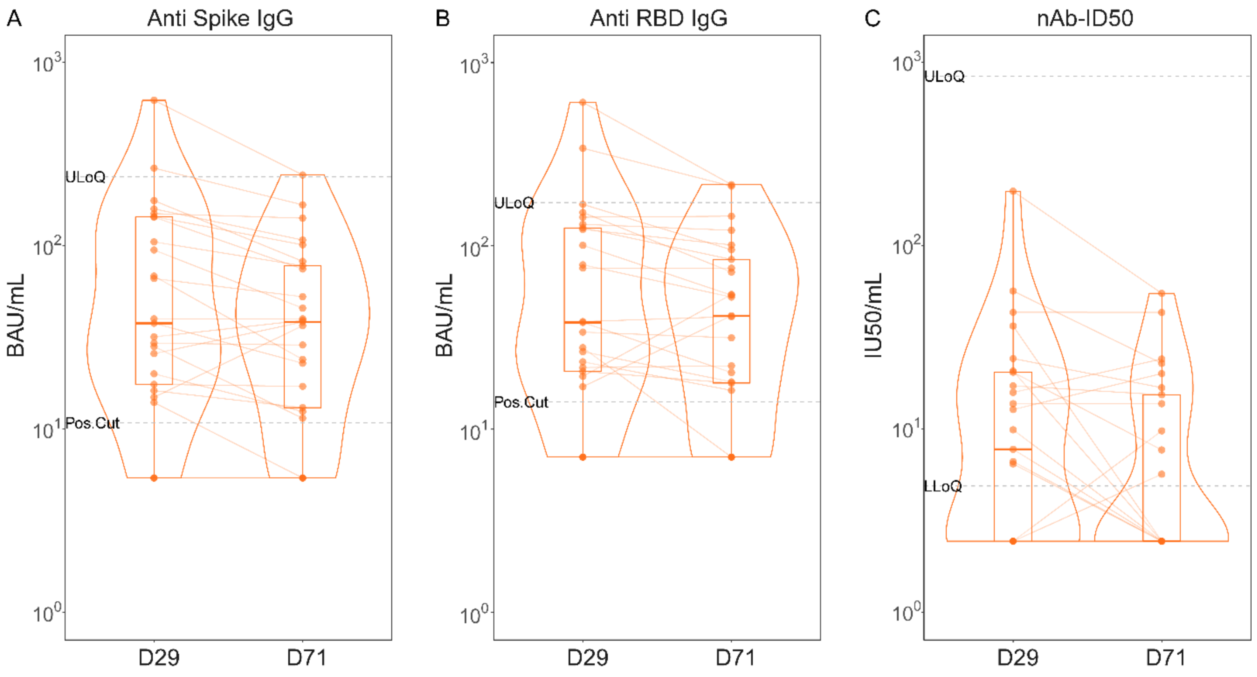

Supplementary Fig. 58. Vaccine efficacy against symptomatic COVID-19 by neutralizing antibody (nAb) ID50 titer in five randomized, placebo-controlled COVID-19 vaccine efficacy trials. Analyses were performed in baseline SARS-CoV-2 negative per-protocol vaccine recipients. Vaccine efficacy (VE) estimates against the primary endpoint in each trial (virologically-confirmed, symptomatic COVID-19) are shown by neutralizing antibody titer (D57 in COVE, D29 in ENSEMBLE-US, D57 in AZD1222, D35 in PREVENT-19, D56 in COV002). Each point on the curve represents the estimated vaccine efficacy at the given nAb-ID50 titer. The dotted lines indicate bootstrap pointwise 95% CIs. The histograms are estimates of the frequency distribution of nAb-ID50 titer. The follow-up periods for the VE assessment were: COVE (doses D1, D29), 7 to 100 days post D57; ENSEMBLE-US (one dose, D1), 7 to 140 days post D29; PREVENT-19 (doses D0, D21), 7 to 59 days post D35; AZD1222 (doses D1, D29), 7 to 92 days post D57; COV002 (doses D1, D29; 7 to approximately 150 days post D57). Curves are plotted over the following antibody marker ranges: COVE: 10 IU50/ml to 97.5th percentile of marker, ENSEMBLE-US: 2.5th percentile to 97.5th percentile, PREVENT-19: 2.5th percentile to 97.5th percentile, AZD1222: 2.5th percentile to 97.5th percentile, COV002: 3 to 140 IU50/ml. Baseline covariates adjusted for were: COVE: baseline risk score, comorbidity status, and Community of color status; ENSEMBLE-US, baseline risk score and geographic region; PREVENT-19: baseline risk score; AZD1222: age, baseline risk score; COV002: baseline risk score. nAb-ID50, 50% inhibitory dilution neutralizing antibody titer. The bottom panel shows the histogram of nAb-ID50 density in each trial.

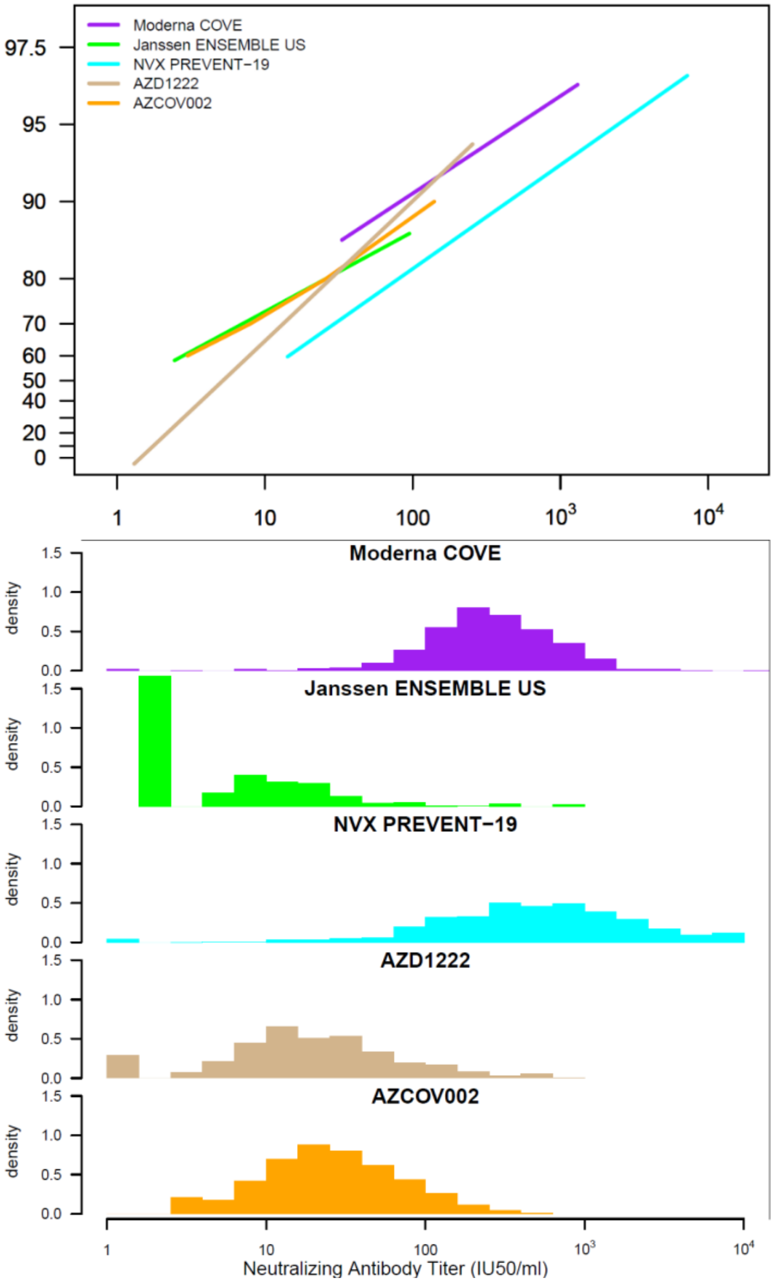

References

1. Gilbert PB, Fong Y, Kenny A, Carone M. A Controlled Effects Approach to Assessing Immune Correlates of Protection. *Biostatistics* 2023; **24**(4): 850–65.
2. Hejazi NS, van der Laan MJ, Janes HE, Gilbert PB, Benkeser DC. Efficient nonparametric inference on the effects of stochastic interventions under two-phase sampling, with applications to vaccine efficacy trials. *Biometrics* 2021; **77**(4): 1241-53.
3. Sadoff J, Gray G, Vandebosch A, et al. Final Analysis of Efficacy and Safety of Single-Dose Ad26.COV2.S. *N Engl J Med* 2022; **386**(9): 847-60.
4. Huang Y, Follmann D. Exposure proximal immune correlates analysis. *Biostatistics* 2024; kxae031. https://doi.org/10.1093/biostatistics/kxae031.
