# Supplementary material for: Neutralizing antibody correlate of protection against severe-critical COVID-19 in the ENSEMBLE single-dose Ad26.COV2.S vaccine efficacy trial
Source: Nat Commun. 2024 Nov 12;15:9785. doi: 10.1038/s41467-024-53727-y (PMC11557889; doi:10.1038/s41467-024-53727-y)
Supplement: Supplementary file 3 — Description of Additional Supplementary Files [file 41467_2024_53727_MOESM3_ESM.docx]

Description of Additional Supplementary Files

**File Name:** Supplementary Sofware 1

**Description:** Code for generating Figure 1 and Supplementary Figure 3.

**File Name:** Supplementary Software 2

**Description:** Code for conducting the stochastic interventional vaccine efficacy analysis (Supplementary Figures 49 and 50).
