# Supplementary material for: Neutralizing antibody correlate of protection against severe-critical COVID-19 in the ENSEMBLE single-dose Ad26.COV2.S vaccine efficacy trial
Source: Nat Commun. 2024 Nov 12;15:9785. doi: 10.1038/s41467-024-53727-y (PMC11557889; doi:10.1038/s41467-024-53727-y)
Supplement: Supplementary file 6 — Reporting Summary [file 41467_2024_53727_MOESM6_ESM.pdf]

nature portfolio

Corresponding author(s): Peter B. Gilbert

Last updated by author(s): Sep 9, 2024

Reporting Summary

Nature Portfolio wishes to improve the reproducibility of the work that we publish. This form provides structure for consistency and transparency in reporting. For further information on Nature Portfolio policies, see our Editorial Policies and the Editorial Policy Checklist.

Statistics

For all statistical analyses, confirm that the following items are present in the figure legend, table legend, main text, or Methods section.

n/a	Confirmed	
☐	☒	The exact sample size (*n*) for each experimental group/condition, given as a discrete number and unit of measurement
☐	☒	A statement on whether measurements were taken from distinct samples or whether the same sample was measured repeatedly
☐	☒	The statistical test(s) used AND whether they are one- or two-sided<br>*Only common tests should be described solely by name; describe more complex techniques in the Methods section.*
☐	☒	A description of all covariates tested
☐	☒	A description of any assumptions or corrections, such as tests of normality and adjustment for multiple comparisons
☐	☒	A full description of the statistical parameters including central tendency (e.g. means) or other basic estimates (e.g. regression coefficient) AND variation (e.g. standard deviation) or associated estimates of uncertainty (e.g. confidence intervals)
☐	☒	For null hypothesis testing, the test statistic (e.g. *F*, *t*, *r*) with confidence intervals, effect sizes, degrees of freedom and *P* value noted<br>*Give P values as exact values whenever suitable.*
☒	☐	For Bayesian analysis, information on the choice of priors and Markov chain Monte Carlo settings
☒	☐	For hierarchical and complex designs, identification of the appropriate level for tests and full reporting of outcomes
☒	☐	Estimates of effect sizes (e.g. Cohen's *d*, Pearson's *r*), indicating how they were calculated

Our web collection on statistics for biologists contains articles on many of the points above.

Software and code

Policy information about availability of computer code

Data collection	For the binding antibody assay: The MSD MESO Sector S 600 detection system quantitates the amount of light emitted and reports the ECL unit response as a result for each test sample, control sample and reference standard of each plate. The system software is proprietary to MSD: https://www.mesoscale.com/en/products_and_services/software.
Data analysis	For the binding antibody assay, MSD Discovery Workbench software (version 4.0) was used for analysis.<br>For the neutralizing antibody assay, data analysis (inhibition curve fitting and ID50 concentrations) was done using Monogram proprietary analysis software.<br>Plots of variants causing the severe-critical cases over time and by region were done in R (version 4.3.1) (Supplementary Software 1).<br>Immune correlates analyses were done reproducibly based on publicly available R scripts (DOI: 10.5281/zenodo.13690802) and the following publicly available R packages: survey (version 4.0), vaccine (version 1.2.1), txshift (version 0.3.8), and sl3 (version 1.4.6).<br>Code for conducting the stochastic interventional vaccine efficacy analysis is available in the Supplementary Software 2 file.

For manuscripts utilizing custom algorithms or software that are central to the research but not yet described in published literature, software must be made available to editors and reviewers. We strongly encourage code deposition in a community repository (e.g. GitHub). See the Nature Portfolio guidelines for submitting code & software for further information.

Data

Policy information about availability of data

All manuscripts must include a data availability statement. This statement should provide the following information, where applicable:

- Accession codes, unique identifiers, or web links for publicly available datasets
- A description of any restrictions on data availability
- For clinical datasets or third party data, please ensure that the statement adheres to our policy

The data sharing policy of Janssen Pharmaceutical Companies of Johnson & Johnson is available at https://www.janssen.com/clinical-trials/transparency. As noted on this site, requests for access to the study data can be submitted through Yale Open Data Access [YODA] Project site at http://yoda.yale.edu. Source data for Figures 2-5 are provided with this paper.

Research involving human participants, their data, or biological material

Policy information about studies with human participants or human data. See also policy information about sex, gender (identity/presentation), and sexual orientation and race, ethnicity and racism.

Reporting on sex and gender	Cell lines: The pseudovirus neutralization assay used the HEK 293 cell line. The HEK 293 cell line was derived from a human fetus, with Lin et al. (https://doi.org/10.1038/ncomms5767) having reported evidence that HEK 293 cells are of female provenance (i.e. the complete absence of any Y-chromosome-derived sequence in high-coverage genomic sequencing data). It is unknown whether alternative neutralization assays would provide different results if based on a HEK cell line derived from a male donor.<br>Human research participants: In the ENSEMBLE trial, information on participant sex was self-reported, solicited, and collected by four predefined options (female, male, unknown, intersex). Sadoff et al. 2021 NEJM (DOI: 10.1056/NEJMoa2101544) determined that sex had no meaningful impact on vaccine efficacy against moderate to severe-critical COVID-19.<br>In the present analysis, sex assigned at birth (female vs. male/undifferentiated/unknown) was included in the list of baseline covariates considered for risk score analysis.<br>Moreover, the SAP for the first ENSEMBLE immune correlates analysis (Fong et al. 2023 Nat Microbiol) prespecified a list of subgroups for which immunogenicity data would be summarized, which included "Sex Assigned at Birth: Male, Female". Supplementary Table 8 in the present manuscript presents geometric mean titers (or concentrations) of D1 and D29 Spike IgG bAb, RBD IgG bAb, and nAb-ID50, each separately by sex, in baseline SARS-CoV-2 seronegative vaccine recipients in the immunogenicity subcohort; Supplementary Fig. 8 presents boxplots with individual-level D29 Spike IgG bAb, RBD IgG bAb, and nAb-ID50 levels, each separately by sex, in baseline SARS-CoV-2 seronegative vaccine recipients in the immunogenicity subcohort.<br>The SAP also prespecified that within baseline seronegative vaccine recipients, antibody levels would be compared for a list of pairs of subgroups, which included male vs. female. Supplementary Table 9 presents the ratios (male/female) of the geometric mean titers (or concentrations) shown in Supplementary Table 8. For the D1 antibody markers, the ratios were 1 or near 1; for the D29 antibody markers, the ratios were slightly below 1. For all ratios, the 95% confidence intervals encompassed 1.<br>For the main objective of this work to assess immune correlates for severe-critical COVID-19, there was insufficient sample size to conduct analyses for males and females separately (across both sexes: 31 severe-critical vaccine endpoints in Latin America, 5 in South Africa, and 6 in the US).
Reporting on race, ethnicity, or other socially relevant groupings	While the present manuscript does not report on these groupings, the primary manuscripts (Sadoff et al. 2021 NEJM, Sadoff et al. 2022 NEJM) reported on Race or ethnic group (American Indian or Alaskan Native, Indigenous South American, Asian, Black, Native Hawaiian or other Pacific Islander, White, Multiracial, Not reported/unknown/missing) as well as Hispanic ethic group (Hispanic, Non-Hispanic, Not reported/unknown/missing). Race and ethnic group were reported by the participants. American Indian or Alaskan Native was reported only by participants residing in the United States.
Population characteristics	Supplementary Tables 2, 3, 4, and 5 provide comprehensive information on demographics and clinical characteristics of the baseline SARS-CoV-2 seronegative per-protocol trial participants in the immunogenicity subcohort (IS), the Latin America subset of the IS, the South Africa subset of the IS, and the United States subset of the IS, respectively.
Recruitment	To ensure diversity and inclusion in the ENSEMBLE trial and based on years of clinical trial experience, Janssen implemented a multifaceted plan for recruitment and enrollment of participants from underrepresented communities. The approach included intentional site selection, community engagement and awareness building, and educational and training support for investigators. Janssen also took steps to remove barriers clinical trial participants often face, including the use of demographic data to identify and utilize clinical trial sites located in underrepresented communities.<br>"We are committed to developing medicines and therapies that meet the needs of all people, and we know that diseases and drugs may impact people differently based on their race and ethnicity, so the alignment of clinical trial enrollment with patient population demographics is key," said Staci Hargraves, Vice President of Patient and Portfolio Solutions, Janssen Research & Development, LLC, and Executive Sponsor of Janssen's Diversity, Equity & Inclusion in Clinical Trials program. "Simple yet impactful decisions, such as making sure trial sites were located in accessible places within historically underserved communities, made a big difference in our ability to reach more participants."<br>Once Janssen selected the ENSEMBLE sites and began recruitment efforts, Janssen's employees built relationships with trial site investigators and staff to provide cultural competency training to help stimulate dialogue about diversity and maintain focus on enrolling and supporting underrepresented groups. These close collaborations with site leaders allowed Janssen to identify any roadblocks in real time and make changes to the recruitment efforts as needed.<br>Identifying clinical trial sites in diverse communities was only the first step, because other barriers to recruitment and

enrollment also exist. Clinical research in the U.S. has a complicated history when it comes to marginalized populations. Past events such as the Tuskegee Syphilis Study, combined with ongoing systemic disparities in the healthcare system, have contributed to distrust in clinical research among many people. Building trust is critical, particularly given the urgency the pandemic presented.

“We felt it was our role to help people understand how clinical trials work — and how trials have evolved to ensure that participant safety and human rights are protected today,” said Hargraves.

To build trust with communities of color, Janssen worked with both local and national organizations, including prominent community advocacy groups and leaders, along with healthcare professional organizations. These groups helped Janssen identify trusted voices within communities who could disseminate information about ENSEMBLE and clinical research in general. Janssen also used its Research Includes Me patient education program to conduct local outreach, including the consumer-facing website ResearchIncludesMe.com, and the dispatch of mobile units of bilingual educators to large community events. These tools helped to dispel misinformation about present-day medical research by providing accessible and empowering education about the clinical trial process and the protections given to participants’ rights and privacy.

source: https://www.jnj.com/our-company/janssen-takes-multifaceted-approach-to-ensuring-diversity-equity-and-inclusion-in-its-covid-19-vaccine-trial

The fact that the trial was a randomized trial, with careful allocation concealment, minimizes the potential for selection bias. As stated in the Protocol (available with Sadoff et al. NEJM 2021): A placebo control was used to establish the frequency and magnitude of changes in clinical and immunological endpoints that may occur in the absence of active vaccine. Randomization was used to minimize bias in the assignment of participants to vaccine groups, to increase the likelihood that known and unknown participant attributes (eg, demographic and baseline characteristics) were evenly balanced across vaccine groups, and to enhance the validity of statistical comparisons across vaccine groups. Blinded study vaccine was used to reduce potential bias during data collection and evaluation of study endpoints. Blinding was guaranteed by the preparation of the study vaccine by an unblinded pharmacist or other qualified study-site personnel with primary responsibility for study vaccine preparation and dispensing, and by the administration of vaccine in a masked syringe by a blinded study vaccine administrator. Participants were randomly assigned to 1 of the groups based on a computer-generated randomization schedule prepared before the study by or under the supervision of the sponsor and using the interactive web response system.

Ethics oversight

The COV3001 (ENSEMBLE) study was reviewed and approved by all relevant local ethics committees and Institutional Review Boards, listed below:

Argentina: ANMAT - Administración Nacional de Medicamentos, Alimentos y Tecnologia Médica (Capital Federal, La Plata, Ramos Mejia – Buenos Aires; Ciudad Autonoma de Buenos Aires), Comite de Etica Dr Carlos Barclay (Capital Federal, Buenos Aires; Ciudad Autonoma de Buenos Aires), Comision Conjunta de Investigacion en Salud – CCIS (La Plata, Ramos Mejia - Buenos Aires), Comite de Bioetica de Fundacion Huesped (Ciudad Autonoma de Buenos Aires), Comité de Docencia e Investigación DIM Clínica Privada (Ramos Mejia, Buenos Aires), Comité de Ética en Investigación Clínica y Maternidad Suizo Argentina (Ciudad Autonoma de Buenos Aires), Comité de Ética en Investigación de CEMIC (Ciudad Autonoma de Buenos Aires), Comite de Etica en Investigacion DIM Clinica Privada (Ramos Mejia, Buenos Aires), Comite de Etica Hospital Italiano de La Plata (La Plata, Buenos Aires), Comite de Etiica en Investigacion Hospital General de Agudos J.M. Ramos Mejia (Ciudad Autonoma de Buenos Aires), Comitéde ética del Instituto Médico Platense (CEDIMP) (La Plata, Buenos Aires), IBC Fundacion Huesped (Ciudad Autonoma de Buenos Aires), IBC Helios Salud (Ciudad Autonoma de Buenos Aires), IBC Hospital General de Agudos J.M. Ramos Mejia (Ciudad Autonoma de Buenos Aires)

Brazil: ANVISA – Agência Nacional de Vigilância Sanitária (Salvador, Bahia; Barretos, Campinas, São Paulo, São Jose Rio Preto, Ribeirão Preto, São Caetano do Sul – São Paulo; Santa Maria, Porto Alegre – Rio Grande do Sul; Natal, Rio Grande do Norte; Para, Pará; Belo Horizonte, Minas Gerais; Rio de Janeiro, Nova Iguaçu – Rio de Janeiro; Curitiba, Paraná; Brasília, Distrito Federal; Campo Grande, Mato Grosso do Sul; Criciúma, Santa Catarina; Cuiabá, Mato Grosso), CONEP - Comissão Nacional de Ética em Pesquisa (Salvador, Bahia; São Paulo, São Paulo; Santa Maria, Rio Grande do Sul; Para, Pará;), CAPPESq – Comissão de Ética de Análise para Projetos de Pesquisa – HCFMUSP (São Paulo, São Paulo), CEP da Faculdade de Medicina de São José do Rio Preto – FAMERP (São Jose Rio Preto, São Paulo), CEP da Faculdade de Medicina do ABC/SP (São Paulo, São Paulo), CEP da Fundação Pio XII - Hospital do Câncer de Barretos/SP (Barretos, São Paulo), CEP da Liga Norteriograndense Contra o Câncer (Natal, Rio Grande do Norte), CEP da Pontificia Universidade Catolica de Campinas / PUC Campinas (Campinas, São Paulo), CEP da Real Benemérita Associaçao Portuguesa de Beneficência - Hospital São Joaquim (São Paulo, São Paulo), CEP da Santa Casa de Misericórdia de Belo Horizonte (Belo Horizonte, Minas Gerais), CEP da Secretaria Municipal De Saúde do Rio de Janeiro – SMS/RJ (Rio de Janeiro, Rio de Janeiro), CEP da Universidade de São Caetano do Sul (CEP da Universidade de São Caetano do Sul, São Paulo), CEP da Universidade Federal de Mato Grosso do Sul – UFMS (Campo Grande, Mato Grosso do Sul), CEP da Universidade Federal de Minas Gerais (Belo Horizonte, Minas Gerais), CEP do Centro de Referência e Treinamento DST/AIDS (São Paulo, São Paulo), CEP do do INI-Ipec/Fiocruz (Rio de Janeiro, Rio de Janeiro), CEP do Grupo Hospitalar Conceição / RS (Porto Alegre, Rio Grande do Sul), CEP do Hospital das Clínicas da Faculdade de Medicina de Ribeirão Preto/USP (Ribeirão Preto, São Paulo), CEP do Hospital de Clinicas da Universidade Federal do Parana - HCUFPR / PR (Curitiba, Paraná), CEP do Hospital de Clínicas de Porto Alegre/HCPA (Porto Alegre, Rio Grande do Sul), CEP do Hospital Geral de Nova Iguaçu (Nova Iguaçu, Rio do Janeiro), CEP do Hospital Municipal São José (Criciúma, Santa Catarina), CEP do Hospital Pró-Cardíaco/RJ (Rio de Janeiro, Rio de Janeiro), CEP do Hospital Sírio Libanês (São Paulo, Sao Paulo), CEP do Hospital Universitário Júlio Muller / MT (Cuiabá, Mato Grosso), CEP do Hospital Universitário Professor Edgard Santos – UFBA (Salvador, Bahia), CEP do Instituto de Cardiologia do Distrito Federal (Brasília, Distrito Federal), CEP do Instituto de Infectologia Emílio Ribas/SP (São Paulo, Sao Paulo), CEP do Instituto de Saude e Bem Estar da Mulher - ISBEM / SP (São Paulo, Sao Paulo), CEP em Seres Humanos do HFSE - Hospital Federal dos Servidores do Estado (Rio de Janeiro, Rio de Janeiro), CONEP - Comissão Nacional de Ética em Pesquisa (Brasília, Distrito Federal, Salvador, Bahia; Belo Horizonte, Minas Gerais; Cuiabá, Mato Grosso; Campo Grande, Mato Grosso do Sul; Nova Iguaçu, Rio Janeiro – Rio Janeiro; Barretos, Campinas, Sao Jose Rio Preto, São Caetano do Sul, Sao Paulo, Ribeirão Preto – Sao Paulo; Porto Alegre, Rio Grande do Sul; Natal, Rio Grande do Norte; Curitiba, Paraná; Criciúma, Santa Catarina)

Chile: Comité de Ética de Investigación en Seres Humanos (Santiago, Region Met), Comité Ético Científico Servicio de Salud Metropolitano Central (Santiago, Region Met), Instituto de Salud Pública de Chile (Santiago, Region Met; Talca, Temuco), Comité Ético-Científico Servicio de Salud Metropolitano Sur Oriente (Talca, Santiago), Comité de Evaluación Ética Científica Servicio de Salud Araucanía Sur Temuco (Temuco), Comité Ético Científico Servicio de Salud Metropolitano Central (Viña del Mar)

Colombia: CEI de la Fundación Cardiovascular de Colombia (Floridablanca), Comité de Ética en Investigación Clínica de la

Costa (Barranquilla), INVIMA - Instituto Nacional de Vigilancia de Medicamentos y Alimentos (Colombia) (Barranquilla), Comite de Etica en Investigacion de la E.S.E. Hospital Mental de Antioquia (Santa Marta), Comite de Etica en la Investigacion CAIMED (Bogotá), INVIMA - Instituto Nacional de Vigilancia de Medicamentos y Alimentos (Colombia) (Bogotá), Comite Corporativo de Etica en Investigacion de la Fundacion Santa Fe de Bogota (Bogotá), Comité de Ética e Investigación Biomédica de la Fundación Valle del Lili (Cali), Comite de Etica e Investigacion IPS Universitaria (Medellin), Comite de Etica en Investigacion Asustencial Cientifica de Alta Complejidad (Bogotá), Comite de Etica en Investigacion Biomedica de la Corporacion Cientifica Pediatrica de Cali (Cali), Comité de Ética en Investigación Clínica de la Costa (Barranquilla), Comite de Etica en Investigacion de la E.S.E. Hospital Mental de Antioquia (Barrio Barzal Villavicencio), Comite de Etica en Investigacion del area de la Salud de la Universidad del Norte (Barranquilla), Comite de Etica en Investigacion Medplus Centro de Recuperación Integral S.A.S (Bogotá), Comité de Ética en Investigaciones CEI-FOSCAL (Floridablanca), Comite de Etica en la Investigacion CAIMED (Bogotá), Comite de Etica para Investigacion Clinica(CEIC) de la Fundacion Centro de Investigacion Clinica CIC (Medellin), Comite de Investigaciones y Etica en Investigaciones Hospital Pablo Tobon Uribe (Medellin), INVIMA - Instituto Nacional de Vigilancia de Medicamentos y Alimentos (Colombia) (Barranquilla, Bogotá, Cali, Floridablanca, Medellin

Mexico: CEI del Hospital Civil de Guadalajara Fray Antonio Alcalde (Guadalajara, Jalisco), CEI Hospital La Mision (Tijuana, Baja California Norte), CI del Hospital Civil de Guadalajara Fray Antonio Alcalde (Guadalajara, Jalisco), CI Hospital La Mision (Tijuana, Baja California Norte), Comite de Bioseguridad del Instituto Nacional de Salud Publica (Mexico, Distrito Federal; Cuernavaca, Morelos), Comite de Etica en Investigacion del Instituto Nacional de Salud Publica (Mexico, Distrito Federal; Cuernavaca, Morelos), Comité de Bioseguridad del Hospital La Misión S.A. de C.V. (Tijuana, Baja California Norte; Oaxaca, Oaxaca; Merida, Yucatán; Tijuana, Baja California Norte), Comité de Bioseguridad de la Coordinación de Investigación en Salud (IMSS) (Mexico, Estado de Mexico), Comité de Bioseguridad de Médica Rio Mayo (CLINBOR) (Mexico, Distrito Federal), Comité de Bioseguridad del Hospital Universitario "Dr. José Eleuterio González" (Monterrey, Nuevo León), COFEPRIS (Comisión Federal para la Protección contra Riesgos Sanitarios) (Cuernavaca, Morelos; Mexico, Distrito Federal; Monterrey, Nuevo León; Oaxaca, Oaxaca; Merida, Yucatán), Comite de Etica de la Fac de Med de la UANL y Hospital Universitario "Dr. Jose Eleuterio Gonzalez" (Monterrey, Nuevo León), Comite de Etica en Investigacion de la Unidad de Atencion Medica e Investigacion en Salud S.C. (Merida, Yucatán), Comite de Etica en Investigacion de Medica Rio Mayo S.C. (Mexico, Distrito Federal), Comite de Etica en Investigacion de Oaxaca Site Management Organization, S.C. (Oaxaca, Oaxaca), Comite de Etica en Investigacion del Centro Medico Nacional Siglo XXI (IMSS) (Mexico, Estado do Mexico), Comité de Investigación de la Coordinación de Investigación en Salud (IMSS) (Mexico, Estado do Mexico), Comite de Investigacion de la Unidad de Atencion Medica e Investigacion en Salud S.C. (Merida, Yucatán), Comite de Investigacion de Oaxaca Site Management Organization, S.C. (Oaxaca, Oaxaca), Comité de Investigación del Hospital Universitario José Eleuterio González (Monterrey, Nuevo León), Comite de Investigacion Medica Rio Mayo, S.C. (Mexico, Distrito Federal)

Peru: Comite Nacional Transitorio de Etica en Invest. de los Ensayos Clinicos de la enfermedad COVID-19 (Iquitos - Maynas, Loreto; Lima, San Miguel – Lima), INS - Instituto Nacional de Salud (Peru) (Lima, San Miguel – Lima; Callao; Iquitos – Maynas, Loreto)

South Africa: Department Agriculture, Forestry and Fisheries (DAFF) (Port Elizabeth, Mthatha – Eastern Cape; Cape Town, Worcester – Western Cape; Durban, Ladysmith, Vulindlela – KwaZulu-Natal; Johannesburg, Pretoria, Mamelodi East, Soweto, Tembisa – Gauteng; Rustenburg, Klerksdorp – North West; Bloemfontein, Free State; Middelburg, Mpumalanga; Dennilton, Limpopo), Pharma Ethics (Port Elizabeth, Eastern Cape; Durban, Ladysmith – KwaZulu-Natal; Cape Town, Western Cape; Pretoria, Mamelodi East, Johannesburg, Tembisa – Gauteng; Rustenburg, Klerksdorp – North West; Bloemfontein, Free State; Middelburg, Mpumalanga; Dennilton, Limpopo), SAHPRA - South African Health Products Regulatory Authority (Port Elizabeth, Mthatha – Eastern Cape; Cape Town, Worcester – Western Cape; Durban, Ladysmith, Vulindlela – KwaZulu-Natal; Johannesburg, Pretoria, Mamelodi East, Soweto, Tembisa – Gauteng; Rustenburg, Klerksdorp – North West; Bloemfontein, Free State; Middelburg, Mpumalanga; Dennilton, Limpopo), WIRB (Mamelodi East, Pretoria – Gauteng; Ladysmith, KwaZulu-Natal; Bloemfontein, Free State; Cape Town, Western Cape; Dennilton, Limpopo), Wits Health Consortium (Soweto, Johannesburg – Gauteng; Ladysmith, KwaZulu-Natal; Mthatha, Eastern Cape), Wits Institutional Biosafety Committee (Soweto, Pretoria, Johannesburg, Tembisa – Gauteng; Rustenburg, Klerksdorp – North West; Mthatha, Eastern Cape), University of Cape Town HREC (Cape Town, Worcester – Western Cape); University of Cape Town Institute of Infectious Disease & Molecular Medicine (Cape Town, Worcester – Western Cape), University of Cape Town Institutional Biosafety Committee (Cape Town, Worcester – Western Cape), SAMRC Human Research Ethics Committee Scientific Review (Durban, KwaZulu-Natal), Sefako Makgatho University Research Ethics Committee (SMUREC) (Pretoria, Gauteng), University of KwaZulu Natal Institutional Biosafety Committee (Durban, KwaZulu-Natal), University of KwaZulu-Natal Ethics (Durban, Vulindlela – KwaZulu-Natal), University of Stellenbosch Ethics Committee (Cape Town, Western Cape), University of KwaZulu Natal Institutional Biosafety Committee (Vulindlela, KwaZulu-Natal)

United States: Advarra IBC (Detroit, MI; Chapel Hill, NC; Boston, MA; Seattle, WA; Winston-Salem, NC; Austin, TX; Peoria, IL; Huntsville, AL; Long Beach, CA; Tucson, AZ), Biomedical Institute of New Mexico - IBC (Albuquerque, NM), Birmingham VA Medical Center - Alabama- IBC (Birmingham, AL), Clinical Biosafety Services (Hollywood, FL), Columbia University IBC (New York, NY), Copernicus Group IRB (Austin, Dallas, Houston, San Antonio – TX; Rochester, New York, Bronx, Binghamton – NY; Hillsborough, Hackensack, Newark, New Brunswick – NJ; West Palm Beach, Coral Gables, Hollywood, Miami, Orlando, Gainesville, Tampa, Hallandale Beach, Pinellas Park, The Villages, Jacksonville, Deland – FL; Fort Worth, Dallas, San Antonio – TX; Norfolk, Charlottesville – VA; Matairie, New Orleans – LA; Nashville, Knoxville, Memphis, Bristol – TN; Cincinnati, Cleveland, Columbus, Akron – OH; Detroit, Ann Arbor, Grand Rapids – MI; Philadelphia, Pittsburgh – PA; Stanford, San Diego, San Francisco, Oakland, Long Beach, Anaheim, Sacramento, West Hollywood – CA, Las Vegas, Reno – NV; Chicago, Peoria – IL; Omaha, NE; Mobile, Birmingham, Huntsville – AL; St Louis, Greer, Kansas City – MO; Boston, MA; Harrisburg, SD; Decatur, Atlanta, Savannah – GA; Baltimore, Rockville, Annapolis – MD; New Haven, Hartford – CT; Chapel Hill, Raleigh, Fayetteville, Charlotte, Durham, Winston-Salem – NC; Indianapolis, Valparaiso, Evansville – IN; Seattle, WA; Aurora, CO; Lexington, Louisville – KY; Murray, West Jordan, Salt Lake City – UT; Phoenix, Tucson, Glendale – AZ; Spartanburg, Columbia, North Charleston, Anderson, Charleston, Mount Pleasant – SC; Portland, Medford, Corvallis – OR; Albuquerque, Gallup – NM; Little Rock, AR; Jackson, MS; Newport News, VA, Minneapolis, MN; Lenexa, KS), WIRB (Hackensack, NJ; Dallas, TX; Baltimore, MD; Chicago, IL; Aurora, CO; Winston-Salem, NC; Minneapolis, MN; Orlando, Miami, Gainesville – FL; Philadelphia, Pittsburgh – PA; Boston, MA; St Louis, MO; Bronx, New York, NY; New Brunswick, NJ; Phoenix, AZ; Birmingham, AL; Louisville, KY; Albuquerque, NM; New Orleans, LA; Baltimore, MD; San Francisco, CA; Tampa, FL; Aurora, CO; Columbia, SC; Decatur, GA; Reno, NV; Raleigh, NC; Little Rock, AS), Clinical Biosafety Services (Dallas, San Antonio – TX; San Diego, CA; Lexington, KY; Murray, UT; Greer, Kansas City, St Louis – MO; Rockville, MD; Las Vegas, NV; Cincinnati, Columbus, Akron – OH; Phoenix, Tucson, Glendale – AZ; North Charleston, Anderson – SC; Orlando, Pinellas Park, The Villages, Miami – FL; Birmingham, AL; Valparaiso, Evansville – IN; Lenexa, KS), Columbia University IBC (Bronx, New York), Durham VA Medical Center-IBC (Raleigh,

NC), Emory University IRB (Decatur, GA), Environmental Health and Safety Office (Atlanta, GA), Institutional Biosafety Committee (New Orleans, LA), James A. Haley Veterans Hospital_IBC (Tampa, FL), Jesse Brown VA Medical Center- IBC (Chicago, IL), Mass General Brigham IBC (Boston, MA), Mount Sinai- Icahn School of Medicine IBC (New York, NY), New York Blood Center IBC (New York, NY), OHSU IBC (Portland, OR), Partners Institutional Biosafety Committee (Boston, MA), Rocky Mountain Regional VA Medical Center-IBC (Aurora, CO), Rush University Medical Center (Chicago, IL), Rush University Medical Center IBC (Chicago, IL), Rutgers Institutional Biosafety Committee (New Brunswick, NJ), Saint Louis University IBC (St Louis, MO), Saint Michael's Medical Center IRB (Newark, NJ), Southeast Louisiana Veterans Health Care System IBC (New Orleans, LA), St. Jude Children's Research Hospital IBC Committee (Memphis, TN), St. Jude Children's Research Hospital IRB (Memphis, TN), Stanford University Administrative Panel on Human Subjects in Medical Research (Stanford, CA), Temple University – IBC (Philadelphia, PA), The University of Chicago Institutional Biosafety Committee (Chicago, IL), UAMS IBC (Little Rock, AS), UIC IBC (Chicago, IL), University of Alabama at Birmingham Institutional Biosafety Committee (Birmingham, AL), University of Arkansas IRB (Little Rock, AS), University of Kentucky Biological Safety (Lexington, KY), University of Kentucky IRB (Lexington, KY), University of Louisville IRB (Louisville, KY), University of Miami-IBC (Miami, FL), University of Mississippi Medical Center IRB (Jackson, MI), University of Pennsylvania Institutional Biosafety Committee (Philadelphia, PA), University of Pittsburgh IBC (Pittsburgh, Pennsylvania), University of South Florida IRB (Tampa, FL), University of Utah Institutional Biosafety Committee (Salt Lake City, UT), University of Utah IRB (Salt Lake City, UT), UTHealth – IBC (Houston, TX), VA Baltimore Research & Education Foundation (BREF)- IBC (Baltimore, MD), VA Central Arkansas Veterans Healthcare System-IBC (Little Rock, AS), VA James J. Peters Department of VA Medical Center-IBC (Bronx, NY), VA Medical Center - Atlanta-IBC (Decatur, GA), VA Medical Center San Francisco- IBC (San Francisco, CA), VA North Florida/South Georgia IBC (Gainesville, FL), VA North Texas Health Care System IBC (Dallas, TX), VA San Diego Healthcare System IBC (Phoenix, AZ), VA Sierra Nevada Health Care System-IBC (Reno, NV), Vanderbilt University Instituitional Review Board (Nashville, TN), Washington University IBC (St Louis, MO), WCG IBCS (Houston, TX; Orlando, FL), Western Institutional Review Board (San Diego, CA; Detroit, MI; New Orleans, LA; New York, NY), WIRB - IBCS Services (Chicago, IL; New Orleans, LA; Oakland, CA; Minneapolis, MN; Columbus, OH; Lexington, KY), WJB Dorne VA Medical Center IBC (Columbia, SC).

Note that full information on the approval of the study protocol must also be provided in the manuscript.

Field-specific reporting

Please select the one below that is the best fit for your research. If you are not sure, read the appropriate sections before making your selection.

☒ Life sciences ☐ Behavioural & social sciences ☐ Ecological, evolutionary & environmental sciences

For a reference copy of the document with all sections, see nature.com/documents/nr-reporting-summary-flat.pdf

Life sciences study design

All studies must disclose on these points even when the disclosure is negative.

Sample size

Section 6 of the Statistical Analysis Plan for the first immune correlates analysis of the ENSEMBLE trial (Fong et al. 2022, Nature Microbiology) provides the following information:

The correlates analyses are initiated by the availability of (a) a data set defined at or after the primary analysis data set triggered by the accrual of a certain number of primary endpoints (approximately 150); and (b) Day 1, 29 antibody marker data from correlates-eligible COVID primary endpoint cases from at least 25 baseline seronegative vaccine recipients. The latter requirement ensures that there are enough endpoint cases to achieve worthwhile precision for CoR analyses. The HVTN 505 trial serves as a precedent where 25 evaluable vaccine recipient cases provided enough data to reasonably characterize correlates of risk for a preventive candidate HIV vaccine (Janes et al., 2017; Fong et al., 2018; Neidich et al., 2019; Gilbert et al., 2020b). In addition, simulation studies show that correlates analyses at 20 endpoints have notably lower precision.

Table 4 shows the minimum number of baseline seronegative vaccine recipient endpoints evaluable for correlates analyses that are required before conducting the various planned correlates analyses.

Table 4: Minimum Numbers of Evaluable Endpoints in baseline seronegative Vaccine Recipients to Initiate Correlates Analyses

CoRs (Risk Prediction Modeling)

a. (Semi)parametric models with strongly parametrized associations: Cox, hinge/threshold logistic regression N=25

b. Flexible parametric models: Generalized additive model N=35

c. Nonparametric thresholds: Donovan et al. (2019)/van der Laan et al. (2021) N=35

d. Superlearner estimated optimal surrogate Price et al. (2018) N=35

In the first correlates analysis (Fong et al.) the number of severe-critical endpoints was too small to assess CoRs against this endpoint; with the present analysis with increased follow-up duration there are sufficient severe-critical endpoints for CoR assessment in all regions pooled (42 severe-critical endpoints) and in Latin America (31 severe-critical endpoints) (endpoints in baseline SARS-CoV-2 seronegative per-protocol vaccine recipients with D1, D29 antibody data).

CoP: Correlates of VE, Controlled VE, Stochastic Interventional VE, Mediators of VE Each N=50

Our initial SAP specified the guideline that CoP analyses are best based on at least 50 vaccine breakthrough cases with the requisite available immune marker data. However, with 42 severe vaccine breakthrough cases with immune marker data available, there is still enough precision to conduct worthwhile CoP analyses, even when falling short of the recommended minimum of 50 cases. The ENSEMBLE trial is the only phase 3 trial in the US Government COVID-19 Response Team's set of trials that had anywhere near 50 severe breakthrough cases, and given the importance and unique opportunity to assess CoP against severe COVID-19, it was decided to go forward with the CoP analyses for this study outcome.

Data exclusions

For the binding antibody assay: plates and samples that did not meet the following quality control criteria were excluded:

	Plate calibrator curve fit r2 ≥ 0.98; calibrator replicate signal CV (coefficient of variation) ≤ 20%.<br>Plate controls signal CV (coefficient of variation) ≤ 20%; recoveries of plate controls within +/-20% of the nominal values.<br>Sample replicate CVs ≤ 20%.<br>For the immune correlates analyses: Correlates analyses were performed in per-protocol baseline SARS-CoV-2 seronegative participants, excluding participants with evidence of SARS-CoV-2 infection up to 6 days post-D29.
Replication	For the binding antibody assay, reproducibility was ensured by running high, medium, low, and negative controls on all plates assayed, and each test sera sample was added to precoated wells in duplicate (within a run) in an 8-point dilution series.<br>For the neutralizing antibody assay, test samples were assayed in singlicate titrations per plate. Each plate included a SARS-CoV-2 positive control, a SARS-CoV-2 negative control, a daily positive control, and a specificity control.<br>All of the immune correlates analyses are implemented in automated and reproducible press-button fashion.
Randomization	In the ENSEMBLE trial, participants were randomized in parallel in a 1:1 ratio to receive intramuscular (IM) injections of Ad26.COV2.S or placebo (as described in Sadoff et al. 2022 NEJM). Randomization was done with the use of randomly permuted blocks in an interactive Web-response system.
Blinding	The ENSEMBLE trial was a double-blinded phase 3 efficacy trial. The treatment arm assignment was blinded to the labs running the assays for the correlates analyses.

Reporting for specific materials, systems and methods

We require information from authors about some types of materials, experimental systems and methods used in many studies. Here, indicate whether each material, system or method listed is relevant to your study. If you are not sure if a list item applies to your research, read the appropriate section before selecting a response.

Materials & experimental systems

n/a	Involved in the study
☐	☒ Antibodies
☐	☒ Eukaryotic cell lines
☒	☐ Palaeontology and archaeology
☒	☐ Animals and other organisms
☐	☒ Clinical data
☒	☐ Dual use research of concern
☒	☐ Plants

Methods

n/a	Involved in the study
☒	☐ ChIP-seq
☒	☐ Flow cytometry
☒	☐ MRI-based neuroimaging

Antibodies

Antibodies used	MSD SULFO-TAGTM anti-human IgG detection antibody. Meso Scale Diagnostics, LLC. Catalog number R32AJ-1. Goat polyclonal antibody. Diluted to 1X from a 200X vendor-provided stock.
Validation	Certificates of analysis and technical notes are available at https://www.mesoscale.com/en/products/msd-gold-sulfo-tag-nhs-ester-r91ao/

Eukaryotic cell lines

Policy information about cell lines and Sex and Gender in Research

Cell line source(s)	The pseudovirus neutralization assay used the HEK 293 cell line, sourced from the Master Cell Bank (LC0027490) established by Monogram Biosciences in 2001.
Authentication	No formal authentication was performed. The HEK293 cell line has been in continuous use at Monogram Biosciences since 1996.
Mycoplasma contamination	Mycoplasma testing is routinely performed per Monogram Standard Operating Procedure.
Commonly misidentified lines (See ICLAC register)	None.

Clinical data

Policy information about clinical studies

All manuscripts should comply with the ICMJE guidelines for publication of clinical research and a completed CONSORT checklist must be included with all submissions.

Clinical trial registration	NCT04505722
Study protocol	Full trial protocol available with Sadoff et al. NEJM 2022: https://www.nejm.org/doi/suppl/10.1056/NEJMoa2117608/suppl_file/nejmoa2117608_protocol.pdf
Data collection	The trial began enrollment on September 21, 2020, and the data-cutoff date for the present analysis was July 9, 2021. Trial sites are listed in the Supplementary Appendix of Sadoff et al. 2021 (https://www.nejm.org/doi/suppl/10.1056/NEJMoa2101544/suppl_file/nejmoa2101544_appendix.pdf). Only participants at sites with access to appropriate processing facilities were considered for sampling into the immunogenicity subcohort. Serum samples were taken on D1 (day of injection) and on D29 for potential antibody measurements.
Outcomes	Moderate, severe-critical, and moderate to severe-critical COVID-19 endpoints were defined as in section 8.1.3.1 of the study protocol of Sadoff et al. (NEJM, 2022), available at https://www.nejm.org/doi/suppl/10.1056/NEJMoa2117608/suppl_file/nejmoa2117608_protocol.pdf, except with the differences outlined by Fong et al. (Nat Microbiol, 2022) in the “Trial design, study cohort, COVID primary endpoints and case/non-case definitions” section of Methods. However, the analysis included moderate, severe-critical, and moderate to severe-critical COVID-19 endpoints starting both ≥7 days (vs. ≥1 day, as in Fong et al.) post D29 and ≥28 days post vaccination up to the end of the correlates study period.

Plants

Seed stocks	N/A
Novel plant genotypes	N/A
Authentication	N/A
